# 31st Annual Meeting and Associated Programs of the Society for Immunotherapy of Cancer (SITC 2016): part one: National Harbor, MD, USA. 9-13 November 2016

**DOI:** 10.1186/s40425-016-0172-7

**Published:** 2016-11-16

**Authors:** Andreas Lundqvist, Vincent van Hoef, Xiaonan Zhang, Erik Wennerberg, Julie Lorent, Kristina Witt, Laia Masvidal Sanz, Shuo Liang, Shannon Murray, Ola Larsson, Rolf Kiessling, Yumeng Mao, John-William Sidhom, Catherine A. Bessell, Jonathan Havel, Jonathan Schneck, Timothy A. Chan, Eliot Sachsenmeier, David Woods, Anders Berglund, Rupal Ramakrishnan, Andressa Sodre, Jeffrey Weber, Roberta Zappasodi, Yanyun Li, Jingjing Qi, Philip Wong, Cynthia Sirard, Michael Postow, Walter Newman, Henry Koon, Vamsidhar Velcheti, Margaret K. Callahan, Jedd D. Wolchok, Taha Merghoub, Lawrence G. Lum, Minsig Choi, Archana Thakur, Abhinav Deol, Gregory Dyson, Anthony Shields, Cara Haymaker, Marc Uemura, Ravi Murthy, Marihella James, Daqing Wang, Julie Brevard, Catherine Monaghan, Suzanne Swann, James Geib, Mark Cornfeld, Srinivas Chunduru, Sudhir Agrawal, Cassian Yee, Jennifer Wargo, Sapna P. Patel, Rodabe Amaria, Hussein Tawbi, Isabella Glitza, Scott Woodman, Wen-Jen Hwu, Michael A. Davies, Patrick Hwu, Willem W. Overwijk, Chantale Bernatchez, Adi Diab, Erminia Massarelli, Neil H. Segal, Vincent Ribrag, Ignacio Melero, Tara C. Gangadhar, Walter Urba, Dirk Schadendorf, Robert L. Ferris, Roch Houot, Franck Morschhauser, Theodore Logan, Jason J. Luke, William Sharfman, Fabrice Barlesi, Patrick A. Ott, Laura Mansi, Shivaani Kummar, Gilles Salles, Cecilia Carpio, Roland Meier, Suba Krishnan, Dan McDonald, Matthew Maurer, Xuemin Gu, Jaclyn Neely, Satyendra Suryawanshi, Ronald Levy, Nikhil Khushalani, Jennifer Wu, Jinyu Zhang, Fahmin Basher, Mark Rubinstein, Mark Bucsek, Guanxi Qiao, Cameron MacDonald, Bonnie Hylander, Elizabeth Repasky, Shilpak Chatterjee, Anusara Daenthanasanmak, Paramita Chakraborty, Kyle Toth, Megan Meek, Elizabeth Garrett-Mayer, Michael Nishimura, Chrystal Paulos, Craig Beeson, Xuezhong Yu, Shikhar Mehrotra, Fei Zhao, Kathy Evans, Christine Xiao, Alisha Holtzhausen, Brent A. Hanks, Nicole Scharping, Ashley V. Menk, Rebecca Moreci, Ryan Whetstone, Rebekah Dadey, Simon Watkins, Robert Ferris, Greg M. Delgoffe, Jonathan Peled, Sean Devlin, Anna Staffas, Melissa Lumish, Kori Porosnicu Rodriguez, Katya Ahr, Miguel Perales, Sergio Giralt, Ying Taur, Eric Pamer, Marcel R. M. van den Brink, Robert Jenq, Nicola Annels, Hardev Pandha, Guy Simpson, Hugh Mostafid, Kevin Harrington, Alan Melcher, Mark Grose, Bronwyn Davies, Gough Au, Roberta Karpathy, Darren Shafren, Jacob Ricca, Taha Merghoub, Jedd D. Wolchok, Dmitriy Zamarin, Luciana Batista, Florence Marliot, Angela Vasaturo, Sabrina Carpentier, Cécile Poggionovo, Véronique Frayssinet, Jacques Fieschi, Marc Van den Eynde, Franck Pagès, Jérôme Galon, Fabienne Hermitte, Sean G. Smith, Khue Nguyen, Sruthi Ravindranathan, Bhanu Koppolu, David Zaharoff, Gustavo Schvartsman, Roland Bassett, Jennifer L. McQuade, Lauren E. Haydu, Michael A. Davies, Hussein Tawbi, Isabella Glitza, Douglas Kline, Xiufen Chen, Dominick Fosco, Justin Kline, Abigail Overacre, Maria Chikina, Erin Brunazzi, Gulidanna Shayan, William Horne, Jay Kolls, Robert L. Ferris, Greg M. Delgoffe, Tullia C. Bruno, Creg Workman, Dario Vignali, Prasad S. Adusumilli, Ephraim A Ansa-Addo, Zihai Li, Andrew Gerry, Joseph P. Sanderson, Karen Howe, Roslin Docta, Qian Gao, Eleanor A. L. Bagg, Nicholas Tribble, Miguel Maroto, Gareth Betts, Natalie Bath, Luca Melchiori, Daniel E. Lowther, Indu Ramachandran, Gabor Kari, Samik Basu, Gwendolyn Binder-Scholl, Karen Chagin, Lini Pandite, Tom Holdich, Rafael Amado, Hua Zhang, John Glod, Donna Bernstein, Bent Jakobsen, Crystal Mackall, Ryan Wong, Jonathan D. Silk, Katherine Adams, Garth Hamilton, Alan D. Bennett, Sara Brett, Junping Jing, Adriano Quattrini, Manoj Saini, Guy Wiedermann, Andrew Gerry, Bent Jakobsen, Gwendolyn Binder-Scholl, Joanna Brewer, MyLinh Duong, An Lu, Peter Chang, Aruna Mahendravada, Nicholas Shinners, Kevin Slawin, David M. Spencer, Aaron E. Foster, J. Henri Bayle, Cristina Bergamaschi, Sinnie Sin Man Ng, Bethany Nagy, Shawn Jensen, Xintao Hu, Candido Alicea, Bernard Fox, Barbara Felber, George Pavlakis, Jessica Chacon, Tori Yamamoto, Thomas Garrabrant, Luis Cortina, Daniel J. Powell, Marco Donia, Julie Westerlin Kjeldsen, Rikke Andersen, Marie Christine Wulff Westergaard, Valentina Bianchi, Mateusz Legut, Meriem Attaf, Garry Dolton, Barbara Szomolay, Sascha Ott, Rikke Lyngaa, Sine Reker Hadrup, Andrew Kelvin Sewell, Inge Marie Svane, Aaron Fan, Takumi Kumai, Esteban Celis, Ian Frank, Amanda Stramer, Michelle A. Blaskovich, Seth Wardell, Maria Fardis, James Bender, Michael T. Lotze, Stephanie L. Goff, Nikolaos Zacharakis, Yasmine Assadipour, Todd D. Prickett, Jared J. Gartner, Robert Somerville, Mary Black, Hui Xu, Harshini Chinnasamy, Isaac Kriley, Lily Lu, John Wunderlich, Paul F. Robbins, Steven Rosenberg, Steven A. Feldman, Kasia Trebska-McGowan, Isaac Kriley, Parisa Malekzadeh, Eden Payabyab, Richard Sherry, Steven Rosenberg, Stephanie L. Goff, Aishwarya Gokuldass, Michelle A. Blaskovich, Charlene Kopits, Brian Rabinovich, Michael T. Lotze, Daniel S. Green, Olena Kamenyeva, Kathryn C. Zoon, Christina M. Annunziata, Joanne Hammill, Christopher Helsen, Craig Aarts, Jonathan Bramson, Yui Harada, Yoshikazu Yonemitsu, Christopher Helsen, Joanne Hammill, Kenneth Mwawasi, Galina Denisova, Jonathan Bramson, Rajanish Giri, Benjamin Jin, Tracy Campbell, Lindsey M. Draper, Sanja Stevanovic, Zhiya Yu, Bianca Weissbrich, Nicholas P. Restifo, Cornelia L. Trimble, Steven Rosenberg, Christian S. Hinrichs, Kwong Tsang, Massimo Fantini, James W. Hodge, Rika Fujii, Ingrid Fernando, Caroline Jochems, Christopher Heery, James Gulley, Patrick Soon-Shiong, Jeffrey Schlom, Weiqing Jing, Jill Gershan, Grace Blitzer, James Weber, Laura McOlash, Bryon D. Johnson, Simin Kiany, Huang Gangxiong, Eugenie S. Kleinerman, Michael Klichinsky, Marco Ruella, Olga Shestova, Saad Kenderian, Miriam Kim, John Scholler, Carl H. June, Saar Gill, Duane Moogk, Shi Zhong, Zhiya Yu, Ivan Liadi, William Rittase, Victoria Fang, Janna Dougherty, Arianne Perez-Garcia, Iman Osman, Cheng Zhu, Navin Varadarajan, Nicholas P. Restifo, Alan Frey, Michelle Krogsgaard, Daniel Landi, Kristen Fousek, Malini Mukherjee, Ankita Shree, Sujith Joseph, Kevin Bielamowicz, Tiara Byrd, Nabil Ahmed, Meenakshi Hegde, Sylvia Lee, David Byrd, John Thompson, Shailender Bhatia, Scott Tykodi, Judy Delismon, Liz Chu, Siddiq Abdul-Alim, Arpy Ohanian, Anna Marie DeVito, Stanley Riddell, Kim Margolin, Isabelle Magalhaes, Jonas Mattsson, Michael Uhlin, Satoshi Nemoto, Patricio Pérez Villarroel, Ryosuke Nakagawa, James J. Mule, Adam W. Mailloux, Melinda Mata, Phuong Nguyen, Claudia Gerken, Christopher DeRenzo, David M. Spencer, Stephen Gottschalk, Mélissa Mathieu, Sandy Pelletier, John Stagg, Simon Turcotte, Nicholas Minutolo, Prannda Sharma, Andrew Tsourkas, Daniel J. Powell, Nadine Mockel-Tenbrinck, Daniela Mauer, Katharina Drechsel, Carola Barth, Katharina Freese, Ulrike Kolrep, Silke Schult, Mario Assenmacher, Andrew Kaiser, John Mullinax, MacLean Hall, Julie Le, Krithika Kodumudi, Erica Royster, Allison Richards, Ricardo Gonzalez, Amod Sarnaik, Shari Pilon-Thomas, Morten Nielsen, Anders Krarup-Hansen, Dorrit Hovgaard, Michael Mørk Petersen, Anand Chainsukh Loya, Niels Junker, Inge Marie Svane, Charlotte Rivas, Robin Parihar, Stephen Gottschalk, Cliona M. Rooney, Haiying Qin, Sang Nguyen, Paul Su, Chad Burk, Brynn Duncan, Bong-Hyun Kim, M. Eric Kohler, Terry Fry, Arjun A. Rao, Noam Teyssier, Jacob Pfeil, Nikolaos Sgourakis, Sofie Salama, David Haussler, Sarah A. Richman, Selene Nunez-Cruz, Zack Gershenson, Zissimos Mourelatos, David Barrett, Stephan Grupp, Michael Milone, Alba Rodriguez-Garcia, Matthew K. Robinson, Gregory P. Adams, Daniel J. Powell, João Santos, Riikka Havunen, Mikko Siurala, Víctor Cervera-Carrascón, Suvi Parviainen, Marjukka Antilla, Akseli Hemminki, Jyothi Sethuraman, Laurelis Santiago, Jie Qing Chen, Zhimin Dai, Seth Wardell, James Bender, Michael T. Lotze, Huizi Sha, Shu Su, Naiqing Ding, Baorui Liu, Sanja Stevanovic, Anna Pasetto, Sarah R. Helman, Jared J. Gartner, Todd D. Prickett, Paul F. Robbins, Steven A. Rosenberg, Christian S. Hinrichs, Shailender Bhatia, Melissa Burgess, Hui Zhang, Tien Lee, Hans Klingemann, Patrick Soon-Shiong, Paul Nghiem, John M. Kirkwood, John M. Rossi, Marika Sherman, Allen Xue, Yueh-wei Shen, Lynn Navale, Steven A. Rosenberg, James N. Kochenderfer, Adrian Bot, Anandaraman Veerapathran, Aishwarya Gokuldass, Amanda Stramer, Jyothi Sethuraman, Michelle A. Blaskovich, Doris Wiener, Ian Frank, Laurelis Santiago, Brian Rabinovich, Maria Fardis, James Bender, Michael T. Lotze, Edmund K. Waller, Jian-Ming Li, Christopher Petersen, Bruce R. Blazar, Jingxia Li, Cynthia R. Giver, Ziming Wang, Steven K. Grossenbacher, Ian Sturgill, Robert J. Canter, William J. Murphy, Congcong Zhang, Michael C. Burger, Lukas Jennewein, Anja Waldmann, Michel Mittelbronn, Torsten Tonn, Joachim P. Steinbach, Winfried S. Wels, Jason B. Williams, Yuanyuan Zha, Thomas F. Gajewski, LaTerrica C. Williams, Giedre Krenciute, Mamta Kalra, Chrystal Louis, Stephen Gottschalk, Gang Xin, David Schauder, Aimin Jiang, Nikhil Joshi, Weiguo Cui, Xue Zeng, Ashley V. Menk, Nicole Scharping, Greg M. Delgoffe, Zeguo Zhao, Mohamad Hamieh, Justin Eyquem, Gertrude Gunset, Neil Bander, Michel Sadelain, David Askmyr, Milad Abolhalaj, Kristina Lundberg, Lennart Greiff, Malin Lindstedt, Helen K. Angell, Kyoung-Mee Kim, Seung-Tae Kim, Sung Kim, Alan D. Sharpe, Julia Ogden, Anna Davenport, Darren R. Hodgson, Carl Barrett, Jeeyun Lee, Elaine Kilgour, Jodi Hanson, Richard Caspell, Alexey Karulin, Paul Lehmann, Tameem Ansari, Annemarie Schiller, Srividya Sundararaman, Paul Lehmann, Jodi Hanson, Diana Roen, Alexey Karulin, Paul Lehmann, Mark Ayers, Diane Levitan, Gladys Arreaza, Fang Liu, Robin Mogg, Yung-Jue Bang, Bert O’Neil, Razvan Cristescu, Philip Friedlander, Karl Wassman, Chrisann Kyi, William Oh, Nina Bhardwaj, Svetlana Bornschlegl, Michael P. Gustafson, Dennis A. Gastineau, Ian F. Parney, Allan B. Dietz, Daniel Carvajal-Hausdorf, Nikita Mani, Vamsidhar Velcheti, Kurt Schalper, David Rimm, Serena Chang, Ronald Levy, John Kurland, Suba Krishnan, Christoph Matthias Ahlers, Maria Jure-Kunkel, Lewis Cohen, Holden Maecker, Holbrook Kohrt, Shuming Chen, George Crabill, Theresa Pritchard, Tracee McMiller, Drew Pardoll, Fan Pan, Suzanne Topalian, Patrick Danaher, Sarah Warren, Lucas Dennis, Andrew M. White, Leonard D’Amico, Melissa Geller, Mary L. Disis, Joseph Beechem, Kunle Odunsi, Steven Fling, Roshanak Derakhshandeh, Tonya J. Webb, Sigrid Dubois, Kevin Conlon, Bonita Bryant, Jennifer Hsu, Nancy Beltran, Jürgen Müller, Thomas Waldmann, Rebekka Duhen, Thomas Duhen, Lucas Thompson, Ryan Montler, Andrew Weinberg, Max Kates, Brandon Early, Erik Yusko, Taylor H. Schreiber, Trinity J. Bivalacqua, Mark Ayers, Jared Lunceford, Michael Nebozhyn, Erin Murphy, Andrey Loboda, David R. Kaufman, Andrew Albright, Jonathan Cheng, S. Peter Kang, Veena Shankaran, Sarina A. Piha-Paul, Jennifer Yearley, Tanguy Seiwert, Antoni Ribas, Terrill K. McClanahan, Razvan Cristescu, Robin Mogg, Mark Ayers, Andrew Albright, Erin Murphy, Jennifer Yearley, Xinwei Sher, Xiao Qiao Liu, Michael Nebozhyn, Jared Lunceford, Andrew Joe, Jonathan Cheng, Elizabeth Plimack, Patrick A. Ott, Terrill K. McClanahan, Andrey Loboda, David R. Kaufman, Alex Forrest-Hay, Cheryl A. Guyre, Kohei Narumiya, Marc Delcommenne, Heather A. Hirsch, Amit Deshpande, Jason Reeves, Jenny Shu, Tong Zi, Jennifer Michaelson, Debbie Law, Elizabeth Trehu, Sriram Sathyanaryanan, Brendan P. Hodkinson, Natalie A. Hutnick, Michael E. Schaffer, Michael Gormley, Tyler Hulett, Shawn Jensen, Carmen Ballesteros-Merino, Christopher Dubay, Michael Afentoulis, Ashok Reddy, Larry David, Bernard Fox, Kumar Jayant, Swati Agrawal, Rajendra Agrawal, Ghayathri Jeyakumar, Seongho Kim, Heejin Kim, Cynthia Silski, Stacey Suisham, Elisabeth Heath, Ulka Vaishampayan, Natalie Vandeven, Natasja Nielsen Viller, Alison O’Connor, Hui Chen, Bolette Bossen, Eric Sievers, Robert Uger, Paul Nghiem, Lisa Johnson, Hsiang-Fong Kao, Chin-Fu Hsiao, Shu-Chuan Lai, Chun-Wei Wang, Jenq-Yuh Ko, Pei-Jen Lou, Tsai-Jan Lee, Tsang-Wu Liu, Ruey-Long Hong, Staci J. Kearney, Joshua C. Black, Benjamin J. Landis, Sally Koegler, Brooke Hirsch, Roberto Gianani, Jeffrey Kim, Ming-Xiao He, Bingqing Zhang, Nan Su, Yuling Luo, Xiao-Jun Ma, Emily Park, Dae Won Kim, Domenico Copploa, Nishi Kothari, Young doo Chang, Richard Kim, Namyong Kim, Melvin Lye, Ee Wan, Namyong Kim, Melvin Lye, Ee Wan, Namyong Kim, Melvin Lye, Ee Wan, Hanna A. Knaus, Sofia Berglund, Hubert Hackl, Judith E. Karp, Ivana Gojo, Leo Luznik, Henoch S. Hong, Sven D. Koch, Birgit Scheel, Ulrike Gnad-Vogt, Karl-Josef Kallen, Volker Wiegand, Linus Backert, Oliver Kohlbacher, Ingmar Hoerr, Mariola Fotin-Mleczek, James M. Billingsley, Yoshinobu Koguchi, Valerie Conrad, William Miller, Iliana Gonzalez, Tomasz Poplonski, Tanisha Meeuwsen, Ana Howells-Ferreira, Rogan Rattray, Mary Campbell, Carlo Bifulco, Christopher Dubay, Keith Bahjat, Brendan Curti, Walter Urba, E-K Vetsika, G. Kallergi, Despoina Aggouraki, Z. Lyristi, P. Katsarlinos, Filippos Koinis, V. Georgoulias, Athanasios Kotsakis, Nathan T. Martin, Famke Aeffner, Staci J. Kearney, Joshua C. Black, Logan Cerkovnik, Luke Pratte, Rebecca Kim, Brooke Hirsch, Joseph Krueger, Roberto Gianani, Amaia Martínez-Usatorre, Camilla Jandus, Alena Donda, Laura Carretero-Iglesia, Daniel E. Speiser, Dietmar Zehn, Nathalie Rufer, Pedro Romero, Anshuman Panda, Janice Mehnert, Kim M. Hirshfield, Greg Riedlinger, Sherri Damare, Tracie Saunders, Levi Sokol, Mark Stein, Elizabeth Poplin, Lorna Rodriguez-Rodriguez, Ann Silk, Nancy Chan, Melissa Frankel, Michael Kane, Jyoti Malhotra, Joseph Aisner, Howard L. Kaufman, Siraj Ali, Jeffrey Ross, Eileen White, Gyan Bhanot, Shridar Ganesan, Anne Monette, Derek Bergeron, Amira Ben Amor, Liliane Meunier, Christine Caron, Antigoni Morou, Daniel Kaufmann, Moishe Liberman, Igor Jurisica, Anne-Marie Mes-Masson, Kamel Hamzaoui, Rejean Lapointe, Ann Mongan, Yuan-Chieh Ku, Warren Tom, Yongming Sun, Alex Pankov, Tim Looney, Janice Au-Young, Fiona Hyland, Jeff Conroy, Carl Morrison, Sean Glenn, Blake Burgher, He Ji, Mark Gardner, Ann Mongan, Angela R. Omilian, Jeff Conroy, Wiam Bshara, Omilian Angela, Blake Burgher, He Ji, Sean Glenn, Carl Morrison, Ann Mongan, Joseph M. Obeid, Gulsun Erdag, Mark E. Smolkin, Donna H. Deacon, James W. Patterson, Lieping Chen, Timothy N. Bullock, Craig L. Slingluff, Joseph M. Obeid, Gulsun Erdag, Donna H. Deacon, Craig L. Slingluff, Timothy N. Bullock, John T. Loffredo, Raja Vuyyuru, Sophie Beyer, Vanessa M. Spires, Maxine Fox, Jon M. Ehrmann, Katrina A. Taylor, Alan J. Korman, Robert F. Graziano, David Page, Katherine Sanchez, Carmen Ballesteros-Merino, Maritza Martel, Carlo Bifulco, Walter Urba, Bernard Fox, Sapna P. Patel, Mariana Petaccia De Macedo, Yong Qin, Alex Reuben, Christine Spencer, Michele Guindani, Roland Bassett, Jennifer Wargo, Adriana Racolta, Brian Kelly, Tobin Jones, Nathan Polaske, Noah Theiss, Mark Robida, Jeffrey Meridew, Iva Habensus, Liping Zhang, Lidija Pestic-Dragovich, Lei Tang, Ryan J. Sullivan, Theodore Logan, Nikhil Khushalani, Kim Margolin, Henry Koon, Thomas Olencki, Thomas Hutson, Brendan Curti, Joanna Roder, Shauna Blackmon, Heinrich Roder, John Stewart, Asim Amin, Marc S. Ernstoff, Joseph I. Clark, Michael B. Atkins, Howard L. Kaufman, Jeffrey Sosman, Jeffrey Weber, David F. McDermott, Jeffrey Weber, Harriet Kluger, Ruth Halaban, Mario Snzol, Heinrich Roder, Joanna Roder, Senait Asmellash, Arni Steingrimsson, Shauna Blackmon, Ryan J. Sullivan, Chichung Wang, Kristin Roman, Amanda Clement, Sean Downing, Clifford Hoyt, Nathalie Harder, Guenter Schmidt, Ralf Schoenmeyer, Nicolas Brieu, Mehmet Yigitsoy, Gabriele Madonna, Gerardo Botti, Antonio Grimaldi, Paolo A. Ascierto, Ralf Huss, Maria Athelogou, Harald Hessel, Nathalie Harder, Alexander Buchner, Guenter Schmidt, Christian Stief, Ralf Huss, Gerd Binnig, Thomas Kirchner, Shankar Sellappan, Sheeno Thyparambil, Sarit Schwartz, Fabiola Cecchi, Andrew Nguyen, Charles Vaske, Todd Hembrough, Jan Spacek, Michal Vocka, Eva Zavadova, Helena Skalova, Pavel Dundr, Lubos Petruzelka, Nicole Francis, Rau T. Tilman, Arndt Hartmann, Irena Netikova, Carmen Ballesteros-Merino, Julia Stump, Amanda Tufman, Frank Berger, Michael Neuberger, Rudolf Hatz, Michael Lindner, Rachel E. Sanborn, John Handy, Bernard Fox, Carlo Bifulco, Rudolf M. Huber, Hauke Winter, Simone Reu, Cheng Sun, Weihua Xiao, Zhigang Tian, Kshitij Arora, Niyati Desai, Anupriya Kulkarni, Mihir Rajurkar, Miguel Rivera, Vikram Deshpande, David Ting, Katy Tsai, Adi Nosrati, Simone Goldinger, Omid Hamid, Alain Algazi, Paul Tumeh, Jimmy Hwang, Jacqueline Liu, Lawrence Chen, Reinhard Dummer, Michael Rosenblum, Adil Daud, Tsu-Shuen Tsao, Julia Ashworth-Sharpe, Donald Johnson, Srabani Bhaumik, Christopher Bieniarz, Joseph Couto, Michael Farrell, Mahsa Ghaffari, Iva Habensus, Antony Hubbard, Tobin Jones, Brian Kelly, Jerome Kosmeder, Cleo Lee, Erin Marner, Jeffrey Meridew, Nathan Polaske, Adriana Racolta, Diana Uribe, Hongjun Zhang, Jian Zhang, Wenjun Zhang, Yifei Zhu, Larry Morrison, Lidija Pestic-Dragovich, Lei Tang, Takahiro Tsujikawa, Rohan N. Borkar, Vahid Azimi, Sushil Kumar, Guillaume Thibault, Motomi Mori, Edward El Rassi, Daniel R. Clayburgh, Molly F. Kulesz-Martin, Paul W. Flint, Lisa M. Coussens, Lisa Villabona, Giuseppe V. Masucci, Gary Geiss, Brian Birditt, Qian Mei, Alan Huang, Andrew M. White, Maribeth A. Eagan, Eduardo Ignacio, Nathan Elliott, Dwayne Dunaway, Lucas Dennis, Sarah Warren, Joseph Beechem, Dwayne Dunaway, Jaemyeong Jung, Chris Merritt, Isaac Sprague, Philippa Webster, Yan Liang, Sarah Warren, Joseph Beechem, Jessica Wenthe, Gunilla Enblad, Hannah Karlsson, Magnus Essand, Barbara Savoldo, Gianpietro Dotti, Martin Höglund, Malcolm K. Brenner, Hans Hagberg, Angelica Loskog, Matthew J. Bernett, Gregory L. Moore, Michael Hedvat, Christine Bonzon, Seung Chu, Rumana Rashid, Kendra N. Avery, Umesh Muchhal, John Desjarlais, Michael Hedvat, Matthew J. Bernett, Gregory L. Moore, Christine Bonzon, Rumana Rashid, Seung Chu, Kendra N. Avery, Umesh Muchhal, John Desjarlais, Matthew Kraman, Katarzyna Kmiecik, Natalie Allen, Mustapha Faroudi, Carlo Zimarino, Mateusz Wydro, Jacqueline Doody, Sreesha P. Srinivasa, Nagaraja Govindappa, Praveen Reddy, Aparajita Dubey, Sankar Periyasamy, Madhukara Adekandi, Chaitali Dey, Mary Joy, Pieter Fokko van Loo, Henrike Veninga, Setareh Shamsili, Mark Throsby, Harry Dolstra, Lex Bakker, Ajjai Alva, Juergen Gschwendt, Yohann Loriot, Joaquim Bellmunt, Dai Feng, Christian Poehlein, Thomas Powles, Emmanuel S. Antonarakis, Charles G. Drake, Haiyan Wu, Christian Poehlein, Johann De Bono, Rajat Bannerji, John Byrd, Gareth Gregory, Stephen Opat, Jake Shortt, Andrew J. Yee, Noopur Raje, Seth Thompson, Arun Balakumaran, Shaji Kumar, Brian I. Rini, Toni K. Choueiri, Mariangela Mariani, Laurence Albiges, John B. Haanen, Michael B. Atkins, James Larkin, Manuela Schmidinger, Domenico Magazzù, Alessandra di Pietro, Robert J. Motzer, Troels Holz Borch, Rikke Andersen, Per Kongsted, Magnus Pedersen, Morten Nielsen, Özcan Met, Marco Donia, Inge Marie Svane, Karim Boudadi, Hao Wang, James Vasselli, Jan E. Baughman, Jon Wigginton, Rehab Abdallah, Ashley Ross, Charles G. Drake, Emmanuel S. Antonarakis, Robert J. Canter, Jiwon Park, Ziming Wang, Steven Grossenbacher, Jesus I. Luna, Sita Withers, William Culp, Mingyi Chen, Arta Monjazeb, Michael S. Kent, William J. Murphy, Smita Chandran, Robert Somerville, John Wunderlich, David Danforth, James Yang, Richard Sherry, Christopher Klebanoff, Stephanie Goff, Biman Paria, Arvind Sabesan, Abhishek Srivastava, Steven A. Rosenberg, Udai Kammula, Brendan Curti, Jon Richards, Mark Faries, Robert H. I. Andtbacka, Mark Grose, Darren Shafren, Luis A. Diaz, Dung T. Le, Takayuki Yoshino, Thierry André, Johanna Bendell, Minori Koshiji, Yayan Zhang, S Peter Kang, Bao Lam, Dirk Jäger, Todd M. Bauer, Judy S. Wang, Jean K. Lee, Gulam A. Manji, Ragini Kudchadkar, John S. Kauh, Shande Tang, Naomi Laing, Gerald Falchook, Edward B. Garon, Balazs Halmos, Hui Rina, Natasha Leighl, Sung Sook Lee, William Walsh, Konstanin Dragnev, Bilal Piperdi, Luis Paz-Ares Rodriguez, Nabeegha Shinwari, Ziewn Wei, Michael P. Gustafson, Mary L Maas, Michael Deeds, Adam Armstrong, Svetlana Bornschlegl, Tim Peterson, Sue Steinmetz, Dennis A. Gastineau, Ian F. Parney, Allan B. Dietz, Thomas Herzog, Floor J. Backes, Larry Copeland, Maria Del Pilar Estevez Diz, Thomas W. Hare, Warner Huh, Byoung-Gie Kim, Kathleen M. Moore, Ana Oaknin, William Small, Krishnansu S. Tewari, Bradley J. Monk, Ashish M. Kamat, Joaquim Bellmunt, Toni K. Choueiri, Kijoeng Nam, Maria De Santis, Robert Dreicer, Noah M. Hahn, Rodolfo Perini, Arlene Siefker-Radtke, Guru Sonpavde, Ronald de Wit, J. Alfred Witjes, Stephen Keefe, Dean Bajorin, Justin Kline, Philippe Armand, John Kuruvilla, Craig Moskowitz, Mehdi Hamadani, Vincent Ribrag, Pier Luigi Zinzani, Sabine Chlosta, Seth Thompson, Arun Balakumaran, Nancy Bartlett, Chrisann Kyi, Rachel Sabado, Yvonne Saenger, Loging William, Michael Joseph Donovan, Erlinda Sacris, John Mandeli, Andres M. Salazar, Philip Friedlander, Nina Bhardwaj, John Powderly, Joshua Brody, John Nemunaitis, Leisha Emens, Jason J. Luke, Amita Patnaik, Ian McCaffery, Richard Miller, Ginna Laport, Andrew L. Coveler, David C. Smith, Juneko E. Grilley-Olson, Thomas F. Gajewski, Sanjay Goel, Shyra J. Gardai, Che-Leung Law, Gary Means, Thomas Manley, Brendan Curti, Kristen A. Marrone, Gary Rosner, Valsamo Anagnostou, Joanne Riemer, Jessica Wakefield, Cynthia Zanhow, Stephen Baylin, Barbara Gitlitz, Julie Brahmer, David F. McDermott, Sabina Signoretti, Wenting Li, Charles Schloss, Jean-Marie Michot, Philippe Armand, Wei Ding, Vincent Ribrag, Beth Christian, Arun Balakumaran, Patricia Marinello, Sabine Chlosta, Yayan Zhang, Margaret Shipp, Pier Luigi Zinzani, Yana G. Najjar, Lin, Lisa H. Butterfield, Ahmad A. Tarhini, Diwakar Davar, Hassane Zarour, Elizabeth Rush, Cindy Sander, John M. Kirkwood, Siqing Fu, Todd Bauer, Chris Molineaux, Mark K. Bennett, Keith W. Orford, Kyriakos P. Papadopoulos, Sukhmani K. Padda, Sumit A. Shah, A Dimitrios Colevas, Sujata Narayanan, George A. Fisher, Dana Supan, Heather A. Wakelee, Rhonda Aoki, Mark D. Pegram, Victor M. Villalobos, Jie Liu, Chris H. Takimoto, Mark Chao, Jens-Peter Volkmer, Ravindra Majeti, Irving L. Weissman, Branimir I. Sikic, David Page, Wendy Yu, Alison Conlin, Janet Ruzich, Stacy Lewis, Anupama Acheson, Kathleen Kemmer, Kelly Perlewitz, Nicole M. Moxon, Staci Mellinger, Carlo Bifulco, Maritza Martel, Yoshinobu Koguchi, Bernard Fox, Walter Urba, Heather McArthur, Magnus Pedersen, Marie Christine Wulff Westergaard, Troels Holz Borch, Morten Nielsen, Per Kongsted, Trine Juhler-Nøttrup, Marco Donia, Inge Marie Svane, Jayesh Desai, Ben Markman, Shahneen Sandhu, Hui Gan, Michael L. Friedlander, Ben Tran, Tarek Meniawy, Joanne Lundy, Duncan Colyer, Malaka Ameratunga, Christie Norris, Jason Yang, Kang Li, Lai Wang, Lusong Luo, Zhen Qin, Song Mu, Xuemei Tan, James Song, Michael Millward, Matthew H. G. Katz, Todd W. Bauer, Gauri R. Varadhachary, Nicolas Acquavella, Nipun Merchant, Gina Petroni, Craig L. Slingluff, Osama E. Rahma, Brian I. Rini, Thomas Powles, Mei Chen, Yang Song, Markus Puhlmann, Michael B. Atkins, Sriram Sathyanaryanan, Heather A. Hirsch, Jenny Shu, Amit Deshpande, Arun Khattri, Jason Reeves, Tong Zi, Ryan Brisson, Christopher Harvey, Jennifer Michaelson, Debbie Law, Tanguy Seiwert, Jatin Shah, Maria Victoria Mateos, Morio Matsumoto, Hilary Blacklock, Albert Oriol Rocafiguera, Hartmut Goldschmidt, Shinsuke Iida, Dina Ben Yehuda, Enrique Ocio, Paula Rodríguez-Otero, Sundar Jagannath, Sagar Lonial, Uma Kher, Patricia Marinello, Jesus San-Miguel, Jatin Shah, Sagar Lonial, Moacyr Ribeiro de Oliveira, Habte Yimer, Maria Victoria Mateos, Robert Rifkin, Fredrik Schjesvold, Enrique Ocio, Paula Rodríguez-Otero, Jesus San-Miguel, Razi Ghori, Patricia Marinello, Sundar Jagannath, Anna Spreafico, Victor Lee, Roger K. C. Ngan, Ka Fai To, Myung Ju Ahn, Quan Sing Ng, Ruey-Long Hong, Jin-Ching Lin, Ramona F. Swaby, Christine Gause, Sanatan Saraf, Anthony T. C. Chan, Elaine Lam, Nizar M. Tannir, Funda Meric-Bernstam, Ulka Vaishampayan, Keith W. Orford, Chris Molineaux, Matt Gross, Andy MacKinnon, Sam Whiting, Martin Voss, Evan Y. Yu, Haiyan Wu, Charles Schloss, Mark R. Albertini, Erik A. Ranheim, Jacquelyn A. Hank, Cindy Zuleger, Thomas McFarland, Jennifer Collins, Erin Clements, Sharon Weber, Tracey Weigel, Heather Neuman, Greg Hartig, David Mahvi, MaryBeth Henry, Jacek Gan, Richard Yang, Lakeesha Carmichael, KyungMann Kim, Stephen D. Gillies, Paul M. Sondel, Vivek Subbiah, Ravi Murthy, Lori Noffsinger, Kyle Hendricks, Marnix Bosch, Jay M. Lee, Mi-Heon Lee, Edward B. Garon, Jonathan W. Goldman, Felicita E. Baratelli, Dorthe Schaue, Gerald Wang, Frances Rosen, Jane Yanagawa, Tonya C. Walser, Ying Q. Lin, Sharon Adams, Franco M. Marincola, Paul C. Tumeh, Fereidoun Abtin, Robert Suh, Karen Reckamp, William D. Wallace, Gang Zeng, David A. Elashoff, Sherven Sharma, Steven M. Dubinett, Nina Bhardwaj, Philip Friedlander, Anna C. Pavlick, Marc S. Ernstoff, Brian Gastman, Brent Hanks, Mark R. Albertini, Jason J. Luke, Tibor Keler, Tom Davis, Laura A. Vitale, Elad Sharon, Patrick Danaher, Chihiro Morishima, Martin Cheever, Steven Fling, Christopher R. Heery, Joseph W. Kim, Elizabeth Lamping, Jennifer Marte, Sheri McMahon, Lisa Cordes, Farhad Fakhrejahani, Ravi Madan, Kwong Tsang, Caroline Jochems, Rachel Salazar, Maggie Zhang, Christoph Helwig, Jeffrey Schlom, James L Gulley, Roger Li, John Amrhein, Zvi Cohen, Monique Champagne, Ashish Kamat, M. Angela Aznar, Sara Labiano, Angel Diaz-Lagares, Manel Esteller, Juan Sandoval, Ignacio Melero, Susannah D. Barbee, David I. Bellovin, John C. Timmer, Nebiyu Wondyfraw, Susan Johnson, Johanna Park, Amanda Chen, Mikayel Mkrtichyan, Amir S. Razai, Kyle S. Jones, Chelsie Y. Hata, Denise Gonzalez, Quinn Deveraux, Brendan P. Eckelman, Luis Borges, Rukmini Bhardwaj, Raj K. Puri, Akiko Suzuki, Pamela Leland, Bharat H. Joshi, Todd Bartkowiak, Ashvin Jaiswal, Casey Ager, Midan Ai, Pratha Budhani, Renee Chin, David Hong, Michael Curran, William D. Hastings, Maria Pinzon-Ortiz, Masato Murakami, Jason R. Dobson, David Quinn, Joel P. Wagner, Xianhui Rong, Pamela Shaw, Ernesta Dammassa, Wei Guan, Glenn Dranoff, Alexander Cao, Ross B. Fulton, Steven Leonardo, Kathryn Fraser, Takashi O. Kangas, Nadine Ottoson, Nandita Bose, Richard D. Huhn, Jeremy Graff, Jamie Lowe, Keith Gorden, Mark Uhlik, Laura A. Vitale, Thomas O’Neill, Jenifer Widger, Andrea Crocker, Li-Zhen He, Jeffrey Weidlick, Karuna Sundarapandiyan, Venky Ramakrishna, James Storey, Lawrence J. Thomas, Joel Goldstein, Henry C. Marsh, Tibor Keler, Jamison Grailer, Julia Gilden, Pete Stecha, Denise Garvin, Jim Hartnett, Frank Fan, Mei Cong, Zhi-jie Jey Cheng, Marlon J. Hinner, Rachida-Siham Bel Aiba, Corinna Schlosser, Thomas Jaquin, Andrea Allersdorfer, Sven Berger, Alexander Wiedenmann, Gabriele Matschiner, Julia Schüler, Ulrich Moebius, Christine Rothe, Olwill A. Shane, Brendan Horton, Stefani Spranger, Thomas F. Gajewski, Dayson Moreira, Tomasz Adamus, Xingli Zhao, Piotr Swiderski, Sumanta Pal, Marcin Kortylewski, Alyssa Kosmides, Kevin Necochea, Jonathan Schneck, Kathleen M. Mahoney, Sachet A. Shukla, Nikolaos Patsoukis, Apoorvi Chaudhri, Hung Pham, Ping Hua, Xia Bu, Baogong Zhu, Nir Hacohen, Catherine J. Wu, Edward Fritsch, Vassiliki A. Boussiotis, Gordon J. Freeman, Amy E. Moran, Fanny Polesso, Lisa Lukaesko, Andrew Weinberg, Emelie Rådestad, Lars Egevad, Jonas Mattsson, Berit Sundberg, Lars Henningsohn, Victor Levitsky, Michael Uhlin, William Rafelson, John L. Reagan, Loren Fast, Pottayil Sasikumar, Naremaddepalli Sudarshan, Raghuveer Ramachandra, Nagesh Gowda, Dodheri Samiulla, Talapaneni Chandrasekhar, Sreenivas Adurthi, Jiju Mani, Rashmi Nair, Amit Dhudashia, Nagaraj Gowda, Murali Ramachandra, Alexander Sankin, Benjamin Gartrell, Kerwin Cumberbatch, Hongying Huang, Joshua Stern, Mark Schoenberg, Xingxing Zang, Ryan Swanson, Michael Kornacker, Lawrence Evans, Erika Rickel, Martin Wolfson, Sandrine Valsesia-Wittmann, Tala Shekarian, François Simard, Rodrigo Nailo, Aurélie Dutour, Anne-Catherine Jallas, Christophe Caux, Aurélien Marabelle

**Affiliations:** 1 Karolinska Institutet, Stockholm, Stockholms Lan Sweden; 2 Weill Cornell Medical College, New York, NY USA; 3 Nova Southeastern University, Cell Therapy Institute, Fort Lauderdale, FL USA; 4 Johns Hopkins University School of Medicine, Baltimore, MD USA; 5 Immunology Program, Johns Hopkins University, School of Medicine, Columbia, MD USA; 6 Memorial Sloan Kettering Cancer Center, New York, NY USA; 7 Johns Hopkins Medical Institute, Baltimore, MD USA; 8 University of Rochester, Monrovia, MD USA; 9 NYU Langone Medical Center, New York, NY USA; 10 H. Lee Moffitt Cancer Center, Tampa, FL USA; 11 Ludwig Collaborative Laboratory, Memorial Sloan Kettering Cancer Center, New York, NY USA; 12 Immune Monitoring Core Facility, Memorial Sloan Kettering Cancer Center, New York, NY USA; 13 Leap Therapeutics, Cambridge, MA USA; 14 Department of Medicine, Memorial Sloan Kettering Cancer Center, New York, NY USA; 15 Case Western Reserve University, Cleveland, OH USA; 16 Cleveland Clinic Main Campus, Cleveland, OH USA; 17 Memorial Sloan Kettering Cancer Center, New York, NY USA; 18 University of Virginia Cancer Center, Charlottesville, VA USA; 19 Stony Brook University Medical Center, Stony Brook, NY USA; 20 Karmanos Cancer Institute, Detroit, MI USA; 21 University of Texas MD Anderson Cancer Center, Houston, TX USA; 22 Idera Pharmaceuticals, Inc., Cambridge, MA USA; 23 University of Texas MD Anderson Cancer Center, Houston, TX USA; 24 Memorial Sloan Kettering Cancer Center, New York, NY USA; 25 Institut Gustave Roussy, Villejuif, Ile-de-France France; 26 Center for Applied Medical Research (CIMA), University of Navarra, Pamplona, Navarra Spain; 27 University of Pennsylvania, Philadelphia, PA USA; 28 Earle A. Chiles Research Institute, Providence Cancer Center, Portland, OR USA; 29 Universitätsklinikum Essen, Essen, Nordrhein-Westfalen, Germany; 30 University of Pittsburgh, Pittsburgh, PA USA; 31 CHU Rennes, Service Hématologie Clinique and INSERM 0203, Unité d’Investigation Clinique, Rennes, Bretagne France; 32 Centre Hospitalier Régional Universitaire de Lille, Lille, Nord-Pas-de-Calais France; 33 Simon Cancer Center, Indiana University, Indianapolis, IN USA; 34 University of Chicago School of Medicine, Chicago, IL USA; 35 Johns Hopkins University School of Medicine, Lutherville, MD USA; 36 Multidisciplinary Oncology and Therapeutic Innovations, Hôpital Nord, Marseille, Provence-Alpes-Cote d’Azur France; 37 Dana-Farber Cancer Institute, Boston, MA USA; 38 Centre Hospitalier Régional Universitaire Hôpital Jean Minjoz, Besançon, Franche-Comte France; 39 Stanford University School of Medicine, Stanford, CA USA; 40 Hospices Civils de Lyon-Université de Lyon, Pierre Benite, Auvergne France; 41 Hospital Universitari Vall d’Hebron, Universitat Autònoma de Barcelona, Barcelona, Catalonia Spain; 42 Bristol-Myers Squibb, Princeton, NJ USA; 43 H. Lee Moffitt Cancer Center, Tampa, FL USA; 44 Medical University of South Carolina, Charleston, SC USA; 45 Roswell Park Cancer Institute, Buffalo, NY USA; 46 MUSC, Charleston, SC USA; 47 Loyola Cancer Center, Maywood, IL USA; 48 Duke University Medical Center, Durham, NC USA; 49 Lineberger Comprehensive Cancer Center, University of North Carolina, Chapel Hill, NC USA; 50 University of Pittsburgh, Pittsburgh, PA USA; 51 University of Pittsburgh Cancer Institute, Pittsburgh, PA USA; 52 Memorial Sloan Kettering Cancer Center, New York, NY USA; 53 University of Surrey, Guildford, England, UK; 54 Royal Surrey County Hospital, Guildford, England, UK; 55 Institute for Cancer Research, London, England, UK; 56 The Institute for Cancer Research, London, England, UK; 57 Viralytics, Inc., Sydney, New South Wales Australia; 58 Memorial Sloan Kettering Cancer Center, New York, NY USA; 59 Ludwig Collaborative Laboratory, Memorial Sloan Kettering Cancer Center, New York, NY USA; 60 Department of Medicine, Memorial Sloan Kettering Cancer Center, New York, NY USA; 61 HalioDx, Marseille, Provence-Alpes-Cote d’Azur France; 62 Université Paris Descartes, APHP, Paris, Ile-de-France France; 63 INSERM, Paris, Ile-de-France France; 64 MI-mAbs, Marseille, Provence-Alpes-Cote d’Azur France; 65 Université Catholique de Louvain, Brussels, Brussels, Hoofdstedelijk Gewest Belgium; 66 APHP, Paris, Ile-de-France France; 67 Joint Department of Biomedical Engineering, North Carolina State University and the University of North Carolina, Raleigh, NC, Cary, NC USA; 68 Cell and Molecular Biology, University of Arkansas, Fayetteville, AR USA; 69 Biomedical Engineering, University of Arkansas, Fayetteville, AR USA; 70 University of Texas MD Anderson Cancer Center, Houston, TX USA; 71 Committee on Immunology, University of Chicago, Chicago, IL USA; 72 Department of Medicine, University of Chicago, Chicago, IL USA; 73 Committee on Immunology and Department of Medicine, University of Chicago, Chicago, IL USA; 74 University of Pittsburgh, Pittsburgh, PA USA; 75 Tsinghua University, Pittsburgh, PA USA; 76 Department of Immunology, University of Pittsburgh, Pittsburgh, PA USA; 77 Memorial Sloan Kettering Cancer Center, New York, NY USA; 78 Medical University of South Carolina, Charleston, SC USA; 79 Adaptimmune, Oxfordshire, England UK; 80 Adaptimmune, Philadelphia, PA USA; 81 National Cancer Institute, Bethesda, MD USA; 82 Adaptimmune, Oxfordshire, England, UK; 83 Stanford University School of Medicine, Stanford, CA USA; 84 Adaptimmune, Oxfordshire, England, UK; 85 GSK, Stevenage, England, UK; 86 Adaptimmune, Philadelphia, PA USA; 87 Bellicum Pharmaceuticals, Houston, TX USA; 88 Bellicum Pharmaceuticals and Baylor College of Medicine, Houston, TX USA; 89 National Cancer Institute at Frederick, Frederick, MD USA; 90 Providence Cancer Center, Portland, OR USA; 91 University of Pennsylvania, Philadelphia, PA USA; 92 Department of Pathology and Laboratory Medicine, Perelman School of Medicine, University of Pennsylvania, Philadelphia, PA USA; 93 Department of Oncology, Center for Cancer Immune Therapy, Herlev Hospital, Herlev, Hovedstaden Denmark; 94 Center for Cancer Immune Therapy, Herlev Hospital, Herlev, Hovedstaden Denmark; 95 Cardiff University School of Medicine, Cardiff, Wales UK; 96 Systems Immunology Institute, Cardiff University, Cardiff, Wales UK; 97 Warwick Systems Biology Centre, University of Warwick, Coventry, England UK; 98 Section for Immunology and Vaccinology, National Veterinary Institute, Technical University of Denmark, Frederiksberg, Hovedstaden Denmark; 99 Augusta University, Augusta, GA USA; 100 Georgia Cancer Center, Augusta University, Augusta, GA USA; 101 Lion Biotechnologies, Inc., Tampa, FL USA; 102 Surgery Branch, National Cancer Institute, National Institutes of Health, Bethesda, MD USA; 103 National Cancer Institute, Bethesda, MD USA; 104 Surgery Branch, National Cancer Institute, National Institutes of Health, Bethesda, MD USA; 105 National Cancer Institute, Bethesda, MD USA; 106 Lion Biotechnologies, Inc., Tampa, FL USA; 107 Translational Genomics Section, Women’s Malignancy Branch, National Cancer Institute, Bethesda, MD USA; 108 Biological Imaging Section, Research Technologies Branch, National Institute of Allergy and Infectious Disease, National Institutes of Health, Bethesda, MD USA; 109 Laboratory of Infectious Diseases, National Institute of Allergy and Infectious Disease, National Institutes of Health, Bethesda, MD USA; 110 McMaster University, Hamilton, ON Canada; 111 Kyushu University, Fukuoka, Fukuoka Japan; 112 McMaster University, Hamilton, ON Canada; 113 Indian Institute of Technology Mandi, Mandi, India; 114 Experimental Transplantation and Immunology Branch, National Cancer Institute, Bethesda, MD USA; 115 National Cancer Institute, Bethesda, MD USA; 116 Surgery Branch, National Cancer Institute, Bethesda, MD USA; 117 Kite Pharma EU, Amsterdam, Noord-Holland Netherlands; 118 Johns Hopkins University, Baltimore, MD USA; 119 Laboratory of Tumor Immunology and Biology, National Cancer Institute, National Institutes of Health, Bethesda, MD USA; 120 National Cancer Institute, National Institutes of Health, Bethesda, MD USA; 121 NantKwest, Inc., Culver City, CA USA; 122 Center for Cancer Research, National Cancer Institute, National Institutes of Health, Bethesda, MD USA; 123 Medical College of Wisconsin, Milwaukee, WI USA; 124 University of Texas MD Anderson Cancer Center, Houston, TX USA; 125 Center for Cellular Immunotherapies, University of Pennsylvania, Philadelphia, PA USA; 126 Perlmutter Cancer Center, NYU School of Medicine, New York, NY USA; 127 Xiangxue Pharmaceutical Co., Ltd., Guangdong, Guizhou People’s Republic of China; 128 Surgery Branch, National Cancer Institute, Bethesda, MD USA; 129 University of Houston, Houston, TX USA; 130 The George W. Woodruff School of Mechanical Engineering, Georgia Institute of Technology, Atlanta, GA USA; 131 Skirball Institute of Biomolecular Medicine, NYU School of Medicine, New York, NY USA; 132 Kite Pharma, Inc., Santa Monica, CA USA; 133 Ronald O. Perelman Department of Dermatology and Department of Medicine and Urology, Perlmutter Cancer Center at NYU School of Medicine, New York, NY USA; 134 New York University Langone School of Medicine, New York, NY USA; 135 Department of Pathology and Perlmutter Cancer Center at NYU School of Medicine, New York, NY USA; 136 Baylor College of Medicine, Houston, TX USA; 137 Seattle Cancer Care Alliance, Fred Hutchinson Cancer Research Center, University of Washington, Seattle, WA USA; 138 University of Washington, Seattle, WA USA; 139 Clinical Research Division at Fred Hutch Cancer Center, University of Washington, Seattle, WA USA; 140 Fred Hutchinson Cancer Research Center, University of Washington, Seattle, WA USA; 141 Fred Hutchinson Cancer Research Center, Seattle, WA USA; 142 Seattle Cancer Care Alliance, Seattle, WA USA; 143 Department of Medical Oncology, City Of Hope, Duarte, CA USA; 144 Karolinska Institutet, Stockholm, Stockholms Lan Sweden; 145 Translational Science, H. Lee Moffitt Cancer Center, Tampa, FL USA; 146 Immunology Program, Cutaneous Oncology Program, H. Lee Moffitt Cancer Center, Tampa, FL USA; 147 Baylor College of Medicine, Center for Cell and Gene Therapy, Houston, TX USA; 148 Bellicum Pharmaceuticals and Baylor College of Medicine, Houston, TX USA; 149 CRCHUM, Montréal, PQ Canada; 150 Department of Pathology and Laboratory Medicine, Perelman School of Medicine, University of Pennsylvania, Philadelphia, PA USA; 151 Department of Bioengineering, University of Pennsylvania, Philadelphia, PA USA; 152 Miltenyi Biotec, Bergisch Gladbach, Nordrhein-Westfalen, Germany; 153 H. Lee Moffitt Cancer Center, Tampa, FL USA; 154 Center for Cancer Immune Therapy and Department of Oncology, Herlev Hospital, Herlev, Hovedstaden Denmark; 155 Department of Oncology, Herlev University Hospital, Herlev, Hovedstaden Denmark; 156 Department of Orthopaedic Surgery, Copenhagen University Hospital, Copenhagen, Hovedstaden Denmark; 157 Department of Pathology, Copenhagen University Hospital, Copenhagen, Hovedstaden Denmark; 158 Department of Oncology, Center for Cancer Immune Therapy, Herlev University Hospital, Herlev, Hovedstaden Denmark; 159 Baylor College of Medicine, Houston, TX USA; 160 Center for Cell and Gene Therapy, Baylor College of Medicine, Houston, TX USA; 161 National Cancer Institute, NIH, Bethesda, MD USA; 162 Frederick National Laboratory for Cancer Research Leidos Biomedical Research, Inc., Frederick, MD USA; 163 University of California, Santa Cruz, Santa Cruz, CA USA; 164 UC Santa Cruz Genomics Institute, University of California, Santa Cruz, Santa Cruz, CA USA; 165 Children’s Hospital of Philadelphia Division of Oncology, Philadelphia, PA USA; 166 University of Pennsylvania, Philadelphia, PA USA; 167 Department of Pathology and Laboratory Medicine, Division of Neuropathology, University of Pennsylvania, Philadelphia, PA USA; 168 University of Pennsylvania, Philadelphia, PA USA; 169 Fox Chase Cancer Center, Philadelphia, PA USA; 170 Department of Pathology and Laboratory Medicine, Perelman School of Medicine, University of Pennsylvania, Philadelphia, PA USA; 171 TILT Biotherapeutics, Helsinki, Uusimaa Finland; 172 University of Helsinki, Helsinki, Uusimaa Finland; 173 Finnish Food Safety Authority, Helsinki, Uusimaa Finland; 174 Lion Biotechnologies, Inc., Tampa, FL USA; 175 The Comprehensive Cancer Center of Drum-Tower Hospital, Medical School of Nanjing University & Clinical Cancer Institute of Nanjing University, Nanjing, Jiangsu People’s Republic of China; 176 Experimental Transplantation and Immunology Branch, National Cancer Institute, National Institutes of Health, Bethesda, MD USA; 177 Surgery Branch, National Cancer Institute, National Institutes of Health, Bethesda, MD USA; 178 Fred Hutchinson Cancer Research Center, University of Washington, Seattle, WA USA; 179 University of Pittsburgh Cancer Institute, Pittsburgh, PA USA; 180 NantBioScience, Inc., Culver City, CA USA; 181 NantKwest, Inc., Culver City, CA USA; 182 UPMC Cancer Center, University of Pittsburgh Cancer Institute, Pittsburgh, PA USA; 183 Kite Pharma, Inc, Santa Monica, CA USA; 184 Surgery Branch, National Cancer Institute, Bethesda, MD USA; 185 Experimental Transplantation and Immunology Branch, National Cancer Institute, Bethesda, MD USA; 186 Lion Biotechnologies, Inc., Tampa, FL USA; 187 Emory University, Atlanta, GA USA; 188 University of Minnesota, Minneapolis, MN USA; 189 University of California, Davis, Sacramento, CA USA; 190 California State University, Sacramento, Sacramento, CA USA; 191 Georg-Speyer-Haus, Institute for Tumor Biology and Experimental Therapy, Frankfurt, Germany; 192 Institute for Neurooncology, Goethe University, Frankfurt, Germany; 193 Edinger Institute, Goethe University, Frankfurt, Frankfurt, Germany; 194 German Red Cross Blood Donation Service North-East, Dresden, Dresden, Germany; 195 University of Chicago, Chicago, IL USA; 196 University of Chicago Medical Center, Chicago, IL USA; 197 Baylor College of Medicine, Houston, TX USA; 198 Center for Cell and Gene Therapy, Baylor College of Medicine, Houston, TX USA; 199 Blood Center of Wisconsin, Milwaukee, WI USA; 200 Department of Immunology, Roswell Park Cancer Institute, Buffalo, NY USA; 201 Koch Institute for Integrative Cancer Research and Department of Biology, Massachusetts Institute of Technology, Cambridge, MA USA; 202 University of Pittsburgh Cancer Institute, Pittsburgh, PA USA; 203 University of Pittsburgh, Pittsburgh, PA USA; 204 Center for Cell Engineering, Memorial Sloan Kettering Cancer Center, New York, NY USA; 205 Department of Urology, Weill Cornell Medical College, New York, NY USA; 206 ENT Departement, Lund University Hospital, Lund, Skane Lan Sweden; 207 Department of Immunotechnology, Lund University, Lund, Skane Lan Sweden; 208 ENT Department, Skåne University Hospital, Lund, Lund, Skane Lan Sweden; 209 AstraZeneca, Cambridge, England, UK; 210 Samsung Medical Center, Seoul, South Korea; 211 Samsung Medical Center, Sungkyunkwan University School of Medicine, Seoul, South Korea; 212 AstraZeneca, Macclesfield, England, UK; 213 University Hospital of South Manchester, Manchester, England, UK; 214 AstraZeneca, Waltham, MA USA; 215 Cellular Technology Ltd, Shaker Hts, OH USA; 216 Cellular Technology Ltd, Shaker Heights, OH USA; 217 Cellular Technology Ltd, Shaker Heights, OH USA; 218 Merck & Co., Inc., West Point, PA USA; 219 Merck & Co., Inc., Kenilworth, NJ USA; 220 Seoul National University College of Medicine, Seoul, Republic of Korea; 221 Indiana University Health University Hospital, Indianapolis, IN USA; 222 Icahn School of Medicine at Mount Sinai, New York, NY USA; 223 Check Point Sciences, Cambridge, MA USA; 224 Tish Cancer Institute, Icahn School of Medicine at Mount Sinai, New York, NY USA; 225 Mayo Clinic, Rochester, MN USA; 226 Yale University School of Medicine, New Haven, CT USA; 227 Cleveland Clinic Main Campus, Cleveland, OH USA; 228 Stanford University School of Medicine, Stanford, CA USA; 229 Bristol-Myers Squibb, Princeton, NJ USA; 230 Department of Surgery, Johns Hopkins University School of Medicine, Sidney Kimmel Comprehensive Cancer Center, and Bloomberg-Kimmel Institute for Cancer Immunotherapy, Baltimore, MD USA; 231 Department of Oncology, Johns Hopkins University School of Medicine, Sidney Kimmel Comprehensive Cancer Center, and Bloomberg-Kimmel Institute for Cancer Immunotherapy, Baltimore, MD USA; 232 NanoString Technologies, Seattle, WA USA; 233 University of Washington, Seattle, WA USA; 234 University of Minnesota, Minneapolis, MN USA; 235 Roswell Park Cancer Institute, Buffalo, NY USA; 236 University of Maryland, Baltimore, Baltimore, MD USA; 237 Lymphoid Malignancies Branch, Center for Cancer Research, National Cancer Institute, Bethesda, MD USA; 238 Earle A. Chiles Research Institute, Providence Cancer Center, Portland, OR USA; 239 AgonOx, Inc., Portland, OR USA; 240 Juno Therapeutics, Portland, OR USA; 241 Johns Hopkins University, Baltimore, MD USA; 242 Heat Biologics, Durham, NC USA; 243 Adaptive Biotechnologies, Seattle, WA USA; 244 Merck & Co., Inc., Kenilworth, NJ USA; 245 University of Washington, Seattle, Seattle, WA USA; 246 University of Texas MD Anderson Cancer Center, Houston, TX USA; 247 University of Chicago, Chicago, Chicago, IL USA; 248 University of California, Los Angeles, Los Angeles, CA USA; 249 Merck & Co., Inc., Kenilworth, NJ USA; 250 MSD China, Beijing, People’s Republic of China; 251 Fox Chase Cancer Center, Philadelphia, PA USA; 252 Dana-Farber Cancer Institute, Boston, MA USA; 253 Thermo Fisher Scientific, Newburyport, MA USA; 254 MBL International, Woburn, MA USA; 255 MBL International, Des Plaines, IL USA; 256 Jounce Therapeutics, Cambridge, MA USA; 257 Janssen R&D, Spring House, PA USA; 258 Providence Cancer Center, Portland, OR USA; 259 Earle A. Chiles Research Institute at Portland Providence Cancer Center, Portland, OR USA; 260 Oregon Health & Science University, Portland, OR USA; 261 Sudha Hospital and Merdical Research Center, Kota, Rajasthan India; 262 Department of Oncology, Barbara Ann Karmanos Cancer Institute, Wayne State University, Detroit, MI USA; 263 Karmanos Cancer Institute, Detroit, MI USA; 264 Fred Hutchinson Cancer Research Center, University of Washington, Seattle, WA USA; 265 Trillium Therapeutics, Mississauga, ON Canada; 266 Department of Oncology, National Taiwan University Hospital, Taipei, Taipei, Taiwan, People’s Republic of China; 267 Institute of Population Health Sciences, National Health Research Institutes, Taiwan, Zhunan, Miaoli Taiwan, People’s Republic of China; 268 Department of Otorhinolaryngology, National Taiwan University Hospital, Taipei, Taipei, Taiwan, People’s Republic of China; 269 Taiwan Cooperative Oncology Group, National Health Research Institutes, Taiwan, Zhunan, Miaoli Taiwan, People’s Republic of China; 270 Flagship Biosciences, Inc, Westminster, CO USA; 271 Advanced Cell Diagnostics, Newark, CA USA; 272 H. Lee Moffitt Cancer Center, Tampa, FL USA; 273 Curiox Biosystems, San Carlos, CA USA; 274 Curiox Biosystems, San Carlos, CA USA; 275 Curiox Biosystems, San Carlos, CA USA; 276 Johns Hopkins University, Baltimore, MD USA; 277 Biocenter, Division of Bioinformatics Medical University of Innsbruck, Innsbruck, Tirol Australia; 278 Sidney Kimmel Comprehensive Cancer Center, Johns Hopkins University, Baltimore, MD USA; 279 CureVac AG, Tubingen, Baden-Wurttemberg, Germany; 280 CureVac AG, Frankfurt am Main, Hessen Germany; 281 Eberhard-Karls-Universität Tübingen, WSI/ZBIT, Applied Bioinformatics Group, Tubingen, Baden-Wurttemberg Germany; 282 Division of Immunology, New England Primate Research Center, Harvard Medical School, Southborough, MA USA; 283 Earle A. Chiles Research Institute, Providence Cancer Center, Portland, OR USA; 284 Robert W. Franz Cancer Research Center, Earle A. Chiles Research Institute, Providence Cancer Center, Portland, Oregon, USA, Portland, OR USA; 285 Providence Cancer Center, Portland, OR USA; 286 Laboratory of Translational Oncology, University of Crete, School of Medicine, Heraklion, Greece; 287 Flagship Biosciences, Inc, Westminster, CO USA; 288 Department of Fundamental Oncology, Ludwig Cancer Research Center, University of Lausanne, Epalinges, Vaud Switzerland; 289 Department of Fundamental Oncology, Centre Hospitalier Universitaire Vaudois (CHUV), Epalinges, Vaud Switzerland; 290 School of Life Sciences Weihenstephan, Technical University Munich, Freising, Bayern Germany; 291 Department of Oncology, Centre Hospitalier Universitaire Vaudois (CHUV), Epalinges, Vaud Switzerland; 292 Rutgers Cancer Institute of New Jersey, Rutgers University, Piscataway, NJ USA; 293 Rutgers Cancer Institute of New Jersey, New Brunswick, NJ USA; 294 University Radiology, New Brunswick, NJ USA; 295 Foundation Medicine, Inc., Cambridge, MA USA; 296 Université de Montréal, Centre de recherche du CHUM (CRCHUM), Montreal, PQ Canada; 297 University of Tunis El Manar II, Tunis, Tunisia; 298 Institut du Cancer de Montréal (ICM), Centre de recherche du CHUM (CRCHUM), Montreal, PQ Canada; 299 Centre Hospitalier de l’Université de Montréal (CHUM), Université de Montréal, Montreal, PQ Canada; 300 Ontario Cancer Institute, University of Toronto, Toronto, ON Canada; 301 Thermo Fisher Scientific, South San Francisco, CA USA; 302 Roswell Park Cancer Institute, Buffalo, NY USA; 303 OmniSeq, LLC, Buffalo, NY USA; 304 Thermo Fisher Scientific, South San Francisco, CA USA; 305 Roswell Park Cancer Institute, Buffalo, NY USA; 306 OmniSeq, LLC, Buffalo, NY USA; 307 Thermo Fisher Scientific, South San Francisco, CA USA; 308 Department of Surgery, University of Virginia, Charlottesville, VA USA; 309 Department of Pathology, Johns Hopkins Medicine, Baltimore, MD USA; 310 Department of Public Health Sciences, University of Virginia, Charlottesville, VA USA; 311 Department of Pathology, University of Virginia, Charlottesville, VA USA; 312 Department of Immunobiology, Yale University, New Haven, CT USA; 313 University of Virginia, Charlottesville, VA USA; 314 Department of Surgery, University of Virginia, Charlottesville, VA USA; 315 Department of Pathology, Johns Hopkins Medicine, Baltimore, MD USA; 316 University of Virginia, Charlottesville, VA USA; 317 Department of Pathology, University of Virginia, Charlottesville, VA USA; 318 Bristol-Myers Squibb, Princeton, NJ USA; 319 Earle A. Chiles Research Institute, Providence Portland Medical Center, Portland, OR USA; 320 Providence Portland Medical Center, Portland, OR USA; 321 Robert W. Franz Cancer Research Center, Earle A. Chiles Research Institute, Providence Cancer Center, Portland, Oregon USA; 322 Earle A. Chiles Research Institute, Providence Cancer Center, Portland, OR USA; 323 University of Texas MD Anderson Cancer Center, Houston, TX USA; 324 Ventana Medical Systems, Inc., Tucson, AZ USA; 325 Medical Oncology Department, Massachusetts General Hospital, Boston, MA USA; 326 Simon Cancer Center, Indiana University, Indianapolis, IN USA; 327 H. Lee Moffitt Cancer Center, Tampa, FL USA; 328 Department of Medical Oncology, City Of Hope, Duarte, CA USA; 329 Case Western Reserve University, Cleveland, OH USA; 330 The Ohio State University, Columbus, OH USA; 331 Texas Oncology-Baylor Charles A. Sammons Cancer Center, Dallas, TX USA; 332 Earle A. Chiles Research Institute, Providence Cancer Center, Portland, OR USA; 333 Biodesix, Inc., Boulder, CO USA; 334 Massachusetts General Hospital Cancer Center, Boston, MA USA; 335 Wake Forest Baptist Medical Center, Winston Salem, NC USA; 336 Levine Cancer Institute, Carolinas HealthCare System, Charlotte, NC USA; 337 Roswell Park Cancer Institute, Buffalo, NY USA; 338 Loyola University Medical Center, Maywood, IL USA; 339 Georgetown-Lombardi Comprehensive Cancer Center, Washington DC, DC USA; 340 Rutgers Cancer Institute of New Jersey, New Brunswick, NJ USA; 341 Robert Lurie Comprehensive Cancer Center of Northwestern University, Chicago, IL USA; 342 NYU Langone Medical Center, New York, NY USA; 343 Beth Israel Deaconess Medical Center, Boston, MA USA; 344 NYU Langone Medical Center, New York, NY USA; 345 Yale Medical Oncology, New Haven, CT USA; 346 Yale University School of Medicine, New Haven, CT USA; 347 Biodesix, Inc., Boulder, CO USA; 348 Massachusetts General Hospital Cancer Center, Boston, MA USA; 349 Medical Oncology Department, Massachusetts General Hospital, Boston, MA USA; 350 PerkinElmer, Hopkinton, MA USA; 351 Definiens AG, Munich, Bayern Germany; 352 Istituto Nazionale dei Tumori di Napoli Fondazione “G. Pascale”, Naples, Campania Italy; 353 Istituto Nazionale Tumori Fondazione, Napoli, Italy; 354 Definiens AG, Munich, Bayern Germany; 355 Ludwig-Maximilians-Universität München, Pathologisches Institut, Munich, Bayern Germany; 356 Ludwig-Maximilians-Universität München, Urologische Klinik und Poliklinik, Munich, Bayern Germany; 357 NantOmics, Rockville, MD USA; 358 NantOmics, Culver City, CA USA; 359 General Hospital First Medical Faculty, Prague, Hlavni mesto Praha Czech Republic; 360 Institut für Pathologie, Universität Bern, Bern, Switzerland; 361 Pathologie, Friedrich-Alexander-Universität Erlangen-Nürnberg, Erlangen, Germany; 362 Earle A. Chiles Research Institute at Portland Providence Cancer Center, Portland, OR USA; 363 Ludwig Maximilian University of Munich and Thoracic Oncology Centre Munich, Munich, Germany; 364 Comprehensive Pneumology Center (CPC) and Member of the German Center for Lung Research (DZL, CPC-M), Munich, Bayern Germany; 365 Department of Clinical Radiology, University of Munich, Munich, Bayern Germany; 366 Comprehensive Pneumology Center (CPC) and Member of the German Center for Lung Research (DZL, CPC-M); Department of General, Visceral, Transplantation, Vascular and Thoracic Surgery, Hospital of the Ludwig Maximilian University, Munich, Bayern Germany; 367 Comprehensive Pneumology Center (CPC) and Member of the German Center for Lung Research (DZL, CPC-M), Munich, Germany; 368 Hospital of the Ludwig Maximilian University, Munich, Germany; 369 Asklepios Clinic, Munich, Bayern Germany; 370 Comprehensive Pneumology Center (CPC) and Member of the German Center for Lung Research (DZL, CPC-M), Munich, Germany; 371 Asklepios Clinic Munich-Gauting, Gauting, Bayern Germany; 372 Robert W. Franz Cancer Research Center, Earle A. Chiles Research Institute, Providence Cancer Center, Portland, Oregon USA; 373 Comprehensive Pneumology Center (CPC) and Member of the German Center for Lung Research (DZL, CPC-M), Institute of Pathology, University of Munich, Munich, Bayern Germany; 374 Institute of Immunology, The Key Laboratory of Innate Immunity and Chronic Disease (Chinese Academy of Medical Science), School of Life Sciences and Medical Center, University of Science & Technology of China, Hefei, Anhui People’s Republic of China; 375 Massachusetts General Hospital Cancer Center, Harvard Medical School, Charlestown, MA USA; 376 Massachusetts General Hospital, Harvard Medical School, Boston, MA USA; 377 University of California, San Francisco, CA USA; 378 University Hospital of Zurich, Zurich, Switzerland; 379 The Angeles Clinic & Research Institute, Los Angeles, CA USA; 380 University of California, Los Angeles, CA USA; 381 Ventana Medical Systems, Inc., Tucson, AZ USA; 382 Spring Bioscience, Pleasanton, CA USA; 383 Oregon Health & Science University, Portland, OR USA; 384 Intel Corporation, Hillsboro, OR USA; 385 Karolinska Institutet, Stockholms Lan, Sweden; 386 NanoString Technologies, Seattle, WA USA; 387 NanoString Technologies, Seattle, WA USA; 388 Uppsala University, Uppsala, Uppsala Lan Sweden; 389 Baylor College of Medicine, Houston, TX USA; 390 Xencor, Inc., Monrovia, CA USA; 391 Xencor Inc., Monrovia, CA USA; 392 F-star Biotechnology Ltd., Cambridge, England, UK; 393 Biocon Research Ltd, Bangalore, India; 394 Merus N.V., Utrecht, Netherlands; 395 Department of Laboratory Medicine, Radboud University Medical Center, Nijmegen, Gelderland Netherlands; 396 University of Michigan, Ann Arbor, MI USA; 397 Technical University of Munich, Munchen, Germany; 398 Gustave Roussy, Université Paris-Saclay, Villejuif, France; 399 Dana-Farber Cancer Institute, Harvard Medical School, Boston, MA USA; 400 Merck & Co., Inc., Kenilworth, NJ USA; 401 Barts Cancer Institute, Queen Mary University of London, London, England, UK; 402 Johns Hopkins University, Sidney Kimmel Cancer Center, Baltimore, MD USA; 403 Johns Hopkins University Cancer Center, Baltimore, MD USA; 404 Merck & Co., Inc., Kenilworth, NJ USA; 405 Royal Marsden Hospital, London, England, UK; 406 Rutgers Cancer Institute of New Jersey, New Brunswick, NJ USA; 407 The Ohio State University, Columbus, OH USA; 408 Monash Health and Peter MacCallum Cancer Centre, Clayton, Victoria Australia; 409 Monash Health, Clayton, Victoria Australia; 410 Massachusetts General Hospital, Boston, MA USA; 411 Merck & Co., Inc., Kenilworth, NJ USA; 412 Mayo Clinic, Rochester, MN USA; 413 Cleveland Clinic Taussig Cancer Institute, Cleveland, OH USA; 414 Dana-Farber Cancer Institute, Brigham and Women’s Hospital, Boston, MA USA; 415 Pfizer Inc., Milano, Lombardia Italy; 416 Department of Cancer Medicine, Gustave Roussy Cancer Campus, University of Paris Sud, Villejuif, Ile-de-France France; 417 Netherlands Cancer Institute, Amsterdam, Noord-Holland Netherlands; 418 Georgetown-Lombardi Comprehensive Cancer Center, Washington DC, USA; 419 Royal Marsden Hospital, London, England, UK; 420 Medical University of Vienna, Vienna, Wien Austria; 421 Memorial Sloan Kettering Cancer Center, New York, NY USA; 422 Department of Oncology, Center for Cancer Immune Therapy, Herlev University Hospital, Herlev, Hovedstaden Denmark; 423 Johns Hopkins Hospital, Baltimore, MD USA; 424 MacroGenics, Inc., Rockville, MD USA; 425 MacroGenics, Inc., South San Francisco, CA USA; 426 Johns Hopkins University Cancer Center, Baltimore, MD USA; 427 Johns Hopkins University, Sidney Kimmel Cancer Center, Baltimore, MD USA; 428 University of California, Davis, Sacramento, CA USA; 429 University of California, Davis, Davis, CA USA; 430 National Cancer Institute, Bethesda, MD USA; 431 Surgery Branch, National Cancer Institute, National Institutes of Health, Bethesda, MD USA; 432 Earle A. Chiles Research Institute, Providence Cancer Center, Portland, OR USA; 433 Oncology Specialists, S.C., Niles, IL USA; 434 John Wayne Cancer Institute, Santa Monica, CA USA; 435 University of Utah, Huntsman Cancer Institute, Salt Lake City, UT USA; 436 Viralytics, Inc., Sydney, New South Wales Australia; 437 Viralytics Limited, Newcastle, New South Wales Australia; 438 Sidney Kimmel Comprehensive Cancer Center at Johns Hopkins University, Baltimore, MD USA; 439 National Cancer Center Hospital East, Kashiwa-shi, Chiba Japan; 440 Saint Antoine Hospital, Paris, Ile-de-France France; 441 Sarah Cannon Research Institute and Department of Medical Oncology, Tennessee Oncology, Nashville, TN USA; 442 Merck & Co., Inc., Kenilworth, NJ USA; 443 National Center for Tumor Diseases, Heidelberg, Baden-Wurttemberg Germany; 444 Sarah Cannon Research Institute, Tennessee Oncology, PLLC, Nashville, TN USA; 445 Sarah Cannon Research Institute, Florida Cancer Specialists, Sarasota, FL USA; 446 Laura and Isaac Perlmutter Cancer Center at NYU Langone Medical Center, New York, NY USA; 447 Columbia University Medical Center, New York, NY USA; 448 Winship Cancer Institute at Emory University, Atlanta, GA USA; 449 Eli Lilly and Company, Bridgewater, NJ USA; 450 AstraZeneca Pharmaceuticals, Waltham, MA USA; 451 Sarah Cannon Research Institute at HealthONE, Denver, CO USA; 452 David Geffen School of Medicine at UCLA, Santa Monica, CA USA; 453 Montefiore Medical Center, White Plains, NY USA; 454 The Crown Princess Mary Cancer Centre Westmead, Westmead, New South Wales Australia; 455 Princess Margaret Cancer Centre, Toronto, ON Canada; 456 Inje University Haeundae Paik Hospital, Busan, Republic of Korea; 457 University of Massachusetts Medical School, Worchester, MA USA; 458 Dartmouth Hitchcock Medical Center, Lebanon, NH USA; 459 Merck & Co., Inc., Kenilworth, NJ USA; 460 Gabinete Radiologico del Dr. Pita, NA, Madrid, Spain; 461 Mayo Clinic, Rochester, MN USA; 462 University of Cincinnati, Cincinnati, OH USA; 463 James Comprehensive Cancer Center, The Ohio State University, Columbus, OH USA; 464 James Cancer Hospital, The Ohio State University, Columbus, OH USA; 465 Instituto do Câncer do Estado de São Paulo - Faculdade de Medicina da Universidade de São Paulo, São Paulo, Brazil; 466 Advaxis Immunotherapies, Princeton, NJ USA; 467 Department of Obstetrics & Gynecology, University of Alabama at Birmingham, Birmingham, AL USA; 468 Samsung Medical Center, Sungkyunkwan University School of Medicine, Seoul, Republic of Korea; 469 Stephenson Oklahoma Cancer Center, Oklahoma City, OK USA; 470 Vall d’Hebron University Hospital, Vall d’Hebron Institute of Oncology (VHIO), Barcelona, Spain; 471 Loyola University, Maywood, IL USA; 472 University of California, Irvine Medical Center, Orange, CA USA; 473 University of Arizona Cancer Center-Phoenix Creighton University School of Medicine at Dignity Health St. Joseph’s Hospital and Medical Center, Phoenix, AZ USA; 474 University of Texas MD Anderson Cancer Center, Houston, TX USA; 475 Dana-Farber Cancer Institute, Harvard Medical School, Boston, MA USA; 476 Dana-Farber Cancer Institute/Brigham and Women’s Hospital, Boston, MA USA; 477 Merck & Co., Inc., Kenilworth, NJ USA; 478 University of Warwick, Coventry, England, UK; 479 University of Virginia School of Medicine, Charlottesville, VA USA; 480 Sidney Kimmel Comprehensive Cancer Center at Johns Hopkins University, Baltimore, MD USA; 481 University of Alabama at Birmingham Comprehensive Cancer Center, Birmingham, AL USA; 482 Erasmus MC Cancer Institute, Rotterdam, Netherlands; 483 University Radboud, Nijmegen, Netherlands; 484 Memorial Sloan Kettering Cancer Center, New York, NY USA; 485 Committee on Immunology and Department of Medicine, University of Chicago, Chicago, IL USA; 486 Dana-Farber Cancer Institute, Boston, MA USA; 487 Princess Margaret Cancer Centre and University of Toronto, Toronto, ON Canada; 488 Memorial Sloan Kettering Cancer Center, New York, NY USA; 489 Medical College of Wisconsin, Milwaukee, WI USA; 490 Institut Gustave Roussy, Villejuif, Ile-de-France France; 491 Institute of Hematology “L. e A. Seràgnoli,” Università di Bologna, Bologna, Emilia-Romagna Italy; 492 Merck & Co., Inc., Kenilworth, NJ USA; 493 Washington University School of Medicine in St. Louis, St Louis, MO USA; 494 Tish Cancer Institute, Icahn School of Medicine at Mount Sinai, New York, NY USA; 495 Hematology and Oncology Division, Columbia University Medical Center, New York, NY USA; 496 Icahn School of Medicine at Mount Sinai, New York, NY USA; 497 Oncovir, Inc., Washington, DC USA; 498 Carolina BioOncology Institute, PLLC, Huntersville, NC USA; 499 Icahn School of Medicine at Mount Sinai, New York, NY USA; 500 Mary Crowley Cancer Research Centers, Texas Oncology, P.A., Gradalis, Inc., Medical City Dallas Hospital, Baylor University Medical Center, Dallas, TX USA; 501 Johns Hopkins University School of Medicine, Baltimore, MD USA; 502 University of Chicago School of Medicine, Chicago, IL USA; 503 South Texas Accelerated Research Therapeutics, LLC, San Antonio, TX USA; 504 Corvus Pharmaceuticals, Burlingame, CA USA; 505 Seattle Cancer Care Alliance, University of Washington, Seattle, WA USA; 506 University of Michigan, Ann Arbor, MI USA; 507 University of North Carolina Lineberger Comprehensive Cancer Center, University of North Carolina Chapel Hill, Chapel Hill, NC USA; 508 University of Chicago Medical Center, Chicago, IL USA; 509 Montefiore Medical Center, Bronx, NY USA; 510 Seattle Genetics, Inc., Bothell, WA USA; 511 Earle A. Chiles Research Institute, Providence Cancer Center, Portland, OR USA; 512 Sidney Kimmel Comprehensive Cancer Center at Johns Hopkins University, Baltimore, MD USA; 513 School of Medicine Oncology Biostatistics, Baltimore, MD USA; 514 Keck School of Medicine of USC, Los Angeles, CA USA; 515 Beth Israel Deaconess Medical Center, Boston, MA USA; 516 Merck & Co., Inc., Kenilworth, NJ USA; 517 Institut Gustave Roussy, Villejuif, Ile-de-France France; 518 Dana-Farber Cancer Institute, Boston, MA USA; 519 Mayo Clinic, Rochester, MN USA; 520 The Ohio State University, Columbus, OH USA; 521 Merck & Co., Inc., Kenilworth, NJ USA; 522 Institute of Hematology “L. e A. Seràgnoli,”, Università di Bologna, Bologna, Emilia-Romagna Italy; 523 University of Pittsburgh Cancer Institute, Pittsburgh, PA USA; 524 University of Pittsburgh Cancer Institute, UPMC Cancer Center, Pittsburgh, PA USA; 525 Department of Investigational Cancer Therapeutics, The University of Texas MD Anderson Cancer Center, Houston, TX USA; 526 Sarah Cannon Research Institute, Nashville, TN USA; 527 Calithera Biosciences, South San Francisco, CA USA; 528 South Texas Accelerated Research Therapeutics, San Antonio, TX USA; 529 Stanford University School of Medicine, Stanford, CA USA; 530 University of Colorado Anschutz Medical Campus, Aurora, CO USA; 531 Institute for Stem Cell Biology and Regenerative Medicine and Forty Seven, Inc., Palo Alto, CA USA; 532 Institute for Stem Cell Biology and Regenerative Medicine, Forty Seven, Inc., Stanford University School of Medicine, Palo Alto, CA USA; 533 Forty Seven, Inc., Menlo Park, CA USA; 534 Institute for Stem Cell Biology and Regenerative Medicine, Stanford University School of Medicine, Stanford, CA USA; 535 Earle A. Chiles Research Institute, Providence Portland Medical Center, Portland, OR USA; 536 Providence St. Vincent Medical Center, Portland, OR USA; 537 Providence Portland Medical Center, Portland, OR USA; 538 Providence Medical Group, Clackamas, OR USA; 539 Providence Medical Group, Portland, OR USA; 540 Providence Cancer Center, Portland, OR USA; 541 Oregon Health & Science University, Portland, OR USA; 542 Providence Medical Group, Newberg, OR USA; 543 Robert W. Franz Cancer Research Center, Earle A. Chiles Research Institute, Providence Cancer Center, Portland, Oregon USA; 544 Earle A. Chiles Research Institute, Providence Cancer Center, Portland, OR USA; 545 Memorial Sloan Kettering Cancer Center, New York, NY USA; 546 Department of Oncology, Center for Cancer Immune Therapy, Herlev University Hospital, Herlev, Hovedstaden Denmark; 547 Department of Oncology, Herlev Hospital, Herlev, Hovedstaden Denmark; 548 Royal Melbourne Hospital and Peter MacCallum Cancer Centre, Melbourne, Victoria Australia; 549 Monash Cancer Center, East Bentleigh, Victoria Australia; 550 Peter MacCallum Cancer Center, Melbourne, Victoria Australia; 551 Austin Hospital, Melbourne, Victoria Australia; 552 Prince of Wales Hospital, Sydney, New South Wales Australia; 553 Royal Melbourne Hospital, Melbourne, Victoria Australia; 554 Linear Clinical Research, Sir Charles Gairdner Hospital, Nedlands, Western Australia Australia; 555 Austin Health and Olivia Newton-John Cancer Research Institute, Melbourne, Victoria Australia; 556 The Prince of Wales Hospital, Randwick, New South Wales Australia; 557 BeiGene (Beijing) Co., Ltd, Beijing, People’s Republic of China; 558 BeiGene (US) Co. Ltd., Fort Lee, NJ USA; 559 Department of Surgical Oncology, Division of Surgery, The University of Texas MD Anderson Cancer Center, Houston, TX USA; 560 Department of Surgery, University of Virginia, Charlottesville, VA USA; 561 Department of Gastrointestinal (GI) Medical Oncology, Division of Cancer Medicine, The University of Texas MD Anderson Cancer Center, Houston, TX USA; 562 Sylvester Comprehensive Cancer Center, University of Miami, Miami, FL USA; 563 University of Virginia, Charlottesville, VA USA; 564 Division of Surgical Oncology, University of Virginia, Charlottesville, VA USA; 565 Dana-Farber Cancer Institute, Harvard University, Boston, MA USA; 566 Cleveland Clinic Taussig Cancer Institute, Cleveland, OH USA; 567 Barts Cancer Institute, Queen Mary University of London, London, England UK; 568 Merck & Co., Inc., Kenilworth, NJ USA; 569 Georgetown-Lombardi Comprehensive Cancer Center, Washington DC, USA; 570 Jounce Therapeutics, Cambridge, MA USA; 571 University of Chicago School of Medicine, Chicago, IL USA; 572 University of Chicago, Chicago, IL USA; 573 University of Texas MD Anderson Cancer Center, Houston, TX USA; 574 Complejo Asistencial Universitario de Salamanca/IBSAL, Salamanca, Castilla y Leon Spain; 575 National Hospital Organization, Shibukawa Medical Center, Gunma, Japan; 576 Middlemore Hospital, Otahuhu, Auckland New Zealand; 577 Hospital Germans Triasi Pugoe, Barcelona, Spain; 578 University Hospital Heidelberg, Heidelberg, Germany; 579 Nagoya City University Graduate School of Medical Sciences, Nagoya, Japan; 580 Hadassah Medical Center, Jerusalem, Italy; 581 Clinica Universidad de Navarra, Pamplona, Navarra Spain; 582 The Mount Sinai Medical Hospital, New York, NY USA; 583 Winship Cancer Institute, Emory University, Atlanta, GA USA; 584 Merck & Co., Inc., Kenilworth, NJ USA; 585 University of Texas MD Anderson Cancer Center, Houston, TX USA; 586 Winship Cancer Institute, Emory University, Atlanta, GA USA; 587 Jane Thompson Russell Cancer Care Center, Tacoma, WA USA; 588 Texas Oncology, Tyler, TX USA; 589 Complejo Asistencial Universitario de Salamanca/IBSAL, Salamanca, Castilla y Leon Spain; 590 Rocky Mountain Cancer Centers, Denver, CO USA; 591 Oslo University Hospital, Oslo, Norway; 592 Clinica Universidad de Navarra, Pamplona, Navarra Spain; 593 Merck & Co., Inc., Kenilworth, NJ USA; 594 The Mount Sinai Medical Hospital, New York, NY USA; 595 Princess Margaret Cancer Centre, Toronto, ON Canada; 596 Li Ka Shing Faculty of Medicine, The University of Hong Kong, Pok Fu Lam, People’s Republic of China; 597 Queen Elizabeth Hospital, Hong Kong, People’s Republic of China; 598 The Chinese University of Hong Kong, Hong Kong, People’s Republic of China; 599 Samsung Medical Center, Sungkyunkwan University, Seoul, Republic of Korea; 600 National Cancer Centre Singapore, Singapore, Singapore; 601 National Taiwan University Hospital, Taipei City, Taiwan, People’s Republic of China; 602 Taichung Veterans General Hospital, Taichung City, Taiwan, People’s Republic of China; 603 Merck & Co., Inc., Kenilworth, NJ USA; 604 University of Colorado Denver, Aurora, CO USA; 605 University of Texas MD Anderson Cancer Center, Houston, TX USA; 606 Karmanos Cancer Institute, Detroit, MI USA; 607 Calithera Biosciences, South San Francisco, CA USA; 608 Memorial Sloan Kettering Cancer Center, New York, NY USA; 609 Seattle Cancer Care Alliance, Seattle, WA USA; 610 Merck & Co., Inc., Kenilworth, NJ USA; 611 University of Wisconsin, Madison, WI USA; 612 University of Wisconsin School of Medicine and Public Health, Madison, WI USA; 613 University of Wisconsin Carbone Cancer Center, Madison, WI USA; 614 Westchester Medical Center, New York Medical College, Valhalla, NY USA; 615 Department of Surgery, Medical University of South Carolina, Charleston, SC USA; 616 Provenance Biopharmaceuticals Corp., Carlisle, MA USA; 617 University of Texas MD Anderson Cancer Center, Houston, TX USA; 618 Cognate Bioservices, Inc, Hanover, MD USA; 619 Northwest Biotherapeutics, Bellevue, WA USA; 620 David Geffen School of Medicine at UCLA, Los Angeles, CA USA; 621 National Institutes of Health Clinical Center, Bethesda, MD USA; 622 Sidra Medical and Research Center, Doha, Qatar; 623 City of Hope and Beckman Research Institute, Duarte, CA USA; 624 VA Greater Los Angeles Healthcare System, Los Angeles, CA USA; 625 Tish Cancer Institute, Icahn School of Medicine at Mount Sinai, New York, NY USA; 626 Icahn School of Medicine at Mt Sinai, New York, NY USA; 627 NYU Perlmutter Cancer Center, New York, NY USA; 628 Roswell Park Cancer Institute, Buffalo, NY USA; 629 Cleveland Clinic, Cleveland, OH USA; 630 Duke Cancer Institute, Durham, NC USA; 631 University of Wisconsin, Madison, WI USA; 632 University of Chicago School of Medicine, Chicago, IL USA; 633 Celldex Therapeutics, Hampton, NJ USA; 634 Cancer Therapy Evaluation Program, National Cancer Institute, National Institutes of Health, Rockville, MD USA; 635 NanoString Technologies, Seattle, WA USA; 636 University of Washington, Seattle, WA USA; 637 Cancer Immunotherapy Trials Network, Fred Hutchinson Cancer Research Center, Seattle, WA USA; 638 Laboratory of Tumor Immunology and Biology, National Cancer Institute, Bethesda, MD USA; 639 Yale Cancer Center, New Haven, CT USA; 640 National Cancer Institute, Bethesda, MD USA; 641 Laboratory of Tumor Immunology and Biology, National Cancer Institute, NIH, Bethesda, MD USA; 642 EMD Serono, Billerica, MA USA; 643 Merck KGaA, Darmstadt, Hessen Germany; 644 Laboratory of Tumor Immunology and Biology, Center for Cancer Research, National Cancer Institute, Bethesda, MD USA; 645 Genitourinary Malignancies Branch, Center for Cancer Research, National Cancer Institute, National Institutes of Health, Bethesda, MD USA; 646 University of Texas MD Anderson Cancer Center, Houston, TX USA; 647 McDougall Scientific Ltd., Toronto, ON Canada; 648 Telesta Therapeutics Inc., Saint-Laurent, PQ Canada; 649 Center for Applied Medical Research (CIMA), University of Navarra, Pamplona, Navarra Spain; 650 Bellvitge Biomedical Research Institute (IDIBELL), Barcelona, Catalonia Spain; 651 Bellvitge Biomedical Research Institute (IDIBELL), Barcelona, Navarra Spain; 652 Medical Research Institute La Fe, Valencia, Comunidad Valenciana Spain; 653 Five Prime Therapeutics, South San Francisco, CA USA; 654 Inhibrx, La Jolla, CA USA; 655 Centre for Biologics Evaluation and Research (CBER), U.S Food and Drug Administration, Silver Spring, MD USA; 656 Department of Immunology, University of Texas MD Anderson Cancer Center, Houston, TX USA; 657 University of Texas MD Anderson Cancer Center, Houston, TX USA; 658 Novartis Institutes for BioMedical Research, Inc, Cambridge, MA USA; 659 Novartis Institutes for BioMedical Research, Inc., Werk Klybeck, Basel-Landschaft, Switzerland; 660 Cellaria Biosciences, LLC, Cambridge, MA USA; 661 Bristol-Myers Squibb, Princeton, NJ USA; 662 Biothera Pharmaceuticals Inc, Eagan, MN USA; 663 Celldex Therapeutics, Hampton, NJ USA; 664 Celldex Therapeutics, Needham, MA USA; 665 Promega Corporation, Madison, WI USA; 666 Pieris Pharmaceuticals, Inc, Freising, Bayern Germany; 667 Oncotest GmbH, Freiburg, Baden-Wurttemberg Germany; 668 University of Chicago, Chicago, IL USA; 669 The Department of Pathology, The University of Chicago, Chicago, IL USA; 670 University of Chicago Medical Center, Chicago, IL USA; 671 City of Hope, Duarte, CA USA; 672 Johns Hopkins School of Medicine, Baltimore, MD USA; 673 Johns Hopkins, Irvine, CA USA; 674 Johns Hopkins Medical Institute, Baltimore, MD USA; 675 Beth Israel Deaconess Medical Center, Dana-Farber Cancer Institute, Boston, MA USA; 676 Dana-Farber Cancer Institute, Boston, MA USA; 677 Beth Israel Deaconess Medical Center, Boston, MA USA; 678 Massachusestts General Hospital, Boston, MA USA; 679 Neon Therapeutics, Inc, Cambridge, MA USA; 680 Earle A. Chiles Research Institute, Providence Cancer Center, Portland, OR USA; 681 Oregon Health & Science University, Portland, OR USA; 682 Karolinska Institutet, Stockholm, Stockholms Lan Sweden; 683 Molecular Partners, Schlieren-Zurich, Zurich Switzerland; 684 Rhode Island Hospital, Warren Alpert School of Medicine, Brown University, Providence, RI USA; 685 Warren Alpert School of Medicine, Brown University, Providence, RI USA; 686 Aurigene Discovery Technologies Limited, Bangalore, Karnataka India; 687 Montefiore Medical Center, Bronx, NY USA; 688 Alpine Immune Sciences, Seattle, WA USA; 689 Centre Leon Berard, Lyon, Rhone-Alpes France; 690 Centre Leon Berard CRCL UMR Inserm 1052/CNRS 5286, Lyon, Rhone-Alpes France; 691 UCBL1,Centre Leon Berard UMR Inserm 1052/CNRS 5286, Lyon, Rhone-Alpes France; 692 CRCL, UMR Inserm 1052/CNRS 5286, IHOP, Centre Léon Bérard, Gustave Roussy, Université Paris-Saclay, Lyon, Rhone-Alpes France

## Adoptive Cellular Therapy

### O1 IL-15 primes an mTOR-regulated gene-expression program to prolong anti-tumor capacity of human natural killer cells

#### Andreas Lundqvist^1^, Vincent van Hoef^1^, Xiaonan Zhang^1^, Erik Wennerberg^2^, Julie Lorent^1^, Kristina Witt^1^, Laia Masvidal Sanz^1^, Shuo Liang^1^, Shannon Murray^3^, Ola Larsson^1^, Rolf Kiessling^1^, Yumeng Mao^1^

##### ^1^Karolinska Institutet, Stockholm, Stockholms Lan, Sweden; ^2^Weill Cornell Medical College, New York, NY, USA; ^3^Nova Southeastern University, Cell Therapy Institute, Fort Lauderdale, FL, USA

###### **Correspondence:** Andreas Lundqvist (andreas.lundqvist@ki.se)

**Background**

NK cell-based immunotherapy is a potential therapeutic modality in patients with advanced cancers as transfer of haploidentical NK cells induces beneficial responses in patients with hematological malignancies; and leukemia clearance correlates with persistence and *in vivo* expansion of NK cells after infusion. Thus, sustained NK cell activity *in vivo* likely represents a therapy performance-limiting factor.

**Methods**

We performed genome-wide analysis of cytosolic and polysome-associated mRNA from interleukin (IL)-2 and IL-15 activated NK cells. Furthermore, the ability of IL-2 and IL-15 to sustain human NK cell activity following cytokine withdrawal as well as their effect on NK cells to resist tumor-induced immunosuppression was compared.

**Results**

After cytokine withdrawal, IL-15-treated NK cells maintained a higher level of cytotoxicity (p < 0.05) and showed lower levels of apoptosis (p < 0.05) compared with cells treated with IL-2. IL-15 augmented mTOR signaling, which correlated with increased expression of genes related to cell metabolism and respiration. Consistently, mTOR inhibition abrogated IL-15-induced cell function advantages. Moreover, mTOR-independent STAT-5 signaling contributed to improved NK cell function during cytokine activation but not following cytokine withdrawal. Upon co-culture with tumor cells or exposure to tumor cell supernatant, IL-15 activated NK cell maintained a significantly higher level of proliferation and cytotoxic activity (p < 0.05). Mechanistically, tumor-derived prostaglandin-E2 suppressed IL-2 cultured NK cells while IL-15 cultured NK cells remained activated. The superior performance of IL-15 stimulated NK cells was also observed using a clinically applicable protocol for NK cell expansion *in vitro* and *in vivo*.

**Conclusions**

This study adds to our understanding about establishment and maintenance of tumor-reactive NK cells and supports clinical implementation of IL-15 for adoptive NK cell therapy. More broadly, our studies suggest that a large aspect of cytokine-mediated gene expression programs and downstream cellular functions, including anti-tumor capacity, are overlooked if post-activation conditions are omitted. This is likely not limited to NK cells and should hence be considered in similar studies of other immune cells.

## Biomarkers and Immune Monitoring

### O2 ImmunoMap: a novel bioinformatics tool for analysis of T cell receptor repertoire data in model systems and clinical settings

#### John-William Sidhom^1^, Catherine A Bessell^2^, Jonathan Havel^3^, Jonathan Schneck^4^, Timothy A Chan^3^, Eliot Sachsenmeier^5^

##### ^1^Johns Hopkins University School of Medicine, Baltimore, MD, USA; ^2^Immunology Program, Johns Hopkins University, School of Medicine, Columbia, MD, USA; ^3^Memorial Sloan Kettering Cancer Center, New York, NY, USA; ^4^Johns Hopkins Medical Institute, Baltimore, MD, USA; ^5^University of Rochester, Monrovia, MD, USA

###### **Correspondence:** John-William Sidhom (jsidhom1@jhmi.edu)

**Background**

There has been a dramatic increase in T cell receptor (TCR) sequencing spurred, in part, by the widespread adoption of this technology across academic medical centers and by the rapid commercialization of TCR sequencing. While the raw TCR sequencing data has increased, there has been little in the way of approaches to parse the data in a biologically meaningful fashion. The ability to parse this new type of ‘big data’ quickly and efficiently to understand the T cell repertoire in a structurally relevant manner has the potential to open the way to new discoveries about how the immune system is able to respond to insults such as cancer and infectious diseases.

**Methods**

Here we describe a novel method utilizing phylogenetic and sequencing analysis to visualize and quantify TCR repertoire diversity. To demonstrate the utility of the approach, we have applied it to understanding the shaping of the CD8 T Cell response to self (Kb-TRP2) and foreign (Kb-SIY) antigens in naïve and tumor bearing B6 mice. Additionally, this method was applied to tumor infiltrating lymphocytes (TIL’s) from patients undergoing Nivolumab (anti-PD-1) therapy in a clinical trial for metastatic melanoma to understand TCR repertoire characteristics between responders and non-responders.

**Results**

Analysis of the naïve CD8 response to SIY showed a lower clonality yet more closely structurally related response whereas CD8 responses to TRP2 were highly clonal yet less structurally related. Presence of tumor exhibited interesting differential effects on SIY vs. TRP2. We believe that differences in TCR repertoire suggest effects from central and peripheral tolerance on self vs. foreign antigens. In clinical trial data, the phylogenetic analysis revealed unique TCR repertoire signatures that differentiated responders from non-responders to anti-PD-1 therapy, including some that could be detected prior to initiation of therapy. Additionally, this analysis revealed that patients whose CD8 response had a larger contribution from novel and unique structural clones responded better to therapy.

**Conclusions**

In summary, we have developed and demonstrated a novel method to meaningfully parse and interpret TCR repertoire data and have applied it to yield a novel understanding of CD8 T Cell responses to different types of antigens as well as key characteristics in those who respond to anti-PD-1 therapy.

**Fig. 1:**
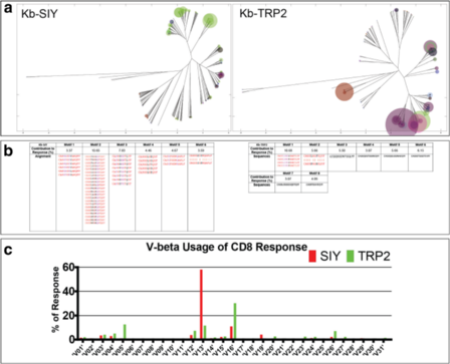
**(Abstract O2). a** Weighted Phylogenetic Trees Comparing Kb-SIY vs Kb-TRP2 TCR Repertoire. Size of circles proportional to frequency of sequence. Color of circle corresponds to V-Beta Usage. **b** Dominant Motifs gathered from phylogenetic trees determined by homologous sequences and their contribution to the response **c** V-Beta Usage of Kb-SIY vs Kb-TRP2 Response

**Fig. 2:**
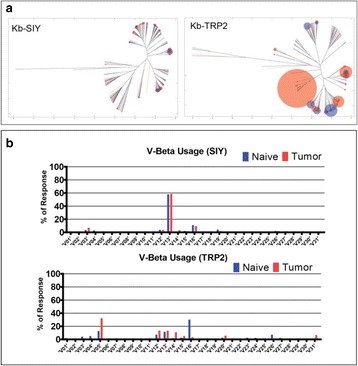
**(Abstract O2). a** Weighted Phylogenetic Trees comparing Naive to Tumor-Bearing TCR Repertoire from spleen. Size of Circles proportional to frequency of sequence. Blue Circles = Naive Repertoire. Red Circles = Tumor-Bearing Repertoire. **b** V-Segment Usage of Kb-SIY vs Kb-TRP2 Responses for Naive vs Tumor-Bearing Response

**Fig. 3:**
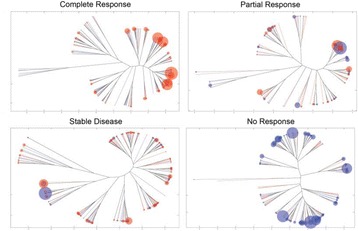
**(Abstract O2).** Examples of weighted phylogenetic trees from four cohorts of responders on anti-PD-1 therapy. Size of circles proportional to frequency of sequence. Blue = TCR repertoire prior to therapy. Red = TCR repertoire on therapy

### O3 Increased STAT3 signaling and decreased suppressive function of regulatory T cells are biomarkers of positive patient outcome to nivolumab therapy

#### David Woods^1^, Anders Berglund^2^, Rupal Ramakrishnan^2^, Andressa Sodre^1^, Jeffrey Weber^1^

##### ^1^NYU Langone Medical Center, New York, NY, USA; ^2^H. Lee Moffitt Cancer Center, Tampa, FL, USA

###### **Correspondence:** David Woods (david.woods@nyumc.org)

**Background**

Antibody-mediated blockade of the inhibitory receptor PD-1 on T cells has shown clinical efficacy in the treatment of various malignancies. However, biomarkers of response and mechanisms of resistance remain largely unidentified. To address this gap, we sought to identify the role(s) of regulatory T cells (Tregs) in metastatic melanoma patients treated with the PD-1 antibody nivolumab.

**Methods**

Pre and post-treatment Tregs were isolated from the peripheral blood of surgically resected stage III/IV metastatic melanoma patients treated with adjuvant nivolumab. Suppressive capacity was assessed in an allogeneic mixed lymphocyte reaction. Paired (pre vs. post-treatment) Tregs were assessed by flow cytometry for phosphorylated STAT3 (pSTAT3) expression. Finally, paired Treg samples were assessed for gene expression by RNA-sequencing.

**Results**

Tregs from non-relapsing patients demonstrated a significant decrease in suppressive capacity post-treatment (p < 0.05). However, suppressive capacity in relapsing patients did not decrease and their Tregs were significantly more suppressive post-treatment relative to non-relapsers (p < 0.01). Significantly increased levels of pSTAT3 post treatment were observed in non-relapsers (p < 0.05) but not in relapsers (p < 0.40). Significantly increased pSTAT3 was not seen in conventional T cells after nivolumab therapy. Culturing treatment-naïve T cells with PD-1 blocking antibodies *in vitro* resulted in increased levels of pSTAT3 in Tregs compared to IgG controls (p < 0.01). *In vitro* PD-1 blockade also significantly increased the number of Tregs (p < 0.01), and significant increases were seen in paired patient samples (p < 0.05). Paired analysis of Treg RNA-seq data using Panther and GeneGo. Metacore showed several significantly increased pathways associated with proliferation in non-relapsers. Changes in these pathways were absent in relapsers. Gene Set Enrichment Analysis of non-relapser Tregs showed significant (q=8.2e-18) overlap with known STAT3 target genes. Conversely, Enrichr analysis of relapsers showed significant upregulation of STAT1 and STAT2 target genes. No overlap of significantly changed gene expression or pathways in Tregs vs. conventional CD4+ T cells were observed.

**Conclusions**

These results highlight the potential importance of Tregs in mediating benefit with PD-1 blockade, demonstrating pSTAT3 induction and reduced suppressive capacity as biomarkers of clinical benefit. PD-1 blockade also increased the percentages of Tregs, consistent with the known roles of STAT3 in promoting cell survival and proliferation. RNA-seq data demonstrated increased STAT3 and proliferation associated gene expression. Intriguingly, Tregs from relapsing patients had increased expression of genes associated with STAT1/2 signaling, warranting further investigation of these pathways. In addition to highlighting STAT signaling as a biomarker of relapse, these results demonstrate distinct differences in the impact of PD-1 blockade in Treg vs. conventional T cells.

### O4 Analysis of pharmacodynamic biomarkers in the first in-human trial of GITR co-stimulation with the agonist antibody TRX-518 in advanced solid cancer patients

#### Roberta Zappasodi^1^, Yanyun Li^1^, Jingjing Qi^2^, Philip Wong^2^, Cynthia Sirard^3^, Michael Postow^4^, Walter Newman^3^, Henry Koon^5^, Vamsidhar Velcheti^6^, Margaret K Callahan^7^, Jedd D Wolchok^4^, Taha Merghoub^1^

##### ^1^Ludwig Collaborative Laboratory, Memorial Sloan Kettering Cancer Center, New York, NY, USA; ^2^Immune Monitoring Core Facility, Memorial Sloan Kettering Cancer Center, New York, NY, USA; ^3^Leap Therapeutics, Cambridge, MA, USA; ^4^Department of Medicine, Memorial Sloan Kettering Cancer Center, New York, NY, USA; ^5^Case Western Reserve University, Cleveland, OH, USA; ^6^Cleveland Clinic Main Campus, Cleveland, OH, USA; ^7^Memorial Sloan Kettering Cancer Center, New York, NY, USA

###### **Correspondence:** Roberta Zappasodi (zappasor@mskcc.org)

**Background**

GITR is a tumor necrosis factor receptor expressed at high levels on regulatory T cells (Tregs) and up-regulated on T cells upon activation. GITR stimulation abrogates Treg suppression and enhances T cell effector function. These observations suggest that GITR could be an attractive target for immunotherapy with agonist antibodies. GITR stimulation in tumor-bearing mice has shown therapeutic activity associated with both Treg reduction and modulation. Here we report results of pharmacodynamic analyses in the first in-human phase I trial with the fully humanized agonist anti-GITR antibody TRX518 as monotherapy in patients with advanced refractory solid tumors.

**Methods**

Patients were accrued to 9 cohorts (up to 6 patients/cohort) to receive a single dose of TRX518 (dose range: 0.0001-8 mg/kg). Pharmacodynamic analyses included flow cytometric evaluation of frequency and phenotype of circulating T cells and cytokine quantification in serum samples at different time points up to 12 weeks after treatment. Relevant changes observed with these analyses were monitored in pre- and post-treatment tumor biopsies by immunofluorescence staining.

**Results**

Here we report results obtained in 37 patients treated with ≥0.005 mg/kg TRX518 (cohorts 3-9), including 6 melanoma, 7 non-small cell lung cancer (NSCLC) and 7 colorectal cancer (CRC) patients and 17 patients with 11 other solid tumors. Among the T cell parameters analyzed, we found frequent reduction in circulating Tregs after treatment with TRX518 across all cohorts, with some exceptions. Importantly, this effect could be maintained over the 12-week observation period. When the analysis was performed by disease type, it revealed a pronounced TRX518 dose-dependent down-regulation of peripheral Tregs in both melanoma and CRC patients. Interestingly, in NSCLC cancer patients, Tregs did not always decrease after treatment. In a subset of patients (n=6; 2 melanoma, 2 CRC, 2 lung), for whom we had pre- and post-treatment tumor biopsies in addition to PBMCs, we tested whether intra-tumor Tregs were consistently affected. In melanoma and CRC patients, intra-tumor Foxp3^+^ Tregs were significantly reduced after treatment, in agreement with the peripheral Treg down-modulation observed in the same patients. In lung cancer patients, lack of circulating Treg reduction was consistently associated with stable or increased intra-tumor Treg infiltration after TRX518.

**Conclusions**

Circulating Treg reduction is a potential pharmacodynamic biomarker of TRX518 biological activity. This parameter may allow predictive correlation with changes in intratumoral Treg infiltration. We plan to further investigate this effect and its relevance for the association with clinical responses in our recently opened TRX518 multi-dose study.

**Trial Registration**

ClinicalTrials.gov identifier NCT01239134.

**Consent**

Written informed consent was obtained from the patient for publication of this abstract and any accompanying images. A copy of the written consent is available for review by the Editor of this journal.

## Bispecific Antibodies

### O5 Clinical responses in advanced pancreatic patients treated with bispecific antibody armed T cells (BATS)

#### Lawrence G. Lum^1^, Minsig Choi^2^, Archana Thakur^1^, Abhinav Deol^3^, Gregory Dyson^3^, Anthony Shields^3^

##### ^1^University of Virginia Cancer Center, Charlottesville, VA, USA; ^2^Stony Brook University Medical Center, Stony Brook, NY, USA; ^3^Karmanos Cancer Institute, Detroit, MI, USA

###### **Correspondence:** Lawrence G. Lum (lgl4f@virginia.edu)

**Background**

Conventional chemotherapy (chemo) for locally advanced pancreatic cancer (LAPC) and metastatic pancreatic cancer (PC) is associated with dismal responses and poor survival rates. Arming activated T cells (ATC) with anti-CD3 x anti-EGFR bispecific antibody (EGFRBi) turns every ATC into a non-MHC restricted EGFR-specific cytotoxic T lymphocyte [1]. Engagement of CD3 on T cells and EGFR on Mia PACA-2 leads to cytokine secretion, proliferation, cytotoxicity by ATC and inhibition of tumor growth [2]. An earlier study using Infusions of anti-CD3 x anti-HER2 (HER2Bi) armed ATC in metastatic breast cancer provided encouraging survival (OS = 36 months) and evidence of anti-breast cancer immunity [3].

**Methods**

In this study, we used anti-CD3 x anti-EGFR bispecific antibody (EGFRBi)-armed T cells (EGFR BATs) to target EGFR in 5 metastatic PC patients and 6 colorectal cancer patients treated at Karmanos Cancer Institute on Protocol #2014-025 in a phase I dose escalation involving 3 weekly infusions of 10, 20, and 40 x 10^9^ BATs/infusion followed by a booster infusion 3 months later.

**Results**

In the 5 PC patients, we report 1 patient was stable for 6.5 months and 2 patients in whom infusions of EGFR BATs may have “sensitized” the tumor to subsequent chemotherapy. The patient with stable disease had a near partial response. The median overall survival in 5 patients is 23.5 months with the median time to progression (TTP) of 7.0 months. Patient IT20102 received BATs and was stable (decreased marker lesion by 27%) at 6.5 mos. IT20091 had a remarkable clinical response to chemotherapy after progressing after immunotherapy at 4.6 months. After 3 BATs infusions, patient IT2010 had a “flare” or progression and subsequently had a complete response to Xeloda and remains in remission. This phase I study shows: 1) long-term stabilization in one patient; 2) a persistent complete responder after BATs "progression" followed by chemotherapy; 3) improved chemotherapy responsiveness after EGFRBi-BATs therapy; and 4) two patients with slow progressive disease who survived beyond 400 days. Survival for the 5 patients was 13.6, 14.5, 23.3 (alive in CR), 24.9 (alive, stable), and 31.0 months after enrollment, respectively (as of 7-20-16).

**Conclusions**

Targeting PC with EGFR BATs resulted in improved survival and remarkable post-immunotherapy chemotherapy responses in a small series of patients. The series provides evidence for anti-tumor activity of EGFR BATs as well as evidence that BATs infusions can sensitize tumors to subsequent chemotherapy.

**Acknowledgements**

Funding for this study was provided by Helen Kay Trust and Philanthropy and Startup Funds at KCI. We acknowledge the efforts of clinical coordinating staff, the clinical trials office staff, GMP laboratory staff, and clinical nursing support staff to making this study possible. The study was conducted at KCI.

**References**

1. Reusch U, Sundarum M, Davol PA, Olson SD, Davis JB, Demel K, *et al*: **Anti-CD3 x anti-EGFR Bispecific Antibody Redirects T Cell Cytolytic Activity to EGFR-Positive Cancers In Vitro and in an Animal Model.** *CCR* 2006, **12**:183-190.

2. Grabert RC, Cousens LP, Smith JA, Olson S, Gall J, Young WB, *et al*: **Human T Cells Armed with Her2/neu Bispecific Antibodies Divide, Are Cytotoxic, and Secrete Cytokines with Repeated Stimulation.** *CCR* 2006, **12**:569-576.

3. Lum LG, Thakur A, Al-Kadhimi Z, Colvin G, Cummings F, Legare R, *et al*: **Targeted T cell Therapy in Stage IV Breast Cancer: A Phase I Clinical Trial.** *CCR* 2015, **21**:2305-2314.

**Table 1:** **(Abstract O5).** Clinical data

Pt	Age	Disease	Prior Tx	BATS (x 10^9^)	TTP (mo)	OS (mo)	Comments
IT20087	58	Mets to liver	Folfirinox	47	6 mo	Died 13.6 mo	Progressed after Immunotherapy
IT20091	63	T3 N1Mets to liver. S/P Whipple	5FU, Leu/5FU Folfirinox	9 79	4.8 mo	Died 31 mo	Folfirinox induced CR after IT and responded a 2^nd^ time to Folfirinox
IT20092	64	T2b Abd Nodes, S/P Whipple	Gemzar, 5FU, radiation	36	7 mo	Died 14.5 mo	Slowly progress with chronic diarrhea
IT20102	56	T4, Mets to liver, lungs	Folfirinox	74	6.5 mo	Alive 24.9 mo	Progressed after 6.5 mo
IT20104	51	T4, Abd Node	FOLFOX, X eloda	72	2.2 mo	Alive 23.3 mo	Chemo Induced CR after IT; On Xeloda

## Combinations: Immunotherapy/Immunotherapy

### O6 Reactivating the anti-tumor immune response by targeting innate and adaptive immunity in a phase I/II study of intratumoral IMO-2125 in combination with systemic ipilimumab in patients with anti-PD-1 refractory metastatic melanoma

#### Cara Haymaker^1^, Marc Uemura^1^, Ravi Murthy^1^, Marihella James^1^, Daqing Wang^2^, Julie Brevard^2^, Catherine Monaghan^2^, Suzanne Swann^2^, James Geib^2^, Mark Cornfeld^2^, Srinivas Chunduru^2^, Sudhir Agrawal^2^, Cassian Yee^1^, Jennifer Wargo^1^, Sapna P Patel^1^, Rodabe Amaria^1^, Hussein Tawbi^1^, Isabella Glitza^1^, Scott Woodman^1^, Wen-Jen Hwu^1^, Michael A Davies^1^, Patrick Hwu^1^, Willem W Overwijk^1^, Chantale Bernatchez^1^, Adi Diab^1^

##### ^1^University of Texas MD Anderson Cancer Center, Houston, TX, USA; ^2^Idera Pharmaceuticals, Inc., Cambridge, MA, USA

###### **Correspondence:** Cara Haymaker (chaymaker@mdanderson.org)

**Background**

While checkpoint inhibitor (CPI) therapy has transformed metastatic melanoma (MM) treatment, many patients remain refractory. We reasoned that combining CPI with an agent that activates antigen presenting cells and improves T cell priming may result in improved response. Our approach is to modulate the tumor microenvironment through intratumoral (i.t.) injection of the TLR9 agonist, IMO-2125, in combination with ipilimumab (ipi). We hypothesize that this will result in dendritic cell (DC) activation and induction of tumor-specific CD8^+^T cells which will synergize with ipilimumab to overcome immune-escape. Based on this rationale we initiated a phase I/II clinical trial.

**Methods**

Adults with refractory MM despite up to 2 lines of CPI including PD-1 blockade therapy (with or without a BRAF inhibitor) are eligible. IMO-2125, in doses escalating from 4mg to 32mg, is given i.t. weeks 1, 2, 3, 5, 8, and 11 along with ipilimumab i.v. 3 mg/kg weeks 2, 5, 8, and 11. Dose-limiting toxicity (DLT) is evaluated using a modified Toxicity Probability Interval design. Primary endpoints are safety, tumor response, and PK. Blood and injected and distal tumor biopsies are obtained pre- and on-treatment. Immune analyses include DC subsets and their activation status as well as T cell activation, function and proliferation. T cell repertoire diversity will be evaluated by high throughput CDR3 sequencing.

**Results**

As of August 2, 2016, 11 pts have been enrolled. DLT has not been observed. Grade 3 hypophysitis (2 subjects) is the only immune-related AE observed to date. No other drug-related grade 3-5 AEs were documented and only 1 subject experienced a grade 2 fever. Five patients are evaluable for response - 2 PR, 2SD, 1PD per investigator assessment. Fresh tumor biopsies show maturation (upregulation of HLA-DR) of the myeloid DC1 subset (CD1c^+^CD303^-^) in the IMO-2125 injected tumor lesion 24 hrs post-treatment compared to pre-treatment biopsy. On-treatment biopsy results are consistent with a higher rate of proliferative (Ki67) effector CD4+ and CD8+ T cells in responders. Cytokine analysis shows a 2-3 fold increase in circulating IFNγ levels compared to pretreatment in responders.

**Conclusions**

Though preliminary, these results demonstrate that the combination of ipi and IMO-2125 is well tolerated with encouraging preliminary activity in a PD-1 refractory population. Dose escalation is ongoing and a phase II expansion will include IMO-2125 in combination with both ipi and anti-PD-1. Updated safety, antitumor activity, and biomarker data will be presented.

**Trial Registration**

ClinicalTrials.gov identifier NCT02644967.

### O7 Clinical safety and efficacy assessment of the CD137 agonist urelumab alone and in combination with nivolumab in patients with hematologic and solid tumor malignancies

#### Erminia Massarelli^1^, Neil H Segal^2^, Vincent Ribrag^3^, Ignacio Melero^4^, Tara C Gangadhar^5^, Walter Urba^6^, Dirk Schadendorf^7^, Robert L Ferris^8^, Roch Houot^9^, Franck Morschhauser^10^, Theodore Logan^11^, Jason J Luke^12^, William Sharfman^13^, Fabrice Barlesi^14^, Patrick A Ott^15^, Laura Mansi^16^, Shivaani Kummar^17^, Gilles Salles^18^, Cecilia Carpio^19^, Roland Meier^20^, Suba Krishnan^20^, Dan McDonald^20^, Matthew Maurer^20^, Xuemin Gu^20^, Jaclyn Neely^20^, Satyendra Suryawanshi^20^, Ronald Levy^17^, Nikhil Khushalani^21^

##### ^1^University of Texas MD Anderson Cancer Center, Houston, TX, USA; ^2^Memorial Sloan Kettering Cancer Center, New York, NY, USA; ^3^Institut Gustave Roussy, Villejuif, Ile-de-France, France; ^4^Center for Applied Medical Research (CIMA), University of Navarra, Pamplona, Navarra, Spain; ^5^University of Pennsylvania, Philadelphia, PA, USA; ^6^Earle A. Chiles Research Institute, Providence Cancer Center, Portland, OR, USA; ^7^Universitätsklinikum Essen, Essen, Nordrhein-Westfalen, Germany; ^8^University of Pittsburgh, Pittsburgh, PA, USA; ^9^CHU Rennes, Service Hématologie Clinique and INSERM 0203, Unité d'Investigation Clinique, Rennes, Bretagne, France; ^10^Centre Hospitalier Régional Universitaire de Lille, Lille, Nord-Pas-de-Calais, France; ^11^Simon Cancer Center, Indiana University, Indianapolis, IN, USA; ^12^University of Chicago School of Medicine, Chicago, IL, USA; ^13^Johns Hopkins University School of Medicine, Lutherville, MD, USA; ^14^Multidisciplinary Oncology and Therapeutic Innovations, Hôpital Nord, Marseille, Provence-Alpes-Cote d'Azur, France; ^15^Dana-Farber Cancer Institute, Boston, MA, USA; ^16^Centre Hospitalier Régional Universitaire Hôpital Jean Minjoz, Besançon, Franche-Comte, France; ^17^Stanford University School of Medicine, Stanford, CA, USA; ^18^Hospices Civils de Lyon-Université de Lyon, Pierre Benite, Auvergne, France; ^19^Hospital Universitari Vall d'Hebron, Universitat Autònoma de Barcelona, Barcelona, Catalonia, Spain; ^20^Bristol-Myers Squibb, Princeton, NJ, USA; ^21^H. Lee Moffitt Cancer Center, Tampa, FL, USA

###### **Correspondence:** Erminia Massarelli (emassarelli@coh.org)

**Background**

Urelumab is a fully human CD137 agonistic monoclonal antibody (mAb) that enhances T cell and natural killer (NK) cell antitumor activity in preclinical models. Nivolumab, a fully human programmed death-1 (PD-1) mAb that blocks the inhibitory function of the PD-1 receptor on T cells, has shown single-agent activity in many advanced malignancies. We hypothesized that the distinct, non-redundant mechanisms of these two mAbs could enhance antitumor activity without compromising safety. Here we report safety/tolerability, pharmacodynamics, and preliminary efficacy of urelumab and urelumab plus nivolumab combination therapy in patients with advanced malignancies.

**Methods**

The monotherapy study evaluated urelumab in patients with advanced malignancies (0.1 or 0.3 mg/kg Q3W) or advanced non-Hodgkin lymphoma (8 mg Q3W or Q6W). The combination study evaluated urelumab (3 or 8 mg Q4W) plus nivolumab (3 mg/kg or 240 mg Q2W) in patients with advanced solid tumors or B cell lymphoma (dose escalation) or patients with diffuse large B cell lymphoma (DLBCL), melanoma, non-small cell lung cancer (NSCLC), or squamous cell carcinoma of the head and neck (SCCHN; cohort expansion). Based on preliminary safety/tolerability/pharmacokinetic assessments of urelumab, cohort expansion focused on flat doses of 8 mg.

**Results**

**Fig. 4:**
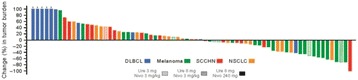
**(Abstract O7).** Best percent reduction in target lesion tumor burden with urelumab plus nivolumab

Overall, patients who received urelumab monotherapy (N=123) experienced infrequent treatment-related serious AEs (7%) and treatment-related AEs (TRAEs) leading to discontinuation (5%; Table 2). In 104 patients treated with urelumab plus nivolumab (melanoma, n=40; NSCLC, n=20; SCCHN, n=22; DLBCL, n=22), the most frequent TRAE was fatigue (26%); grade 3/4 ALT/AST elevations (3%/3%) and TRAEs leading to discontinuation (7%) were infrequent. No treatment-related deaths were reported. Urelumab stimulated peripheral IFN-γ–induced cytokine production; induction was greater with urelumab plus nivolumab. In most melanoma tumors evaluated, a trend toward increased T and NK cell number and expression of IFN-γ and CXCL9 was observed upon treatment with the combination. Six patients with lymphoma treated with urelumab monotherapy had a partial (n=3) or complete (n=3) remission. Nine of 86 evaluable patients treated with the combination had partial responses (melanoma, n=8; SCCHN, n=1); no patients with NSCLC or DLBCL had confirmed responses at the interim analysis. Of 71 patients treated with urelumab plus nivolumab with RECIST/IWG assessments, 33 had reductions in tumor burden (Fig. 4).

**Table 2:** **(Abstract O7).** Treatment-related safety events

Patients, n (%)	Urelumab monotherapy N=23	Urelumab + nivolumab N=104
Treatment-related AEs	65 (53)	65 (53)
Most frequent treatment-related AEs^a^		
Fatigue	18 (15)	27 (26)
AST increased	16 (13)	9 (9)
ALT increased	12 (10)	13 (13)
Treatment-related grade ¾ AST elevation	4 (3)	3 (3)
Treatment-related ¾ ALT elevation	3 (2)	3 (3)
Treatment-related serious AEs	9 (7)	10 (10)
Treatment-related AEs leading to discontinuation	6 (5)	7 (7)
Treatment-related deaths	0	0

AE, adverse event; AKT, alanine aminotransferase; AST, aspartate aminotransferase.

^a^Treatment-related AEs occurring in ≥10% of all patients

**Conclusions**

Urelumab with or without nivolumab is safe/tolerable at flat and weight-based doses of 8 mg and 0.1 mg/kg. Although urelumab has demonstrated single-agent pharmacodynamic and antitumor activity in lymphoma, combination with nivolumab did not appear to provide significant additive/synergistic clinical benefit at the doses evaluated.

**Trial Registration**

ClinicalTrials.gov identifier NCT01471210 and NCT02253992.

### O8 Beyond immune checkpoint: first-in-class antibody targeting soluble NKG2D ligand sMIC for cancer immunotherapy

#### Jennifer Wu, Jinyu Zhang, Fahmin Basher, Mark Rubinstein

##### Medical University of South Carolina, Charleston, SC, USA

###### **Correspondence:** Jennifer Wu (wujjd@musc.edu)

**Background**

In response to oncogenic insult, human cells were induced to express a family of MHC I-chain related molecules A and B (MICA and MICB, generally termed MIC) on the surface which serve as the ligands for the activating immune receptor NKG2D expressed by all human NK, CD8 T, NKT, and subsets of gamma-delta T cells. Theoretically, engagement of NKG2D by tumor cell surface MIC is thought to signal and provoke the immune system to eliminate transformed cells. Clinically, almost all advanced tumors in cancer patients produce soluble MIC through proteolytic shedding mediated by metalloproteases, or by release in exosomes derived from the cell membrane. Tumor-derived sMIC is known to be highly immune suppressive and profoundly insults the immune system by downregulating receptor NKG2D expression on effector NK and T cells, driving the expansion of tumor-favoring myeloid suppressor cells, skewing macrophages into alternatively activated phenotypes, and perturbing NK cell peripheral maintenance. High levels of serum sMIC significantly correlate with advanced diseases of many types of cancer. These observations clearly endorse sMIC to be a cancer immune therapeutic target. However, due to mice lacking homologues to human MIC, this concept was not proven until our recent studies.

**Methods**

Using a “humanized” MIC-transgenic spontaneous mouse model which recapitulates the NKG2D-mediated onco-immune dynamics of human cancer patients, we addressed whether sMIC is a cancer immunotherapeutic target and whether antibody targeting sMIC synergizes with immune checkpoint blockade or adoptive T or NK cell therapy.

**Results**

We show that therapy with a first-in-field non-blocking antibody B10 that does not block the interaction of MIC with NKG2D revamps endogenous innate and antigen-specific CD8+ T cell responses and remodels immune reactive tumor microenvironment, by restoring NK cell hemostatic maintenance and function, enhancing CD8+ T cell infiltration to tumors and TCR clonality/diversity, modulating CD8+ T cells metabolic preferences, eliminating MDSCs and TAMS. Anti-sMIC stand-alone therapy resulted in effective debulking of primary tumors and eliminated metastasis. Using multiple pre-clinical animal models, we further demonstrate that antibody B10 neutralizing sMIC synergizes with CTLA-4 and PD-1/PD-L1 checkpoint blockade therapy and adoptive cell based therapy with no observed toxicity.

**Conclusions**

Our study has launched a new avenue of cancer immunotherapy which can be readily translated into clinical trials.

**Fig. 5:**
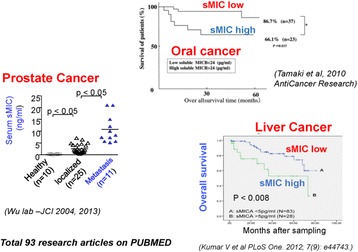
**(Abstract O8).** These are the examples in human cancer patients, prostate cancer, Oral cancer, and HBV-induced Liver cancer, where high levels of circulating sMIC correlates with advanced disease stages and poor survival.

**Fig. 6:**
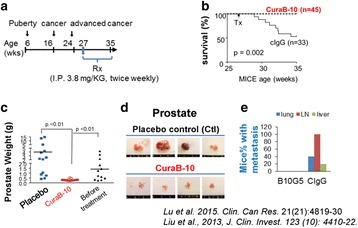
**(Abstract O8). a**. Therapy of the clinically relevant spontaneous prostate tumor TRAMP/MICB model (Liu et al, 2013, JCI 123 (10) 4410 ) with CuraB10 (also called B10G5) or control IgG (placebo) at advanced stage via I.P. injection at the dose of 3.8 mg/KG body weight twice weekly for 8 weeks (**b**). Mice with CuraB-1o therapy all enjoyed longtime survived whereas mice in placebo group are succumbed to cancer (**c**). Prostate weight. Comparisons made between Placebo group and CuraB-10 group and between before and after treatment of CuraB-10. (**d**). Representative images of the prostate. Top showing large tumor burden. Bottom showing normal prostate size. (**e**). All mice in the control group developed metastasis whereas no metastasis was detected in animals received CuraB-10 therapy. In summary, the data demonstrate that CuraB-10 stand-alone therapy can effectively induce regression of primary tumors and eliminate metastasis

**Fig. 7:**
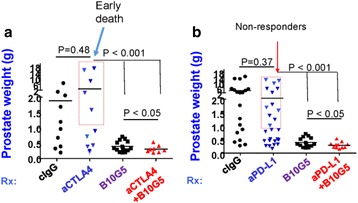
**(Abstract O8).** We further addressed the synergistic effect of CuraB-10 therapy with FDA approved checkpoint blockade therapy using the clinically relevant TRAMP/MIC spontaneous prostate tumor mouse model. Two points: 1) a percentage of TRAMP/MIC mice do not respond to checkpoint (CTLA4 or PD-1) blockade therapy, whereas all TRAMP/MIC mice are responsive to CuraB-10 therapy; also, a population of TRAMP/MICB animals died at 3-4 weeks of CTLA4 Rx alone. 2) CuraB-10 synergizes with checkpoint blockade (CTLA4 or PD-1) therapy when used in combination

**Fig. 8:**
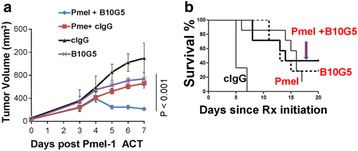
**(Abstract O8).** Because rodents do not express MIC, we engineered mouse melanoma B16 tumor cells to express sMIC (B16-sMIC). We implanted B16-sMIC into syngeneic host. When tumors grew to 50-100mm3 in size, treatment starts. Four treatments were given: Adoptive transfer of melanoma antigen-specific Pmel CD8 T cells once, B10G5 (CuraB-10) twice 2 week Adoptive transfer of melanoma antigen-specific Pmel CD8 T cells once, control IgG (cIgG) twice 2 week B10G5 alone 4) cIgG alone Tumor growth curve demonstrating that treatment with B10G5 (CuraB-10) effectuates the effect of Pmel CD8 T therapy Survival curve. Tumor volume of 1000mm3 was defined as survival end point. In one experiment, 2/7 animals received B10G5 and Pmel therapy had complete tumor regression. Note: currently Adoptive T cell transfer (ACT) requires prior-depletion of patient’s immune cells with chemotherapy to be effective. With B10G5 therapy, not only lymph depletion is not required prior to ACT, but also ACT is more effective

## Diet, Exercise and/or Stress and Impact on the Immune System

### O9 β-adrenergic signaling induced by cool housing temperatures mediates immune suppression and impairs the efficacy of anti-PD-1 checkpoint blockade immunotherapy in laboratory mice

#### Mark Bucsek, Guanxi Qiao, Cameron MacDonald, Bonnie Hylander, Elizabeth Repasky

##### Roswell Park Cancer Institute, Buffalo, NY, USA

###### **Correspondence:** Mark Bucsek (mark.bucsek@roswellpark.org)

**Background**

Recent work from our laboratory has shown that anti-tumor immunity is suppressed in mice housed at standard temperatures (ST; 22°C) which could be reversed by housing mice at warmer, thermoneutral temperatures (TT; 30°C) [1]. However, the mechanisms causing this impairment at ST remain unclear. Cold stress is mediated specifically by activation of the sympathetic nervous system and the release of norepinephrine (NE), which is highly suppressive when signaling through β-adrenergic receptors (β-ARs) on immune cells. We found that NE levels are significantly elevated in tumor-bearing mice housed at ST compared to TT, which led us to hypothesize that chronic stress induced by cool housing temperatures increases β-AR signaling that dampens the anti-tumor immune response and the efficacy of immune modulating therapies.

**Methods**

We used both physiologic (housing temperature; ST and TT) and pharmacologic blockade (β-blockers) to modulate β-AR signaling levels in immune-competent and SCID mice bearing 4T1 or B16-OVA tumors. Flow cytometry was used for immune cell analysis. Anti-PD-1 checkpoint blockade was given in 6, 200μg doses (Days 0, 2, 4, 6, 9, and 12) starting the day after tumors became detectable.

**Results**

We found that the addition of β-blockade significantly delayed 4T1 and B16-OVA tumor growth in mice housed at ST, recapitulating the slower tumor growth observed in mice housed at TT. However, β-blockade had no impact on tumor growth in SCID mice at ST or TT indicating dependence on the adaptive immune system. Analysis of 4T1 and B16-OVA tumors from immune-competent mice showed increased IFN-γ expression in both CD4+ and CD8+ T cells in mice treated with β-blockade indicating a more robust anti-tumor immune response. Lastly, we investigated the impact of β-AR signaling on anti-PD-1 checkpoint blockade efficacy and found that reducing β-AR signaling by both physiologic (TT) and pharmacologic (β-blockade) strategies improved responses in both tumor models. Further analysis of 4T1 tumors from mice treated with β-blockade and anti-PD-1 showed an increase in IFN-γ, producing CD8+ T cells compared to either β-blockade or anti-PD-1 alone.

**Conclusions**

Taken together, these data indicate that elevated β-AR stress signaling caused by cool housing temperatures impairs anti-tumor immunity and the response of tumors to anti-PD-1 checkpoint blockade.

**Acknowledgements**

Supported by: The Peter T. Rowley Breast Cancer Research Grant, The Harry J. Lloyd Charitable Trust, the Roswell Park Alliance Foundation, and 5T32CA085183-12.

**Reference**

1. Kokolus K, *et al*: **Baseline tumor growth and immune control in laboratory mice are significantly influenced by subthermoneutral housing temperature**. *PNAS* 2013, **110**:20176-20181.

## Immune Metabolism

### O10 NAD-Sirt1 axis is central to the unique immuno-metabolic phenotype of Th1/17 hybrid cells in regulating its enhanced anti-tumor potential

#### Shilpak Chatterjee^1^, Anusara Daenthanasanmak^1^, Paramita Chakraborty^1^, Kyle Toth^1^, Megan Meek^1^, Elizabeth Garrett-Mayer^1^, Michael Nishimura^2^, Chrystal Paulos^1^, Craig Beeson^1^, Xuezhong Yu^1^, Shikhar Mehrotra^1^

##### ^1^MUSC, Charleston, SC, USA; ^2^Loyola Cancer Center, Maywood, IL, USA

###### **Correspondence:** Shilpak Chatterjee (chatherj@musc.edu)

**Background**

Th17 cells hold promise for immunotherapy of cancer [1]. While the anti-tumor potential of Th17 cells primarily depends upon IFN-γ secretion and persistence [1], a long-term tumor control has still remained elusive. Given that both the “effector” and “stemness like” features are prerequisites for T cells to mount durable anti-tumor responses, we hypothesized that combining the culture conditions of Th1 (effector) and Th17 (stemness like) cells could generate hybrid Th1/17 cells with improved anti-tumor properties.

**Methods**

Melanoma epitope tyrosinase reactive CD4^+^ T cells obtained from h3T TCR transgenic mice were differentiated *ex vivo* to Th1, Th17, and Th1/17 cells before adoptive transfer (0.25×10^6^ cells/animal i.v.) to C57BL/6 recipient animals with subcutaneously established B16 melanoma. Quantitative PCR (q-PCR), flow cytometry, and metabolomic analyses were used to evaluate the expression of various metabolism and stemness associated genes as well as protein expression in the T cells. To compare the metabolic commitment between different subsets (Th1, Th17 and Th1/17), real time metabolic flux analyzer (Seahorse Biosciences, USA) and radioactive tracer studies were used.

**Results**

The combined culture conditions of Th1 and Th17 generates hybrid Th1/17 cells with a IFN-γ^hi^, IL17^hi^, GM-CSF^hi^, CD107a^hi^, T-bet^hi^, Granzyme B^hi^, IL23R^hi^, IL22^hi^, Bcl6^hi^, Tcf7^hi^ signature. These hybrid Th1/17 cells exhibit enhanced tumor control in subcutaneous and lung metastasis models of murine melanoma. A hypothesis generating transcriptional, metabolic, and proteomic profiling, followed by confirmatory experiments established that the enhanced anti-tumor properties were attributed to increased NAD+ mediated activity of histone deacetylase Sirt1 in hybrid Th1/17 cells. Inhibition of NAD+ and Sirt1 activity either pharmacologically or by genetic ablation (Sirt1-KO T cells) led to loss of stable anti-tumor control. Importantly, anti-tumor T cells or tumor infiltrating lymphocytes programmed in the presence of exogenous NAD+ also led to the similar metabolic phenotype and improved anti-tumor control.

**Conclusions**

The present study discloses that metabolic status plays an important role in dictating the anti-tumor response of the T cells. Combining the culture conditions of Th1 and Th17 cells renders hybrid Th1/17 cells with a unique immune-metabolic feature that enables them to orchestrate distinct transcriptional programs leading to highly effector and stem-like T cells.

**Reference**

1. Muranski P, Boni A, Antony PA, *et al*: **Tumor-specific Th17-polarized cells eradicate large established melanoma.** *Blood* 2008, **112**:362-373.

### O11 The Wnt5a-beta-catenin pathway triggers a metabolic switch that drives indoleamine 2,3-dioxygenase activity and dendritic cell tolerization in the melanoma microenvironment: optimizing checkpoint inhibitor immunotherapy

#### Fei Zhao^1^, Kathy Evans^1^, Christine Xiao^1^, Alisha Holtzhausen^2^, Brent A. Hanks^1^

##### ^1^Duke University Medical Center, Durham, NC, USA; ^2^Lineberger Comprehensive Cancer Center, University of North Carolina, Chapel Hill, NC, USA

###### **Correspondence:** Brent A. Hanks (hanks004@mc.duke.edu)

**Background**

Despite recent advances, many cancers remain refractory to available immunotherapies by developing various strategies to evade the immune system. Emerging evidence indicates that the tolerization of local dendritic cells (DCs) within the tumor microenvironment plays a critical role in immune evasion. The role of metabolic re-programming in DC tolerization remains poorly characterized and the mechanisms by which cancers may utilize these pathways to promote the establishment of an immunotolerant microenvironment have not been described.

**Methods**

We investigated the role of the Wnt-beta-catenin pathway in the metabolic reprogramming of melanoma-derived DCs using real-time metabolic flux analysis. The impact of DC metabolic re-programming on the enzymatic activity of indoleamine 2,3-dioxygenase (IDO) was analyzed by HPLC while protoporphyrin IX(PpIX) levels were quantified by flow cytometry. The role of DC fatty acid oxidation (FAO) on regulatory T cell (Treg) generation was investigated using pharmacologic and genetic approaches. The impact of FAO inhibition on anti-tumor immune responses to anti-PD-1 antibody therapy were investigated in a transgenic melanoma model.

**Results**

**Fig. 9:**
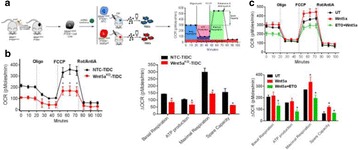
**(Abstract O11).** Wnt5a Promotes DC FAO in the Melanoma Microenvironment. A. Schematic of tumor-infiltrating DC (TIDC) metabolic analysis. B. Melanoma-derived Wnt5a promotes TIDC OXPHOS. C. Wnt5a promotes DC FAO

**Fig. 10:**
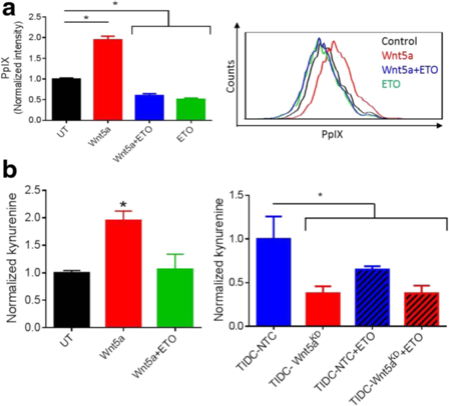
**(Abstract O11).** Wnt5a-induced FAO Promotes DC Synthesis of PpIX and Enhances IDO Enzyme Activity. A. Wnt5a stimulates DC PpIX synthesis. B,C. Wnt5a promotes DC IDO activity in a FAO-dependent manner both *in vitro* and *in vivo*

**Fig. 11:**
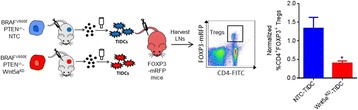
**(Abstract O11).** Wnt5a-induced DC OXPHOS Promotes Treg Generation in the Melanoma Microenvironment. Melanoma-derived Wnt5a conditions DCs to promote Treg generation *in vivo*

**Fig. 12:**
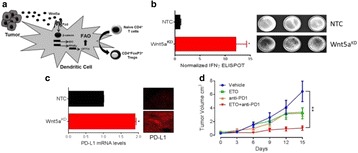
**(Abstract O11).** Inhibition Wnt5a-DC FAO Enhances Melanoma PD-L1 Expression and Augments anti-PD-1 antibody Efficacy. A. Schematic of Wnt5a paracrine signaling pathway. B,C. Genetic silencing of Wnt5a in melanoma promotes T cell infiltration and PD-L1 upregulation. D. Inhibition of CPT1A/FAO synergizes with anti-PD-1 antibody therapy in melanoma

We show that the Wnt5a-beta-catenin-PPARg pathway shifts DCs from glycolysis to FAO in the melanoma microenvironment in a manner dependent upon induction of the mitochondrial fatty acid transporter, CPT1A (Fig. 9). This metabolic shift promotes DC tolerization by 1) elevating DC levels of the PpIX prosthetic group of IDO, resulting in the enhanced activity of this enzyme (Fig. 10) and 2) potently suppressing DC-expression of IL-6 and IL-12, both culminating in the generation of Tregs both *in vitro* and *in vivo* (Fig. 11). Genetic silencing and the pharmacologic inhibition of CPT1A potently enhances the ability of DCs to stimulate effector T cell responses. Indeed, genetic silencing of melanoma-expressed Wnt5a significantly promotes T cell tumor infiltration and augments PD-L1 expression in this melanoma model. Consistent with these findings, we further show FAO inhibition to enhance the efficacy of anti-PD-1 therapy while augmenting melanoma antigen-specific T cell responses (Fig. 12).

**Conclusions**

Our findings implicate the Wnt5a-beta-catenin-PPARg-CPT1A paracrine signaling axis as a driver of DC FAO and functional DC tolerization in the melanoma microenvironment and connect this pathway with the promotion of a “non-inflamed” phenotype in melanoma. This work describes a novel association between DC metabolism and the regulation of IDO enzymatic activity and suggests that this pathway may be a potent pharmacological target for increasing the responsiveness of “non-inflamed” tumors to anti-PD-1 antibody immunotherapy.

### O12 Mitochondrial biogenesis is repressed in tumor-infiltrating CD8+ T cells resulting in metabolic insufficiency and T cell dysfunction

#### Nicole Scharping^1^, Ashley V Menk^2^, Rebecca Moreci^2^, Ryan Whetstone^1^, Rebekah Dadey^1^, Simon Watkins^1^, Robert Ferris^1^, Greg M Delgoffe^1^

##### ^1^University of Pittsburgh, Pittsburgh, PA, USA; ^2^University of Pittsburgh Cancer Institute, Pittsburgh, PA, USA

###### **Correspondence:** Nicole Scharping (nes63@pitt.edu)

**Background**

CD8^+^ tumor-infiltrating T lymphocytes (CD8^+^ TIL) in the tumor microenvironment (TME) are unable to effectively control their tumor targets due to a variety of immunosuppressive mechanisms, including direct tumor cell-T cell inhibition and soluble immunosuppressive factors. This allows cancer to progress unchecked as T cells are rendered functionally inert. Recently, poor metabolite availability in the TME has been identified as an additional suppressive mechanism exploited by bioenergetically-dysregulated tumors. Because T cell activation also has robust metabolic demands, we hypothesized that CD8^+^ TIL dysfunction was a result of metabolic insufficiency.

**Methods**

Metabolic capacity was measured at the single cell level by 2NBDG and MitoTracker FM. Metabolic output was measured by Seahorse extracellular flux analysis. T cell reprogramming was performed by retroviral transduction on OVA-specific transgenic T cells *in vitro* before adoptive transfer into B16^OVA^ bearing mice.

**Results**

We found CD8^+^ TIL are characterized by dramatic loss of mitochondrial mass in B16, MC38, and LLC implantable mouse tumors and human CD8^+^ TIL, which correlates with upregulation of co-inhibitory checkpoint molecules PD-1 and Tim-3. CD8^+^ TIL mitochondrial mass loss is caused by decreased mitochondrial biogenesis, due in part to repression of the transcriptional co-activator PGC1α resulting from chronic Akt signaling. Surprisingly, anti-PD-1 therapy had no effect on increasing PGC1α or mitochondrial mass in CD8^+^ TIL. We then asked whether improving CD8^+^ TIL metabolism genetically might result in enhanced effector function, so we reprogrammed tumor-specific CD8^+^ T cells to upregulate mitochondrial biogenesis prior to adoptive cell therapy. We found increased mitochondrial mass, restored cytotoxic functionality, and dramatically improved tumor regression in mice with reprogrammed CD8^+^ TIL. To better understand why mitochondrial loss causes T cell dysfunction, we are exploring the importance of mitochondria for T cell functionality, including ATP and nucleotide production, calcium buffering, and ROS production.

**Conclusions**

Our data support a model in which chronically-activated CD8^+^ TIL are unable to metabolically support their effector functions. By understanding these metabolic insufficiencies, we can both better understand T cell dysfunction and design metabolic modulation strategies to improve cancer immunotherapy.

## Inflammation, Innate Immunity, and the Microbiome

### O13 Intestinal microbiota and relapse after hematopoietic-cell transplantation

#### Jonathan Peled, Sean Devlin, Anna Staffas, Melissa Lumish, Kori Porosnicu Rodriguez, Katya Ahr, Miguel Perales, Sergio Giralt, Ying Taur, Eric Pamer, Marcel R. M. van den Brink, Robert Jenq

##### Memorial Sloan Kettering Cancer Center, New York, NY, USA

###### **Correspondence:** Jonathan Peled (peledj@mskcc.org)

**Background**

The major causes of mortality after allogeneic hematopoietic-cell transplantation (allo-HCT) are relapse, graft-versus-host disease (GVHD), and infection. We have previously reported that alterations in the intestinal flora are associated with GVHD, bacteremia, and reduced overall survival after allo-HCT. As intestinal bacteria are potent modulators of systemic immune responses including antitumor effects triggered by checkpoint blockade, we hypothesized that components of the intestinal flora could be associated with relapse after allo-HCT.

**Methods**

**Fig. 13:**
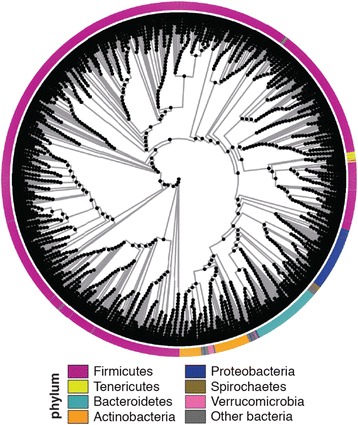
**(Abstract O13).** Phylogenetic tree of OTUs and clusters of related operational taxonomic units (crOTUs). Each black point is a crOTU. Phylum is color coded along the circumference. Members of the same phyla were largely grouped together, indicating that the tree was broadly concordant with standard taxonomy

**Fig. 14:**
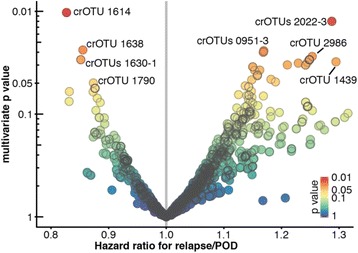
**(Abstract O13).** Multivariate screening of microbial features for association with relapse. Volcano plot of multivariate p values of crOTUs against the multivariate hazard ratios for relapse/progression of disease in the discovery set. crOTUs are color coded by p value. Multivariate adjustment was performed for Disease Risk Index score, graft source, and conditioning intensity. The most abundant species in each of the labeled crOTUs are 1614: Eubacterium limosum. 2022-3: Streptococcus sinensis. 1638: Eubacterium limosum. 1630-1: Eubacterium limosum. 1790: Parvimonas micra. 0951-3: Leptotrichia hongkongensis 2986: Flavonifractor plautii. 1439: Actinomyces odontolyticus

The intestinal microbiota of 541 patients admitted for allo-HCT was profiled by means of 16S ribosomal sequencing of prospectively collected stool samples. We hypothesized that evolutionarily related species exhibit functional similarities, and we therefore defined clusters of related operational taxonomic units (crOTUs) to evaluate for associations with clinical outcomes. To group OTUs by evolutionary distances, a phylogenetic tree was empirically constructed from a sequence alignment of all OTUs identified in the whole cohort (Fig. 13). We examined the relationship between abundance of microbiota species or groups of related species and relapse/progression of disease during two years of follow-up after allo-HCT using cause-specific Cox proportional hazards in a retrospective discovery-validation study (Fig. 14).

**Results**

**Fig. 15:**
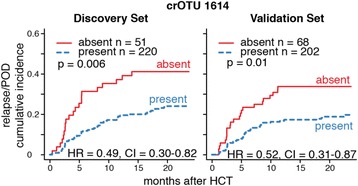
**(Abstract O13).** crOTU 1614, which includes members of family Eubacteriaceae is associated with decreased relapse after allo-HCT.Cumulative incidence of relapse/POD in the discovery (n = 271) and validation (n = 270) sets stratified by presence or absence of crOTU 1614

The intestinal presence of a group comprised mostly of *Eubacterium limosum* in the validation set was associated with less relapse/progression of disease (HR 0.52, CI 0.31–0.87, p = 0.01, Fig. 15). The two-year cumulative incidence of relapse/progression among patients with and without this group of bacteria was 33.8% and 19.8%, respectively. The relative abundance of this group was also associated with less relapse/progression of disease (HR 0.82, CI 0.71–0.95, p = 0.009). These associations remained significant in multivariate models and were strongest among recipients of T cell-replete allografts.

**Conclusions**

We found associations between the abundance of a group of bacteria in the intestinal flora and relapse/progression of disease after allo-HCT. These might serve as potential biomarkers or therapeutic targets to prevent relapse and improve survival after allo-HCT.

## Oncolytic Viruses

### O14 Phase I/II CANON study: oncolytic immunotherapy for the treatment of non-muscle invasive bladder (NMIBC) cancer using intravesical Coxsackievirus A21

#### Nicola Annels^1^, Hardev Pandha^1^, Guy Simpson^1^, Hugh Mostafid^2^, Kevin Harrington^3^, Alan Melcher^4^, Mark Grose^5^, Bronwyn Davies^5^, Gough Au^5^, Roberta Karpathy^5^, Darren Shafren^5^

##### ^1^University of Surrey, Guildford, England, UK; ^2^Royal Surrey County Hospital, Guildford, England, UK; ^3^Institute for Cancer Research, London, England, UK; ^4^The Institute for Cancer Research, London, England, UK; ^5^Viralytics, Inc., Sydney, New South Wales, Australia

###### **Correspondence:** Nicola Annels (n.annels@surrey.ac.uk)

**Background**

As a clinical setting in which local live biological therapy is already well established, non-muscle invasive bladder cancer (NMIBC) presents intriguing opportunities for oncolytic virotherapy. Coxsackievirus A21 (CVA21, CAVATAK^TM^) is a novel intercellular adhesion molecule-1 (ICAM-1)-targeted immunotherapeutic virus which exerts potent oncolytic activity against NMIBC cell lines and *ex-vivo* human bladder tumour. CVA21 in combination with low doses of Mitomycin C enhances CVA21 viral replication and oncolysis by increasing surface expression levels of ICAM-1.

**Methods**

A two stage Phase I/II study (CANON) was initiated to study the tolerance of escalating intravesicular (IV) doses of CVA21 administered alone or in combination with MitomycinC (10mg) in 16 first-line NMIBC cancer patients prior to TURBT surgery. Cystoscopy photography was performed before and after treatment. Tissues were analysed for CVA21 replication, apoptosis, changes in immune cell infiltrates (multi-spectral imaging) and immune-checkpoint molecules.

**Results**

IV administration of CAVATAK was well tolerated with no adverse events. Anti-cancer activity including viral induced tumour inflammation was demonstrated by serial cystoscopy including a complete response observed in one of 3 patients in the highest dose monotherapy cohort. Tumour targeting by CVA21 was shown by detection of secondary viral load peaks in the urine and by immunohistochemical analysis of TURBT tissue displaying tumour-specific viral replication and apoptotic cell death. Nanostring analysis revealed an upregulation of interferon-response and immune checkpoint inhibitory genes in CVA21-treated tissues compared to untreated historical controls. Notable changes in immune cell infiltrates and expression of PD-L1 within the CVA21-treated NMIBC tissue were also observed. Increased urinary levels of the chemokine, HMGB1, was observed in six of eleven patients following exposure to CVA21.

**Conclusions**

The utility of CVA21 as a potent immunotherapeutic agent has been demonstrated by the observed tumour targeting and viral replication. Upregulation of checkpoint molecules following CVA21 exposure may also allow potential sequential combination therapies with checkpoint targeting.

**Trial Registration**

ClinicalTrials.gov identifier NCT02316171.

### O15 Pre-existing immunity to oncolytic virus potentiates its therapeutic efficacy.

#### Jacob Ricca^1^, Taha Merghoub^2^, Jedd D Wolchok^3^, Dmitriy Zamarin^1^

##### ^1^Memorial Sloan Kettering Cancer Center, New York, NY, USA; ^2^Ludwig Collaborative Laboratory, Memorial Sloan Kettering Cancer Center, New York, NY, USA; ^3^Department of Medicine, Memorial Sloan Kettering Cancer Center, New York, NY, USA

###### **Correspondence:** Jacob Ricca (riccaj92@gmail.com)

**Background**

Despite the significant promise of oncolytic viral (OV) therapy in preclinical models, clinical efficacy of systemically-administered viruses has proven to be modest. One major limitation of the systemic OV therapy is neutralization of the virus by pre-existing immunity, or development of neutralizing antibodies shortly after therapy initiation, which limit viral delivery to tumor sites. Recently, we and others have demonstrated that intratumoral therapy with OV can lead to systemic anti-tumor immunity and abscopal effects, and several clinical trials are currently exploring intratumorally administered OVs in patients. The effect of pre-existing anti-viral immunity or the development of new anti-viral immunity on the anti-tumor efficacy, however, is not well defined.

**Methods**

Using oncolytic Newcastle Disease Virus (NDV) as a model, we explored the effect of pre-existing immunity to the virus on its therapeutic efficacy using syngeneic B16-F10 melanoma and MB49 bladder carcinoma models.

**Results**

BL6 mice were immunized with NDV and subsequently implanted with B16 or MB49 murine cancer cells. Immunized and naïve tumor-bearing mice were treated intratumorally with NDV. As expected, pre-immunized animals demonstrated decreased levels of NDV replication. Surprisingly, pre-existing immunity to the virus did not decrease the antitumor efficacy and led to superior tumor clearance and long-term animal survival. Analysis of tumor-infiltrating lymphocytes from the treated animals demonstrated marked increase in infiltration with CD8+ and CD4+FOXP3- cells, and significant decrease in CD4+FOXP3+ cells, an effect that was significantly more pronounced in the pre-immunized animals. This was observed in both virus-injected and contralateral flank tumors, in absence of viral spread to distant tumor sites. Concurrent adoptive transfer of luciferase-tagged tumor-specific Trp-1 lymphocytes demonstrated increased intratumoral accumulation of Trp-1 cells in pre-immunized mice. Furthermore, lymphocytes isolated from tumors of NDV-treated pre-immunized mice produced more IFNg than those of NDV-treated naïve mice when cultured with tumor cells *in vitro*, suggestive of antigen spreading. Finally, in an animal model of recurrent cancer after “cure” with NDV, re-treatment with NDV resulted in regression of tumors and long-term animal survival, an effect accompanied by significant increase in tumor-infiltrating immune cells.

**Conclusions**

Our findings demonstrate that pre-existing immunity to OVs might not deter, and even augment the efficacy of intratumoral OV therapy, which is likely mediated by enhanced tumor-specific immune response. This is a clinically-relevant question, which suggests that prior anti-viral immunity should not be a deterrent to OV therapy with locoregional administration, though it remains to be demonstrated whether such findings would translate to other oncolytic viruses.

## Promoting and Measuring Anti-Tumor Immunity

### O16 Immunoscore® Colon analytical performance

#### Luciana Batista^1^, Florence Marliot^2^, Angela Vasaturo^3^, Sabrina Carpentier^4^, Cécile Poggionovo^1^, Véronique Frayssinet^1^, Jacques Fieschi^1^, Marc Van den Eynde^5^, Franck Pagès^6^, Jérôme Galon^3^, Fabienne Hermitte^1^

##### ^1^HalioDx, Marseille, Provence-Alpes-Cote d'Azur, France; ^2^Université Paris Descartes, APHP, Paris, Ile-de-France, France; ^3^INSERM, Paris, Ile-de-France, France; ^4^MI-mAbs, Marseille, Provence-Alpes-Cote d'Azur, France; ^5^Université Catholique de Louvain, Brussels, Brussels Hoofdstedelijk Gewest, Belgium; ^6^APHP, Paris, Ile-de-France, France

###### **Correspondence:** Fabienne Hermitte (fabienne.hermitte@haliodx.com)

**Background**

The Immunoscore® was validated as a powerful prognostic marker in colon cancer in a study conducted by the Immunoscore® worldwide consortium, led by the Society for Immunotherapy of Cancer (SITC) involving 23 pathology centers from 17 countries, and including more than 3800 patients. HalioDx has developed a standardized version of the test that was used in this study. Here we show the concordance with the research version and present the main analytical performances of the system.

**Methods**

For each colon tumor block, 2 slides are stained using an automated IHC staining instrument: one with CD3 and one with CD8. Digital images of stained slides are obtained using a whole slide scanner, and analyzed by a software program (Immunoscore® Analyzer, HalioDx). The Immunoscore® Analyzer automatically processes images for tissue detection (core of the tumor, CT and invasive margin, IM). Densities of positive lymphocytes in the CT and IM are reported. For each marker and each zone, densities distributions have been established in the SITC study training set. The Immunoscore® is reported in 5 categories from 0 to 4, or as IS High (IS3 and IS4), Low (IS0 and IS1) & intermediate (IS2). Precision of HalioDx Immunoscore® Colon assay in terms of repeatability and reproducibility was evaluated using commercial FFPE colon cancer blocks, with 152 independent stainings from 4 samples, corresponding to 62 CD3 and CD8 pairs. Intra-block and inter-block variability were assessed from 8 additional blocks. Accuracy based on inter-laboratory concordance was determined using 119 samples. The European Hospital Georges Pompidou (HEGP - center of reference for the SITC study) workflow was used as reference.

**Results**

**Fig. 16:**
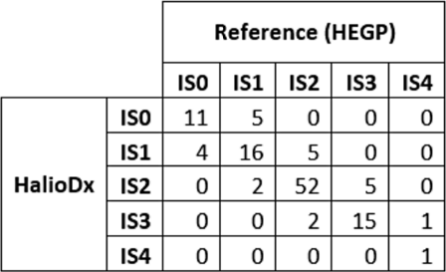
**(Abstract O16).**

The inter-instrument, inter-lot and inter-operator/-reader precision in terms of cell density (cells/mm^2^) CV were below 12%, 22% and 18%, respectively. Only 1 change in Immunoscore® category (out of 62 IS assessments) was observed, from IS1 to IS0. The equivalency between HalioDx and HEGP workflows was assessed in terms of cell densities. Deeming regression slopes were not significantly different from 1 for both CD3 and CD8 antibodies. Pearson correlation coefficients were above 0.89. The concordance table is provided in Fig. 16, corresponding to a weighted Cohen’s kappa coefficient of 0.88.

**Conclusions**

The Immunoscore® Colon is a robust, easy-to-use and accurate assay. It is the first standardized immune-based assay for the classification of cancer.

**References**

1. Galon J, *et al*: *J Pathol* 2014, **232(2)**:199-209.

2. Galon J, *et al*: *JCO* 2016, **34(15_suppl)**:3500.

### O17 Mechanisms of chitosan/IL-12 immunotherapy for the treatment of bladder cancer

#### Sean G. Smith^1^, Khue Nguyen^2^, Sruthi Ravindranathan^3^, Bhanu Koppolu^1^, David Zaharoff^1^

##### ^1^Joint Department of Biomedical Engineering, North Carolina State University and the University of North Carolina, Raleigh, NC, Cary, NC, USA; ^2^Cell and Molecular Biology, University of Arkansas, Fayetteville, AR, USA; ^3^Biomedical Engineering, University of Arkansas, Fayetteville, AR, USA

###### **Correspondence:** Sean G. Smith (sgseangr@ncsu.edu)

**Background**

Bladder cancer afflicts 430,000 people every year globally and is plagued by recurrence rates as high as 50%. One way to mitigate the risk of recurrence is by engaging adaptive immunity. Our group has been able to direct adaptive immunity via intravesical treatment with CS/IL-12, a coformulation of interleukin(IL)-12 and the biopolymer chitosan. Four twice-weekly administrations of CS/IL-12 routinely eliminate more than 90% of orthotopic bladder tumors in mice while providing systemic protection from recurrence and rechallenge for the duration of the lifespan of treated mice. The purpose of this study is to gain insights into the mechanisms underlying both the initial elimination and later rejection of bladder tumors by exploring the importance of the number of administrations, lymphocyte subtypes, and the immune cell infiltration throughout and following treatment.

**Methods**

Female C57BL/6J mice were implanted orthotopically with 75,000 MB49 bladder cancer cells. Beginning 7 days after implantation, mice were treated intravesically 2x/week for two weeks with CS/IL-12 (1 μg). The importance of the number of treatments was investigated by monitoring survival while varying the treatment number. The role of lymphocyte subtypes was investigated by monitoring survival after depleting CD4+, CD8+, or NK1.1+ cells prior to and throughout treatment or rechallenge. Cellular responses 24 hours after each treatment were measured in the bladder, bladder draining lymph nodes (BDLNs), and the spleen via flow cytometry.

**Results**

**Fig. 17:**
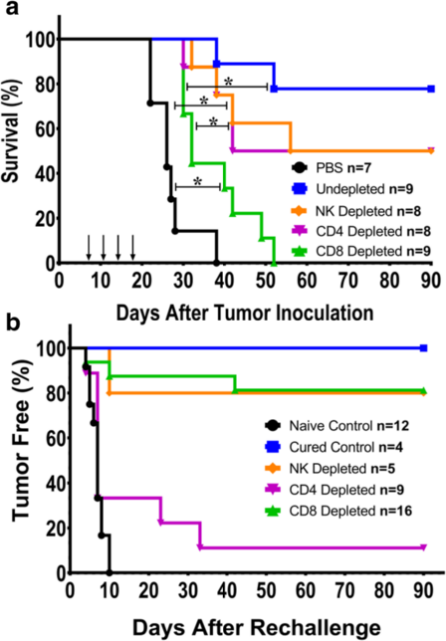
**(Abstract O17).** Depletion studies reveal role of T cell subtypes. Mice were depleted of NK1.1+, CD4+, or CD8+ cells either throughout treatment with CS/IL-12 (**a**) or rechallenge at a distant site (**b**). Significant differences (P<0.05) in median survival by the Log-Rank test are indicated by *

**Fig. 18:**
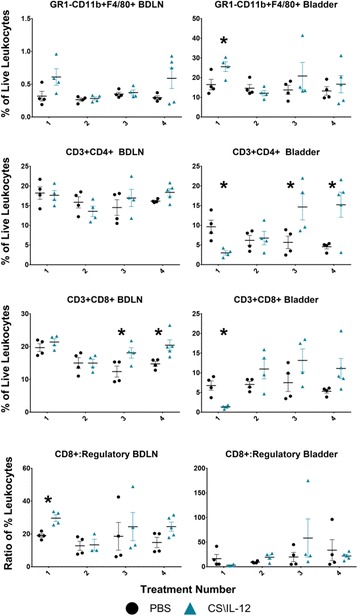
**(Abstract O17).** Immune infiltrates after each treatment. Bladder tumor tissues and bladder draining lymph nodes (BDLNs) were disassociated and analyzed via flow cytometry 24 hours after intravesical treatment with either PBS or CS/IL-12. Regulatory T cells were defined as CD3+CD4+CD25+FoxP3+. Each symbol represents an individual mouse. Significant differences (P<0.05) by T-tests are indicated by *

Varying the number of treatments revealed that a single administration significantly extended survival beyond saline with 4/10, 2/8, 6/9, and 7/8 mice surviving long term after 1, 2, 3, or 4 applications respectively. Depletion studies showed a dependence on CD8+ T cells for tumor elimination (Fig. 17a) and on CD4+ T cells for rejection of subsequent tumor rechallenge (Fig. 17b). Flow cytometry revealed fluctuations in the immune-cell populations over the course of treatment (Fig. 18). The first treatment was characterized by a 54% increase of macrophages in the bladder and a 56% increase in the CD8:Regulatory T cell ratio in the BDLNs. By the third treatment there was an influx of CD4+ and CD8+ T cells in the bladder as well as increased CD8+ T cells in the BDLNs.

**Conclusions**

Even a single administration of CS/IL-12 eliminates established tumors, though higher rates of survival were possible with 3 or 4 treatments. The initial response is inflammatory and driven by macrophage infiltration and CD8+ T cells while the memory responses is directed by CD4+ T cells.

## Survivorship Issues Related to Immunotherapy

### O18 Incidence and outcomes of central nervous system metastasis in metastatic melanoma patients treated with anti-PD-1 therapy

#### Gustavo Schvartsman, Roland Bassett, Jennifer L McQuade, Lauren E Haydu, Michael A Davies, Hussein Tawbi, Isabella Glitza

##### University of Texas MD Anderson Cancer Center, Houston, TX, USA

###### **Correspondence:** Gustavo Schvartsman (gschvartsman@mdanderson.org)

**Background**

Central nervous system (CNS) metastasis are common in patients with metastatic melanoma (MM) and represent a frequent site of treatment failure with current therapies. However, little is known about the incidence, characteristics and outcomes of CNS metastasis in MM patients treated with anti-programmed death-1 (PD-1) and in conjunction with more intensive local CNS treatment strategies.

**Methods**

Under an IRB-approved protocol, outcomes of MM patients treated with anti-PD-1 at The University of Texas MD Anderson Cancer Center from January 2012 to February 2016 were reviewed. The association between development of CNS metastasis and overall survival (OS) was assessed using Cox regression analysis with time to CNS metastasis treated as a time-varying covariate.

**Results**

We identified 264 MM patients who received anti-PD-1 treatment, including 74 (28%) who had CNS metastasis prior to the first dose of anti-PD-1. With a median follow-up of 10.4 months (range 0-51.6) from the start of this therapy, 37 (19% of patients without prior CNS metastasis) developed CNS metastasis after the initiation of anti-PD-1. Of those, 27 patients were diagnosed with CNS metastasis during anti-PD-1 or within 90 days of treatment discontinuation, and 10 patients were diagnosed with CNS mets >90 days after last anti-PD-1 dose. The majority of these patients were male (62%), their mean age at new CNS metastasis was 62 years (range 31-86), and most patients had a history of cutaneous primary (59%). Of the 26 patients who were tested for mutations, BRAF was identified in 8 (22%, V600E in 6 patients, V600K in 2 patients), NRAS in 5 (14%) and KIT in 6 (16%). 86% received at least one CNS directed treatment approach. 62% were treated with stereotactic radiosurgery, 11% received whole-brain radiation and 30% underwent surgery. Median OS from start of anti-PD-1 was 34 months (range 0-51.6 months) for the whole anti-PD-1 treatment cohort. Development of CNS metastasis while on anti-PD-1 therapy was strongly significantly associated with death (HR 3.39, 95% CI 2.06, 5.59, p < .0001).

**Conclusions**

To our knowledge, this is the first report describing the incidence of CNS metastasis as an initial site of disease progression in MM patients treated with anti-PD-1 and associated with worse OS, despite additional CNS directed therapy.

## Tumor Microenvironment

### O19 CD8α+ dendritic cells regulate leukemia antigen-specific CD8+ T cell tolerance

#### Douglas Kline^1^, Xiufen Chen^2^, Dominick Fosco^2^, Justin Kline^3^

##### ^1^Committee on Immunology, University of Chicago, Chicago, IL, USA; ^2^Department of Medicine, University of Chicago, Chicago, IL, USA; ^3^Committee on Immunology and Department of Medicine, University of Chicago, Chicago, IL, USA

###### **Correspondence:** Justin Kline (jkline@medicine.bsd.uchicago.edu)

**Background**

*Batf3*-lineage CD8α^+^ and CD103^+^ dendritic cells (DCs) are required for the spontaneous priming of CD8^+^ T cells against solid tumors. In contrast, the APCs that regulate immune responses against hematological malignancies have not been characterized. Syngeneic transplantable and genetically-engineered acute myeloid leukemia (AML) models associated with a dense CD8^+^ T cell tolerant state were employed to identify the APCs responsible for inducing T cell tolerance *in vivo*.

**Methods**

Transplantable C1498 and genetically-engineered *Mx1-Cre* x LSL^*AML1-ETO/+*^ x *FLT3* ^*ITD/ITD*^ *x R26-LSL* ^*SIY/+*^ (MAFFS) AML models were employed to characterize APCs involved in generating leukemia-specific CD8^+^ T cell tolerance.

**Results**

Following systemic introduction of viable, CellTrace violet-labeled AML cells, leukemia cell-derived fluorescence was observed exclusively within splenic CD8α^+^ DCs, whereas uptake of proteins from dead AML cells was mediated by CD11b^+^ macrophages. CD8α^+^ DCs were also uniquely capable of cross-presenting leukemia antigens to CD8^+^ T cells directly *ex vivo*. Interestingly, antigen encounter by leukemia-specific CD8^+^ T cells was severly reduced in *Batf3* ^*-/-*^ mice, indicating that CD8α^+^ DCs mediate T cell recognition of leukemia antigens, and that their absence is associated with immunological ignorance of AML antigens. Moreover, leukemia-specific CD8^+^ T cells in wildtype AML-bearing mice failed to respond following vaccination with the tolerizing antigen, while those in leukemia-bearing *Batf3* ^*-/-*^ mice expanded vigorously, demonstrating that CD8α^+^ DCs induce leukemia-specific tolerance *in vivo*. Activation of CD8α^+^ DCs with the TLR-3 agonist, poly(I:C) restored functional anti-leukemia T cell responses and protected mice from disease progression in a *Batf3*-dependent manner. RNA-seq analysis of "tolerogenic" versus "naive" CD8α^+^ DCs from leukemia-bearing mice revealed ~200 differentially expressed genes in the former, suggesting that tolerance induction by CD8α^+^ DCs is an active process.

**Conclusions**

Our data support a growing body of evidence that has defined a prominent role for *Batf3*-dependent DCs in regulating anti-cancer immune responses. *Batf3*-lineage DCs generate functional CD8^+^ T cell responses against solid tumors, but actively and exclusively induce CD8^+^ T cell tolerance to systemic leukemia, indicating that the same DC lineage can imprint disparate T cell fates in mice with solid verses hematopoietic malignancies, and suggesting that environmental cues perceived by CD8α^+^ DCs may dictate their ability to activate or tolerize cancer-specific CD8^+^ T cells. These results highlight stark differences in the regulation of anti-cancer immunity in hosts with solid versus hematological malignancies.

### O20 Neuropilin-1-deficient regulatory T cell-derived interferon-γ drives infectious instability and tumor clearance

#### Abigail Overacre^1^, Maria Chikina^1^, Erin Brunazzi^1^, Gulidanna Shayan^2^, William Horne^1^, Jay Kolls^1^, Robert L Ferris^1^, Greg M. Delgoffe^1^, Tullia C Bruno^3^, Creg Workman^1^, Dario Vignali^1^

##### ^1^University of Pittsburgh, Pittsburgh, PA, USA; ^2^Tsinghua University, Pittsburgh, PA, USA; ^3^University of Pittsburgh/Department of Immunology, Pittsburgh, PA, USA

###### **Correspondence:** Abigail Overacre (overacre@pitt.edu)

**Background**

Regulatory T cells (T_regs_) play an integral role in maintaining immune homeostasis; however, they are detrimental in cancer through suppression of the anti-tumor immune response. Therefore, identifying T_reg_ targets that are specifically required in the tumor microenvironment is warranted. We have previously shown that the Neuropilin-1 (Nrp1) pathway is required for functional stability of intratumoral T_regs_, but remains disposable in maintaining peripheral immune homeostasis. However, 1) the mechanisms that drive T_reg_ functional instability, 2) the fate and impact of functionally unstable Nrp1-deficient (Nrp1^–/–^) T_regs_ on the tumor microenvironment and 3) how NRP1 affects function in human T_regs_ remain unknown.

**Methods**

In order to further understand the role of Nrp1^–/–^ T_regs_ in cancer, we injected B16.F10 melanoma into *Nrp1* ^*L/L*^ *Foxp3* ^*Cre-YFP/DTR-GFP*^ cellular heterozygous mice comprised of 50% WT T_regs_ and 50% Nrp1^–/–^ T_regs_. Using these mice, we performed whole transcriptome sequencing to determine global transcriptomic changes. Once identified, differentially regulated pathways were tested both *ex vivo* through functional assays and *in vivo* with T_reg_ transfers into Foxp3^–/–^ mice. Lastly, we obtained head and neck squamous cell carcinoma and metastatic melanoma samples to determine the abundance and function of NRP1 on human T_regs_.

**Results**

Using *Nrp1* ^L/L^ *Foxp3* ^Cre-YFP/DTR-GFP^ mice, we found that intratumoral Nrp1^–/–^ T_regs_ produce interferon-γ (IFNγ), driving the functional destabilization of surrounding WT T_regs_, which in turn boosts antitumor immunity and facilitates tumor clearance. Furthermore, we have shown that NRP1 is expressed on a proportion of TIL T_regs_ in head and neck cancer as well as metastatic melanoma and that the IFNγ pathway is likely conserved in human T_regs_. In addition, human TIL T_regs_ pre-treated with IFNγ show significantly reduced suppressive function in comparison to those without pre-treatment.

**Conclusions**

Overall, we have shown that Nrp1 is required for functional stability of intratumoral T_regs_, and in its absence, there is an alteration in the tumor microenvironment, leading to an enhanced anti-tumor immune response. These studies uncover a novel potential target for cancer immunotherapies that preserves peripheral immune health. This is of clinical interest, given that NRP1 is expressed on select T_regs_ in human melanoma and head and neck cancer and that NRP1^+^ T_regs_ show a suppressive advantage over NRP1^–^ T_regs_.

## Adoptive Cellular Therapy

### P1 Converting tumor-mediated PD-L1 inhibition into CAR T cell costimulation to potentiate adoptive T cell therapy

#### Prasad S. Adusumilli

##### Memorial Sloan Kettering Cancer Center, New York, NY, USA

###### **Correspondence:** Prasad S Adusumilli (adusumip@mskcc.org)

**Background**

To overcome tumor-mediated inhibition of chimeric antigen receptor (CAR) T cells, we herein investigated the impact of tumor PD-L1 upregulation on CAR T cell exhaustion and anti-tumor efficacy, and further developed clinically translatable T cell-extrinsic as well as -intrinsic strategies to overcome PD-L1 inhibition in models of lung cancer (LC) and malignant pleural mesothelioma (MPM).

**Methods**

Human T cells were transduced with MSLN-specific CAR with CD28 and CD3zeta domains (M28z) were tested *in vitro* and in clinically-relevant LC and MPM mouse models by bioluminescence imaging (BLI) of tumor burden progression. To counteract PD-1/PD-L1 inhibition *in vivo*, we evaluated the efficacy of PD-1 blocking antibody or cell-intrinsic genetic-engineering strategies by cotransducing M28z CAR T cells with a PD-1 dominant negative receptor (PD1-DNR) or with PD-1/4-1BB fusion protein.

**Results**

A single, low-dose ofM28z CAR T cells is able to resist the progression of established tumor for 40 days, but mice eventually died with progressing tumor. Tumor harvest analysis demonstrated the PD-1 and PD-L1 upregulation on CAR T cells and tumor cells (Figure Panel A). We then confirmed *in vitro* that PD-L1 inhibits M28z T cell effector functions (proliferation, cytotoxicity and cytokine secretion). The addition of PD-1 blocking potentiates CAR T cell therapy *in vivo* but its efficacy requires multiple injections (Panel B). In contrast, a single dose of M28z T cells coexpressing PD-1-DNR restore effector functions, enhance tumor burden control (Panel C) and prolong median survival (56 vs 82 days, p=0.001). Converting PD-L1 inhibition into a positive costimulatory signal by PD-1/4-1BB construct cotransduction into M28z CAR T cells enhanced cytokine secretion and T cell accumulation (Panel D).

**Conclusions**

Our results demonstrate the therapeutic benefit of providing optimal costimulation and coinhibitory blockade to counteract PD-L1/PD-1 immunosuppression, thus potentiate CAR T cell therapy for lung cancer and mesothelioma.

**References**

1. Cherkassky L, Morello A, Villena-Vargas J, Feng Y, Dimitrov DS, Jones DR, *et al*: **CAR T cells with cell-intrinsic checkpoint blockade resist tumor-mediated inhibition**. *J Clin Invest* 2016, PMID: 27454297.

**Fig. 19:**
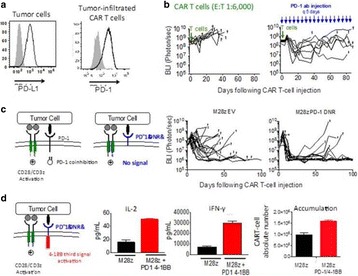
**(Abstract P1).**

### P2 Lack of moesin improves adoptive T cell therapy by potentiating anti-tumor functions

#### Ephraim A Ansa-Addo, Zihai Li

##### Medical University of South Carolina, Charleston, SC, USA

###### **Correspondence:** Ephraim A Ansa-Addo (ansaaddo@musc.edu)

**Background**

Moesin is a member of the ezrin-radixin-moesin (ERM) protein family that are crucial for organizing membrane domains [1]. However, the role of ERM proteins in regulating signal transduction activities is still less clear and identifying new target proteins regulated by the ERMs for drug targeting remains an important area within the field due to their increased levels in multiple cancers. Whether ERM proteins play any role during the differentiation of naïve CD4^+^ T cells to TGF-β-induced Tregs is completely unknown.

**Methods**

We utilized a combination of knockout (MsnKO) mice, polyribosome profiling, RT-PCR and immunoblotting to demonstrate that a lack of moesin promotes efficient adoptive T cell therapy in mice by controling translational upregulation of moesin by TGF-β in T cells.

**Results**

**Fig. 20:**
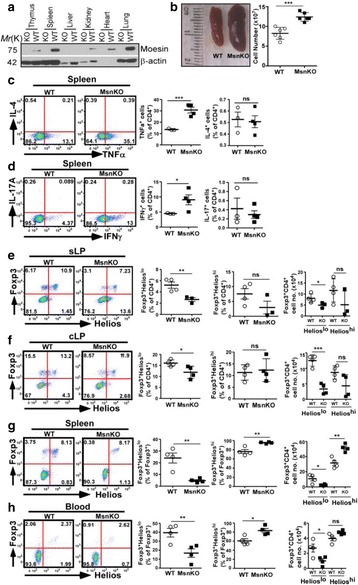
**(Abstract P2). a** Immunoblot of moesin knockout (KO or MsnKO) in multiple organs of mice. **b** Representative enlarged spleen size and increased cell number as observed in MsnKO mice. Data are reported as the mean ± SEM; ***P <0.001 by Student’s t-test. n = 6 per group. **c** and **d** Flow cytometry of inflammatory cytokines, TNF-α and IL-4 (**c**), and IFN-γ and IL-17A (**d**) produced by CD4+ T cells from spleens of WT and MsnKO mice after PMA-Ionomycin stimulation for 4 h. Data are reported as the mean ± SEM; *P <0.05 and ***P <0.001 by Student’s t-test. WT n = 3, MsnKO n = 4. **e** and **f** Flow cytometry analysis and absolute number of pTregs (Foxp3+Heliolo) in the small intestine lamina propria (sLP) (**e**) and colon lamina propria (cLP) (**f**) of 10-12 weeks old mice. Data are reported as the mean ± SEM; *P <0.05, **P<0.01 and ***P <0.001 by Student’s t-test. n = 4 per group, MsnKO sLP n = 3. **g** and **h** Flow cytometry analysis and absolute number of pTregs (Foxp3+Helioslo) in the spleen (**g**) and peripheral blood (**h**) of 10-12 weeks old mice. Data are reported as the mean ± SEM; ns, not significant; *P <0.05 and **P <0.01 by Student’s t-test. n = 4 per group

**Fig. 21:**
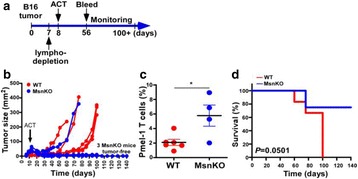
**(Abstract P2). a** Treatment scheme for B6 mice injected subcutaneously with B16-F1 melanoma tumor cells (2.5 x105) 7 days prior to lympho- 31 depletion with 6Gy total body irradiation and adoptive transfer (ACT) of 2 x106 Pmel-1 T cells (i.v.) at day 8. **b** Tumor growth kinetics in individual mice treated as indicated in A, (each line represents one mouse); WT (n=6), MsnKO (n=4). **c** Frequency of donor Pmel-1 CD8+ T cells circulating in the blood of WT and MsnKO mice from B at 8 weeks. **d** Survival analysis of WT and MsnKO mice upon B16 melanoma tumor injection and adoptive cell transfer. *P = 0.05 by Log-rank test (B and D), *P < 0.05 by Student’s t-test (C). Data are reported as the mean ± SEM

**Fig. 22:**
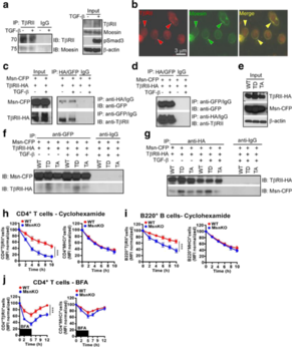
**(Abstract P2). a** Immunoprecipitation (IP), with anti-TβRII and control immunoglobulin G antibodies of EL4 cell lysates, followed by immunoblot of pulldown and input samples with the indicated antibodies. **b** Confocal microscopy images of EL4 cells stimulated with TGF-β (5 ng/ml for 1 h) and stained with anti-TβRII and anti-moesin antibodies. **c** and **d** HEK293FT cells co-transfected with plasmids encoding wild-type moesin-tagged with CFP at the carboxy terminus (Msn-WT-CFP) and TβRII-tagged with haemagglutinin at the carboxy terminus (TβRII-HA). Immunoprecipitation of solubilised proteins using anti-GFP and anti-HA antibodies and immunoblot of the pull-down samples. Input - whole cell lysate immunoblotting (throughout). **e**-**g** Immunoprecipitation and immunoblot (as in **c**) of HEK293FT cells co-transfected with CFP-tagged wild-type or phosphomimetic moesin mutants and TβRII-HA constructs. Data are representative of at least three (**a**-**c**) or four (**d**-**f**) independent experiments. **h** and **i** Primary CD4+ T cells (**h**) and B220+ B cells (**i**) from the spleen of WT and MsnKO mice were treated with cyclohexamide at the indicated times and surface TβRII analyzed by flow cytometry. Data represents the mean ± SD of at least three independent experiments. ***P <0.001 by two-way analysis of variance (ANOVA). **j** Flow cytometry analysis of primary CD4+ T cells isolated from the spleen of WT and MsnKO mice, and treated with brefeldin A (BFA), 20 μg/ml for up to 5 h and then washed. Cell surface TβRII was left to recover for up to 12 h prior to analysis. Data represents the mean ± SD of at least three independent experiments. ***P <0.001 by two-way analysis of variance (ANOVA)

**Fig. 23:**
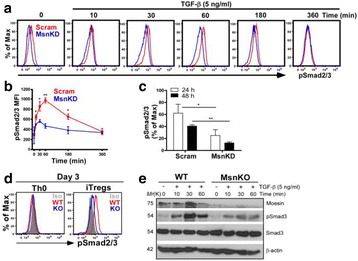
**(Abstract P2). a-c** EL4 LAF (EL4) cells transduced with lentiviral scrambled vector (Scram) or moesin shRNA (MsnKD), stimulated with TGF-β (5 ng/ml) and analyzed for intracellular pSmad2/3 by flow cytometry at the times indicated. Data are representative of the mean ± SD of at least three independent experiments. *P <0.05 and **P <0.01 by Student’s t-test. (**d** and **e**) Analyses of phospho-Smad2/3 (pSmad2/3) by flow cytometry after 3-day cultures (**d**) and pSmad3 and total Smad3 by immunoblotting (**e**) stimulated at the indicated times in WT and MsnKO iTregs. Data are representative of at least two independent experiments.

**Fig. 24:**
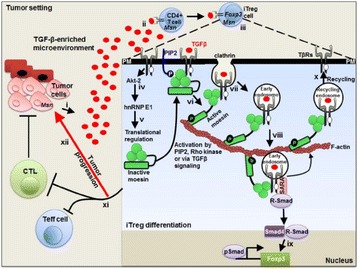
**(Abstract P2).** (i, ii) Under pathological conditions such as cancer, TGF-β production by tumor cells binds to cognate receptors on T cell surfaces and triggers signaling events that lead to Foxp3 expression and induced Treg cell development. (iii-iv) Signaling via the TGF-β non-canonical Akt-hnRNP E1-axis leads to post-transcriptional moesin expression. The TGF-β signaling pathway, Rho kinase and/or Phosphatidylinositol 4,5-bisphosphate[PtdIns(4,5)P2] pathways, lead to phosphorylation/activation of moesin and aids moesin binding to the F-actin. (v-viii) Moesin-F-acting binding may promote internalization of TGF-β receptors possibly via early endosomes which feeds forward to promote optimal TGF-β signaling leading to efficient Foxp3 induction and iTreg cell differentiation. (ix) Moesin may also promote efficient recycling of TGF-β receptors to maintain the abundance of TβRII on the cell surface. (x) Differentiated induced Treg cells then suppress the proliferation of other immune cells such as cytotoxic T lymphocytes and effector T (Teff) cells to limit anti-tumor responses and promote tumor progression

The lack of moesin led to poorer development and function of both peripherally-inducible Tregs and *in vitro*-induced Treg cells (Fig. 20). We found that the loss of moesin significantly delayed tumor recurrence in a mouse model of melanoma and supported the rapid expansion of adoptively transferred CD8 ^+^ T cells against cancer-associated antigens (Fig. 21). Of note, moesin knockout CD4^+^ T cells exhibited no defects in T cell receptor activation, proliferation or cytokine production, suggesting no alternations in T cell activation in these mice. Instead, our data indicate that moesin interacts with TGF-β receptor II and controls its surface abundance and stability (Fig. 22). Indeed, the lack of moesin significantly impaired optimal TGF-β signaling (Fig. 23) and improved adoptive T cell therapy under cancer setting (Fig. 24).

**Conclusions**

This finding is important and suggests that modulation of moesin (via inhibitors or agonists), such as developed recently for ezrin [2], could serve as a potential candidates for use in immunotherapy combinations for the treatment of cancer as well as advance our knowledge.

**Acknowledgements**

This work was supported by the US National Institutes of Health (R01DK098819 to D.C.R.; P01CA186866, R01CA188419 and R01AI070603 to Z.L.).

**References**

1. Hirata T, Nomachi A, Tohya K, Miyasaka M, Tsukita S, Watanabe T, Narumiya S: **Moesin-deficient mice reveal a non-redundant role for moesin in lymphocyte homeostasis**. *Int Immunol* 2012, **24(11)**:705-717.

2. Celik H, Bulut G, Han J, Graham GT, Minas TZ, Conn EJ, Hong SH, Pauly GT, Hayran M, Li X, Ozdemirli M, Ayhan A, Rudek MA, Toretsky JA, Uren A: **Ezrin inhibition up-regulates stress response gene expression**. *J Biol Chem* 2016, **291(25)**:13257-13270.

### P3 Preclinical evaluation of an optimal-affinity MAGE-A4 T cell receptor for adoptive T cell therapy

#### Andrew Gerry, Joseph P Sanderson, Karen Howe, Roslin Docta, Qian Gao, Eleanor A L Bagg, Nicholas Tribble, Miguel Maroto

##### Adaptimmune, Oxfordshire, England, UK

###### **Correspondence:** Andrew Gerry (andrew.gerry@adaptimmune.com)

**Background**

Adoptive immunotherapy employing optimal affinity T cell receptor (TCR) engineered T cells is a highly attractive treatment modality for multiple cancer indications. In order to ensure the safety of novel T cell receptor therapies, it is important both that expression of the target antigen is tightly restricted to tumor, and that the TCR does not display off-target activity. Here we describe development of an optimal-affinity MAGE-A4 TCR for adoptive T cell therapy.

**Methods**

Expression profiling of the cancer-germline antigen MAGE-A4 was performed in tumor and normal tissues, determined by analyzing public RNAseq datasets and by in-house qPCR. We then generated an enhanced affinity TCR that recognizes a validated MAGE-A4 HLA-A*02 peptide, selected based on potency and specificity in *in vitro* testing from panels of engineered TCRs originating from multiple parental TCRs. The selected TCR was subject to full preclinical characterization using Adaptimmune’s extensive preclinical testing process. This process involves potency testing against both tumor cell lines and primary tumor tissue in 2D and 3D, and safety testing consisting of extensive screening of TCR-transduced T cell responses to a wide range of tumor lines, normal human primary cells and induced pluripotent stem cell-derived cells. In addition, the fine specificity of the TCR was characterized to allow the generation of a binding motif and the identification of putative mimotype peptides within the human proteome.

**Results**

The MAGE-A4 antigen was found to be highly over-expressed in several clinically important solid tumor indications, such as lung squamous cell cancer (60%), head and neck cancer (42%), bladder cancer (34%) and esophageal cancer (33%), while expression in non-tumor material was limited to expression in the testes and placenta, both immune-privileged tissues. We generated an enhanced-affinity TCR that demonstrated enhanced potency against MAGE-A4-expressing tumor cell lines and fresh tumor tissue, whilst retaining absence of relevant response against MAGE-A4-negative cells and non-MAGE peptide mimotypes.

**Conclusions**

MAGE-A4 is an attractive target antigen for adoptive T cell therapy using enhanced affinity TCRs. We have generated and characterized an optimal enhanced-affinity TCR, which shows enhanced potency against MAGE-A4-positive tumor targets whilst maintaining specificity. These data will be used to support an IND for the use of this TCR for investigatory clinical trials.

### P4 Case report: specific peptide enhanced affinity receptor T cells (SPEAR® T cells) demonstrate long-term persistence and both in vivo and ex vivo tumoricidal activity

#### Gareth Betts^1^, Natalie Bath^1^, Luca Melchiori^1^, Daniel E Lowther^1^, Indu Ramachandran^1^, Gabor Kari^1^, Samik Basu^1^, Gwendolyn Binder-Scholl^1^, Karen Chagin^1^, Lini Pandite^1^, Tom Holdich^1^, Rafael Amado^1^, Hua Zhang^2^, John Glod^2^, Donna Bernstein^2^, Bent Jakobsen^3^, Crystal Mackall^4^

##### ^1^Adaptimmune, Philadelphia, PA, USA; ^2^National Cancer Institute, Bethesda, MD, USA; ^3^Adaptimmune, Oxfordshire, England, UK; ^4^Stanford University School of Medicine, Stanford, CA, USA

###### **Correspondence:** Samik Basu (samik.basu@adaptimmune.com)

**Background**

SPEAR® T cells reactive against the NY-ESO-1 specific HLA-A02:01 restricted peptide (SLLMWITQC) have demonstrated clinical activity (ORR 50%) in patients (n=12) with advanced synovial sarcoma (SS). The mechanisms underlying tumor relapse in the presence of persisting SPEAR® T cells remain unclear. Here, we report on phenotypic and functional studies on both engineered T cells and tumor biopsies from a patient with a NY-ESO-1^+^ SS treated with NY-ESO-1^C259^ SPEAR® T cells.

**Methods**

Engineered T cell persistence was determined by qPCR for the vector backbone and flow cytometry for HLA-A2:01-SLLMWITQC reactive pentamer^+^ T cells in post-infusion PBMC samples. Multi-parameter flow cytometric analyses were performed on pre-infusion manufactured product and post-infusion PBMCs to assess memory subsets using CD45RA and CCR7, exhaustion using CD28 and PD-1, and functionality by IFN-ɣ and Gzmb. Tumor and NY-ESO-1^C259^ T cells from patient PBMCs were isolated at 28 months post-infusion to determine their *ex vivo* killing capacity against a NY-ESO-1^+^ cell line, A375 . Antigen expression and immunomodulatory milieu (e.g. PD-L1) in baseline and post-treatment biopsies were assessed by immunohistochemistry. Serum cytokines were measured by a Luminex based immunoassay. Tumor response was determined by RECIST v1.1.

**Results**

The patient achieved a partial response to NY-ESO-1^C259^ SPEAR® T cells with progression at 9 months post-infusion. Persistence at 28 months with NY-ESO-1^C259^ T cells was observed by qPCR and flow cytometry. Over the course of treatment, the phenotype of the engineered cells changed from a mix of T_EMRA_ (CD45RA^+^CCR7^-^), T_EM_ (CD45RA^-^CCR7^-^), and T_SCM_ (CD45RA^+^CCR7^+^) populations at the time of infusion to a predominately T_SCM_ (~98.7%) within five months. PBMC derived NY-ESO-1^C259^ SPEAR® T cells 28 months post-infusion exhibited substantial *ex vivo* killing of NY-ESO-1^+^ A375 cells without additional *ex vivo* re-stimulation. Pre- and post-infusion biopsies showed NY-ESO-1 expression and exhibited minimal to moderate leukocytic (CD45^+^) infiltration accompanied by minimal lymphocytic infiltration post-infusion. Of note, PD-L1 expression was exclusive to CD45^+^ cells.

**Conclusions**

Despite an initial response to NY-ESO-1^C259^ SPEAR® T cells, this patient eventually relapsed despite the persistence of functional SPEAR® T cells and antigen positive tumor . The basis for tumor progression following response remains unclear, but does not appear to result from T cell exhaustion. Other possibilities include loss of antigen expression and/or diminished tumor infiltration, which could result from the large peripheral T_SCM_ population, known to traffic to lymphoid tissue rather than tumor.

**Trial Registration**

ClinicalTrials.gov identifier NCT01343043.

**Consent**

Written informed consent was obtained from patient for publication. A copy is available for editor review.

### P5 Engineering 2nd generation SPEAR® T cells to overcome TGF-β-mediated immunosuppression for adoptive cell therapy

#### Ryan Wong^1^, Jonathan D Silk^1^, Katherine Adams^1^, Garth Hamilton^1^, Alan D Bennett^1^, Sara Brett^2^, Junping Jing^2^, Adriano Quattrini^1^, Manoj Saini^1^, Guy Wiedermann^1^, Andrew Gerry^1^, Bent Jakobsen^1^, Gwendolyn Binder-Scholl^3^, Joanna Brewer^1^

##### ^1^Adaptimmune, Oxfordshire, England, UK; ^2^GSK, Stevenage, England, UK; ^3^Adaptimmune, Philadelphia, PA, USA

###### **Correspondence:** Andrew Gerry (andrew.gerry@adaptimmune.com)

**Background**

Adoptive cell therapy (ACT) with NY-ESO SPEAR® T cells, is showing promising initial clinical responses in phase I/II trials for both solid and liquid tumors including synovial sarcoma and multiple myeloma. However, the depth and durability of response may be affected by the inhibitory tumor microenvironment. Tumors utilize many different methods to inhibit anti-tumor immunity including secretion of inhibitory cytokines, such as transforming growth factor-β (TGF-β) and induction/recruitment of other inhibitory cells including regulatory T cells and myeloid-derived suppressor cells. These inhibitory cells also secrete cytokines such as IL-10 and TGF-β that potentially reduce the efficacy of T cells. TGF-β is expressed at high levels in a range of cancer indications.

**Methods**

We investigated whether SPEAR® T cells can be engineered to express additional proteins, allowing them to overcome such immune resistance mechanisms, potentially improving clinical responses. TGF-β inhibits T cells by binding to a dimer of TGFβRII, which then recruits a dimer of TGFβRI forming a heterotetrameric complex that activates inhibitory intracellular SMAD signaling pathways. Truncating the intracellular signaling domain produces a dominant negative TGF-β receptor (dnTGFβRII) that, although capable of binding TGF-β, is unable to signal. We therefore generated SPEAR® T cells co-expressing enhanced affinity T cell receptors (TCR) that recognize a peptide from NY-ESO/LAGE-1A in the context of HLA-A2, together with dnTGFβRII and tested their function *in vitro*.

**Results**

Firstly we showed that the function of NY-ESO SPEAR® T cells is inhibited with physiologically-relevant concentrations of TGF-β. We further show that dnTGFbRII can be co-expressed with enhanced affinity NY-ESO TCR in SPEAR® T cells. T cells expressing dnTGFβRII had reduced SMAD phosphorylation in response to TGF-β compared with cells expressing TCR alone, indicating that inhibitory signaling in response to TGF-β was reduced. Subsequently we showed that T cells expressing dnTGFβRII were partially or completely resistant to the effects of TGF-β, using assays for T cell proliferation, cytotoxicity (in 2D and with 3D microtissue models) and Th1 cytokine release (IFN-γ and IL-2) in response to antigen positive tumor cells.

**Conclusions**

Together these data indicate that co-expression of dnTGFβRII may be a viable approach to improve the efficacy of SPEAR® T cells in treating cancer.

### P6 Inducible MyD88/CD40 (iMC) costimulation drives ligand-dependent tumor eradication by CD123-specific chimeric antigen receptor T cells

#### MyLinh Duong^1^, An Lu^1^, Peter Chang^1^, Aruna Mahendravada^1^, Nicholas Shinners^1^, Kevin Slawin^1^, David M Spencer^2^, Aaron E Foster^1^, J Henri Bayle^1^

##### ^1^Bellicum Pharmaceuticals, Houston, TX, USA; ^2^Bellicum Pharmaceuticals and Baylor College of Medicine, Houston, TX, USA

###### **Correspondence:** J. Henri Bayle (jhbayle@bellicum.com)

**Background**

CD123/IL-3Rα is a promising chimeric antigen receptor (CAR)-T cell target due to its high expression on both acute myeloid leukemia (AML) blasts and leukemic stem cells (AML-LSCs). However, the antigen is also expressed at lower levels on normal stem cell progenitors, presenting a major toxicity concern should CD123-specific CAR-T cells show long-term persistence. Here, we describe a CAR platform, “GoCAR-T”, that uses a proliferation-deficient, first generation, CD123-specific CAR together with a ligand-dependent costimulatory switch (inducible MyD88/CD40 (iMC)) to provide physician-controlled eradication of CD123 ^+^ tumor cells and regulate long-term CAR-T cell engraftment.

**Methods**

T cells were activated and transduced with a bicistronic retrovirus encoding iMC (MyD88 and CD40 cytoplasmic signaling domains fused with tandem copies of FKBPv36 (binding domain for the dimerizing ligand rimiducid (Rim)) and a first generation CAR targeting CD123 (SFG--iMC-CD123.ζ). Ligand dependence for costimulation with iMC was assessed in coculture assays with CD123^+^ AML cell lines (KG1, THP-1 and MOLM-13) by examination of cytokine production and observation by IncuCyte-based live cell imaging. *In vivo* efficacy was assessed by i.v. injection of 10^6^ EGFP*luc*-expressing CD123^+^ THP-1 tumor cells into immunodeficient NSG mice. After seven days 2.5x10^6^ non-transduced or iMC-CD123.ζ-modified T cells were injected and Rim (1 mg/kg) or vehicle only administered i.p. on days 0 and 15 post-T cell injection. Animals were evaluated for tumor burden using IVIS bioluminescent imaging (BLI) and for T cell persistence by flow cytometry and qPCR at day 30 post-T cell injection.

**Results**

In coculture assays, both CD123 antigen recognition and Rim-dependent iMC costimulation were required for IL-2 production (285±41 versus 2,541±255 pg/ml for control and 1 nM Rim, respectively), robust CAR-T cell proliferation (87-fold increase with Rim stimulation) and enhanced KG1 cell killing. In NSG mice engrafted with CD123^+^ THP-1-EGFP*luc* tumor cells, only animals treated with iMC-CD123.ζ-modified T cells and Rim controlled tumor growth, showing a 2-log reduction in tumor burden with Rim treatment. Two weeks after the second Rim injection, CAR-T cells were infrequent (<1.0%) in the spleen and bone marrow of both CAR groups, suggesting that active costimulation is required for CAR-T persistence.

**Conclusions**

GoCAR-T, a platform comprising a ligand-dependent activation switch and a proliferation-deficient first generation CAR, efficiently eradicates CD123^+^ leukemic cells when costimulation is provided by systemic rimiducid administration. Deprivation of iMC costimulation results in reduction of CAR-T levels, providing a user-controlled system for managing persistence and safety of CD123-specific CAR-T cells.

**Fig. 25:**
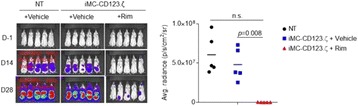
**(Abstract P6).** Rimiducid-dependent MyD88/CD40 costimulation enhances antitumor activity of a first-generation CD123-specific CAR

### P7 Heterodimeric IL-15 treatment enhances tumor infiltration, persistence and effector functions of adoptively transferred tumor-specific T cells in the absence of lymphodepletion

#### Cristina Bergamaschi^1^, Sinnie Sin Man Ng^1^, Bethany Nagy^1^, Shawn Jensen^2^, Xintao Hu^1^, Candido Alicea^1^, Bernard Fox^2^, Barbara Felber^1^, George Pavlakis^1^

##### ^1^National Cancer Institute at Frederick, Frederick, MD, USA; ^2^Providence Cancer Center, Portland, OR, USA

###### **Correspondence:** Cristina Bergamaschi (cristina.bergamaschi@nih.gov)

**Background**

Adoptive cell transfer (ACT) is a promising immunotherapeutic approach for cancer. Host lymphodepletion is associated with favorable ACT therapy outcomes, but it may cause detrimental effects in humans. Among the benefits provided by lymphodepletion, ablation of cells forming a cytokine “sink” results in high levels of homeostatic cytokines that support proliferation and survival of the transferred lymphocytes. Interleukin-15 (IL-15) is a lymphocyte growth and activation factor presently in clinical trials for immunotherapy of metastatic cancers. We previously showed that bioactive IL-15 *in vivo* comprises a stable complex of the IL-15 chain with the IL-15 receptor alpha chain (IL-15Rα), termed heterodimeric IL-15 (hetIL-15). In this study, we tested the hypothesis that hetIL-15 administration enhances ACT in the absence of lymphodepletion.

**Methods**

We evaluated the effects of the combination regimen ACT+hetIL-15 in the absence of lymphodepletion by transferring melanoma-specific Pmel-1 T cells into B16 melanoma-bearing mice. Tumors were analyzed by both flow cytometry and multi-parameter immunohistochemistry. Tumor-infiltrating transferred Pmel-1 were analyzed for their persistence, proliferation and effector functions.

**Results**

hetIL-15 treatment delayed tumor growth by promoting infiltration and persistence of both adoptively transferred Pmel-1 cells and endogenous CD8^+^ T cells into the tumor. In contrast, persistence of Pmel-1 cells was severely reduced following irradiation in comparison to hetIL-15 treatment. Importantly, we found that hetIL-15 led to the preferential enrichment of Pmel-1 cells in B16 tumor sites in an antigen-dependent manner. Upon hetIL-15 administration, tumor-infiltrating Pmel-1 cells showed a “non-exhausted” effector phenotype, characterized by increased IFN-g secretion, proliferation and cytotoxic potential and low level of PD-1. hetIL-15 treatment also resulted in an improved Pmel-1 to Treg ratio in the tumor.

**Conclusions**

This study shows that hetIL-15 administration improves the outcome of ACT in immunocompetent hosts and is able to replace the need for lymphodepletion prior ACT for cancer therapy. Applications of heterodimeric IL-15 to ACT will provide new tools and techniques for cancer immunotherapy protocols. Elimination of the need for lymphodepletion will make more patients eligible for cell transfer protocols. In addition, IL-15 could be a general method to place T cells into tumors, increasing the success rate of other immunotherapy interventions.

### P8 Withdrawn

### P9 Partially differentiated polyfunctional T cells dominate the periphery after tumor-infiltrating lymphocytes therapy for cancer

#### Marco Donia^1^, Julie Westerlin Kjeldsen^2^, Rikke Andersen^1^, Marie Christine Wulff Westergaard^1^, Valentina Bianchi^3^, Mateusz Legut^3^, Meriem Attaf^3^, Garry Dolton^3^, Barbara Szomolay^4^, Sascha Ott^5^, Rikke Lyngaa^6^, Sine Reker Hadrup^6^, Andrew Kelvin Sewell^3^, Inge Marie Svane^1^

##### ^1^Department of Oncology, Center for Cancer Immune Therapy, Herlev Hospital, Herlev, Hovedstaden, Denmark; ^2^Center for Cancer Immune Therapy, Herlev Hospital, Herlev, Hovedstaden, Denmark; ^3^Cardiff University School of Medicine, Cardiff, Wales, UK; ^4^Systems Immunology Institute, Cardiff University, Cardiff, Wales, UK; ^5^Warwick Systems Biology Centre, University of Warwick, Coventry, England, UK; ^6^Section for Immunology and Vaccinology, National Veterinary Institute, Technical University of Denmark, Frederiksberg, Hovedstaden, Denmark

###### **Correspondence:** Marco Donia (doniamarco@gmail.com)

**Background**

Infusion of highly heterogeneous populations of autologous tumor-infiltrating lymphocytes (TILs) can result in tumor regression of exceptional duration. Initial tumor regression has been associated with persistence of tumor-specific TILs one month after infusion, but mechanisms leading to long-lived memory responses are currently unknown. Here, we studied the dynamics of bulk tumor-reactive CD8^+^ T cell populations in patients with metastatic melanoma following treatment with TILs.

**Methods**

We analyzed the function and phenotype of tumor-reactive CD8^+^ T cells contained in serial blood samples of sixteen patients treated with TILs.

**Results**

Polyfunctional tumor-reactive CD8^+^ T cells accumulated over time in the peripheral lymphocyte pool. Combinatorial analysis of multiple surface markers (CD57, CD27, CD45RO, PD-1 and LAG-3) showed a unique differentiation pattern of polyfunctional tumor-reactive CD8^+^ T cells. This subpopulation acquired simultaneously expression of the early differentiation marker CD27, alongside typical features of late effector cells such as loss of CD45RO and up-regulation of PD-1 and CD57. The differentiation and functional status appeared very similar from 1 month to 1 year after infusion. Despite some degree of clonal diversification occuring *in vivo* within the bulk tumor-reactive CD8^+^ T cells, further analyses showed that CD8^+^ T cells specific for defined tumor-antigens had similar differentiation status.

**Conclusions**

We demonstrated that tumor-reactive CD8^+^ T cell subsets that persist after TIL therapy are mostly polyfunctional,and display a stable partially differentiated phenotype. These atypical CD27^+^ incompletely differentiated polyfunctional TILs may have a high capacity for persistence and be crucial in keeping patients tumor free.

**Trial Registration**

ClinicalTrials.gov identifier NCT00937625.

### P10 Peptide vaccine enhances antitumor effect of adoptive cell transfer using genetically engineered T cells

#### Aaron Fan^1^, Takumi Kumai^2^, Esteban Celis^2^

##### ^1^Augusta University, Augusta, GA, USA; ^2^Georgia Cancer Center, Augusta University, Augusta, GA, USA

###### **Correspondence:** Aaron Fan (afan@augusta.edu)

**Background**

Adoptive cell therapy (ACT) has shown promise in tumor eradication in cancer, but current methods require harmful and toxic adjunct procedures. Our laboratory has developed a potent peptide vaccination strategy called TriVax that bypasses the necessity of these adjunct procedures. Previous studies show that retrovirally (RV)-transduced T cells are effective in ACT against various cancers. The present study aimed to determine whether RV-transduced T cells could respond to TriVax specific for melanosomal tumor antigen gp100, and to see if the responses could be enhanced when transduced with a constitutively active form of STAT5 (CA-STAT5), which has been shown to increase survival of CD8 effector/effector memory T cell populations.

**Methods**

CD8 T cells were purified from B6 mouse splenocytes and RV-transduced with a gene encoding the clonal T cell receptor (TCRs) for mouse gp100. In some experiments, cells were also co-transduced with a gene encoding for CA-STAT5 (co-expressing Thy1.1). Transduction efficiency was assessed using flow cytometry with gp100 tetramer and Thy1.1 antibody. Functional activity was measured using flow cytometry and EliSpot (IFNγ) assays. These cells were then adoptively transferred (1.0x10^5^ tetramer+ cells) into naïve and tumor bearing congenic CD45.1 B6 mice, which were then vaccinated with TriVax. Tumor growth was assessed 3 times per week, and immune status was assessed in blood using flow cytometry every 7 days.

**Results**

TriVax administration selectively expanded the ACT cell population expressing gp100-TCR. Cell numbers in spleen indicate a 14-fold expansion 25 days after vaccination from what was initially transferred. When co-transduced with CA-STAT5, an even higher fold expansion (55-fold) was observed. CA-STAT5+ cells also expanded more robustly than CA-STAT5- cells when stimulated with a subsequent vaccine boost, demonstrating a 5000-fold increase in cell numbers with 85% of CD8+ T cells also being positive for gp100 tetramer. ACT of these cell populations into tumor bearing mice also yielded a dramatic increase in cell numbers, which greatly enhanced the survival of mice in treatment groups.

**Conclusions**

CD8 T cells RV-transduced to express a gp100 TCR and CA-STAT5 are capable of expansion in response to TriVax, bypassing the necessity of adjunct procedures. Co-expression of CA-STAT5 greatly enhances the boost effect of TriVax, leading to a dramatic anti-tumor effect.

### P11 Stable tumor-infiltrating lymphocytes (TIL) phenotype following cryopreservation

#### Ian Frank, Amanda Stramer, Michelle A Blaskovich, Seth Wardell, Maria Fardis, James Bender, Michael T Lotze

##### Lion Biotechnologies, Inc., Tampa, FL, USA

###### **Correspondence:** Ian Frank (Ian.frank@lionbio.com)

**Background**

Lion Biotechnologies focuses on the development and commercialization of cancer immunotherapies based on tumor-infiltrating lymphocytes (TIL). Cryopreservation is a beneficial process which allows the final cell product to be shipped in a safe manner with less time constraints [1]. Clinical studies using cryopreserved TIL have not been conducted so far. Freezing and thawing of the cells may cause phenotypic changes such as loss of cell surface receptors [2]. Here, we tested fresh versus frozen/thawed TIL samples and evaluated the expression of phenotypic markers.

**Methods**

Briefly, PreREP TILs were obtained by culturing melanoma tumor fragments in IL-2 (6000 IU/ml). Rapid expansion protocol (REP) cells were initiated using allogeneic PBMC feeder cells with OKT3 and IL-2 in a Grex-100 flask. When the desired confluency was reached, the cells were cryopreserved in a 5% DMSO solution. We used flow cytometry to phenotype the fresh and thaw/rested TIL at 0h, 24h and 7d following reREP TIL. Flow cytometric analysis was performed using fluorescent antibodies specific for TCRa/b, CD4, CD8, CD27, CD28, CD56, CCR7, CD45RA, CD95, PD-1, and CD25.

**Results**

No significant differences in CD4, CD8 and TCRa/b expression or memory markers comparing fresh TIL versus thaw/rested TIL at 24h was noted. CD27 expression on TIL was reduced by 50% on thawed TIL, however after a 24h resting period the expression recovered. All other surface antigens that we tested were within 10% difference in their expression levels as compared to baseline.

**Conclusions**

Cryopreservation did not affect the measured phenotypic characteristics of TIL. We are further investigating the possibility of using cryopreserved TIL in a clinical setting.

**References**

1. Axelsson S, Faresjo M, Hedman M, Ludvigsson J, Casas R: **Cryopreserved peripheral blood mononuclear cells are suitable for the assessment of immunological markers in type 1 diabetic children.** *Cryobiology* 2008, **57**:201–208.

2. Sadeghi A, Ullenhag G, Wagenius G, Tötterman TH, Eriksson F: **Rapid expansion of T cells: Effects of culture and cryopreservation and importance of short-term cell recovery**. *Acta Oncol* 2013, **52**:978-986.

### P12 Recognition of autologous neoantigens by tumor infiltrating lymphocytes derived from human breast cancer metastases

#### Stephanie L. Goff^1^, Nikolaos Zacharakis^1^, Yasmine Assadipour^1^, Todd D Prickett^1^, Jared J Gartner^1^, Robert Somerville^2^, Mary Black^1^, Hui Xu^1^, Harshini Chinnasamy^1^, Isaac Kriley^1^, Lily Lu^1^, John Wunderlich^1^, Paul F Robbins^1^, Steven Rosenberg^1^, Steven A Feldman^1^

##### ^1^Surgery Branch, National Cancer Institute, National Institutes of Health, Bethesda, MD, USA; ^2^National Cancer Institute, Bethesda, MD, USA

###### **Correspondence:** Stephanie L. Goff (stephanie.goff@nih.gov)

**Background**

Adoptive transfer of tumor infiltrating lymphocytes (TIL) can effect long-term durable regression in patients with metastatic melanoma but has not been widely tested in common epithelial cancers. When examining the TIL of successfully treated patients with melanoma, a heterogeneous T cell population can be identified with reactivity against melanoma differentiation antigens, cancer germline antigens, and personalized non-synonymous somatic mutations. Common epithelial cancers, including breast cancer, express far fewer somatic mutations than melanoma, however, in a study of metastatic gastrointestinal cancer, lymphocytes targeting neoantigens were identified in the majority of specimens. This pilot study investigates the ability to grow TIL from breast cancer metastases, to identify personalized non-synonymous somatic mutations and potential neoantigens, and to adoptively transfer TIL into patients with breast cancer.

**Methods**

Eligible patients were evaluated and treated under IRB-approved protocols for tissue procurement, genomic testing, and adoptive cell transfer. Portions of resected tumors were placed in culture under standard TIL conditions. DNA was extracted from tumor and matched normal peripheral blood samples for whole exome sequencing (WES). Non-synonymous somatic mutations were identified and tested for potential immunogenicity using previously described tandem mini-gene and long (25mer) peptide techniques. Recognition was assessed by IFNγ release on ELISPOT and/or CD137 (4-1BB) upregulation with appropriate controls.

**Results**

Nine patients underwent surgical resection in this ongoing pilot study, and TIL were successfully grown from the tumors of all patients. All were primarily CD3+ (median 79%) with a small population of natural killer cells. Of the CD3+ cells, 7 of 9 patients had a predominantly CD4+ population (median CD4:CD8 ratio 2.2, range 0.4-5.8). For eight tumors, WES was performed, and non-synonymous somatic mutations were identified as potential neoantigens (median count 96.5, range 71-148). Autologous T cell reactivity has been identified against tumor-specific mutations in 6 of 8 patients.

**Conclusions**

Tumor-infiltrating lymphocytes derived from metastatic breast cancer can react to tumor-specific non-synonymous somatic mutations *in vitro*. TIL grown from breast cancers are predominantly CD4+ and can form the basis of an adoptive cell transfer experimental approach to patients with metastatic breast cancer.

**Trial Registration**

ClinicalTrials.gov identifier NCT01174121.

### P13 Comparison of RECIST 1.0 to RECIST 1.1 in the evaluation of adoptive cell transfer

#### Kasia Trebska-McGowan^1^, Isaac Kriley^1^, Parisa Malekzadeh^1^, Eden Payabyab^1^, Richard Sherry^2^, Steven Rosenberg^1^, Stephanie L. Goff^1^

##### ^1^Surgery Branch, National Cancer Institute, National Institutes of Health, Bethesda, MD, USA; ^2^National Cancer Institute, Bethesda, MD, USA

###### **Correspondence:** Stephanie L. Goff (stephanie.goff@nih.gov)

**Background**

Adoptive transfer of tumor infiltrating lymphocytes (TIL) can effect long-term durable regression in patients with metastatic melanoma. The earliest studies utilized WHO criteria for evaluation of response, but more recent studies replaced the bidimensional analysis with simplified unidimensional criteria of RECIST 1.0. With improved cross-sectional imaging, there became concern that the pathophysiology of lymph node response required distinct evaluation criteria, as tumor clearance may not completely eradicate normal structures. RECIST 1.1 was developed and validated in a variety of histologies with various treatments, but has not been evaluated for adoptive cell transfer.

**Methods**

Eligible patients were enrolled on an IRB-approved protocol of adoptive cell transfer, randomizing patients to receive one of two lymphodepleting regimens prior to transfer of TIL. This study was reported using RECIST 1.0 criteria, with 24 complete responders and 30 partial responders among 99 treated patients [1]. The official tumor measurements of target lesions and notations of non-target lesions were re-evaluated using RECIST 1.1 criteria, which limits the total number of target lesions, the number of target lesions/organ, and uses short-axis measurements for lymph node disease.

**Results**

By design, the number of target lesions/patient was decreased, from 4 (range 1-10) to 3 (range 0-5). Thirty-eight lymph nodes did not meet short-diameter criteria for target lesions (10-14mm), and an additional 12 measured <10mm were reclassified as “non-pathologic”. With retrospective application of RECIST 1.1, three patients would not have met eligibility criteria for lack of evaluable disease. In assessing overall response to treatment, 25 patients met CR criteria, with an additional 27 with PR. While there were five patients who achieved CR at an earlier time point, overall time to response was not significantly different (median 16.0 v 19.8 months p=0.19). One patient with lymph node disease did not achieve CR by original criteria, was an early CR in this analysis, but recurred three months later.

**Conclusions**

Adoption of RECIST 1.1 demonstrated comparable response rates for this trial. A hallmark of our modern studies is the durability of complete responses with RECIST 1.0, and only by further application of the new criteria will we be able to confirm the validity of lymph node response criteria in adoptive cell transfer.

**Trial Registration**

ClinicalTrials.gov identifier NCT01319565.

**References**

1. Goff SL, Dudley ME, Citrin DE, Somerville RP, Wunderlich JR, Danforth DN, *et al*: **Randomized, Prospective Evaluation Comparing Intensity of Lymphodepletion Before Adoptive Transfer of Tumor-Infiltrating Lymphocytes for Patients with Metastatic Melanoma.** *J Clin Oncol* 2016, **34(20)**:2389-2397.

### P14 Bioluminescent redirected lysis assay (BRLA) as an efficient potency assay to assess tumor-infiltrating lymphocytes (TILs) for immunotherapy

#### Aishwarya Gokuldass, Michelle A Blaskovich, Charlene Kopits, Brian Rabinovich, Michael T. Lotze

##### Lion Biotechnologies, Inc., Tampa, FL, USA

###### **Correspondence:** Aishwarya Gokuldass (aishwarya.gokuldass@lionbio.com)

**Background**

Administration of TILs is a promising treatment for patients with melanoma and other solid tumors. TIL therapy involves culturing and expanding T cells isolated from a patient’s tumor *in vitro* and then reinfusing them back into the patient. TIL antitumor activity is commonly measured using tumor cells from the patient’s tumor, when available. However, autologous tumor cell lines are difficult to grow consistently. In order to test the potency of TILs that are infused into patients, we developed a surrogate target cell line that can be used to assess the lytic potential of TILs in a BRLA.

**Methods**

Mouse mastocytoma P815 cells expressing endogenous CD16, transduced with eGFP and firefly luciferase were selected as a surrogate for autologous tumor cells. The CD16 Fc receptor binds anti-CD3 antibodies providing a potent TCR activation signal. The P815 cells were sorted and cloned and then co-cultured with TILs +/- anti-CD3 antibodies to assess tumor reactivity through TCR activation and basal non-specific killing. Following 4 hours of incubation, luciferin was added to the wells for 5 minutes. After the incubation, bioluminescence intensity was determined. The % cytotoxicity and survival were calculated thus: % Survival = (experimental luminescence-background)/ (maximum luminescence-background)*100; % Cytotoxicity = 100-(% Survival); and Interferon-γ release in the media of the co-cultured TILs and P815 cells was analyzed by ELISA and LAMP1 (CD107a) expression analyzed by flow cytometry to evaluate the cytotoxicity of TILs.

**Results**

TCR-mediated TIL cytotoxicity can be measured in a highly sensitive dose-dependent manner. As shown below, this assay is highly sensitive. The following data will be presented: Highly Sensitive Potency Assay for TILs. A) % cytotoxicity of TIL (from patient M1033T-1) when co-cultured with P815 Clone G6 (with and without anti-CD3 antibody) at different ratios of effector to target cells. B) ELISA data showing amount of IFN-γ released at different ratios of effector to target cells. C) % LAMP1 marker expressed by the M1033T-1 when co-cultured with P815 Clone G6 and anti-CD3 at 1:1 effector to target cells for 4 and 24hr co-culture.

**Conclusions**

Our ‘Bioluminescent Redirected Lysis Assay’ (BRLA) using an engineered P815 cell line can be used as an assay to measure TIL potency. It requires no radionuclides and is more efficient than traditional cytotoxicity assays.

**Fig. 26:**
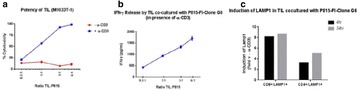
**(Abstract P14). a** % cytotoxicity of TIL (from patient M1033T-1) when co-cultured with P815 Clone G6 (with and without anti-CD3 antibody) at different ratios of effector to target cells. **b** ELISA data showing amount of IFN-γ released at different ratios of effector to target cells. **c** % LAMP1 marker expressed by the M1033T-1 when co-cultured with P815 Clone G6 and anti-CD3 at 1:1 effector to target cells for 4 and 24hr co-culture

### P15 Culturing and live cell confocal imaging of ovarian cancer spheroids with monocytes and interferons

#### Daniel S. Green^1^, Olena Kamenyeva^2^, Kathryn C Zoon^3^, Christina M Annunziata^1^

##### ^1^Translational Genomics Section, Women's Malignancy Branch, National Cancer Institute, Bethesda, MD, USA; ^2^Biological Imaging Section, Research Technologies Branch, National Institute of Allergy and Infectious Disease, National Institutes of Health, Bethesda, MD, USA; ^3^Laboratory of Infectious Diseases, National Institute of Allergy and Infectious Disease, National Institutes of Health, Bethesda, MD, USA

###### **Correspondence:** Daniel S. Green (daniel.green2@nih.gov)

**Background**

Ovarian cancer is the number one cause of death due to gynecological malignancies and the fifth leading cause of death in cancer in women. 75% of patients will have recurrent disease. Recurrent disease is chemotherapy refractive that progresses to chemotherapy resistant disease. Currently there are no definitive second-line treatments for patients. Patients with ovarian cancer have a 5-year survival rate of 25-30%, making it one of the most lethal malignancies.

**Methods**

One of the limitations of *in vitro* studies is the use of immortalized cell lines grown in two-dimensional flasks. To address this issue, many laboratories have been growing spheroids. We have employed 3-dimensional culturing techniques to create complex spheroids from ovarian cancer cell lines that have phenotypic structure similar to metastatic ovarian cancer. To further characterize spheroids we have created a system for sorting spheroids based on size. The spheroids can then be cultured in 2-dimensions for the study of chemotherapy sensitivities, and the efficacy of monocyte and interferon therapies for the treatment of ovarian cancer. Further, we have developed a single-photon confocal microscopy protocol for the multi-parameter imaging of live spheroids and monocytes with and without interferons. To create experimental robustness, we have employed a technique to image multiple conditions (4) over long periods of time (14-16 hours), allowing for the simultaneous imaging of both control and experimental conditions. Post-acquisition analysis of the images can be used to study migration of the individual cells within the spheroids, loss of cell dye viability, and migration of monocytes into the spheroids.

**Results**

We have found that the size of the spheroid defines, in part its sensitivity to standard chemotherapy agents. Post-acquisition analysis of the images have been used to study migration of the individual cells within the spheroids, loss of cell dye viability, and migration of monocytes into the spheroids. The addition of interferons with or without monocytes significantly reduces the movement of the individual cells within the spheroids. Furthermore, we have found that the addition of interferons slows monocyte migration and initiates monocyte attachment and entry into the spheroids.

**Conclusions**

The combination of novel cell culturing techniques with modern imaging and post-acquisition data analysis will increase our understanding of ovarian cancer response to both standard chemotherapy and novel cell based therapies.

**Acknowledgements**

This work was supported by the Intramural Research Program of the National Institutes of Health (NIH), National Institute of Allergy and Infectious Diseases (NIAID), and National Cancer Institute (NCI).

### P16 A novel xenograft model of chimeric antigen receptor-mediated toxicity sheds light on the influence of T cell source on the severity of the toxic sequellae

#### Joanne Hammill, Christopher Helsen, Craig Aarts, Jonathan Bramson

##### McMaster University, Hamilton, ON, Canada

###### **Correspondence:** Joanne Hammill (hammilja@mcmaster.ca)

**Background**

Chimeric antigen receptor (CAR)-engineered T lymphocytes are demonstrating striking clinical success, yet these treatments can be accompanied by severe on- and off-tumor toxicities. The availability of murine models to study these toxic phenomenon are currently lacking.

**Methods**

In our model, human T lymphocytes are engineered with a second-generation CD28-based CAR, targeted with a designed ankyrin repeat protein (DARPin) with picomolar affinity against HER2 (anti-HER2 DARPin CAR-T cells), and adoptively transferred into NRG mice at doses ranging from 6 x 10^5^ – 1.2 x 10^7^ CAR-T cells/mouse.

**Results**

Toxicity manifested in a drop in core body temperature and weight and, in some cases, lethality. The onset and severity of the toxicity varied with the source of the T lymphocytes (i.e., donor) used to generate CAR-T cells. We evaluated seven different donors and the toxicities associated with each donor’s cell product was reproducible across multiple experiments and multiple manufacturing runs. Anti-HER2 DARPin CAR-T cells were toxic in tumor-free mice, but toxicities were most severe in the presence of HER-2+ tumors demonstrating both on-tumor and off-tumor effects. The CAR-T cells did not respond to murine HER-2 indicating that the toxicity was both off-tumor and off-target. The toxicity was not due to the DARPin itself, as CAR-T cells bearing DARPins with other specificities were not toxic in this model. Further characterization of the model indicated that severity of toxicity was dose-dependent and could be exacerbated by the presence of a HER2^+^ tumor. Moribund mice were found to have aggregates of T cells in their lungs, liver, and heart and displayed a cytokine storm in the blood. The toxicity was triggered by CD4+ T cells in the anti-HER2 DARPin CAR-T cell product. While anti-HER2 DARPin CAR-T cells generated from donors demonstrating reduced toxicity were able to mediate anti-tumor efficacy *in vivo* in a xenograft model of ovarian carcinoma at low doses, they exhibited a narrow therapeutic window consistent with data emerging from the clinic where CAR-T therapy is effective.

**Conclusions**

This model offers a promising avenue to test strategies for the prevention or mitigation of toxicities associated with adoptive CAR-T cell transfer as well as insights into the contribution of T cell source to toxicities. Investigations are ongoing.

**Acknowledgements**

This work was supported by the Samuel Family Foundation, the Terry Fox Foundation, the Canadian Breast Cancer Foundation, and Triumvira Immunologics.

### P17 Ex vivo generation of highly purified and activated natural killer cells from human peripheral blood in accordance with GMP/GCTP for clinical studies

#### Yui Harada, Yoshikazu Yonemitsu

##### Kyushu University, Fukuoka, Fukuoka, Japan

###### **Correspondence:** Yui Harada (rkfraile@med.kyushu-u.ac.jp)

**Background**

Natural killer (NK) cells play a crucial role during the innate immune responses, and as such form part of the body of immunological defense against various diseases, including infectious diseases and malignancies. Therefore, adoptive immunotherapy using NK cell is emerging as promising treatments for intractable malignancies; however, there has been still developing because of difficulties in culture, shortage of overall effector numbers, contamination of considerable numbers of T cells, and their limited anticancer potencies. We here established the simple feeder-free method to generate purified (>90%) and highly activated NK cells from human peripheral blood-derived mononuclear cells (PBMCs) in accordance with GMP/GCTP for clinical studies.

**Methods**

Under approval of the institutional ethical committee, PBMCs were collected from healthy volunteers by using CliniMACS Prodigy® (automatic/closed system). CD3^+^ cells were depleted by CliniMACS CD3 beads, and CD3-depleted PBMCs were cultured at a concentration of 5 x 10^5^ cells/ml with high concentration of hIL-2 and 5% human AB serum for 14 days. Fresh medium wad added every 4-5 days throughout the culture period. Then, we confirmed the expression of surface markers, CD107a mobilization and cell-mediated cytotoxicity against various tumor cells and normal cells with or without monoclonal antibody drugs *in vitro* and antitumor effects against K562 *in vivo*.

**Results**

Among the several parameters, we found that simply 1) only CD3-depletion, 2) high dose IL-2, and 3) use of specific culture medium were sufficient to obtain the highly purified, expanded (~200-fold) and activated CD3^-^/CD56^+^ NK cells from PBMCs. Almost all activated NK cells expressed lymphocyte-activated marker CD69, and showed dramatically high expression of NK activation receptors (i.e. NKG2D, NKp30, NKp46, etc.), interferon-g, perforin and granzyme B. Importantly, only 2 hours reaction at effector/target ratio=1:1 was sufficient to kill almost all K562 cells, and antitumor activity was also representative on tumor bearing mice *in vivo*. Cytolysis was specific for various tumor cells, but not for normal cells, irrespective of MHC class I expression.

**Conclusions**

These findings strongly support that NK cells activated by our method is purified, expanded, and near fully activated. The cells were currently under investigation in clinical trial (phase I/IIa).

### P18 T cell antigen couplers (TAC) demonstrate strong effectivity against solid tumors with no measurable toxicities, demonstrating an enhanced therapeutic index

#### Christopher Helsen^1^, Joanne Hammill^1^, Kenneth Mwawasi^1^, Galina Denisova^1^, Jonathan Bramson^1^, Rajanish Giri^2^

##### ^1^McMaster University, Hamilton, ON, Canada; ^2^Indian Institute of Technology Mandi, Mandi, India

###### **Correspondence:** Christopher Helsen (helsenc@mcmaster.ca)

**Background**

Engineering T cells with chimeric antigen receptors (CARs) is proving to be an effective method for directing T cells to attack tumors in an MHC-independent manner [1, 2]. Current generation CARs aim to recapitulate T cell signaling by incorporating modular functional components of the TCR and co-stimulatory molecules. We sought to develop an alternate method to re-direct the T cell receptor which employs the native TCR. To this end, we developed the T cell Antigen Coupler (TAC) technology, a membrane-anchored receptor redirects the TCR and co-receptor in the presence of tumor antigen.

**Methods**

The utility of the TAC receptor has been assessed using *in vitro* and *in vivo* assays. *In vitro* assays are based on receptor surface staining, cytokine release and cytotoxicity assays. *In vivo* studies examined the anti-tumor effect of TAC-engineered T cells against established xenografts.

**Results**

*In vitro* testing has demonstrated robust and specific cytokine production and cytotoxicity by TAC-engineered human T cells directed against either HER-2 or CD19. *In vivo* TAC-engineered T cells revealed strong activity against HER-2 expressing solid xenograft tumor models such as MDA-MB-231 and OVCAR-3. Curiously, the TAC-engineered T cells outperformed a matched CD28-based second generation CAR in these models, demonstrating both increased anti-tumor efficacy and reduced toxicity. Whereas, mice treated with CAR engineered T cells showed serious toxicities that were both donor- and dose-dependent, we did not observe any toxicities arising from the TAC-engineered T cell, even at doses that produced complete tumor regression.

**Conclusions**

These differences in toxicities and efficacy highlight the biological differences of TAC and CAR receptors and indicated the potential for a superior therapeutic index for TAC engineered T cells. Current TAC’s in development target lymphatic malignancies and have shown great promise in early *in vitro* and *in vivo* assays.

**Acknowledgements**

Samuel Family Foundation, the Terry Fox Foundation, the Canadian Breast Cancer Foundation and Triumvira Immunologics.

**References**

1. Barrett DM, Singh N, Porter DL, Grupp S, June CH: **Chimeric antigen receptor therapy for cancer.** *Annu Rev Med* 2014, **65**:333–347.

2. Gill S, June CH: **Going viral: chimeric antigen receptor T-cell therapy for hematological malignancies.** *Immunol Rev* 2015, **263** :68–89.

3. Zhao Z, *et al*: **Structural Design of Engineered Costimulation Determines Tumor Rejection Kinetics and Persistence of CAR T Cells.** *Cancer Cell* 2015, **28**:415–428.

4. Sadelain M, Brentjens R, Rivière I: **The basic principles of chimeric antigen receptor design.** *Cancer Discov* 2013, **3**:388–398.

### P19 T cell receptor gene engineered T cells targeting human papillomavirus (HPV)-16 E7 induce regression of HPV-16+ human tumors in a murine model

#### Benjamin Jin^1^, Tracy Campbell^1^, Lindsey M Draper^2^, Sanja Stevanovic^1^, Zhiya Yu^3^, Bianca Weissbrich^4^, Nicholas P Restifo^3^, Cornelia L Trimble^5^, Steven Rosenberg^3^, Christian S Hinrichs^3^

##### ^1^Experimental Transplantation and Immunology Branch, National Cancer Institute, Bethesda, MD, USA; ^2^National Cancer Institute, Bethesda, MD, USA; ^3^Surgery Branch, National Cancer Institute, Bethesda, MD, USA; ^4^Kite Pharma EU, Amsterdam, Noord-Holland, Netherlands; ^5^Johns Hopkins University, Baltimore, MD, USA

###### **Correspondence:** Christian S Hinrichs (hinrichs@nih.gov)

**Background**

The human papillomavirus (HPV)-16 E7 oncoprotein is constitutively expressed by HPV-16-associated cancers and absent from healthy tissues, and it is therefore an attractive therapeutic target for T cell receptor (TCR) gene engineered T cell therapy. However, E7 displays manifold mechanisms of immune evasion, and its potential as a target for TCR-T cell therapy is unknown.

**Methods**

The nucleotide sequence of a TCR targeting HPV-16 E7_11-19_ was determined by study of cervix-infiltrating T cells from a subject with HPV-16+ cervical intraepithelial neoplasia. Expression of the TCR was optimized by hydrophobic substitutions in the alpha chain transmembrane region and introduction of an additional inter-chain TCR constant region disulfide bond. Second-party T cells genetically engineered to express the optimized TCR (E7 TCR T cells) were evaluated in *in vitro* immunologic assays and in an *in vivo* murine model of human cervical cancer.

**Results**

E7 TCR T cells displayed binding to HLA-A*02:01-E7_11-19_ tetramers. HLA-A*02:01 restriction was confirmed with major histocompatibility complex (MHC) blocking and with testing for recognition of target cells either expressing or lacking HLA-A*02:01. In functional assays, E7 TCR T cells recognized and killed a panel of HLA-A*02:01+ HPV-16+ cervical and oropharyngeal cancer cells lines but not cell lines that lacked either the restriction element or target antigen. Tumor recognition by CD8 and CD4 T cells that expressed the E7 TCR was observed. They displayed minimal if any cross-reactivity against peptides from human proteins that were identified by BLAST search based on protein sequence similarity or the presence of key shared residues mapped by alanine scanning of E7_11-19_. E7 TCR T cells recognized E7_11-19_ peptide-pulsed T2 cells at concentrations as low as 10^-11^ M indicating relatively high functional avidity. Direct comparison to a previously described E6-specific TCR revealed greater functional avidity, a slower TCR-pMHC Koff-rate, and superior tumor cell recognition for the E7 TCR. In a murine xenograft model, a single intravenous injection of E7 TCR T cells induced complete regression of tumors from one human cervical cancer line and controlled tumors from another human cervical cancer line.

**Conclusions**

E7 TCR T cells specifically recognized and killed HPV-16+ cancer cells *in vitro* and mediated regression of HPV-16+ tumors *in vivo*. These findings provide the preclinical basis for a new personalized TCR-T cell therapy for metastatic HPV-16+ cancers including many cervical, oropharyngeal, and anal malignancies. A clinical trial for E7 TCR T cells is now active (NCI-16-C-0138).

### P20 haNK, a cytotoxic human high affinity natural killer cell line, exerts enhanced ADCC mediated by avelumab (an anti-PD-L1 antibody) against multiple human tumor cell lines

#### Kwong Tsang^1^, Massimo Fantini^1^, James W Hodge^2^, Rika Fujii^2^, Ingrid Fernando^1^, Caroline Jochems^1^, Christopher Heery^1^, James Gulley^1^, Patrick Soon-Shiong^3^, Jeffrey Schlom^4^

##### ^1^Laboratory of Tumor Immunology and Biology, National Cancer Institute, National Institutes of Health, Bethesda, MD, USA; ^2^National Cancer Institute, National Institutes of Health, Bethesda, MD, USA; ^3^NantKwest, Inc., Culver City, CA, USA; ^4^Center for Cancer Research, National Cancer Institute, National Institutes of Health, Bethesda, MD, USA

###### **Correspondence:** James W Hodge (jh241d@nih.gov)

**Background**

Immune checkpoints have been implicated in the down-regulation of antitumor immunity. Anti-PD-1/PD-L1 checkpoint inhibitory monoclonal antibodies (mAbs) can restore immune function in the tumor microenvironment, and have demonstrated clinical benefit in patients with melanoma, Hodgkin’s lymphoma, lung and bladder carcinomas, and other tumor types. In addition to its checkpoint inhibitory function, avelumab, a fully human IgG1 anti-PD-L1 mAb, can mediate antibody-dependent cellular cytotoxicity (ADCC) to lyse human tumor cells in the presence of natural killer (NK) cells [1]. NK cells can be used for cancer therapy. However, obtaining sufficient numbers of functionally active NK cells from patients is technically challenging since only about 10% of the population expresses on NK cells the high-affinity FcR that provides maximum ADCC. One alternative is to use established NK cell lines that have antitumor activity. High affinity NK cells (haNK), provided by NantKwest, derived from the human NK cell line NK-92, are genetically engineered to express high-affinity human CD16 (FcgRIIIA-V158) and transduced with the human IL-2 gene. In addition, haNK cells have little inhibitory killer-cell immunoglobulin-like receptor (KIR) expression, a unique feature that may be a factor in their highly cytotoxic activity against a broad range of malignancies. We report here our investigation of 1) whether haNK cells used in combination with avelumab can lyse human tumor cells via the ADCC mechanism, and 2) the factors that may influence this cytotoxic activity.

**Methods**

Cell lines used in our experiments included human carcinomas of the head and neck, cervix, bladder, and colon, as well as prostate and pancreatic cancers. haNK cells irradiated with 10 Gy were used as effector cells at various effector-to-target-cell ratios in all experiments. Four- and 18-hour ^111^In release assays were performed to evaluate ADCC activity.

**Results**

Our results show that 1) haNK cells can lyse a range of human carcinoma cells when avelumab is used to target PD-L1 expression; 2) the addition of anti-CD16 neutralizing antibody significantly inhibits ADCC lysis, implicating CD16 ligation as a major mechanism of action for ADCC lysis mediated by haNK and avelumab; and 3) in combination with avelumab, haNK cells mediate significantly higher levels of ADCC lysis than do NK cells isolated from healthy donor peripheral blood mononuclear cells (PBMC).

**Conclusions**

These results provide a rationale for using haNK cells in combination with avelumab or other ADCC-mediating cytotoxic mAbs to treat human malignancies.

### P21 Adoptive transfer of *ex vivo*-expanded PD-1+ CD8+ and CD4+ T cells eliminates myeloma in mice

#### Weiqing Jing, Jill Gershan, Grace Blitzer, James Weber, Laura McOlash, Bryon D Johnson

##### Medical College of Wisconsin, Milwaukee, WI, USA

###### **Correspondence:** Bryon D Johnson (bjohnson@mcw.edu)

**Background**

Adoptive T cell therapy (ACT) has emerged as a potential curative therapy for patients with advanced solid tumors. However, for hematologic cancers, identifying and enriching the cancer antigen-reactive T lymphocytes for ACT remains a challenge. Our lab previously demonstrated that blockade of the PD-1/PD-L1 pathway (in the context of non-myeloablative, lymphodepleting whole body irradiation) was capable of eliminating established myeloma in mice [1, 2]. In the current study, we tested whether PD-1 is a marker for myeloma-reactive T cells.

**Methods**

C57BL/KaLwRij (KaLwRij) mice were inoculated with 5T33 myeloma cells intravenously, and 28 days later, splenic PD-1+ and PD-1- T cells (CD8+ and CD4+) were isolated by flow cytometric sorting. The purified T cells were expanded in culture for 7 days on plate-bound CD3 and soluble CD28 antibodies plus IL-2, IL-7 and IL-15. Some of the expanded T cells were assayed *in vitro* for myeloma reactivity. Equal mixtures of expanded CD4+ and CD8+ T cells, or each subset alone, were infused to myeloma-bearing Rag1-deficient recipients, and the mice were followed for myeloma progression.

**Results**

We found that numbers of cancer antigen-reactive T lymphocytes in myeloma-bearing mice were enriched in PD-1+ CD8+ and CD4+ T cell subsets. PD-1+ T cells could be efficiently expanded *ex vivo* for adoptive transfer, and the expanded cells maintained their anti-myeloma reactivity. Adoptive transfer of the *ex vivo*-expanded PD-1+ T cells effectively eliminated established myeloma in Rag1-deficient recipients. In contrast, adoptive transfer of expanded PD-1- T cells failed to demonstrate anti-myeloma efficacy *in vivo*. Notably, both CD8+ and CD4+ PD-1+ T cell subsets played a role in eradicating myeloma, but combined administration of the *ex vivo*-expanded subsets was most efficacious.

**Conclusions**

Our results show that PD-1 is a biomarker for myeloma-specific CD8+ and CD4+ T cells in mice. Furthermore, these PD-1-expressing T cells can be expanded in culture for effective adoptive cell immunotherapy of myeloma-bearing recipients.

**Acknowledgements**

Support from the Midwest Athletes Against Childhood Cancer (MACC) Fund, Children's Research Institute, and a former grant from the Multiple Myeoma Research Foundation.

**References**

1. Jing W, Gershan JA, Weber J, Tlomak D, McOlash L, Sabatos-Peyton C, Johnson BD: **Combined immune checkpoint protein blockade and low dose whole body irradiation as immunotherapy for myeloma**. *J Immunother Cancer* 2015, **3**:2.

2. Kearl TJ, Jing W, Gershan JA, Johnson BD: **Programmed death receptor-1/programmed death receptor ligand-1 blockade after transient lymphodepletion to treat myeloma**. *J Immunol* 2013, **190**:5620-5628.

### P22 Entinostat sensitized osteosarcoma cells for cytotoxic effect of natural killer cells

#### Simin Kiany, Huang Gangxiong, Eugenie S Kleinerman

##### University of Texas MD Anderson Cancer Center, Houston, TX, USA

###### **Correspondence:** Simin Kiany (skiany@mdanderson.org)

**Background**

The goal of this study is to find an alternate therapy for osteosarcoma (OS) lung metastasis. We previously showed that NK cell therapy significantly decreased, but did not cure, OS lung metastasis in a mouse model. Other studies have shown that histone deacetylase inhibitors (HDACi) sensitize tumor cells to NK cell cytotoxicity, primarily by increasing expression of ligands for NK cells on tumor cells; thus, to augment NK cell therapy, we combined it with the HDACi, entinostat.

**Methods**

Flow cytometry, western blot analysis, and Q-PCR were used to determine whether entinostat increased expression of NK cell ligands on OS cells. Effects of entinostat on NK cell viability, receptor expression, and cytotoxicity were explored using a viability test, flow cytometry, and calcein release assay, respectively. NK cells were isolated from blood buffy coats and were expanded *ex vivo* using genetically engineered K562 cells and human IL-2. Q-PCR was used to measure microRNAs expression in OS cells and a CHIP assay was used to determine increased histone acetylation at the MICA/B gene promoters. For the *in vivo* study, mice were given 10, 5, or 2.5 mg/kg of entinostat orally to determine the subtherapeutic dose of the drug.

**Results**

We demonstrated that 2 μM entinostat for 48 h upregulated expression of NK cell ligands on OS cell lines. Increased expression of ligands on OS cells resulted in increased susceptibility of OS cell lines to NK cell cytotoxicity *in vitro.* NK cell treatment with up to 2 μM entinostat did not affect NK cell viability or NK cell receptor (NKG2D, NKp30, NKp44, NKp46, and DNAM-1) expression. NK cells pretreated with entinostat for 24 h did not decrease cytotoxicity of NK cells to OS cell lines. We also showed two mechanisms by which entinostat controls MICA/B expression on OS cells: 1) by increasing H4 acetylation at the MICA/B genes promoters and 2) by down-regulating mir-20a, mir-93, and mir-106b expression. We demonstrated that the sub-therapeutic dose of entinostat that significantly increased MICA/B on OS lung metastasis was 5 mg/kg three times a week for 5 weeks. Combining 5mg/kg entinostat and NK cell therapy is our ongoing *in vivo* experiment.

**Conclusions**

We demonstrated that entinostat immunosensitized OS cells to NK cell lysis by inducing upregulation of ligands for NK cells on OS cells. Our results suggest that NK cell therapy combined with entinostat provides an innovative approach to enhance the immunotherapeutic effect of NK cells against OS pulmonary metastases.

### P23 Chimeric antigen receptor macrophages (CARMA) for adoptive cellular immunotherapy of solid tumors

#### Michael Klichinsky, Marco Ruella, Olga Shestova, Saad Kenderian, Miriam Kim, John Scholler, Carl H June, Saar Gill

##### Center for Cellular Immunotherapies, University of Pennsylvania, Philadelphia, PA, USA

###### **Correspondence:** Michael Klichinsky (mklich@mail.med.upenn.edu)

**Background**

Anti-CD19 chimeric antigen receptor (CAR) redirected T cells have demonstrated profound efficacy in relapsed/refractory B cell malignancies. However, CAR T cells have thus far failed to recapitulate these results in solid tumors. Accumulating evidence suggests that macrophages naturally traffic to and persist in solid tumors. The goal of this study was to evaluate the anti-tumor function of genetically engineered CAR macrophages (CARMA) and assess their potential for translation as a novel immunotherapeutic platform for solid tumors.

**Methods**

To examine the function of CARs in macrophages, first generation CARs with a CD3ζ intracellular domain were introduced into the THP-1 macrophage model. *In vitro* function was assessed via quantitative phagocytosis and luciferase-based specific killing assays. To assess the function of the CD3ζ-domain, CD3ζ-null CAR constructs were compared. Ad5f35 was used to transduce primary human monocyte derived macrophages with an anti-HER2 CAR construct, and anti-tumor function was tested *in vitro* and *in vivo*. The impact of inhibiting the CD47/SIRPα “do-not-eat-me” signal was tested with blocking antibodies and CRISPR-Cas9 mediated SIRPα knockout. Macrophage M1/M2 phenotype was determined by flow cytometry. Immunodeficient mouse xenograft models of a human HER2(+) ovarian cancer cell-line (SKOV3) were used for *in vivo* efficacy studies.

**Results**

**Fig. 27:**
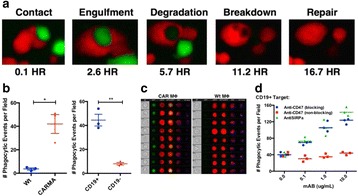
**(Abstract P23).** Timelapse of a CAR19 macrophage (mRFP+) phagocytosing a CD19+ K562 cells (GFP+) cell (A). CARMA but not Wt macrophages phagocytosed CD19+ but not CD19- tumor cells (B). Phagocytosis was confirmed by Imagestream cytometry, gating on mRFP+GFP+ events (C). Blockade of CD47 and SIRPa led to a dose dependent increase in CARMA phagocytosis. Non-blocking anti-CD47 clone 2D3 (opsonization control) did not enhance phagocytosis (D)

**Fig. 28:**
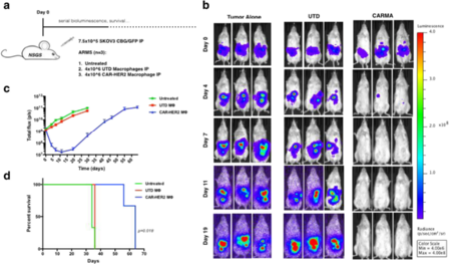
**(Abstract P23).** Anti-HER2 primary human CARMA were tested in immunodeficient mouse models of human HER2+ ovarian cancer (schema, **a**). CARMA but not control untransduced macrophages reduced tumor burden (**b**, **c**) and prolonged survival by 30 days (p=0.018, **d**)

CAR19, but not untransduced macrophages, phagocytosed CD19+ (but not CD19-) K562 cells. Deletion of CD3ζ in CAR19 macrophages abrogated phagocytosis and significantly reduced specific killing. Phagocytosis was inhibited by pharmacologic blockade of phagocytic signaling - suggesting that CAR activation drives productive cell-signaling in macrophages. Phagocytosis was also demonstrated in HER2 and mesothelin CARMA models. Blockade of the inhibitory CD47/SIRPα “do-not-eat-me” signal enhanced CARMA phagocytosis of antigen-bearing target cells in a dose-dependent manner (Fig. 27). We identified Ad5f35 as an efficient transduction approach for engineering primary human macrophages, resulting in >70% CAR transduction efficiency. Primary human anti-HER2 CARMA demonstrated targeted phagocytosis and specific killing. Ad5f35 transduction polarized human macrophages to the pro-inflammatory M1 phenotype, and rendered them resistant to downstream M2 subversion by immunosuppressive cytokines and cell lines. Anti-HER2 CARMA was evaluated *in vivo* in an ovarian cancer xenograft model. Mice that received CARMA had a decrease in tumor burden of approximately two orders of magnitude and had a 30-day survival benefit relative to untreated or control macrophage treated mice (p=0.018, Fig. 28).

**Conclusions**

Here, we introduce for the first time that human macrophages engineered with a CAR exert antigen-specific tumor phagocytosis and killing, and propose a novel immunotherapeutic platform for the treatment of diverse solid tumors.

### P24 Regulation of T cell sensitivity by TCR-proximal signaling components during anti-melanoma responses

#### Duane Moogk^1^, Shi Zhong^2^, Zhiya Yu^3^, Ivan Liadi^4^, William Rittase^5^, Victoria Fang^6^, Janna Dougherty^1^, Arianne Perez-Garcia^7^, Iman Osman^8^, Cheng Zhu^5^, Navin Varadarajan^4^, Nicholas P Restifo^3^, Alan Frey^9^, Michelle Krogsgaard^10^

##### ^1^Perlmutter Cancer Center, NYU School of Medicine, New York, NY, USA; ^2^Xiangxue Pharmaceutical Co., Ltd., Guangdong, Guizhou, People’s Republic of China; ^3^Surgery Branch, National Cancer Institute, Bethesda, MD, USA; ^4^University of Houston, Houston, TX, USA; ^5^The George W. Woodruff School of Mechanical Engineering, Georgia Institute of Technology, Atlanta, GA, USA; ^6^Skirball Institute of Biomolecular Medicine, NYU School of Medicine, New York, NY, USA; ^7^Kite Pharma, Inc., Santa Monica, CA, USA; ^8^Ronald O. Perelman Department of Dermatology and Department of Medicine and Urology, Perlmutter Cancer Center at NYU School of Medicine, New York, NY, USA; ^9^New York University Langone School of Medicine, New York, NY, USA; ^10^Department of Pathology and Perlmutter Cancer Center at NYU School of Medicine, New York, NY, USA

###### **Correspondence:** Michelle Krogsgaard (krogsm01@nyumc.org)

**Background**

Immunotherapies for cancers have made great strides in recent years, yet new and improved approaches are required to achieve more durable responses in a greater number of patients. The *in vitro* expansion phase of adoptive T cell therapy prior to reinfusion into the patient present opportunities to genetically enhance T cell subsets to improve *in vivo* performance. While the most common genetic modification is the incorporation of engineered antigen-specific TCRs or chimeric antigen receptors, modification to signaling pathways in T cell memory subsets in order to enhance T cell sensitivity is an underexplored strategy. This is mainly because contributions of TCR signaling components that confer differences in activation sensitivity and functional outcomes between CD8+ T_cm_ and T_em_ are unclear.

**Methods**

To understand how TCR-proximal signaling differs significantly between T cell memory subsets, we derived T_CM_ and T_EM_ from the humanized TCR-transgenic melanoma mouse model (JR209). We quantified differences in TCR activation and feedback regulation by novel live-cell imaging technologies, phospho-specific protein assays and used modeling of early TCR signaling to reveal the physiological significance of these differences.

**Results**

One of the critical steps of T cell triggering is the coordinated phosphorylation and binding of CD3 and Zap-70 by Lck following TCR ligation by pMHC. Here, we show that T_cm_ and T_em_ possess differential constitutive Lck activities. Immediately proximal to Lck signaling, we observed enhanced Zap-70 phosphorylation in TEM following TCR ligation compared with TCM. Further, we observed increased intracellular calcium influx and cytotoxic effector function in TEM compared with TCM, and provide evidence that this results from a lower probability of TCM reaching threshold activation signaling due to the decreased magnitude of TCR-proximal signaling. We show that the differences in Lck constitutive activity between CD8+ T_cm_ and T_em_ are driven in part by differential regulation by SH2 domain-containing phosphatase-1 (Shp-1) and C-terminal Src kinase (Csk). We demonstrate that inhibition of Shp-1 results in increased constitutive Lck and cytotoxic activity in T_CM_ to levels similar to that of T_EM_.

**Conclusions**

Together, this work demonstrates that differential activities of TCR-proximal signaling components may contribute to establishing the divergent effector properties of T_CM_ and T_EM_. Inhibition of negative regulatory molecules, for example Shp-1 or Csk, or generalized augmentation of T cell sensitivity with miRNA offer potential therapeutic approaches in T cell immunotherapy but must be considered in the context of specificity and optimal targeting.

### P25 Co-expression of synthetic PD-1 fusion proteins augments HER2 CAR T cell functionality against glioblastoma

#### Daniel Landi, Kristen Fousek, Malini Mukherjee, Ankita Shree, Sujith Joseph, Kevin Bielamowicz, Tiara Byrd, Nabil Ahmed, Meenakshi Hegde

##### Baylor College of Medicine, Houston, TX, USA

###### **Correspondence:** Daniel Landi (landi@bcm.edu)

**Background**

HER2-CAR T cells home to the central nervous system (CNS) and induce tumor regression in patients with glioblastoma (GBM) [1]. However, most responses are transient, as CAR T cells fail to expand and have limited persistence. *Ex vivo* analyses of tumor infiltrating lymphocytes demonstrate high levels of inhibitory receptors, including PD-1. Monoclonal antibodies blocking PD-1/PD-L1 induce responses in some patients with solid tumors and potentiate anti-tumor T cell activity. However, because antibodies exhibit erratic CNS pharmacokinetics, combining this approach with CAR T cells is not optimal for CNS tumors. We hypothesize that co-expression of a synthetic PD-1 fusion protein will convert PD-L1 into a costimulatory T cell signal, improving expansion, persistence, and anti-tumor activity of adoptively transferred HER2-CAR T cells.

**Methods**

We generated an array of bicistronic vectors encoding our clinically utilized 2^nd^ generation HER2-CAR (CD28ζ-endodomain) and a PD-1 fusion protein. All PD-1 fusion proteins contained the native PD-1 ectodomain fused to either the CD28 transmembrane and endodomain (PD-1/CD28) or CD8α-transmembrane and 4-1BB-endodomain (PD-1/4-1BB). T cell expansion, persistence, and exhaustion (LAG3, TIM3, PD-1) were measured using flow cytometry following coculture with autologous HER2+/PD-L1+ GBM cells for 2-4 weeks. Cytokine release at 24 hours was measured using standard ELISA, and the xCELLigence impedance-based system was used to evaluate cytolytic activity. Using high-resolution immunofluorescence imaging, we interrogated differences at the CAR T cell/GBM contact point, also referred to as the immunologic synapse (IS).

**Results**

**Fig. 29:**
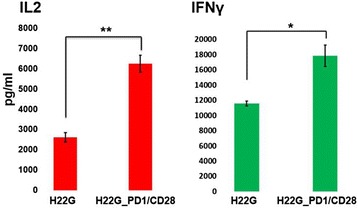
**(Abstract P25).** 2nd generation HER2 CAR T cells with and without PD-1/CD28 fusion proteins were incubated with autologous GBM cells at a 1:1 ratio. Cytokine concentrations from culture supernatant collected at 24 hours were measured using standard ELISA

**Fig. 30:**
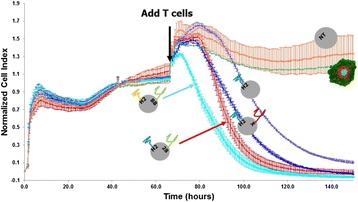
**(Abstract P25).** The xCELLigence platform uses impedance across resistor-coated plates to measure adherant tumor cell viability. Once 10,000 tumor cells became adherant and confluent, 1,000 T cells were added that expressed either a HER2 CAR only (H2) or a HER2 CAR with a truncated PD-1 protein (X), PD-1/CD28 protein (28), or PD-1/4-1BB protein (BB). Non-transduced T cells and tumor only wells served as controls

Compared to conventional HER2-CAR T cells, PD-1/CD28 T cells expanded more quickly with significantly higher IL2/IFNγ-release at 24 hours (Fig. 29), whereas PD-1/4-1BB T cells demonstrated enhanced cytolytic ability against autologous GBM cells in prolonged killing assays (Fig. 30) and better long-term persistence. Inhibitory receptor expression following coculture was comparable among T cell products, but PD-1/4-1BB T cells maintained a greater percentage of central memory cells (CCR7+/CD45RA-). HER2-CAR T cells bearing PD-1 fusion proteins demonstrated increased levels of activated kinases in CD28/4-1BB signaling pathways. Three-dimensional reconstitution and quantification of confocal images of the CAR T cell/tumor interface revealed increased stability of the IS between the HER2-CAR and the HER2 molecule on GBM.

**Conclusions**

We conclude that PD-L1 can be converted into a costimulatory signal using synthetic PD-1 fusion molecules to drive key T cell activation pathways and enhance stability of the CAR-target antigen interface, leading to improved HER2-CAR T cell functionality against GBM.

**References**

1. Ahmed N: **Autologous HER2 CMV bispecific CAR T cells for progressive glioblastoma: Results from a phase I clinical trial**. *J Clin Oncol* 2015, **33**:3008.

### P26 Adoptive therapy with tumor-infiltrating lymphocytes for melanoma: interim results of a phase II single-institution study

#### Sylvia Lee^1^, David Byrd^2^, John Thompson^3^, Shailender Bhatia^4^, Scott Tykodi^4^, Judy Delismon^5^, Liz Chu^5^, Siddiq Abdul-Alim^5^, Arpy Ohanian^5^, Anna Marie DeVito^6^, Stanley Riddell^5^, Kim Margolin^7^

##### ^1^Seattle Cancer Care Alliance, Fred Hutchinson Cancer Research Center, University of Washington, Seattle, WA, USA; ^2^University of Washington, Seattle, WA, USA; ^3^Clinical Research Division at Fred Hutch Cancer Center, University of Washington, Seattle, WA, USA; ^4^Fred Hutchinson Cancer Research Center, University of Washington, Seattle, WA, USA; ^5^Fred Hutchinson Cancer Research Center, Seattle, WA, USA; ^6^Seattle Cancer Care Alliance, Seattle, WA, USA; ^7^Department of Medical Oncology, City Of Hope, Duarte, CA, USA

###### **Correspondence:** Sylvia Lee (smlee@fredhutch.org)

**Background**

Adoptive cell therapy using tumor-infiltrating lymphocytes (TIL) has been established as an effective treatment option for metastatic melanoma but is available at only a small number of institutions. We have developed a TIL program at the Fred Hutchinson Cancer Research Center and present our initial results in melanoma patients.

**Methods**

Patients with metastatic melanoma were enrolled in a two-step fashion to a nonrandomized, phase II TIL trial: Step 1 for TIL generation and Step 2 for TIL treatment. TIL are cultured from tumor fragments, using standard methodologies developed at the NCI. At a time of future disease progression, patients are given cyclophosphamide 60mg/kg/day x 2 days, fludarabine 25mg/m2/day x 5 days, then TIL up to 1.5 x 10^11, followed by high-dose IL-2, 600,000 units/kg every 8 hours, up to 14 doses as tolerated. TIL populations are selected for infusion based on growth, phenotype, and MHC-class-I-dependent autologous tumor recognition, as assessed by interferon-gamma release.

**Results**

Thirty-two patients have been enrolled on Step 1 for TIL generation. Of these, 23(72%) achieved adequate TIL number of >40 x10^6 by 5 weeks of culture. TIL in 17(53%) patients also demonstrated positive autologous tumor reactivity. To date, 7 patients have received TIL therapy on Step 2; all had progressed on prior immunotherapy (Table 3). In these 7 patients, the RECIST 1.1 responses are: 2 CR, 1 PR, 3 SD, 1PD. All patients experienced tumor regression of some or all baseline target lesions; the patient with PD progressed in the brain, but experienced a partial response of extracranial sites. Two patients were on concurrent BRAF therapy and had reached a plateau in their response to BRAF inhibitors prior to start of TIL treatment, and both experienced further tumor reduction after TIL. Responses were associated with a higher percent CD8+TIL, a lower percent Treg(CD4+CD25+CD127-), and TCR oligoclonality, while functional markers PD-1 and TIM3 did not associate with response.

**Table 3:** **(Abstract P26).** Characteristics and responses of treated patients

	Age/Gender	Prior Treatments	M Classification and Metastatic Sites	Response	Response Duration (months)
1	54 F	Anti-CLA-4, interferon (IFN)	M1c: Mesentery, small bowel, lymph nodes (LN)	PR	8
2	34 F	Anti-PD1, anti-CLA-4, IFN, IL-2 + radiation (XRT), temozolomide	M1c: Brain, kidneys, adrenals, bone, liver, LN, subcutaneous (SC)	SD	3
3	61 M	IL-2, BRAF inhibitor	M1c: Brain, liver, lungs, SC, mesentery, chest wall	PD (Progression in brain, but PR in extracranial sites)	8, for extracranial response
4	27 F	IL-2, anti-CTLA-4+IL-21, anti-CTLA-4 + XRT, recombinant IL-15	M1c: Brain, SC, LN, pleura, kidneys, peritoneum	SD	3
5	63 M	Anti-CTLA-4	M1a: Intramuscular, LN	CR	17, ongoing
6	52 M	Anti-CTLA-4	M1a: SC, LN	CR	20, ongoing
7	31 F	Anti-PD1, anti-CTLA-4, decarbazine, BRAF/MEK inhibitor	M1c: Brain, lungs, liver, SC	SD	5

**Conclusions**

Our single-institution study validates the utility of TIL therapy in patients with advanced melanoma, refractory to other immunotherapy. TIL can induce durable CRs and can also mediate additional tumor regression in patients on active BRAF inhibition. The replication of TIL methodology across different institutions, with reproducible clinical efficacy, supports the feasibility of its widespread application, as well as further investigation into optimizing elements of this treatment modality.

**Acknowledgements**

We wish to thank the NCI Surgery Branch and MD Anderson for their generous guidance with the development of our TIL therapy program, and Prometheus for their generous IL-2 support.

### P27 Functional characterization of CD4+ and CD8+ CD19 chimeric antigen receptor T cells

#### Isabelle Magalhaes, Jonas Mattsson, Michael Uhlin

##### Karolinska Institutet, Stockholm, Stockholms Lan, Sweden

###### **Correspondence:** Isabelle Magalhaes (isabelle.magalhaes@ki.se)

**Background**

Chimeric antigen receptor (CAR) T cells targeting CD19 have shown dramatic results in patients with refractory B cell malignancies but the precise mechanisms of how CD19 CAR T cells kill tumor cells are not all well understood.

**Methods**

Second generation CD19 CAR T cells were produced from peripheral blood mononuclear cells (PBMCs) obtained from pediatric patients with acute lymphoblastic leukemia (ALL) and adult patients with chronic lymphocytic leukemia (CLL). Here we present the phenotype, cytokine and cytotoxic profile of CD4+ and CD8+ CD19 CAR T cells generated from patients and healthy donors (HDs).

**Results**

T cell frequency in PBMCs from patients (ALL and CLL) was low (<4% of lymphocytes). In patients with CLL, 90% of lymphocytes were CD19+ B cells. In patients with CLL, as compared to HDs, the majority of CD8+ T cells displayed a terminally differentiated phenotype, while CD4+ T cells were mostly effector memory cells. Retroviral transduction was performed on PBMCs without prior B cell depletion or T cell enrichment step. CAR T cell cytotoxicity towards CD19+ target cells was mediated via granzymes, but not perforin, Fas-L or TRAIL. In patients and HDs, CD4+ and CD8+ CD19 CAR T cells showed a comparable cytokine (IL-2, IFN-g, TNF) production, CD107a expression in response to stimulation with K562 CD19+ cells. The majority of CAR T cells were polyfunctional (≥2 functions). However, patients with CLL, as compared to HDs and patients with ALL, displayed a higher frequency of IFN-g producing and polyfunctional CD4+ and CD8+ CD19 CAR T cells. When stimulated with autologous CD19+ B cells, as compared to K562 CD19+, lower frequencies of CD4+ and CD8+ CD19 CAR T cells produced IL-2, IFN-g and TNF. The frequency of polyfunctional CD19 CAR T cells was lower when stimulated with autologous B cells as compared to K562 CD19+ cells. Stimulation with other CD19+ cell lines induced a different cytokine production profile of CD19 CAR T cells.

**Conclusions**

CD4+ and CD8+ CD19 CAR T cells display comparable differentiation phenotype, cytokine production and cytotoxic capacity. The presence of a high frequency of CD19+ B cells during the generation of CAR T cells did not have an impact on CAR T cell phenotype. However CAR T cells from patients with CLL produced more cytokines when stimulated with CD19+ target cells suggesting that activation of CD19 CAR T cells by B cells during cell expansion impacts the cytokine profile. Furthermore, our data show that the level of CD19 cell surface expression modulates CAR T cells cytokine production.

### P28 Directing traffic: fostering chemokine receptor expression on tumor-infiltrating lymphocytes improves re-trafficking in vivo

#### Satoshi Nemoto^1^, Patricio Pérez Villarroel^1^, Ryosuke Nakagawa^1^, James J Mule^2^, Adam W. Mailloux^1^

##### ^1^Translational Science, H. Lee Moffitt Cancer Center, Tampa, FL, USA; ^2^Immunology Program, Cutaneous Oncology Program, H. Lee Moffitt Cancer Center, Tampa, FL, USA

###### **Correspondence:** Adam W. Mailloux (adam.mailloux@moffitt.org)

**Background**

Previously, a critical role for chemokines was found in a unique immune-related gene expression signature (GES) for immune cell recruitment and tertiary lymphoid structure formation in metastatic melanoma [1]. Regarding adoptive cell therapy (ACT) of tumor-infiltrating lymphocytes (TIL), we hypothesized that expression of chemokine receptors (CCR)2, CCR5, and CCR7, which bind multiple GES chemokines, favor TIL re-trafficking to the tumor, thereby bolstering ACT efficacy.

**Methods**

We utilized a preclinical ACT model in which TIL from syngeneic MC-38 colon carcinomas grown in wild-type (WT) C57BL/6 mice, or gene knock-out (KO) C57BL/6 mice lacking CCR2, CCR5, or CCR7, were expanded *ex vivo*, and adoptively transferred into MC-38-bearing C57BL/6 recipient mice bearing congenic CD45.1. After seven days, tumor burden was assessed, and Spanning-tree Progression Analysis of Density-normalized Events (SPADE) of 18-parameter cytometry data was used to identify and quantify TIL subsets, and ^51^Cr-release assays quantified cytotoxicity among sorted TIL populations.

**Results**

**Fig. 31:**
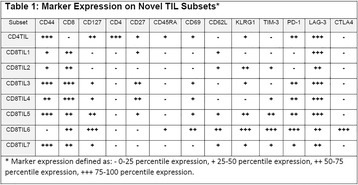
**(Abstract P28).**

ACT of WT TIL reduced tumor burden by 50% compared to untreated mice (p=0.0098). This benefit was lost when transferring CCR5KO or CCR7KO TIL, and reduced when transferring CCR2KO TIL (all p < 0.05). MC-38 tumors contained 90%, 85%, and 70% less transferred TIL with ACT of CCR2KO, CCR5KO, or CCR7KO TIL, respectively, compared to WT TIL (all p < 0.01) . SPADE identified eight novel subsets in re-trafficked TIL with unique patterns of activation, memory, and immune checkpoint markers (Fig. 31). Nearly all subsets displayed impaired re-trafficking with CCR2KO, CCR5KO, or CCR7KO TIL ACT (all p < 0.05). Cytotoxicity assays suggest a range of cytotoxic potential among identified subsets, with CD8^+^TIL3 and 4 subsets being most cytotoxic (all p < 0.05). During TIL expansion, IL-2 up-regulated CCR2, CCR5, and CCR7 in a cell density-dependent fashion (all p < 0.007). ACT of TIL with up-regulated CCRs increased tumor re-trafficking three-fold (p < 0.05), and decreased tumor burden by an additional 25% (p < 0.05) versus WT TIL without up-regulated CCRs.

**Conclusions**

CCR2, CCR5, and CCR7 are vital for TIL re-trafficking to MC-38 tumors following ACT, including cytotoxic subsets among eight novel TIL phenotypes. Fostering CCR expression during TIL expansion augments ACT efficacy in murine MC-38 colon carcinoma. Translational studies to human TIL ACT are currently underway.

**Acknowledgements**

Supported by NCI–NIH (CA148995-01; P30CA076292; P50CA168536), V Foundation, Dr. Miriam and Sheldon G. Adelson Medical Research Foundation, and the Chris Sullivan Foundation.

**References**

1. Messina JL, Fenstermacher DA, *et al*: **12-Chemokine gene signature identifies lymph node-like structures in melanoma: potential for patient selection for immunotherapy?** *Scientific Reports* 2012, **2**:765-770.

### P29 Provision of inducible MyD88 and CD40 co-stimulation in CAR T cells results in potent antitumor activity in preclinical solid tumor models

#### Melinda Mata^1^, Phuong Nguyen^1^, Claudia Gerken^1^, Christopher DeRenzo^1^, David M Spencer^2^, Stephen Gottschalk^1^

##### ^1^Baylor College of Medicine, Center for Cell and Gene Therapy, Houston, TX, USA; ^2^Bellicum Pharmaceuticals and Baylor College of Medicine, Houston, TX, USA

###### **Correspondence:** Melinda Mata (mxmata@texaschildrens.org)

**Background**

Although adoptive immunotherapy using T cells expressing chimeric antigen receptors (CAR) is successful in refractory hematological malignancies, limited clinical responses have been observed in solid tumors. We reasoned that introducing an inducible co-stimulation (iCO-STIM) gene into T cells would allow for improved activation of CAR T cells, resulting in enhanced antitumor activity. Due to the co-stimulatory properties of MyD88 and CD40 in T cells, we explored whether CAR T cells expressing an iCO-STIM molecule consisting of a myristoylation-targeting sequence, two FKBP dimerizing domains, MyD88ΔTIR, and the intracellular domain of CD40, have superior effector function relative to standard CAR T cells *in vitro* and *in vivo*.

**Methods**

T cells expressing a HER2-CAR and iCO-STIM (HER2-CAR/iCO-STIM T cells) were generated by transduction with a retroviral vector encoding FRP5.ζ (HER2-CAR), a 2A peptide, and iCO-STIM. HER2-CAR/iCO-STIM T cell effector function was then evaluated *in vitro* and in murine xenograft models.

**Results**

In the presence of CID (AP20187), HER2-CAR/iCO-STIM T cells produced significantly higher levels of IL2 (p < 0.05) compared to HER2-CAR/iCO-STIM T cells in the absence of CID or HER2-CAR.CD28.ζ T cells in co-culture assays with HER2+ tumor cells (MDA-HER2, LM7, A549). In contrast, HER2-negative tumor cells (MDA) did not induce IL2 production by HER2-CAR/iCO-STIM T cells +/- CID or HER2-CAR.CD28.ζ T cells. In repeat stimulation assays, HER2-CAR/iCO-STIM T cells showed robust antigen-dependent expansion in the presence of CID and were able to lyse HER2-positive LM7 cells after 4 re-stimulations compared to HER2-CAR.CD28.ζ T cells (p < 0.0001). *In vivo*, a low dose of HER2-CAR/iCO-STIM T cells (3x10^5^) + CID had superior antitumor activity in the metastatic LM7 osteosarcoma murine xenograft model compared to HER2-CAR/iCO-STIM T cells without CID or HER2-CAR.CD28.ζ T cells, resulting in a significant survival advantage (p < 0.001). In 3 mice that developed late recurrences after HER2-CAR/iCO-STIM T cell + CID therapy, a second dose of CID, given 100 days post T cell injection, eliminated 2/3 tumors. Superior antitumor activity of HER2-CAR/iCO-STIM T cell + CID therapy was confirmed in the HER2+ A549 lung cancer murine xenograft model.

**Conclusions**

CID-mediated activation of MyD88 and CD40 co-stimulatory signals in HER2-CAR T cells results in superior effector function compared to standard HER2-CAR T cells. Thus, expressing iCO-STIM molecules in CAR T cells has the potential to improve the efficacy of CAR T cell therapy approaches for solid tumors. In addition, our results indicate that the CID/iCO-STIM system will enable the ‘remote control’ of infused T cells.

### P30 In vivo administration of immune checkpoint inhibitors prior to tumor harvest enhances the function of tumor-infiltrating T lymphocytes expanded for adoptive T cell transfer

#### Mélissa Mathieu, Sandy Pelletier, John Stagg, Simon Turcotte

##### CRCHUM, Montréal, PQ, Canada

###### **Correspondence:** Mélissa Mathieu (melissamathieu911@gmail.com)

**Background**

T cell reactivity against mutated antigens, derived from cancer genomic alterations, is a key mediator of immunotherapy efficacy. Adoptive cell transfer (ACT) of mutation-reactive tumor-infiltrating T lymphocytes (TILs) however has been only effective in a minority of patients with metastatic gastrointestinal cancers. We hypothesize that the low frequency of mutation-reactive TILs and their exhaustion features may contribute to the lack of efficacy of TIL ACT, and that these factors can be overcome by *in vivo* administration of blocking antibodies against CTLA-4 or PD-1 prior to tumor harvest and TIL expansion.

**Methods**

We selected the mouse MC-38 colorectal cancer model to study CD8+ TIL reactive to 2 mutated, 2 self, and 1 retroviral antigens using co-cultures with peptide-pulsed splenocytes followed by IFN-γ intracellular staining. MC-38 cancer cells were inoculated subcutaneously and allowed to grow until 20mm^2^. Then anti-CTLA-4 (9H10) and/or anti-PD-1 (RPM1-14) antibodies were administered twice (4 days interval). Three days following the last antibody injection, tumors were harvested and analyzed for immune cell infiltration, phenotype and functionality. TILs were also expanded *in vitro* and characterized regarding the specificity and the functionality against the known antigens.

**Results**

The administration of anti-CTLA-4 prior to tumor harvest increased immune cell infiltration, decreased the proportion of myeloid derived suppressor cells and regulatory T cells in the tumors and enhanced CD8+ T cell IFN-γ and TNF-α production. Administration of both anti-CTLA-4 and anti-PD-1 was more effective to eliminate the tumor burden and recapitulated the effects observed following anti-CTLA-4 injection alone. Anti-CTLA-4 and/or anti-PD-1 alone did not appear to change the relative frequency of TIL reactive to mutated, self, or viral antigens but increased their polyfunctionality.

**Conclusions**

*In vivo* pre-conditioning of MC-38-bearing mice with immune checkpoint blocking antibodies may generate TILs more fit for ACT immunotherapy. Experiments are underway to compare the efficacy of this approach to conventional TIL ACT in this mouse model.

### P31 Redirecting gene-engineered T cells through covalent attachment of targeting ligands to a universal immune receptor

#### Nicholas Minutolo^1^, Prannda Sharma^1^, Andrew Tsourkas^2^, Daniel J Powell^1^

##### ^1^Department of Pathology and Laboratory Medicine, Perelman School of Medicine, University of Pennsylvania, Philadelphia, PA, USA; ^2^Department of Bioengineering, University of Pennsylvania, Philadelphia, PA, USA

###### **Correspondence:** Nicholas Minutolo (ngminutolo@gmail.com)

**Background**

Infusion of T cells genetically engineered to express a chimeric antigen receptor (CAR) is a promising approach for the treatment of certain cancers. Though CAR T cells are highly efficacious against CD19+ hematological malignancies, limitations exist in broadening their use. Conventional CAR T cells target a single tumor associated antigen (TAA), limiting their effectiveness against tumors with heterogeneous TAA expression as well as emerging antigen loss variants, as observed in CD19 CAR trials. Additionally, stably engineered CAR T cells may continually proliferate and activate in the presence of antigen, potentially causing fatal toxicity, without a method of elimination. To overcome these issues, we and others have developed a variety of universal immune receptors (UIRs) that allow for targeting of multiple TAAs with a single receptor-expressing T cell. Although these UIRs are a promising new technology, their reliance on noncovalent interactions between receptor and targeting ligand can lead to potential issues of affinity, specificity and activity.

**Methods**

Our UIR platform employs the use of the SpyCatcher and SpyTag proteins that, when combined, can form a covalent bond with high efficiency both *in vitro* and *in vivo*. Our SpyCatcher immune receptor is composed of an extracellular SpyCatcher domain attached to intracellular T cell signaling motifs in a lentiviral expression vector. Anti-TAA antibodies conjugated to SpyTag are used to confer redirected specificity to SpyCatcher expressing T cells. Measurements of T cell effector function include T cell cytokine secretion, activation, and targeted tumor cell lysis *in vitro*.

**Results**

In this study, we demonstrate the first universal immune receptor platform that allows for the endowment of function through post-translational covalent attachment of targeting ligands, securing their loading on the T cell surface while expanding recognition specificity. We demonstrate that the SpyCatcher immune receptor is expressed in primary human T cells and allows for specific T cell activation and cytokine secretion against plate bound SpyTag. Notably, in the presence of SpyTag-labeled targeting antibody, SpyCatcher T cells recognize and lyse antigen-expressing human tumor cells in a target-specific and dose-dependent fashion.

**Conclusions**

The SpyCatcher immune receptor is the first universal immune receptor designed for its capacity to covalently bind targeting ligands and redirect T cells against a diverse array of antigens, addressing current limitations of conventional CAR T cell therapy.

### P32 Simple automated manufacturing of gene engineered T cells under serum free conditions for adoptive T cell therapy

#### Nadine Mockel-Tenbrinck, Daniela Mauer, Katharina Drechsel, Carola Barth, Katharina Freese, Ulrike Kolrep, Silke Schult, Mario Assenmacher, Andrew Kaiser

##### Miltenyi Biotec, Bergisch Gladbach, Nordrhein-Westfalen, Germany

###### **Correspondence:** Nadine Mockel-Tenbrinck (nadinet@miltenyibiotec.de)

**Background**

Adoptive immunotherapy using gene-modified T cells redirected against cancer has proven clinical efficacy and tremendous potential in several medical fields. However, such personalized medicine faces several challenges in the complexity associated with the current clinical manufacturing methods. The processes are mostly suboptimal requiring cell manufacturers to deal with open steps, liquid handlings between devices used, manual interventions, removal of activation beads and often the use of reagents for which commercial availability is not in line with the high and increasing demand. GMP compliant human AB serum is one of such reagents. Therefore, developing improved methods to generate gene-engineered T cells suitable for clinical use that do not require serum are essential for the commercial scalability of such therapies.

**Methods**

We have developed a robust and reproducible automated manufacturing process for the lentiviral gene-modification and expansion of selected T cells. The CliniMACS Prodigy TCT (for T Cell Transduction) process software allows automated purification and polyclonal T cell stimulation followed by gene-modification and expansion of T cells in a single-use closed tubing set.

**Results**

Here we show that the TCT process enables the manufacturing of gene-modified T cells without the need for serum supplementation (human AB serum) when using TexMACS GMP Medium. Furthermore, implementation of a humanized recombinant activation reagent TransAct allows for a simplification of the process whereby the “bead removal” step is unnecessary. Comparable results from healthy donor or patients starting cells are demonstrated.

**Conclusions**

Taken together the TCT process in combination with the CliniMACS Prodigy and minimal user interactions enables the preparation of gene-modified T cells in serum free conditions. Process risks, due to use of different devices, unnecessary manipulations, or potential shortage of human AB serum availability can be minimized by automation of the entire process as developed and shown here. Overall, these improvements are meant to fully support commercial treatment of a large number of patients.

### P33 Tumor Infiltrating lymphocytes from soft tissue sarcoma have tumor-specific function

#### John Mullinax, MacLean Hall, Julie Le, Krithika Kodumudi, Erica Royster, Allison Richards, Ricardo Gonzalez, Amod Sarnaik, Shari Pilon-Thomas

##### H. Lee Moffitt Cancer Center, Tampa, FL, USA

###### **Correspondence:** John Mullinax (john.mullinax@moffitt.org)

**Background**

Adoptive Cell Transfer (ACT) using Tumor Infiltrating Lymphocytes (TIL) for unresectable metastatic melanoma results in a median overall survival of 52 months at our institution. This stands in contrast to the median overall survival for metastatic soft tissue sarcoma (mSTS) which is 12 months. The purpose of this report is to describe the phenotype and function of TIL from fresh surgical sarcoma specimens as a rationale for applying ACT to mSTS.

**Methods**

Fresh surgical sarcoma specimens were acquired under an IRB-approved protocol. Half of the specimen was digested and phenotyped by flow cytometry. The remaining half was plated as fragments for the isolation of TIL, which were expanded *in vitro* with conditions validated for melanoma-derived TIL. Cultured TIL were phenotyped by flow cytometry and propagated further with a rapid expansion protocol (REP). Tumor-specific reactivity was assessed by co-culture of TIL with autologous tumor or HLA-matched cell lines with measurement of IFN-gamma using ELISA.

**Results**

Sixteen patient-derived sarcoma specimens were collected. Histology of primary tumor included dedifferentiated liposarcoma (9), well-differentiated liposarcoma (2), undifferentiated pleomorphic sarcoma (2), and one each of gastrointestinal stromal tumor, myxofibrosarcoma and synovial sarcoma. Analysis of tumor digests indicated an average of 48% of cells from the lymphocyte gate were CD3+ (range 3.6%-76%). TIL were grown from all specimens, with TIL observed in 152 out of 192 (79%) fragments. The phenotype of the CD3+ subpopulations from TIL cultures included a median of 90.8% (range 2-99.9%) CD8+ and 2.3% (range 0-96.4%) CD4+ T cells. TIL were expanded in a REP to clinically meaningful numbers (mean 1385-fold) with no change in the CD8+ T cell proportion. Tumor-specific function of TIL generated from fragments was measured in two patients. There was tumor-specific IFN-gamma release (mean 148.8±13.5 pg/ml) in TIL co-cultured with HLA-matched cell lines and also in TIL co-cultured with autologous tumor digest (mean 259.9±14.7 pg/ml). The degree of IFN-gamma release was significantly greater when TIL were co-cultured with autologous digest compared to an HLA-mismatched cell line (p=0.0295).

**Conclusions**

CD3+CD8+ TIL can be isolated from human sarcoma tumors *in vitro*, expanded to meaningful numbers for therapeutic use, and demonstrate reactivity to the tumor from which they were cultured. These data form the basis for efforts to develop a clinical trial using ACT for patients with advanced sarcoma.

**Acknowledgements**

Work funded by a grant from the Chotiner Family Foundation.

### P34 Preclinical development of tumor infiltrating lymphocyte (TIL) based adoptive cell transfer (ACT) immunotherapy for patients with sarcoma

#### Morten Nielsen^1^, Anders Krarup-Hansen^2^, Dorrit Hovgaard^3^, Michael Mørk Petersen^3^, Anand Chainsukh Loya^4^, Niels Junker^5^, Inge Marie Svane^5^

##### ^1^Center for Cancer Immune Therapy and Department of Oncology, Herlev Hospital, Herlev, Hovedstaden, Denmark; ^2^Department of Oncology, Herlev University Hospital, Herlev, Hovedstaden, Denmark; ^3^Department of Orthopaedic Surgery, Copenhagen University Hospital, Copenhagen, Hovedstaden, Denmark; ^4^Department of Pathology, Copenhagen University Hospital, Copenhagen, Hovedstaden, Denmark; ^5^Department of Oncology, Center for Cancer Immune Therapy, Herlev University Hospital, Herlev, Hovedstaden, Denmark

###### **Correspondence:** Morten Nielsen (morten.nielsen.03@regionh.dk)

**Background**

ACT based on infusion of autologous TILs has the ability to induce complete and durable response in some patients with advanced malignant melanoma [1]. We believe that this approach could also be effective in sarcoma. In this preclinical study we are investigating feasibility of expanding TILs from sarcoma, as well as performing functional *in vitro* analyses on these TILs. This abstract is an update on our results so far.

**Methods**

A portion (>1 cm^3^) of the excised sarcoma tumor tissue is cut into fragments and placed in a growth medium containing IL-2 for initial TIL expansion. Afterwards, TIL cultures undergo a rapid expansion protocol (REP) expansion by adding OKT-3 and feeder cells. Phenotype and functional analyses are performed using flow cytometry and Elispot.

**Results**

To this date we were able to expand TILs from 18 of 20 tumor samples. TILs were harvested and frozen when an estimated number of 100x10^6^ to 200x10^6^ cells were reached. Mean expansion time was 32 days (16 - 61). 87.7% (36.4 – 99.1) of these cells were CD3+, and of these 66.7% (16.3 – 99.1) were CD4+, and 21.8 % (0.1 – 50.6) were CD8+. Most of the expanded TILs were effector memory subtype, while a smaller fraction was the more differentiated effector T cells. REP expansion rates ranged from 630 fold to 2.300 fold, and followed expansion pattern similar to TILs from malignant melanoma. TILs from 6 of 10 tested tumor samples with 4 different sarcoma subtypes (undifferentiated pleomorphic sarcoma, myxofibrosarcoma, myxoid liposarcoma and osteosarcoma) demonstrated reactivity against autologous tumor cells using Elispot. Further assessment is ongoing.

**Conclusions**

We were able to expand TILs from 90% of the included tumor samples to numbers needed for possible future clinical implementation. TILs were a mix of CD4+ and CD8+ with CD4+ being predominant. As of yet we have demonstrated TIL reactivity against autologous tumor cells from 6 of 10 tested patients. Thus, we conclude that it is feasible to translate TIL based ACT into clinical testing in sarcoma patients.

**References**

1. Rosenberg SA, Yang JC, Sherry RM, *et al*: **Durable complete responses in heavily pretreated patients with metastatic melanoma using T-cell transfer immunotherapy**. *Clin Cancer Res* 2011, **17(13)**:4550-4557.

### P35 Human natural killer cells engineered to express a chimeric NK activating receptor have activity against highly suppressive cells of the solid tumor microenvironment

#### Charlotte Rivas^1^, Robin Parihar^1^, Stephen Gottschalk^2^, Cliona M Rooney^1^

##### ^1^Baylor College of Medicine, Houston, TX, USA; ^2^Center for Cell and Gene Therapy, Baylor College of Medicine, Houston, TX, USA

###### **Correspondence:** Robin Parihar (rxpariha@txch.org)

**Background**

The suppressive microenvironment of solid tumors inhibits the anti-tumor activity of endogenous and chimeric antigen receptor (CAR)-bearing T cells, thereby limiting the efficacy of adoptive T cell therapies. Myeloid-derived suppressor cells (MDSCs; CD33^+^ CD11b^+^ HLA-DR^low^) and regulatory T cells (Tregs; CD4^+^ CD25^high^ FoxP3^+^ Helios^+^) contribute to the inhibitory tumor microenvironment (TME) through secretion of suppressive cytokines, expression of inhibitory ligands, and by promoting tumor neo-vascularization. NKG2D is an activating surface receptor expressed on natural killer (NK) cells, whose ligands are highly expressed by human Tregs and MDSCs. We genetically modified primary human NK cells to express a chimeric NKG2D molecule (ectodomain of endogenous NKG2D fused to the cytotoxic CD3-zeta chain; called NKG2D.z) in order to promote NK cell activation against Tregs and MDSCs. We hypothesized that NKG2D.z NK cells would exhibit enhanced cytolytic activity against suppressive autologous Tregs and MDSCs via the chimeric NKG2D, as well as secrete chemokines and cytokines that recruit and activate tumor-specific T cells. The objective of the current study was to compare the activity of NKG2D.z NK cells vs. non-transduced (NT)-NK cells on Tregs, MDSCs, or the combination.

**Methods**

We isolated MDSCs and Tregs from normal donors and patients with solid tumors, confirmed their phenotype by flow cytometry and their suppressive activity in T and NK cell proliferation assays, and used them as targets in NK cell cytotoxicity and co-culture assays.

**Results**

NT (endogenous) and mock-engineered (empty vector control) NK cells were unable to mediate cytotoxicity or release pro-inflammatory cytokines in response to autologous MDSCs or Tregs, either alone or in combination. In contrast, NKG2D.z NK cells exhibited enhanced cytolysis and secreted T cell-recruiting and -activating cytokines in response to both suppressive cell types alone. Further, when NKG2D.z NK cells were co-cultured in a highly suppressive environment containing both MDSCs and Tregs, their cytolytic and cytokine-secreting activities against either cell type were unimpaired. We have established an *in vivo* TME model comprising tumor cells with MDSCs and Tregs, and experiments testing the ability of NKG2D.z NK cells to eliminate suppressive cells and recruit endogenous or CAR-T cells *in vivo* are underway.

**Conclusions**

Our results suggest that our modified NK cells may reverse immune suppression by the TME and improve T cell-based immune therapies for solid tumors.

**Acknowledgements**

Authors thank Charles L. Sentman, PhD for initial use of the NKG2D.zeta sequence.

### P36 Cytokines induced by pre-B leukemia progression mediates irreversible T cell dysfunction

#### Haiying Qin^1^, Sang Nguyen^1^, Paul Su^1^, Chad Burk^1^, Brynn Duncan^1^, Bong-Hyun Kim^2^, M. Eric Kohler^1^, Terry Fry^1^

##### ^1^National Cancer Institute, NIH, Bethesda, MD, USA; ^2^Frederick National Laboratory for Cancer Research Leidos Biomedical Research, Inc., Frederick, MD, USA

###### **Correspondence:** Haiying Qin (qinh@mail.nih.gov)

**Background**

Acute lymphoblastic leukemia (ALL) is the most common childhood malignancy. The cure rate has reached up 90% with modern therapy, outcomes are still very poor for patients who relapse. Immunotherapy with donor lymphocyte infusions for ALL has not demonstrated the success seen in other hematologic malignancies, suggesting there are characteristics inherent to ALL that make it less amenable to detection and elimination by the immune system. T cell dysfunction in the setting of leukemia has been well described, but the mechanisms have not been fully elucidated. It is also unclear if this T cell dysfunction underlies some of the treatment failures seen in patients receiving adoptive therapy with chimeric antigen receptor (CAR) expressing T cells. Immune checkpoint blockade has led to advances in the treatment of many solid tumors, but it has yet to demonstrate similar success in ALL.

**Methods**

Using two preclinical models of pre-B cell ALL, we studied the negative impact of ALL progression on T cell function and the efficacy of CAR T cell therapy and immune checkpoint blockade. We also dissected the mechanism underlying the observed T cell dysfunction.

**Results**

Prophylactic vaccination protects mice against the murine pre-B ALL in a T cell-dependent manner. However, therapeutic vaccination is ineffective, even in the setting of low disease burden. Adoptive transfer of primed T cells from immunized donors can completely eradicate established leukemia; however, this efficacy is not seen with the adoptive transfer of T cells from leukemia-bearing mice. Expression of a CD19 CAR in T cells from leukemic mice fails to eradicate ALL, while the CAR T cells derived from naïve mice can. T cells from leukemic mice express markers of T cell dysfunction. The expression of these molecules was associated with elevated levels of IL6, TNFa and IL10 in the serum of leukemia-bearing mice. Incubation of naïve T cells in these cytokines *ex vivo* recapitulated the upregulation of exhaustion markers seen *in vivo*, suggesting these cytokines play a role in the observed T cell dysfunction. Blockade of PD-1, its ligand, PD-L1, or Tim3 were ineffective at reversing T cell dysfunction and preventing leukemia progressions *in vivo*, suggesting other mechanisms must be targeted to restore immune function in leukemia bearing hosts.

**Conclusions**

Cytokines induced by Pre-B Leukemia progression mediates irreversible T cell dysfunction. These findings underscore the need to elucidate the mechanisms of leukemia-induced immune suppression to fully optimize the use of CAR-expressing T cells in the treatment of ALL.

### P37 Identification of a recurrent high-affinity MHC class I restricted neoepitope in neuroblastoma using ProTECT

#### Arjun A. Rao^1^, Noam Teyssier^1^, Jacob Pfeil^1^, Nikolaos Sgourakis^1^, Sofie Salama^1^, David Haussler^2^

##### ^1^University of California, Santa Cruz, Santa Cruz, CA, USA; ^2^UC Santa Cruz Genomics Institute, University of California, Santa Cruz, Santa Cruz, CA, USA

###### **Correspondence:** Arjun A. Rao (aarao@ucsc.edu)

**Background**

T cells are trained to differentiate between cell-surface MHC-displayed peptide sequences from self- and non-self proteins and act on the latter. The numerous mutations often associated with cancers can occur in coding regions of the genome and modify the sequence of wild-type proteins, potentially creating targets for immunotherapies. We have developed an analysis pipeline ProTECT (Prediction of T cell Epitopes for Cancer Therapy) to identify and rank neo-epitopes in terms of immunogenicity. Running ProTECT on a set of recurrent neuroblastomas showed that recurrent tumors share neo-epitopes with their corresponding primary tumors suggesting that immunotherapies could provide long-term protection.

**Methods**

ProTECT accepts paired tumor and normal DNA sequencing fastq files, and tumor RNA sequencing fastqs. Mutations are called using a panel of callers, and are annotated to identify coding mutations. Prediction of self-MHC:mutated-peptides is carried out and the final binding predictions are ranked using an in-house algorithm. We support both MHCI and MHCII predictions.

**Results**

Running ProTECT on 6 recurrent neuroblastoma samples (NBL) from the TARGET (Therapeutically Applicable Research To Generate Effective Treatments) project revealed that the relapsed tumor inherits neo-epitopes predicted in the primary tumor. We also predicted neo-epitopes from 2 well-known hotspot mutations in NBL (NRAS Q61K and ALK R1275Q) that bind to common MHC alleles (HLA-A*01:01, HLA-A*03:01 and HLA-B*15:01). We carried out *in vitro* refolding and crystallization assays [1] for the five highest-ranking mutant NRAS and ALK predictions. Properly conformed MHC trimers were verified by a single, monodisperse peak after anion exchange chromatography and validated by SDS gel electrophoresis. Mass spec confirmed bound peptide for 4/5 tested predictions and 3 of these were used to set up hanging-drop crystallization trials in various conditions. Positive hits were obtained for one (MAQDIYRASY::HLA-B*15:01). ProTECT has also been run on a large subset of The Cancer Genome Atlas (TCGA). We aim to reveal clinically relevant hotspot-mutation:MHC pairs.

**Conclusions**

We have described a pipeline for identification and ranking of therapeutically relevant neo-epitopes. We have predicted potentially therapeutic targets for NBL that have been validated *in vitro*.

**References**

1. Garboczi DN, Hung DT, Wiley DC: **HLA-A2-peptide complexes: refolding and crystallization of molecules expressed in Escherichia coli and complexed with single antigenic peptides** . *PNAS* 1992, **89**:8.

### P38 A higher-affinity variant of a GD2-specific CAR significantly enhances potency *in vivo* and allows for a novel model of on-target off-tumor toxicity

#### Sarah A. Richman^1^, Selene Nunez-Cruz^2^, Zack Gershenson^2^, Zissimos Mourelatos^3^, David Barrett^1^, Stephan Grupp^1^, Michael Milone^3^

##### ^1^Children's Hospital of Philadelphia Division of Oncology, Philadelphia, PA, USA; ^2^University of Pennsylvania, Philadelphia, PA, USA; ^3^Department of Pathology and Laboratory Medicine, Division of Neuropathology, University of Pennsylvania, Philadelphia, PA, USA

###### **Correspondence:** Sarah A. Richman (richmans@email.chop.edu)

**Background**

As many potential targets of chimeric antigen receptor (CAR) T cell immunotherapy are self-antigens that are over-expressed in tumors but also present at lower levels on some normal tissue, understanding the nature of on-target off-tumor toxicity and how to overcome it is important in the development of new CAR T cell therapies. Preclinical modeling of such toxicity is complicated by the fact that most antigens are not shared between humans and mice, and strategies have largely relied on co-injection of antigen-low tumors or introduction of human antigen into mouse tissue by viral gene delivery. The GD2 tumor antigen would provide an excellent model in which to study on-target off-tumor toxicity as the exact glycolipid antigen is naturally shared between mice and humans. However, as of yet, GD2-specific CAR T cells have yielded modest efficacy and little toxicity in preclinical studies. Here we have engineered a higher potency GD2 CAR by introducing an affinity-enhancing mutation (E101_H_K), previously described by Horwacik et al., that we show significantly enhances CAR T cell activity and provides a model for toxicity.

**Methods**

Primary human T lymphocytes were transduced with lentivirus encoding either wild-type 14G2a-based anti-GD2 CAR, E101K mutant GD2 CAR, or a negative control CAR. After standard stimulation and expansion, T cells were analyzed for CAR surface expression by flow cytometry and for *in vitro* effector function by chromium release and IFN gamma ELISA. To evaluate *in vivo*, NOD-SCID-IL2Rγc-/- (NSG) mice were injected with the luciferase-expressing GD2-high human neuroblastoma cell line SY5Y, and four days later 3,000,000 CAR+ T cells were injected via tail vein. Tumor burden was measured using *in vivo* bioluminescence, and tumor and normal tissues were evaluated histologically by H&E staining and immunohistochemistry.

**Results**

The higher affinity mutant displayed comparable surface expression and T cell expansion but significantly enhanced GD2-specific cytotoxicity and cytokine release *in vitro* and tumor control *in vivo*. However, this enhanced efficacy was associated with severe CNS toxicity causing neurologic symptoms and death, and post-mortem evaluation of tissues revealed extensive CAR T cell infiltration into certain brain structures, particularly cerebellum, known to contain GD2.

**Conclusions**

The introduction of an affinity-enhancing mutation into the GD2-specific CAR dramatically increases CAR T cell potency and permits off-tumor CAR T cell activity in areas of the brain containing GD2. This scenario provides a new opportunity to investigate the mechanism of this toxicity and test strategies to achieve a therapeutic window.

### P39 Evaluating the potential of Müllerian inhibiting substance type II receptor (MISIIR) as a target for CAR T cell therapy against ovarian cancer

#### Alba Rodriguez-Garcia^1^, Matthew K Robinson^2^, Gregory P Adams^2^, Daniel J Powell^3^

##### ^1^University of Pennsylvania, Philadelphia, PA, USA; ^2^Fox Chase Cancer Center, Philadelphia, PA, USA; ^3^Department of Pathology and Laboratory Medicine, Perelman School of Medicine, University of Pennsylvania, Philadelphia, PA, USA

###### **Correspondence:** Alba Rodriguez-Garcia (albarod@mail.med.upenn.edu)

**Background**

Ovarian cancer is responsible for 5% of cancer-related deaths among women, and the majority of the cases are diagnosed at a late stage, accounting for a 5-year survival rate of 27%. Therefore, there is a dire need for effective therapies. The recent success of adoptive cell therapy using T cells engineered to express anti-CD19 chimeric antigen receptors (CARs) for the treatment of hematologic malignancies, rationalizes the development of similar strategies for solid tumors such as ovarian cancer. The achievement of safe, effective therapy requires the selection of a target antigen that is overexpressed in malignant cells but present in few to no normal cells. The Müllerian inhibiting substance type 2 receptor (MISIIR) is a member of the TGF-β receptors family involved in the regression of the primordial female reproductive tract in male embryos. This action is exerted through its interaction with soluble Müllerian inhibiting substance (MIS), triggering a downstream signaling cascade that induces apoptosis. MIS signaling through MISIIR has been shown to cause growth inhibition in ovarian, breast, prostate and endometrial cancer cell lines *in vitro*. In humans, MISIIR is expressed at very low levels in a restricted set of healthy tissues but is overexpressed in gynecologic cancers, including 69% of epithelial ovarian cancers, making it a candidate target antigen.

**Methods**

Here, we evaluate for the first time the potential of MISIIR as a target for CAR T cell therapy. In this work, we generated and functionally tested 5 distinct CARs comprised of different human MISIIR-specific single-chain antibody variable fragments (scFv) isolated from a phage display library coupled to the T cell signaling domains from CD27 and CD3Z.

**Results**

All the CARs were efficiently expressed primary human T cells transduced using recombinant lentivirus technology and showed specific binding and reactivity against recombinant MISIIR protein. Interestingly, when co-cultured with target cells engineered to overexpress MISIIR, just one of the CARs, GM7-27Z, showed specific reactivity in terms of cytolytic function and proinflammatory cytokines secretion. The activity of this CAR was further evaluated *in vitro* and *in vivo* in a panel of tumor cells lines expressing different levels of the target antigen.

**Conclusions**

Although the assessment of CAR-mediated antitumor activity and on-target off-tumor toxicity potential *in vivo* is required, the results obtained so far support the further exploration of an anti-MISIIR CAR-based therapy for the effective treatment of ovarian cancer as well as other gynecologic malignancies.

### P40 IL-2 in adoptive cell therapy–local production from an adenovirus vector instead of systemic administration results in safety and efficacy gains

#### João Santos^1^, Riikka Havunen^2^, Mikko Siurala^2^, Víctor Cervera-Carrascón^1^, Suvi Parviainen^1^, Marjukka Antilla^3^, Akseli Hemminki^2^

##### ^1^TILT Biotherapeutics, Helsinki, Uusimaa, Finland; ^2^University of Helsinki, Helsinki, Uusimaa, Finland; ^3^Finnish Food Safety Authority, Helsinki, Uusimaa, Finland

###### **Correspondence:** João Santos (joao@tiltbio.com)

**Background**

The use of interleukin-2 (IL-2) has been a major asset to boost the therapeutic anti-tumor efficacy of adoptive cell therapy, including tumor infiltrating lymphocyte (TIL) therapy in the context of solid tumors. However, clinical assessments have revealed that its systemic administration results in poor accumulation at solid tumor sites. Additionally, the half-life of this recombinant molecule is short. High dose administration has therefore been used but this results in severe adverse events, including mortality. Hence, local production at the tumor is an attractive concept which might retain or even increase the useful aspects of IL-2, while reducing systemic side effects.

**Methods**

We aimed to evaluate the efficacy and safety of, intratumoral delivered IL-2-armed adenoviruses combined with T cell transfer in rodents. Experiments were set up using the syngeneic CB57BL/6 mouse B16.OVA melanoma tumor model infused with OVA-specific T cells, and the syngeneic Syrian hamsters Hapt1 pancreatic tumor model infused with TILs. Both therapeutic schedules involved once-a-week intratumoral administration of replication deficient serotype 5 (mice) or oncolytic serotype 5/3 chimeric (hamsters) IL-2-armed adenoviruses comparing with weekly-continuous systemic administration of recombinant IL-2.

**Results**

In both models, local production of IL-2 successfully replaced that need for systemic IL-2. In fact, efficacy was even higher than with systemic IL-2. Furthermore, the vectored delivery of IL-2 significantly potentiated the infiltration of CD8+ T cells and, significantly decreased the percentage of regulatory T cells. In animals that received systemic recombinant IL-2 therapy, significant histological changes were observed in the lungs, liver, heart, spleen and kidneys that should be considered as side-effects of the treatment.

**Conclusions**

In summary, local production of IL-2 seems appealing from the point of view of efficacy and safety in the context of adoptive cell therapy. This preclinical assessment provides the rational for clinical translation, which is ongoing by TILT Biotherapeutics Ltd.

### P41 Successful expansion and characterization of tumor infiltrating lymphocytes (TILs) from non-melanoma tumors

#### Jyothi Sethuraman, Laurelis Santiago, Jie Qing Chen, Zhimin Dai, Seth Wardell, James Bender, Michael T Lotze

##### Lion Biotechnologies, Inc., Tampa, FL, USA

###### **Correspondence:** Jyothi Sethuraman (jyothi.sethuraman@lionbio.com)

**Background**

Adoptive cell therapy (ACT) has shown promise in comparison to other methods of cancer immunotherapy that rely on the active development of antitumor T cells *in vivo* to mediate cancer regression [1]. Administration of autologous TILs in melanoma patients has shown an overall response rate of 55% at NCI, 38% at Moffitt Cancer Center, 48% at MD Anderson Cancer Center and 40% in Sheba at the Ella Cancer Institute, Israel [1]. TILs have been found in a variety of solid tumors, and their presence has been shown to be a prognostic indicator of improved survival. Here, we demonstrate the feasibility of growing TILs and the potential to develop TIL therapies to treat other solid tumors, such as lung, breast, and bladder cancers.

**Methods**

Upon receiving surgically resected tumor specimens, samples were washed in HBSS and cut into small fragments prior to propagating *in vitro* in G-REX-10 cell culture flasks with interleukin-2 (commonly referred to as a pre-rapid expansion protocol [pre-REP]). After culture initiation, media was exchanged. The media was changed every 3 days subsequently for 2 weeks. TILs were harvested to assess cell count and viability, followed by immunophenotyping and cryopreservation.

**Results**

The average yield of TILs cultured and expanded from bladder, cervical, head & neck, lung, and triple-negative breast tumors is listed in Table 4. Phenotypic characterization of TILs from bladder, cervical and lung cancer were >60-70% CD8+ T cells whereas TILs from head and neck demonstrated variable distribution of CD8+ and CD4+ T cells. TILs propagated from TNBC were >80% CD4+ T cells. Regardless of the tumors, most cultures had < 20% CD56+ NK cells.

**Table 4:** **(Abstract P41).**

Tumor	Number of patient tumors	Average yield (and range) or TILs from pre-REP (10^6^)
Bladder	2	290 (97-600)
Cervical	4	360 (147-800)
H&N	7	539 (132-738)
Lung	8	688 (50-915)
TNBC	13	429 (81-665)

**Conclusions**

We have been successful in culturing and expanding TILs from various non-melanoma solid tumors. We will initiate REP from pre-REP TILs from non-melanoma tumors to enable product development for subsequent possible clinical trials. Efforts are currently focused on culturing TILs from smaller tumor specimens/biopsies to assess utility in promoting expansion of TILs with central and effector memory phenotypes and selecting for mutanome reactive TILs.

**References**

1. Rosenberg SA, Restifo NP: **Adoptive cell transfer as personalized immunotherapy for human cancer.** *Science* 2015, **348**:62-68.

### P42 A tumor-penetrating recombinant protein anti-EGFR-iRGD enhances efficacy of antigen-specific CTL in gastric cancer in vivo

#### Huizi Sha, Shu Su, Naiqing Ding, Baorui Liu

##### The Comprehensive Cancer Center of Drum-Tower Hospital, Medical School of Nanjing University & Clinical Cancer Institute of Nanjing University, Nanjing, Jiangsu, People’s Republic of China

###### **Correspondence:** Huizi Sha (shahuizinju@126.com)

**Background**

Strategies that enhance the function of T cells are critical for immunotherapy. Targeted delivery of T cells through BiTE (bispecific T cell engager) platform to cancerous tissues shows potential in sparing unaffected tissues. However, it has been a major challenge for cells penetration in solid tumor tissues due to the complicated tumor microenvironment. Activated T cells expression integrin, which is the target of peptide RGD. Peptide iRGD (CRGDK/RGPD/EC) increased vascular and tissue permeability in a tumor-specific and neuropilin-1-dependent manner, allowing co-administered drugs to penetrate into extravascular tumor tissue. Recombinant protein anti-EGFR-iRGD was purified and examined.

**Methods**

Recombinant protein anti-EGFR-iRGD consisting of an anti-EGFR VHH (the variable domain from the heavy chain of the antibody) fused to iRGD, a tumor-specific binding peptide with high permeability were expressed in *E. coli* BL21 (DE3) and purified by nickel-nitrilotriacetic acid affinity chromatography. We use tumor cell lines and mice to analyze the targeting, penetrating and antitumor activity of antigen-secific T cells together with recombinant protein.

**Results**

We have successfully constructed a recombinant protein named anti-EGFR-iRGD, a dual targets of EGFR and integrin and high permeable protein. It could spread extensively throughout both the multicellular spheroids and the tumor mass. The recombinant protein anti-EGFR-iRGD could help more T cells infiltrating into tumor mass and also exhibited antitumor activity in tumor cell lines and mice.

**Conclusions**

Our results provide impetus for further studies for potentially using iRGD based fusion protein anti-EGFR-iRGD with immune therapy regimens for enhancing therapy of gastric cancer patients.

**Fig. 32:**
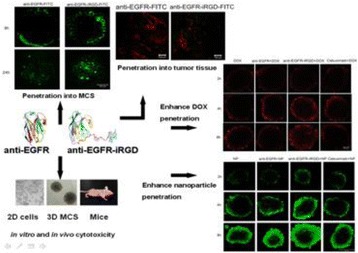
**(Abstract P42).** Purification and verification of recombinant protein

**Fig. 33:**
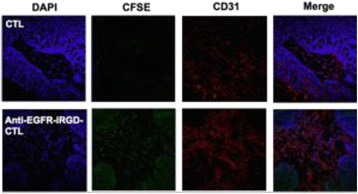
**(Abstract P42).** Penetration of CTL together with recombinant protein

### P43 Immunodominance of cancer neoantigen and cancer-germline antigen T cell reactivities in successful immunotherapy of virally-induced epithelial cancer

#### Sanja Stevanovic^1^, Anna Pasetto^2^, Sarah R Helman^1^, Jared J Gartner^2^, Todd D Prickett^2^, Paul F Robbins^2^, Steven A Rosenberg^2^, Christian S Hinrichs^1^

##### ^1^Experimental Transplantation and Immunology Branch, National Cancer Institute, National Institutes of Health, Bethesda, MD, USA; ^2^Surgery Branch, National Cancer Institute, National Institutes of Health, Bethesda, MD, USA

###### **Correspondence:** Sanja Stevanovic (sanja.stevanovic@nih.gov)

**Background**

Immunotherapy has clinical activity in human papillomavirus (HPV)-induced epithelial cancers, but the tumor antigens targeted by T cells resulting in cancer regression are poorly defined. The viral proteins expressed by these malignancies are generally considered the primary targets of T cell based immune attack. However, HPV+ cancers also harbor somatic mutations and express cancer-germline antigens that may be targets of tumor-specific T cells. Here, we aimed to elucidate the landscape of tumor antigens targeted by T cells in two patients with metastatic HPV+ cervical cancer who experienced durable complete tumor regression after adoptive transfer of tumor-infiltrating lymphocytes (TIL).

**Methods**

To this end, reactivity of therapeutic TIL was assessed in immunological assays against HPV-derived antigens (L1, L2, E1, E2, E4, E5, E6 and E7), mutated neoantigens and cancer-germline antigens identified by whole-exome and/or RNA sequencing of patient’s tumors. T cell receptor (TCR) clonotypes conferring specificity to tumor antigens were elucidated and quantified, and their *in vivo* persistence was profiled by TCR deep sequencing.

**Results**

T cell reactivity against the HPV-E6 and/or -E7 antigens was detected in both patient’s infused TIL, consistent with previously reported results. No T cell reactivity was detected against other six HPV-derived antigens. However, our data indicated that these patient’s infused TIL distinctly recognized mutated neoantigens or a cancer-germline antigen. Detailed TCR clonotype analysis showed that in one patient multiple CD8+ clonotypes (35% of infused TIL) recognized somatically mutated gene products (n=3) unique to patient’s tumor in addition to several CD8+ and/or CD4+ clonotypes (14% of TIL) targeting HPV-E6 and/or -E7. In the other patient, one CD8+ clonotype (67% of TIL) recognized the cancer-germline antigen Kita-kyushu lung cancer antigen 1 in addition to one CD4+ clonotype (14% of TIL) that targeted HPV-E7. Administered viral and non-viral tumor antigen-specific T cells in both patients remained functional and persisted at elevated levels in the circulation for months during ongoing remission.

**Conclusions**

Our data show that both patients who experienced complete tumor regression received TIL that contained low frequency of HPV-targeted T cells but a high frequency of mutated neoantigen- or cancer-germline antigen-targeted T cells. These results reveal a previously unappreciated role for T cells targeting non-viral antigens in HPV+ cervical cancer. By expanding the categories of potential tumor regression antigens for cervical cancer and possibly other HPV-induced malignancies, our findings provide new targets for personalized cancer vaccines and/or adoptive T cell therapies as well as for immune-monitoring of various cancer immunotherapies.

**Consent**

Written informed consent was obtained from patients.

### P44 Adoptive cellular therapy (ACT) with allogeneic activated natural killer (aNK) cells in patients with advanced Merkel cell carcinoma (MCC): preliminary results of a phase II trial

#### Shailender Bhatia^1^, Melissa Burgess^2^, Hui Zhang^3^, Tien Lee^4^, Hans Klingemann^4^, Patrick Soon-Shiong^4^, Paul Nghiem^1^, John M Kirkwood^5^

##### ^1^Fred Hutchinson Cancer Research Center, University of Washington, Seattle, WA, USA; ^2^University of Pittsburgh Cancer Institute, Pittsburgh, PA, USA; ^3^NantBioScience, Inc., Culver City, CA, USA; ^4^NantKwest, Inc., Culver City, CA, USA; ^5^UPMC Cancer Center, University of Pittsburgh Cancer Institute, Pittsburgh, PA, USA

###### **Correspondence:** Shailender Bhatia (sbhatia@uw.edu)

**Background**

MCC is an aggressive skin cancer associated with Merkel cell polyomavirus (MCPyV). Downregulation of class I major histocompatibility complex (MHC) expression in >80% of MCC tumors suggests potential susceptibility to adoptively transferred NK cells. aNK cells are derived from a NK cell line that is highly cytotoxic to a broad range of tumor cells. Phase I studies suggest that aNK cell therapy is well tolerated and has antitumor activity in patients with advanced hematologic or solid cancers. This study seeks to determine the efficacy of aNK cell therapy in patients with advanced MCC.

**Methods**

In this open-label phase II study, advanced MCC patients (planned N=24) receive aNK cells (2 × 10^9^ cells/m^2^) intravenously on two consecutive days (1 cycle) every 2 weeks. Key eligibility criteria include age ≥18 years, unresectable stage III or IV MCC, and ECOG performance status ≤2. Up to two prior systemic chemotherapies and/or immunotherapies are allowed. The study uses a Simon optimal two-stage design. The primary efficacy endpoint is 4-month progression-free survival (PFS) rate. Secondary endpoints include objective response rate by RECIST 1.1, time to progression, overall survival, safety, and quality of life assessment (FACT-G). Planned correlative studies include genomic and proteomic tumor profiles, MCPyV status, and immunohistochemical assessment of MHC-1, correlated to treatment outcome.

**Results**

As of August 2016, 3 patients have been enrolled. Treatment-related adverse events have been grade 2 or milder with no serious adverse events. The efficacy criterion for the first stage of the study has been met, with a patient with advanced MCC refractory to multiple prior therapies including PD-1 blockade demonstrating an impressive partial response (PR) with >70% regression, ongoing at 20 weeks. Intriguing changes were observed clinically in another patient’s superficial tumors just a few hours after aNK infusions, although this patient developed progressive disease at 4 weeks. Correlative studies on tumor biopsies of the patient with PR are ongoing.

**Conclusions**

ACT with allogeneic aNK cells has been safe and well tolerated in the initial three patients with advanced MCC. Encouraging antitumor activity has been observed with an impressive PR in a patient with advanced MCC refractory to PD-1 blockade. The pre-specified efficacy criterion for the first stage of the trial has been met and enrollment continues on the trial. Updated results will be presented at the meeting.

**Trial Registration**

ClinicalTrials.gov identifier NCT02465957.

### P45 Low dose conditioning chemotherapy and CD19-directed CAR T cells may elicit distinct immune programs associated with clinical responses

#### John M Rossi^1^, Marika Sherman^1^, Allen Xue^1^, Yueh-wei Shen^1^, Lynn Navale^1^, Steven A Rosenberg^2^, James N Kochenderfer^3^, Adrian Bot^1^

##### ^1^Kite Pharma, Inc, Santa Monica, CA, USA; ^2^Surgery Branch, National Cancer Institute, Bethesda, MD, USA; ^3^Experimental Transplantation and Immunology Branch, National Cancer Institute, Bethesda, MD, USA

###### **Correspondence:** Adrian Bot (abot@kitepharma.com)

**Background**

Anti-CD19 CAR T cell therapy has shown promising clinical efficacy. Recent evidence points to a critical role for non-myeloablative conditioning chemotherapy, influencing the expansion, persistence and activity of CAR T cells. To diminish toxicities, Kochenderfer, *et al.* pioneered a conditioning chemotherapy regimen with low dose cyclophosphamide (300-500mg/m^2^) and fludarabine (30mg/m^2^) administered daily for 3 days [1]. This resulted in a response rate of 73% in patients with advanced Non-Hodgkin lymphoma, with lower rate of hematologic toxicities. Post CAR T cell-treatment peak levels of cytokines in blood were associated with T cell expansion, clinical efficacy or neurotoxicity [1].

**Methods**

We analyzed 41 blood biomarkers in 22 patients treated with anti-CD19 CAR T cells, preceded by low dose conditioning chemotherapy. We also measured cytokine levels produced by CAR T cells *ex vivo*, upon culture with CD19-expressing target cells. The expansion of CAR T cells in blood was measured by quantitative PCR.

**Results**

Conditioning chemotherapy enhanced IL-15 and decreased lymphocytes and perforin blood levels. Several cytokines peaked sequentially in blood post CAR T cell infusion: among those, IL-15 and GM-CSF at days 2-3, followed by IL-10 and Granzyme B, at day 6. CAR T cell expansion in blood occurred within 7-14 days. IL-15 blood levels associated with T cell expansion, clinical response and toxicities. In addition, early post-CAR T cell treatment levels GM-CSF and peak blood levels of IL-10 and Granzyme B, were associated with clinical efficacy or neurotoxicity. When stimulated *ex vivo* with CD19-expressing cells, CAR T cells produced a broad range of molecules including GM-CSF, IL-10 and Granzyme B, but not IL-15.

**Conclusions**

In conclusion, these preliminary findings suggest that three immune programs impact clinical outcome of CAR T cell treatment: a T cell proliferative program initiated by conditioning chemotherapy, together with an inflammatory and a cytotoxic program deployed by CAR T cells. In addition, this analysis highlights the need to carefully optimize the conditioning chemotherapy regimen.

**Acknowledgements**

This study was conducted under a CRADA between NCI and Kite Pharma.

**Trial Registration**

ClinicalTrial.gov identifier NCT00924326.

**References**

1. Kochenderfer J, Somerville R, Lu T, Shi V, Yang JC, Sherry R, *et al*: **Anti-CD19 chimeric antigen receptor T cells preceded by low-dose chemotherapy to induce remissions of advanced lymphoma [abstract]**. *J Clin Oncol* 2016, **34 Suppl**:LBA3010.

### P46 Artificial antigen presenting cells promote expansion of tumor infiltrating lymphocytes (TILs)

#### Anandaraman Veerapathran, Aishwarya Gokuldass, Amanda Stramer, Jyothi Sethuraman, Michelle A Blaskovich, Doris Wiener, Ian Frank, Laurelis Santiago, Brian Rabinovich, Maria Fardis, James Bender, Michael T Lotze

##### Lion Biotechnologies, Inc., Tampa, FL, USA

###### **Correspondence:** Anandaraman Veerapathran (anand.veerapathran@lionbio.com)

**Background**

For more than a decade, allogeneic peripheral blood mononuclear cells (PBMC) have been used as accessory feeder cells that provide “costimulatory signals” necessary for the expansion of tumor-infiltrating lymphocytes (TILs) in the presence of IL-2 and CD3 stimulation (Rapid Expansion Protocol [REP]) [1]. The intrinsic heterogeneity of allogeneic PBMC is an important variable when considering the expansion and resulting phenotype of post-REP TIL prepared for transplantation. The procurement of allo-PBMC in large numbers is also challenging and expensive. Our objective was to evaluate artificial antigen presenting cells (aAPC) as a potential substitute for PBMC. As such, we developed a novel aAPC from the CD64^+^ MOLM-14 human leukemia cell line, genetically engineered to express recombinant CD86 (B7-2) and CD137-L (41BBL) (MOLM14-86/137).

**Methods**

The MOLM-14-86/137 cell line was generated via transduction of wild type MOLM-14 with lentiviral virions encoding genomic RNA of CD86 or CD137 downstream of the U3 promoter from MSCV. MOLM-14-86/137 was ɣ-irradiated at 100Gy and co-cultured with TILs at a 1:100 ratio (TIL:aAPC) in media containing OKT3 (30 ng/ml) and IL-2 (3000 IU/ml) for 14 days. On Day 14, we calculated their expansion and examined their differentiation (flow cytometry), metabolic rate, and cytotoxicity.

**Results**

**Fig. 34:**
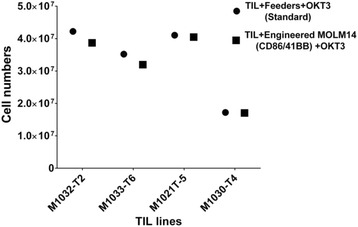
**(Abstract P46).** Rapid expansion of TILs using irradiated engineered MOLM-14 or PBMC feeders. TIL were co-cultured with PBMC Feeders or MOLM14 (CD86/41BBL) at 1:100 ratios plus OKT3(30ng/ml) and IL-2 (3000 IU/ml). Cells were counted and split on Day 6 and 11. Each dot represents cell numbers determined on Day 14

Compared to TIL co-cultured with PBMC or wild type MOLM-14, we obtained 95-100% TILs via co-culture with MOLM-14-86/137. This value was within the expected range using PBMC (Fig. 34). TIL differentiation, cellular respiration (OXPHOS) and redirected cytotoxicity were also within the range expected via co-culture with PBMC.

**Conclusions**

According to our measurements, co-culture of TIL with MOLM-14-86/137 aAPC resulted in expansion, metabolic activity and cytotoxicity that were sufficiently similar to that obtained with PBMC. This data suggests that investigation of a MOLM-14-86/137 based REP protocol in a clinical setting is warranted.

**References**

1. Dudley ME, Wunderlich JR, Shelton TE, Even J, Rosenberg SA: **Generation of tumor-infiltrating lymphocyte cultures for use in adoptive transfer therapy for melanoma patients**. *J Immunother* 2003, **26**:332–342.

### P47 Blocking vasoactive intestinal peptide signaling modulates immune checkpoints and graft-versus-leukemia in allogeneic transplantation in mice

#### Edmund K Waller^1^, Jian-Ming Li^1^, Christopher Petersen^1^, Bruce R Blazar^2^, Jingxia Li^1^, Cynthia R Giver^1^

##### ^1^Emory University, Atlanta, GA, USA; ^2^University of Minnesota, Minneapolis, MN, USA

###### **Correspondence:** Edmund K Waller (ewaller@emory.edu)

**Background**

The goal of allogeneic bone marrow transplantation (allo-BMT) is the elimination of leukemia cells through the graft-versus-leukemia (GvL) activity of donor cells, while limiting graft-versus-host disease (GvHD). Immune checkpoint pathways regulate GvL and GvHD activities, but blocking these pathways can cause lethal GVHD. Vasoactive intestinal peptide (VIP) is an immunosuppressive neuropeptide that regulates co-inhibitory pathways.

**Methods**

Murine models of MHC-mismatched allogeneic bone marrow transplantation were used to evaluate the effect of blocking VIP-signaling on the graft-versus-leukemia (GvL) and graft-versus host disease (GvHD) effect of donor T cells. Mice were transplanted with donor grafts from VIP-KO mice or recipients of wild-type grafts were treated with seven daily injections of a peptide antagonist to VIP (VIPhyb). Survival, GvHD and the growth of luciferase+ LBRM, a T cell lymphoblastic leukemia cell line, or C1498, a myeloid leukemia cell line, were monitored by bio-luminescent imaging. Expression of chemokine receptors, cytokines and check-point molecules were measured by flow cytometry. VIP expression on donor and host cells was visualized using a transgenic mouse in which GFP expression is driven by the VIP promoter. T cell repertoire from T cells in mice with GvHD or GvL was analyzed by deep sequencing.

**Results**

**Fig. 35:**
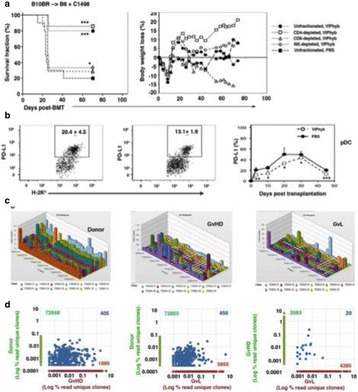
**(Abstract P47).** Treatment of allo-BMT recipients with a VIP antagonist induces a CD8+ donor T cell-dependent GvL response associated with down-regulation of PD-L1 on donor pDC and expansion of oligoclonal donor CD8+ T cells. A: Survival curves and GVHD clinical scores for B10.BR-->B6 allo-BMT harboring C1498 leukemia cells. Donor T cell subsets were depleted of specific populations by MACS prior to transplantation as shown. B: Expression of PD-L1 on donor pDC in B6-->B10.BR transplants. C: TCR Vbeta and J gene segments present in donorCD8+ T cells (left), CD8+ T cells from mice with GVHD (middle) and Cd8+ T cells from mice with GvL (right). D: Lack of correspondence between TCR Vbeta and J genes sequenced in mice with GvHD and mice with GVL

VIP is expressed transiently in donor NK, NKT, dendritic cells, and T cells after allo-transplant, as well as in host leukocytes. A peptide antagonist of VIP signaling (VIPhyb) increased T cell proliferation *in vitro* and reduced IL-10 expression in donor T cells. Treatment of allo-BMT recipients with VIPhyb, or transplanting donor grafts lacking VIP (VIP-KO), activated donor T cells in lymphoid organs, reduced T cell homing to GvHD target organs, and enhanced GvL without increasing GvHD in multiple allo-BMT models. Genetic or *ex vivo* depletion of donor NK cells or CD8+ T cells from allografts abrogated the VIPhyb-enhanced GvL activity (Fig. 35A). VIPhyb treatment led to downregulation of PD-1 and PD-L1 expression on donor immune cells (Fig. 35B), increased effector molecule expression, and expanded oligoclonal CD8+ T cells that protected secondary allo-transplant recipients from leukemia (Fig. 35C & D).

**Conclusions**

VIP production by donor immune cells is dynamically regulated after allo-BMT, and transplanting VIP-KO cells, or daily treatment with VIPhyb, significantly enhanced survival of leukemia-bearing transplant recipients via a CD8+ T cell dependent GvL effect without increased GvHD in murine models of MHC mis-matched allo-BMT. Blocking VIP-signaling thus represents a novel pharmacological approach to separate GvL from GvHD and enhance adaptive T cell responses to leukemia-associated antigens in allo-BMT.

### P48 Bortezomib sensitizes cancer stem cells from solid human tumors to natural killer cell-mediated killing

#### Ziming Wang^1^, Steven K Grossenbacher^1^, Ian Sturgill^2^, Robert J Canter^1^, William J Murphy^1^

##### ^1^University of California, Davis, Sacramento, CA, USA; ^2^California State University, Sacramento, Sacramento, CA, USA

###### **Correspondence:** Ziming Wang (zswang@ucdavis.edu)

**Background**

Cancer stem cells (CSCs) from solid and hematopoietic tumors resist conventional cytotoxic therapies that target rapidly proliferating cells. Thus, residual CSCs can hide within the tumor niche and seed relapse and metastasis. Due to their relapse potential there is an urgent need to identify ways to therapeutically target CSCs. We previously found that cells expressing high amounts of the stem cell associated protein aldehyde dehydrogenase (ALDH) are effectively killed by activated natural killer (NK) lymphocytes. NK cells are known to kill malignant cells though apoptotic processes inherent to the target cell, such as TRAIL-DR5 or Fas-FasL binding, without prior immunization. We and others have also found that the FDA approved proteasome inhibitor, bortezomib, sensitizes tumor cells to NK cell killing by upregulating DR5 and intracellular machinery associated with apoptosis. Based on this previous work, we investigated the effects of bortezomib to promote NK cell killing of ALDH^bright^ CSCs. We evaluated CSCs derived from solid tumors, *in vitro* and *in vivo*, for the induction of receptors associated with NK cell mediated killing, and for their susceptibility to NK killing after treatment.

**Methods**

*In vitro* sensitization and cytotoxicity assays were performed using cultured NK cells isolated from human peripheral blood. The glioblastoma and sarcoma cell lines, U87 and A673, respectively, were first treated with bortezomib, then co-cultured with activated NK cells at serial effector:target ratios. Target tumor cells were analyzed using flow cytometry for cell survival, and expression of Fas, DR4, DR5, and MICA/B on both ALDH^bright^ and ALDH^dim^ cells.

**Results**

Bortezomib led to a 3-fold increase in the percentage of viable ALDH^bright^ glioblastoma and sarcoma cells, *in vitro*, compared to untreated controls. Bortezomib enhanced the expression of Fas and DR5 by 10% and 40%, respectively, in ALDH^bright^ U87 cells. It increased the expression of DR4 by 20% in ALDH^bright^ A673 cells. However, bortezomib had little effect on ALDH^dim^ cells. Bortezomib pretreatment lead to a 98% decrease in viable ALDH^bright^ cells following NK cytotoxicity assays *in vitro. In vivo*, bortezomib improved the efficacy of adoptive NK cell therapy in multiple mouse xenograft models.

**Conclusions**

ALDH^bright^ CSCs are resistant to the cytotoxic effects of bortezomib. Bortezomib resistance is marked by increases in the expression of Fas, DR4, and DR5 and leads to increased susceptibility to lysis by activated NK cells. The combined use of bortezomib with activated natural killer cells could act as a potential anti-CSC strategy to improve outcomes for patients with solid tumors.

### P49 Targeted NK cells display potent activity against glioblastoma and induce protective antitumor immunity

#### Congcong Zhang^1^, Michael C Burger^2^, Lukas Jennewein^3^, Anja Waldmann^1^, Michel Mittelbronn^3^, Torsten Tonn^4^, Joachim P Steinbach^2^, Winfried S Wels^1^

##### ^1^Georg-Speyer-Haus, Institute for Tumor Biology and Experimental Therapy, Frankfurt, Germany; ^2^Institute for Neurooncology, Goethe University, Frankfurt, Germany; ^3^Edinger Institute, Goethe University, Frankfurt, Frankfurt, Germany; ^4^German Red Cross Blood Donation Service North-East, Dresden, Dresden, Germany

###### **Correspondence:** Winfried S Wels (wels@gsh.uni-frankfurt.de)

**Background**

Significant progress has been made over the last decade towards realizing the potential of natural killer (NK) cells for cancer immunotherapy. In addition to donor-derived primary NK cells, also continuously expanding cytotoxic cell lines such as NK-92 are being considered for adoptive cancer immunotherapy. High cytotoxicity of NK-92 has previously been shown against malignant cells of hematologic origin in preclinical studies, and general safety of infusion of NK-92 cells has been established in phase I clinical trials. To enhance their therapeutic utility, here we genetically modified NK-92 cells to express a chimeric antigen receptor (CAR), consisting of an ErbB2 (HER2)-specific scFv antibody fragment fused via a linker to a composite CD28-CD3ζ signaling domain. GMP-compliant protocols for vector production, lentiviral transduction and expansion of a genetically modified NK-92 single cell clone (NK-92/5.28.z) were established.

**Methods**

Functional analysis of NK-92/5.28.z cells revealed high and stable CAR expression, and selective cytotoxicity against ErbB2-expressing but otherwise NK-resistant tumor cells of different origins *in vitro*. Ongoing work focuses on the development of these cells for adoptive immunotherapy of ErbB2-positive glioblastoma (GBM). ErbB2 expression in primary tumors and cell cultures was assessed by immunohistochemistry and flow cytometry. Cell killing activity of NK-92/5.28.z cells was analyzed in *in vitro* cytotoxicity assays. *In vivo* antitumor activity was evaluated in NOD-SCID IL2Rγnull (NSG) mice carrying orthotopic human GBM xenografts and C57BL/6 mice carrying orthotopic ErbB2-expressing murine GBM tumors.

**Results**

We found elevated ErbB2 protein expression in >40% of primary GBM samples and in the majority of GBM cell lines investigated. In *in vitro* assays, NK-92/5.28.z in contrast to untargeted NK-92 cells lysed all ErbB2-positive established and primary GBM cells analyzed. Potent *in vivo* antitumor activity of NK-92/5.28.z was observed in orthotopic GBM xenograft models in NSG mice, leading to a marked extension of symptom-free survival upon repeated stereotactic injection of CAR NK cells into the tumor area. In immunocompetent mice, local therapy with NK-92/5.28.z cells resulted in cures of transplanted syngeneic GBM in the majority of animals, induction of endogenous antitumor immunity and long-term protection against tumor rechallenge at distant sites.

**Conclusions**

Our data demonstrate the potential of ErbB2-specific NK-92/5.28.z cells for adoptive immunotherapy of glioblastoma, justifying evaluation of this approach for the treatment of ErbB2-positive GBM in clinical studies.

### P50 Shared T cell receptor sequences between HLA-A2+ patients vaccinated against a Melan-A epitope correlate with clinical benefit

#### Jason B Williams^1^, Yuanyuan Zha^1^, Thomas F Gajewski^2^

##### ^1^University of Chicago, Chicago, IL, USA; ^2^University of Chicago Medical Center, Chicago, IL, USA

###### **Correspondence:** Jason B Williams (jaybwilliams1@uchicago.edu)

**Background**

Adoptive T Cell Therapy (ACT) of *in vitro* expanded T cell clones or transduced T cells redirected against defined tumor antigens has shown therapeutic efficacy in some patients. Ideas to improve upon this therapy are multifaceted, including combining ACT with checkpoint blockade, increasing the number of defined T cell receptors (TCR) against defined antigens, identifying new tumor-specific somatic mutations to target, and engineering TCRs to have increased avidity. However, even for well characterized antigens such as Melan-A, the optimal TCR is not known. Some engineered TCRs have shown off-tumor toxicity, and so selecting TCRs with maximal therapeutic efficacy but at the same time giving minimal side effects remains an important goal.

**Methods**

We reasoned that one strategy for selecting optimal TCRs might be to identify T cells expanded after active immunization against defined epitopes in patients who experienced clinical benefit but no apparent side effects. To this end, we performed deep TCR sequencing of HLA-2/Melan-A^+^ CD8^+^ T cells from 16 metastatic melanoma patients vaccinated against a Melan-A epitope.

**Results**

While changes in overall TCR clonality measured before and after vaccination did not correlate with clinical benefit, many TCRs showed a significant increase in representation of the total TCR repertoire after vaccination. Of the 6 patients that received a clinical benefit we found 122 public TCRβ and 124 public TCRα sequences. 105 of these sequences showed expansion after vaccination in 2 or more patients. Surprisingly, we did not observe the defined Melan-A-specific TCRs used previously in redirected ACT clinical trials, designated DMF4 and DMF5. Mapping of public sequences by frequency per patient and aligning TCRα/TCRβ sequences highlighted several potential TCRα/TCRβ pairings. One patient was of particular interest as he had participated in two vaccine trials, with a 32-month interim between trials and clinical benefit each time. By the end of the second treatment period, the patient’s TCR repertoire contained 55 public sequences. Interestingly, 7 of these sequences showed an initial contraction at the end of the first trial followed by a significant expansion by the end of the second trial, suggesting a strong clonotypic response to Melan-A.

**Conclusions**

Together, these data highlight multiple TCRα and TCRβ sequences correlating with clinical benefit in the setting of no treatment-related toxicities. Similar results have been observed in other trials utilizing CEA peptide or WT1 peptide immunization. These sequences should enable full-length cloning of TCRs to be used in redirect adoptive cell therapy.

**Trial Registration**

ClinicalTrials.gov identifier NCT00515528.

### P51 T cells redirected to TEM8 have antitumor activity but induce ‘on target/off cancer toxicity’ in preclinical models

#### LaTerrica C. Williams^1^, Giedre Krenciute^1^, Mamta Kalra^1^, Chrystal Louis^1^, Stephen Gottschalk^2^

##### ^1^Baylor College of Medicine, Houston, TX, USA; ^2^Center for Cell and Gene Therapy, Baylor College of Medicine, Houston, TX, USA

###### **Correspondence:** LaTerrica C. Williams (laterriw@bcm.edu)

**Background**

Targeting the tumor vasculature holds promise to improve the outcome for patients with refractory solid tumors. Tumor endothelial marker (TEM) 8 is an attractive target for T cell therapies since it is expressed at higher levels in malignant cells and the tumor endothelium as judged by studies using monoclonal antibodies (mAbs). However T cells do not require high expression of the targeted antigen for activation, because of the higher overall avidity of a multivalent T cell compared to bivalent mAbs. Thus, the aim of this project was to determine the safety and antitumor activity of T cells expressing TEM8/CD3-specifc T cell engagers (TEM8-ENG).

**Methods**

qPCR and FACS analysis was used to determine the expression of TEM8 in solid tumor and endothelial cells. TEM8-ENG T cells were generated by transducing T cells with a retroviral vector encoding a TEM8-ENG consisting of the TEM8-specific scFv L2 linked to a scFv recognizing CD3. TEM8-ENG T cell effector function was evaluated *in vitro* and *in vivo*. Appropriate controls were used including ENG T cells specific for an irrelevant antigen (CD19).

**Results**

To confirm the specificity of TEM8-ENG T cells we used targets that did not express TEM8 (BV173) or BV173 cells that were genetically modified to express human TEM8, murine TEM8, or murine TEM1. TEM8-ENG T cells recognized TEM8^pos^ targets (BV173.hTEM8, BV173.mTEM8) as judged by their ability to secrete IFNγ in coculture assays and kill both targets in cytotoxicity assays; in contrast, TEM8^neg^ cells (BV173, BV173.mTEM1) were not recognized. Specificity of TEM8-ENG T cells was further confirmed with TEM8^pos^ U373 glioma cells and U373.k/oTEM8 cells. TEM8-ENG T cells recognized a panel of TEM8^pos^ solid tumor cells (A431, A549, LM7, LAN1, U87), and primary endothelial cells (HHSEC, HPAEC) in contrast to TEM8^neg^ tumor cells (KG1a, Daudi). *In vivo*, intratumoral administration of TEM8-ENG T cells induced regression of U373 gliomas in an orthoptic xenograft model. Intravenous administration of 1x10^7^ TEM8-ENG T cells resulted in antigen-dependent expansion and death of 7/10 mice; no toxicity was observed after the injection of 1x10^6^ TEM8-ENG T cells.

**Conclusions**

TEM8 is expressed in tumor endothelium, normal endothelial cells and solid tumor cells as judged by qPCR, FACS, and functional assays. TEM8-ENG T cells had antitumor activity *in vivo*, but displayed dose-dependent toxicity. Our studies highlight that mAbs and T cells may have different toxicity profiles, most likely due to differences in their avidity for the targeted antigen. TEM8-ENG T cell xenograft models represent an ideal model to study genetic approaches to prevent ‘on target/off cancer toxicities’ of cell therapies.

### P52 A pathogen boosted adoptive cell transfer immunotherapy to treat solid tumors

#### Gang Xin^1^, David Schauder^1^, Aimin Jiang^2^, Nikhil Joshi^3^, Weiguo Cui^1^

##### ^1^Blood Center of Wisconsin, Milwaukee, WI, USA; ^2^Department of Immunology, Roswell Park Cancer Institute, Buffalo, NY, USA; ^3^Koch Institute for Integrative Cancer Research and Department of Biology, Massachusetts Institute of Technology, Cambridge, MA, USA

###### **Correspondence:** Gang Xin (Gang.Xin@BCW.edu)

**Background**

Despite the remarkable success in treating hematological malignancies, adoptive cell transfer (ACT) still faces several challenges in treating solid tumors. The main stumbling blocks include insufficient quantity of tumor-specific T cells for transfer, impaired migration of transferred T cells into the tumor and the immunosuppressive microenvironment within the tumor.

**Methods**

To overcome these problems, we designed an innovative approach that not only overcomes immunosuppression, but also induces robust anti-tumor T cell responses in the tumor. We first genetically engineered dual-specific CD8 T cells that can recognize both a tumor associated antigen and a bacterial antigen *in vitro*. Then, we treated tumor-bearing mice with ACT using a small number of the dual-specific CD8 T cells. This was accompanied by intratumoral injection of a low dose of the bacteria, which was sufficient to break local immunosuppression.

**Results**

The dual-specific CD8 T cells expanded robustly and migrated to the tumor bed in response to the infection. At the same time, the second TCR of these effector CD8 T cells recognized tumor antigen and executed effector function, causing tumor regression. As a result of this enhanced anti-tumor effect, 60% of the treated mice successfully eradicated their solid tumor at the primary site.

**Conclusions**

Our approach not only overcomes immunosuppression, but also recruits robust anti-tumor T cell responses to the tumor. Overall, our study harnesses the power of multiple arms of the immune system with promising translational value, which can be used to target many types of solid tumors.

### P53 Pharmacologic rejuvenation of exhausted T cells to improve adoptive TIL therapy

#### Xue Zeng^1^, Ashley V Menk^1^, Nicole Scharping^2^, Greg M Delgoffe^2^

##### ^1^University of Pittsburgh Cancer Institute, Pittsburgh, PA, USA; ^2^University of Pittsburgh, Pittsburgh, PA, USA

###### **Correspondence:** Xue Zeng (xuz27@pitt.edu)

**Background**

Immunotherapy has emerged as a strategy for the treatment of cancer. One of these immunotherapies is adoptive tumor-infiltrating lymphocyte (TIL) therapy, in which T cells from resected tumors are expanded *in vitro* and then given to patients. However adoptive TIL therapy has little efficacy for many patients, because the tumor microenvironment creates an extreme environment for T cells. Our lab has revealed that T cells display metabolic defects, especially a loss of mitochondria, when they infiltrate the tumor microenvironment. This loss is related to T cell exhaustion. We hypothesize that these exhausted T cells were the most functional cells as they responded to tumor earliest and strongest. However their loss of mitochondria prevents them from further expansion when removed and cultured *in vitro*. Thus, we are utilizing what we have identified about their metabolic dysfunction to rejuvenate those T cells during *ex vivo* expansion. Our goal is to make exhausted T cells more metabolically active and provide a potent method for TIL therapy.

**Methods**

Tumor injection: mice were given either 250,000 B16 or MC38 tumor cells injected intradermally in the center of the back. T cell activation: TILs are activated with 3 ug/ml anti-CD3 (plate bound), 2 ug/ml anti-CD28, 50 units/ml IL-2. Adoptive TIL transfer: treated and non-treated TIL are given to the mice that bear tumors by intravascular injection.

**Results**

PD-1^hi^ cells remain mitochondrially deficient and fail to proliferate *ex vivo*. Rosiglitazone can rescue mitochondrial mass and proliferation. PD-1 Tim-3^hi^ cells are over proliferated by PD-1 Tim-3^lo^ cells. Preliminary data has shown that glitazone compounds to long-term expansion protocols prevents loss of the previously-exhausted T cells during expansion.

**Conclusions**

Cells expressing high levels of PD-1 and Tim-3 have low mitochondrial mass and fail to proliferate effectively *in vitro*. Mixing congenically marked cells from the non-exhausted or exhausted compartment shows exhausted cells are quickly overtaken by the non-exhausted (less than 1 week). Adding glitazone compounds to stimulate mitochondrial biogenesis results in short-term improvement of T cell proliferation *in vitro*. Preliminary data has shown that glitazone compounds to long-term expansion protocols prevents loss of the previously-exhausted T cells during expansion.

**Acknowledgements**

UPCI Cytometry Core and Animal Facility (supported by NCI P30CA047904), University of Pittsburgh Department of Immunology, University of Pittsburgh Cancer Institute, Tumor Microenvironment Center, Chinese Scholar Council.

**References**

1. Delgoffe GM, Powell JD: **Feeding an army: The metabolism of T cells in activation, anergy, and exhaustion.** *Mol Immunol* 2015, **68(2 Pt C)**:492–496.

### P54 A turbocharged chimeric antigen receptor against prostate cancer

#### Zeguo Zhao^1^, Mohamad Hamieh^1^, Justin Eyquem^1^, Gertrude Gunset^1^, Neil Bander^2^, Michel Sadelain^1^

##### ^1^Center for Cell Engineering, Memorial Sloan Kettering Cancer Center, New York, NY, USA; ^2^Department of Urology, Weill Cornell Medical College, New York, NY, USA

###### **Correspondence:** Zeguo Zhao (zhaoz@mskcc.org)

**Background**

Both CD28- and 4-1BB-based second-generation CAR T cells elicit dramatic clinical responses in patients with refractory/relapsed CD19 positive malignancies, especially patients with acute lymphoblastic leukemia. We recently demonstrated that co-expressing the second-generation 19-28z CAR with 4-1BBL yields balanced tumoricidal function and T cell persistence, resulting in the greater therapeutic efficacy (Turbocharged CAR). However, due in part to their tumor microenvironment, solid tumors often resist CAR T cell therapy. We hypothesized that CD28-based second-generation CAR T cells coexpressing 4-1BBL would have better therapeutic efficacy against solid tumors than current second-generation CARs, owning to their unique intrinsic and immunomodulatory qualities.

**Methods**

Prostate-specific membrane antigen (PSMA) is a dimeric type II integral membrane glycoprotein, which is overexpressed in castrate-resistant, metastatic prostate cancer. We constructed a tricistronic PSMA-targeted CAR vector encoding the Pd28z CAR, 4-1BBL and a truncated, nonfunctional EGFR as a safety control (Pd28z–4-1BBL–EGFRt). Two second-generation CARs (Pd28z and PdBBz) served as controls.

**Results**

In a high tumor burden model of disseminated prostate cancer, we used the *in vivo* “stress test” in which the T cell dose is gradually lowered lowered to levels where CAR therapy begins to fail, in order to compare the relative functionality and persistence of these CAR T cells. CAR T cells coexpressing Pd28z with 4-1BBL exhibited higher tumor eradication and T cell persistence in NSG mice bearing diffuse metastatic prostate cancer, compared to both second-generation CARs Pd28z and PdBBz.

**Conclusions**

4-1BBL Turbocharged CAR T cells thus seem to possess striking therapeutic potential against solid tumors.

## Biomarkers and Immune Monitoring

### P55 Map of targets on dendritic cells (DC) in human tonsils and lymph nodes potentially facilitating antigen cross-presentation

#### David Askmyr^1^, Milad Abolhalaj^2^, Kristina Lundberg^2^, Lennart Greiff^3^, Malin Lindstedt^2^

##### ^1^ENT Departement, Lund University Hospital, Lund, Skane Lan, Sweden; ^2^Department of Immunotechnology, Lund University, Lund, Skane Lan, Sweden; ^3^ENT Department, Skåne University Hospital, Lund, Lund, Skane Lan, Sweden

###### **Correspondence:** Milad Abolhalaj (milad.abolhalaj@immun.lth.se)

**Background**

Dendritic cells (DCs) orchestrate adaptive and innate immune responses and are therefore key targets for immunotherapy (IT). Of special interest for IT directed against cancer are DCs with a high capacity of antigen cross-presentation, potentially resulting in cell-mediated tumor antigen-specific effects. This study reports an update on the map of DC subtypes and phenotypical aspects that may be reached by adjuvant measures in human tonsils and lymph nodes, both potential sites for “vaccine” deposition.

**Methods**

From biopsies of tonsils (n=23) and neck lymph nodes (n=16), single cell suspensions were prepared by enzymatic digestion and DCs were identified by an 8-color flow cytometry Ab panel. DC subsets frequencies (CD1c^+^, CD123^+^, CD141^+^, CD1c^-^ CD141^-^), maturity status, and surface receptor profiles, focusing on a variety of C-type lectin receptors. Toll-like receptor 2 (TLR2) and the chemokine receptor XCR1, were then described and investigated at protein level via flow cytometry. The results were then analyzed through two-group comparison tests.

**Results**

DCs with similar myeloid CD11c^+^/plasmacytoid CD123^+^ ratios and largely similar frequencies among CD45^+^ leukocytes were observed in tonsils as well as lymph nodes. However, among the DC subsets studied, CD141^+^ DCs showed a higher frequency in tonsils compared to lymph nodes. No maturity differences were found among the DC subsets in tonsils and lymph nodes based on expression of CD80 and CD86. DC subsets expressing XCR1, TLR2, CLECSF14, CD206, DEC205, and Dectin-1 were observed with similar frequencies in tonsils (n < 18) and lymph nodes (n < 11).

**Conclusions**

DCs in tonsils and lymph nodes largely share similar features in terms of frequency, maturation, and PRR expression. However, a higher frequency of CD141^+^ cells in tonsils may be of interest considering this subset’s capability in antigen cross-presentation. Our work suggests tonsils as well as lymph nodes as vaccine deposition sites in DC-mediated IT. Furthermore, specific adjuvant measures directed at C-type lectin receptors, TLR2, and XCR1 may be employed to achieve cross-presentation of antigen and cell-mediated tumor antigen-specific effects.

### P56 PD-L1 and immune infiltrates are prognostic and differentially expressed in distinct subtypes of gastric cancer

#### Helen K Angell^1^, Kyoung-Mee Kim^2^, Seung-Tae Kim^2^, Sung Kim^3^, Alan D Sharpe^1^, Julia Ogden^4^, Anna Davenport^5^, Darren R Hodgson^4^, Carl Barrett^6^, Jeeyun Lee^3^, Elaine Kilgour^4^

##### ^1^AstraZeneca, Cambridge, England, UK; ^2^Department of Pathology & Translational Genomics, Samsung Medical Center, Sungkyunkwan University School of Medicine, Seoul, South Korea; ^3^Division of Hematology-Oncology, Department of Medicine, Samsung Medical Center, Sungkyunkwan University School of Medicine, Seoul, South Korea; ^4^AstraZeneca, Macclesfield, England, UK; ^5^University Hospital of South Manchester, Manchester, England, UK; ^6^AstraZeneca, Waltham, MA, USA

###### **Correspondence:** Helen K Angell (helen.angell@astrazeneca.com)

**Background**

Gastric cancer (GC) is often diagnosed at an advanced stage, for which therapeutic options are largely limited to cytotoxic chemotherapy and five-year survival is less than 20%. Immune checkpoint blockade with anti-programmed cell death-1 (PD-1) or anti-programmed cell death ligand-1 (PD-L1) antibodies is emerging as a promising therapeutic approach for several cancer types. An important question is whether the clinical efficacy of PD-1/PD-L1 checkpoint blockade can be improved through combination with targeted agents, such as trastuzumab, for use in human epidermal growth factor receptor 2 (HER2)-positive disease and olaparib, a poly (ADP-ribose) polymerase (PARP) inhibitor. This study determines the association of PD-L1 expression and immune cell infiltrates with clinical outcome and investigates the overlap of these with microsatellite instability (MSI)-high, ATM low and HER2 high segments.

**Methods**

PD-L1 membrane expression on tumour cells (TC) and infiltrating immune cells (IC), CD3+ T lymphocytes, CD8+ cytotoxic T cells, ATM and HER2 were assessed by immunohistochemistry (IHC) in a cohort of 380 Korean gastric cancer patients. PD-L1 positivity was assessed by a pathologist (positive < 0%). CD3 and CD8 were quantified by HALO® image analysis (cells/mm^2^). EBV status was determined using *in situ* hybridization and MSI status was performed using PCR and MLH1 IHC.

**Results**

The ATM-low and HER2-high segments are mutually exclusive and differ markedly in their immune profile; the ATM-low segment being enriched for MSI (p < 0.01), PD-L1 TC positivity (p < 0.01) and CD8+ cytotoxic immune infiltrates (p=0.033), while the HER2 segment is enriched for MSS, with no enrichment for immune markers. The PD-L1 segment is associated with increased T cell infiltrates: CD3 (p < 0.01) and CD8 (p < 0.01), while the MSI-high segment is enriched for PD-L1 TC (p < 0.01), PD-L1 IC (p < 0.001), CD3 (p < 0.05) and CD8 (p < 0.01), and has significant overlap with the ATM-low but not HER2 segments. Multivariate analysis confirmed PD-L1 TC positivity (p < 0.01), high CD3 (overall survival [OS] P < 0.01; disease-free survival [DFS] p=0.021) and high CD8 (OS p < 0.01; DFS p=0.027) as independent prognostic factors for both DFS and OS. Patients with MSI-high tumours had better overall survival by both univariate (p < 0.01) and multivariate (p < 0.05) analysis.

**Conclusions**

Here we present evidence for segmentation of gastric cancers into four distinct molecular segments, namely ATM-low, HER2-high, PD-L1 positive and MSI-high. This illustrates the potential for subsets of GC patients to respond differently to immune therapy and the opportunity to employ different strategies for maximising the benefit from immune therapies in these segments.

### P57 Four color T and B cell ELISPOT assays for simultaneous detection of analytes

#### Jodi Hanson, Richard Caspell, Alexey Karulin, Paul Lehmann

##### Cellular Technology Ltd, Shaker Hts, OH, USA

###### **Correspondence:** Jodi Hanson (jodi.hanson@immunospot.com)

**Background**

ELISPOT assays are a key research tool for enumerating antigen-specific T and B cells in PBMC. As both T and B cells occur in major classes, immune monitoring has to be concerned with identifying these as well. So far, ELISPOT assays have been primarily done single or double color. In this report, we demonstrate the development of four color T and B cell ELISPOT assays.

**Methods**

PBMC were cultured for 4 days with a peptide pool of CMV-, EBV- and Flu- viruses for T cell assays or polyclonal B cell activators for B cell assays. On day 4, cells were washed, counted and plated in a low autofluorescence PVDF plate. Plates were precoated with capture antibodies for detection of IFN-g, IL-2, GzB, or TNF-α (T cell assays) or Ig secretion (B cell assays). During a 4h culture, the cytokine or antibodies secreted by the individual T or B cells respectively was captured on the membrane. The plate-bound “spots” were visualized using cytokine-specific or IgG subclass- or Ig class-specific detection reagents, whereby each detection reagent is distinguished from the other 3 reagents through its unique fluorescence. The four-color assays were analyzed using an ImmunoSpot® S6 Analyzer.

**Results**

We show that the four color assay has the same sensitivity for detecting individual cells secreting analytes as the respective single color assays, and that the four fluorescent colors can be discerned unambiguously, without overlap. Cells secreting any of the four analytes can therefore be identified unambiguously in an automated fashion, without the need for compensation. Cells co-expressing analytes can be identified by superimposing the individual colors. Studying B cells and T cells experimentally has permitted us to verify the accuracy of co-expression measurements. Each B cell secretes only one type of Ig class/subclass. T cells, in contrast, frequently coexpress cytokines. Serial dilution experiments showed that for T cells the numbers of co-expressors linearly decreased with the numbers of cells plated. For B cells, no coexpressors were found.

**Conclusions**

The feasibility of four color T and B cell assays have been shown here. This is particularly important when conserving cell material thereby allowing researchers the opportunity for comprehensive immune monitoring spanning multiple cytokines.

### P58 A positive control for the detection of functional CD4 T cells in human PBMC – CPI protein pool

#### Tameem Ansari, Annemarie Schiller, Srividya Sundararaman, Paul Lehmann

##### Cellular Technology Ltd, Shaker Heights, OH, USA

###### **Correspondence:** Tameem Ansari (tameem.asari@gimmunospot.com)

**Background**

Testing of PBMC for immune monitoring purposes requires verification of their functionality. This is of particular concern when testing cryopreserved PBMC or cells that have been shipped, stored for prolonged periods of time. The CEF peptide pool has been developed as a positive control for CD8 cell functionality. A positive control for detecting CD4 memory cell functionality so far is lacking.

**Methods**

Protein antigens from infectious/ environmental organisms have been selected that are ubiquitous. T cell reactivity to these antigens has been tested in an IFN-g Immunospot® assay from CTL. Cryopreserved PBMC from 100 Caucasian donors were selected from CTL’s ePBMC database for testing. Magnetic bead depletion experiments were performed to verify CD4 or CD8 response.

**Results**

Of an initial selection of 12 antigenic systems, (Varicella, Influenza, Parainfluenza, Mumps, Cytomegalovirus, Streptococcus, Mycoplasma, Lactobacillus, Neisseria, Candida, Rubella, and Measles) 3 were selected as a) eliciting CD4 cells exclusively and b) eliciting recall responses in the majority of donors. While individually none of the antigens triggered recall responses in all of the donors, the pool of these three antigens did. Only 2 of 100 donors did not respond to the CPI (Cytomegalo-, Epstein Barr-, and Influenza- virus) protein pool. These two however were impaired functionally non-viable cells with increased numbers of dead and apoptotic cells and showed increased apoptotic progression. Comparisons with CEF peptide pool, showed clear cut responses in ~50% of donors, borderline responses in xx and no responses in 30% of donors.

**Conclusions**

CPI protein pool is suited as a positive control in Caucasians. Studies are on the way to establish the suitability of this pool for functionality testing in Asians and Africans.

### P59 Maximizing odds for detecting a positive T cell response by ELISPOT

#### Jodi Hanson, Diana Roen, Alexey Karulin, Paul Lehmann

##### Cellular Technology Ltd, Shaker Heights, OH, USA

###### **Correspondence:** Jodi Hanson (jodi.hanson@immunospot.com)

**Background**

It has been a matter of debate to determine the best cutoffs in ELISPOT assay analysis for the unambiguous identification of a positive T cell response. At present, the answers to the above question is largely based on empirical or mixed criteria. To come up with scientifically validated answers, parametric statistical analysis has to be used, which in turn requires knowledge about the distributional properties of ELISPOT counts in replicate wells.

**Methods**

PBMC of HLA-A2-0201 positive human subjects who had been infected with HCMV were plated with a HCMV peptide antigen (CEF-7 peptide, pp65 (495-503)) at 100,000 cells per well in IFH-g ELISPOT assays. However, we selected donors whose PBMC, when tested at 100,000 cells per well and challenged with the HLA-A2-0201-restricted HCMV peptide, pp65(495-503) did not display spot counts over medium background. We tested the PBMC for pp65 reactivity in 96 replicate wells to establish the distributional properties of these low frequency ELISPOTs. The distributional properties of the spot counts in the replicate wells were analyzed using diagnostic plots (QQ plots) and the Shapiro-Wilk normality tests.

**Results**

We observed that increasing the number of PBMC plated per well resulted in higher positive to negative count ratio, lower relative experimental error (CV), and higher power for detecting pp65-induced positive responses without causing false positive results from HCMV negative subjects. This decrease of CV and increase in the power of the test was directly proportional to the numbers PBMC plated. The distributional properties showed that the spot counts in replicate wells follow Normal distribution. We showed that parametric statistics, such as Student's t test can be used and provide higher statistical power detecting weak positive HCMV responses than nonparametric methods (Wilcoxon or DFR). We also show that compared to increasing cell numbers per well, the increase of replicate wells is a more efficacious means of establishing positivity when statistical analysis is at the limits of confidence.

**Conclusions**

The Normal distribution of ELISPOT counts permits us to make precise predictions regarding the numbers of replicate wells needed, and cut off values, especially when responses from donors are low.

### P60 Association between microsatellite instability and clinical response across tumor types in the phase Ib KEYNOTE-012 and KEYNOTE-028 studies of pembrolizumab in PD-L1-expressing advanced solid tumors

#### Mark Ayers^1^, Diane Levitan^2^, Gladys Arreaza^2^, Fang Liu^2^, Robin Mogg^2^, Yung-Jue Bang^3^, Bert O'Neil^4^, Razvan Cristescu^2^

##### ^1^Merck & Co., Inc., West Point, PA, USA; ^2^Merck & Co., Inc., Kenilworth, NJ, USA; ^3^Seoul National University College of Medicine, Seoul, Republic of Korea; ^4^Indiana University Health University Hospital, Indianapolis, IN, USA

###### **Correspondence:** Mark Ayers (mark.ayers@merck.com)

**Background**

High levels of microsatellite instability (MSI-H) can occur in some patients with colorectal cancer (CRC) due to defects in mismatch repair (MMR). In the phase II KEYNOTE-016 study, MSI-H CRC patients were more responsive to PD-1 inhibition with pembrolizumab, as were MSI-H non-CRC patients. Here, we evaluate loss of *MLH1* gene expression across tumor types, and effect of MSI status on clinical response to pembrolizumab in the KEYNOTE-012 and KEYNOTE-028 studies in patients with recurrent, metastatic tumors including breast, gastric, urothelial, and CRC tumors.

**Methods**

Microarray analysis of loss of *MLH1* gene expression was used as a surrogate for MSI-H status to assess prevalence of MSI-H in the proprietary Moffitt database of approximately 18,000 annotated, archived tumors. MSI status of tumors from KEYNOTE-012 and KEYNOTE-028 was evaluated using the Promega MSI Analysis system v1.2. Microsatellite markers were amplified from DNA isolated from tumors and separated and size analyzed by capillary electrophoresis. MSI-H status was identified by comparison of formalin-fixed, paraffin-embedded tumor samples to matched blood DNA. Samples were MSI-H if 2 or more markers changed or non-MSI-H if 1 or no markers changed relative to blood. Logistic regression analyses assessed the correlation between MSI status and overall response rate (ORR, centrally-assessed RECIST v1.1).

**Results**

**Fig. 36:**
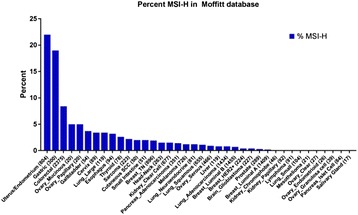
**(Abstract P60).**

In the Moffitt database, loss of *MLH1* expression mirrored results from a clinical MSI immunohistochemistry assay in CRC patients. Loss of *MLH1* expression also correlated with mutational load. Inferred MSI-H status was identified across multiple tumor types (aggregate prevalence 3.2%) in the Moffitt database (Fig. 36). In KEYNOTE-012 (n=96) and KEYNOTE-028 (n=265), MSI-H status was identified in 6% and 2% of patients, respectively, (3% overall), similar to the unselected population in the Moffitt database. Gastric cancer had the highest prevalence (n=4 patients), with estrogen receptor positive breast, biliary, esophageal, triple negative breast, endometrial, CRC, and bladder tumors also having MSI-H in at least one patient. For patients with MSI and response data (n=310), ORR was 70% versus 12% (1-sided p=0.0001) in those with MSI-H and non-MSI-H status, respectively.

**Conclusions**

In this retrospective analysis, identification of MSI-H status was associated with a statistically relevant increase in response to pembrolizumab. This suggests that determination of MSI-H status may be predictive of response to pembrolizumab regardless of histology. The association between MSI-H status and clinical benefit of anti-PD-1 therapy is being evaluated in ongoing studies (KEYNOTE-158 [NCT02628067], KEYNOTE-164 [NCT02460198], and KEYNOTE-177 [NCT02563002]).

**Trial Registration**

ClinicalTrials.gov identifier NCT01848834 and NCT02054806.

### P61 Whole-blood RNA transcript-based signatures predict pre- and post-treatment response in two large independent clinical studies of patients with advanced melanoma treated with tremelimumab

#### Philip Friedlander^1^, Karl Wassman^2^, Chrisann Kyi^1^, William Oh^1^, Nina Bhardwaj^3^

##### ^1^Icahn School of Medicine at Mount Sinai, New York, NY, USA; ^2^Check Point Sciences, Cambridge, MA, USA; ^3^Tish Cancer Institute, Icahn School of Medicine at Mount Sinai, New York, NY, USA

###### **Correspondence:** Nina Bhardwaj (nina.bhardwaj@mssm.edu)

**Background**

Tremelimumab is a cytotoxic T lymphocyte-associated antigen-4-blocking monoclonal antibody. An unmet clinical need exists for blood-based response-predictive gene signatures to facilitate use of cancer immunotherapy in the most clinically effective and cost-efficient manner.

**Methods**

Pre- and post-treatment (30 days) peripheral blood samples were taken from 210 treatment-naïve melanoma patients receiving tremelimumab in a worldwide, multicenter phase III study. Objective response was determined by an expert panel of radiologists using RECIST criteria. 169 mRNA transcripts were measured for the n=210 patients using reverse transcription polymerase chain reaction (RT-PCR) [1]. Pre- and post-treatment response-predictive signatures were identified in the n=210 training dataset. An independent population of n=150 refractory/relapsed melanoma patients receiving tremelimumab after chemotherapy enrolled in a worldwide, multicenter phase II study [2] was the test dataset.

**Results**

A 16-gene pre-treatment and 8-gene post-treatment mRNA signatures were identified in the n=210 training dataset. These pre- and post-treatment signatures were tested in the n=150 test dataset first, for objective response as determined by RECIST criteria, and second for one-year survival after treatment. The same genes, coefficients and constant from the training dataset were used in the test cases with the results in Table 5. Both the 16-gene pre- and 8-gene post-treatment response-prediction training gene signatures validated when compared to objective response in the test dataset. The one-year survival criteria also validated with even higher AUC’s for both pre- and post-treatment.

**Table 5:** **(Abstract P61).**

	Pre-Treatment Blood-Based			Post-Treatment Blood-Based		
	16-Gene mRNA Signature			8-Gene mRNA Signature		
Melanoma	Training	Test	Test	Training	Test	Test
Cases	N=210	N=150	N=150	N=210	N=150	N=150
Biomarker	Response	Response	Survival	Response	Response	Survival
Sensitivity	96.4%	65.0%	74.4%	85.7%	70.0%	69.8%
Specificity	66.5%	59.2%	55.1%	64.3%	60.8%	59.8%
NPV	99.2%	91.7%	84.3%	96.7%	92.9%	83.1%
AUC	0.8634	0.6376	0.6785	0.7853	0.6125	0.6512

**Conclusions**

This is the first large clinical study of tremelimumab, independently validated in a second large clinical study, to show both pre- and post-treatment response-predictive mRNA signatures in blood. The pre-treatment biological signature may represent expression levels of particular immune system genes that are needed for a robust immune response against cancer. They may identify patients whose immune systems are already primed to fight the cancer and are particularly amenable to a boost in effectiveness provided by immunotherapy. The 30-day post-treatment biological signature represents a timely way to determine whether the patient is responding positively to the immunotherapy.

**Trial Registration**

ClinicalTrials.gov identifier NCT00257205.

**References**

1. Ribas A, *et al*: **Phase III randomized clinical trial comparing tremelimumab with standard-of-care chemotherapy in patients with advanced melanoma.** *J Clin Oncol* 2013, **31(5)**:616-622.

2. Kirkwood JM, *et al*: **Phase II trial of tremelimumab (CP-675,206) in patients with advanced refractory or relapsed melanoma.** *Clin Cancer Res* 2010, **16(3)**:1042-1048.

### P62 Extensive analysis of PD-1 and CTLA-4 in HVs and GBM patients: implications for monitoring patients on checkpoint inhibitors

#### Svetlana Bornschlegl, Michael P Gustafson, Dennis A Gastineau, Ian F Parney, Allan B Dietz

##### Mayo Clinic, Rochester, MN, USA

###### **Correspondence:** Svetlana Bornschlegl (bornschlegl.svetlana@mayo.edu)

**Background**

Checkpoint inhibitors are becoming widely used for immunotherapy but methods to monitor dosing and duration for each individual patient needs to be more fully understood. Immune monitoring by flow cytometry is a tool that can be utilized for measuring responses to immunotherapy in patients. In this study we assessed the expression of PD-1 and CTLA-4 on numerous cell types in healthy volunteers (HVs) and glioblastoma (GBM) patients enrolled in a dendritic cell clinical trial.

**Methods**

Peripheral blood was collected from 20 HV and 20 GBM patients receiving a DC vaccine in a clinical trial. Whole blood was stained using a previously established method for the identification of multiple cell populations by flow cytometry and novel analysis that captures data on over 120 phenotypes [1]. An extended analysis focused on T cell phenotypes was performed using markers for CD154, CD45RO, CD56, CD3, CD8, CD28, CD4, and CD45. T cell parent populations were characterized by SS, FS, CD45^+^, CD3^+^, CD4^+^, CD8^+^, CD4^+^/CD8^+^ sub populations. Non-T cell populations were assessed by various gating strategies. These populations were measured for PD-1^+^, CTLA4^+^, DP, and DN populations.

**Results**

We identified 15 parent populations, of which 11 expressed PD-1 and 9 expressed CTLA-4. Within subsets of the parent populations we found statistically significant differences (p <0.001) in PD-1 between CD8^+^ memory and CD8^+^ naïve cells, CD4^+^ memory and CD4^+^ naïve cells, CD8^+^ NKT and CD8^+^CD3^+^ cells, as well as NKT and NK cells. These statistical differences hold true for both HV and GBM patients. We also found HVs to have higher levels of CTLA-4 on CD4^+^CD8^+^ cells and B cells compared to GBM patients, and lower levels of PD-1 on CD8^+^ and naïve CD8^+^ cells.

**Conclusions**

This panel allows us to measure approximately 60 phenotypes related to checkpoint proteins. The data presented here identify PD-1 and CTLA-4 phenotypic differences within parent populations, within subsets of parent populations, and differences in healthy volunteers compared to GBM patients. These results may help optimize the targeting of checkpoint proteins as well as other immunotherapeutic approaches in clinical trials.

**Acknowledgements**

This study is funded in part by the Ivy Foundation.

**Trial Registration**

ClinicalTrials.gov identifier NCT01957956.

**References**

1. Gustafson MP, *et al*: **A method for identification and analysis of non-overlapping myeloid immunophenotypes in humans.** *PloS ONE* 2015, **10.3**:e0121546.

### P63 Objective measurement and clinical significance of IDO1 protein in hormone receptor-positive breast cancer

#### Daniel Carvajal-Hausdorf^1^, Nikita Mani^1^, Vamsidhar Velcheti^2^, Kurt Schalper^1^, David Rimm^1^

##### ^1^Yale University School of Medicine, New Haven, CT, USA; ^2^Cleveland Clinic Main Campus, Cleveland, OH, USA

###### **Correspondence:** Daniel Carvajal-Hausdorf (daniel.carvajal@yale.edu)

**Background**

Immunostimulatory therapies targeting immune suppressive pathways produce durable clinical responses in advanced solid tumors. Indoleamine 2, 3-dioxygenase (IDO) is the rate-limiting oxidoreductase that catalyzes the degradation of tryptophan to kynurenine. IDO induces immune tolerance by downregulating CD8+ and effector CD4+ T cell responses. IDO1, the most active isoform, is expressed in diverse tumor types and can be targeted using small molecule inhibitors. Here, we used a validated quantitative in situ assay to measure the levels of IDO1 protein in a retrospective collection of hormone receptor-positive breast cancer (BC).

**Methods**

IDO1 was measured using quantitative immunofluorescence (QIF) in 362 stage I-III hormone-receptor-positive breast carcinomas represented in tissue microarray format. The levels of IDO1 were determined in the tumor compartment; and were stratified using the median as cut-point. The association between IDO1 levels, clinico-pathological features, CD3+, CD8+ and CD20+ tumor-infiltrating lymphocytes (TIL) and survival was studied.

**Results**

IDO1 protein was detected in 76.2% of hormone receptor-positive BC. There was no significant association between IDO1 levels and major clinico-pathological characteristics. Increased IDO1 correlated with decreased CD20+ infiltration (P=0.0004) but not with changes in CD3+ or CD8+ levels. Elevated IDO1 expression was associated with worse 20-year overall survival (log-rank P=0.02, HR=1.39, 95% C.I.: 1.05-1.82). IDO1 scores were independently associated with outcome in multivariable analysis.

**Conclusions**

IDO1 protein is expressed in the majority of hormone receptor-positive BC and is an independent negative prognostic marker. Additionally, IDO1 expression is negatively associated with tumor B cell infiltration. Measurement of IDO1 has the potential to identify a population that might derive benefit from IDO1 blockade.

**References**

1. Munn DH, Zhou M, Attwood JT, Bondarev I, Conway SJ, Marshall B, *et al*: **Prevention of allogeneic fetal rejection by tryptophan catabolism**. *Science* 1998, **281(5380)**:1191-1193.

2. Uyttenhove C, Pilotte L, Theate I, Stroobant V, Colau D, Parmentier N, *et al*: **Evidence for a tumoral immune resistance mechanism based on tryptophan degradation by indoleamine 2,3-dioxygenase**. *Nat Med* 2003, **9(10)**:1269-1274.

3. Ling W, Zhang J, Yuan Z, Ren G, Zhang L, Chen X, *et al*: **Mesenchymal stem cells use IDO to regulate immunity in tumor microenvironment**. *Cancer Res* 2014, **74(5)**:1576-1587.

4. Schalper KA, Carvajal-Hausdorf D, McLaughlin J, Altan M, Velcheti V, Gaule P, *et al*: **Differential expression and significance of PD-L1, IDO-1 and B7-H4 in human lung cancer**. *Clin Cancer Res* 2016.

### P64 CD8+ T cell subsets may be associated with response to anti-CD137 agonist antibody treatment

#### Serena Chang^1^, Ronald Levy^1^, John Kurland^2^, Suba Krishnan^2^, Christoph Matthias Ahlers^2^, Maria Jure-Kunkel^2^, Lewis Cohen^2^, Holden Maecker^1^, Holbrook Kohrt^1^

##### ^1^Stanford University School of Medicine, Stanford, CA, USA; ^2^Bristol-Myers Squibb, Princeton, NJ, USA

###### **Correspondence:** Serena Chang (ssc233@stanford.edu)

**Background**

Cancer immunotherapies historically, have low success rates. One way to increase these success rates involves investigating baseline or early response biomarkers that predict success. Selecting patients with early biomarkers for success and competent immune status are essential when choosing the best therapeutic strategy. CD137 agonists, activators of T and NK cells and a promising newer immunotherapy, have dramatically reduced tumor size and disease in murine tumor models [1] and are currently being examined in clinical trials [2].

**Methods**

This study investigated the immune response and potential early immune biomarkers in both solid tumor and hematologic cancer patients participating in the phase I BMS-663513 NCT01471210 clinical trial of urelumab monotherapy. We used peripheral blood mononuclear cells to determine the circulating immune competence of patients (n=8) before treatment (baseline/C1D1), 24 hours after the first treatment (C1D2), before the second treatment (C2D1), and up to 30 days after the third treatment (C3R). Analysis was performed using mass cytometry, surveying 38 different immune proteins simultaneously.

**Results**

At all time points, we observed a trend toward higher central memory and naïve CD8+ T cells in patients with stable disease (n=3) or partial response (n=1) vs. progressors (n=4), while the opposite was true in effector and effector memory RA cells. The most striking difference was seen when considering all CD8+CD27+ T cells, which were higher in those with stable disease or partial response, at all time points. CD8+FcεR1α+ T cells showed a similar trend, albeit to a lesser extent, while CD57+CD8+ T cells showed the opposite trend. CD8+ T cells in both groups were comparably responsive to PMA/ionomycin stimulation, producing multiple cytokines. These trends were not seen in CD4+ T cells or with head and neck solid tumor patients treated with cetuximab.

**Conclusions**

The aforementioned trends suggest that CD27+CD8+ T cells, and possibly other subsets of CD8+ T cells, should be further explored to determine whether they predict response to anti-CD137 agonist therapy. They also suggest that potential predictive measures of immune status prior to immunotherapy are detectable in peripheral blood.

**References**

1. Sabel MS, *et al*: **Monoclonal antibodies directed against the T-cell activation molecule CD137 (interleukin-A or 4-1BB) block human lymphocyte-mediated suppression of tumor xenografts in severe combined immunodeficient mice**. *J Immunother* 2000, **23(3)**:362-368.

2. Sznol M, *et al*: **Phase I study of BMS-663513, a fully human anti-CD137 agonist monoclonal antibody, in patients (pts) with advanced cancer** J. *Clin Oncol* 2008.

### P65 Regulation of PD-L1 expression in melanoma and immune cells

#### Shuming Chen^1^, George Crabill^1^, Theresa Pritchard^1^, Tracee McMiller^1^, Drew Pardoll^2^, Fan Pan^2^, Suzanne Topalian^1^

##### ^1^Department of Surgery, Johns Hopkins University School of Medicine, Sidney Kimmel Comprehensive Cancer Center, and Bloomberg-Kimmel Institute for Cancer Immunotherapy, Baltimore, MD, USA; ^2^Department of Oncology, Johns Hopkins University School of Medicine, Sidney Kimmel Comprehensive Cancer Center, and Bloomberg-Kimmel Institute for Cancer Immunotherapy, Baltimore, MD, USA

###### **Correspondence:** Shuming Chen (schen72@jhmi.edu)

**Background**

The therapeutic effects of PD-1/PD-L1 inhibition in multiple cancers indicates a critical role for this pathway in immunosuppression in the tumor microenvironment (TME), but factors regulating PD-L1 expression on tumor and immune cells are poorly understood. The dichotomous transcription factors STAT1 and STAT3 have both been reported to bind the PD-L1 promoter. The current study investigates the role of STAT1/3 and other signaling in cytokine-induced PD-L1 expression on human melanoma (MEL) cells and monocytes.

**Methods**

Seventeen cultured MELs or short-term monocyte cultures were treated with recombinant cytokines including IFN-g, IL-6, IL-10, IL-32g, or TNF-a. PD-L1 cell surface protein expression was detected by flow cytometry, and mRNA by quantitative real-time RT-PCR (qRT-PCR). STAT1 or/and STAT3 were knocked down by small interfering RNAs (siRNAs). Total and phosphorylated STAT1/3 proteins were quantified by Western blotting.

**Results**

While PD-L1 is expressed on 35-40% of MELs *in situ*, it was not expressed on 17 cultured MELs. IFN-g significantly enhanced PD-L1 protein expression on MELs (p=0.0003), increasing PD-L1 mRNA expression (qRT-PCR) in association with PD-L1 cell surface protein expression (FACS) in all MELs tested (p=0.0004). This suggests that IFN-g regulates PD-L1 expression primarily at the transcriptional level and not via translocation of intracellular protein stores. Enhanced PD-L1 expression in IFN-g-treated MELs correlated with increased STAT1 phosphorylation (p=0.05). Consistent with this, siRNA knockdown of STAT1 reduced PD-L1 expression by 40-70% in 2 MELs after 24-48hr IFN-g exposure. In contrast, STAT3 knockdown reduced IFN-g-induced PD-L1 expression by only 12-15%, on one of two MEL lines. In cultured monocytes from two donors, PD-L1 mRNA expression was induced by IFN-g, IL-10, IL-32-g and TNF-a, and significantly correlated with PD-L1 cell surface protein expression, suggesting that these cytokines acted at the transcriptional level. In monocytes, IFN-g was associated with markedly increased pSTAT1, and IL-10 with increased pSTAT3. These cytokines were not associated with increased pp65 or focal adhesion kinase (FAK) phosphorylation in monocytes.

**Conclusions**

In addition to IFN-g, other cytokines in the TME may play important, coordinate and selective roles in promoting PD-L1 expression on different cell types including tumor and stromal cells. pSTAT1 and pSTAT3 are associated with PD-L1 protein expression in response to different cytokine stimuli. Future studies will further characterize cytokine-triggered transcription factors and signaling pathways responsible for PD-L1 expression on tumor cells and immune cells. Understanding mechanisms regulating PD-L1 expression will help guide the development of more optimal predictive biomarkers and combinatorial therapies based on anti-PD-1.

### P66 Gene expression markers of tumor infiltrating leukocytes

#### Patrick Danaher^1^, Sarah Warren^1^, Lucas Dennis^1^, Andrew M White^1^, Leonard D'Amico^2^, Melissa Geller^3^, Mary L Disis^2^, Joseph Beechem^1^, Kunle Odunsi^4^, Steven Fling^2^

##### ^1^NanoString Technologies, Seattle, WA, USA; ^2^University of Washington, Seattle, WA, USA; ^3^University of Minnesota, Minneapolis, MN, USA; ^4^Roswell Park Cancer Institute, Buffalo, NY, USA

###### **Correspondence:** Patrick Danaher (pdanaher@nanostring.com)

**Background**

Assays of the abundance of immune cell populations in the tumor microenvironment promise to inform immune oncology research and the choice of immunotherapy for individual patients. We propose to measure the intratumoral abundance of various immune cells populations with gene expression. In contrast to IHC and flow cytometry, gene expression assays yield high information content from a clinically practical workflow. Previous studies of gene expression in purified immune cells have reported hundreds of genes showing enrichment in a single cell type, but the utility of these genes in tumor samples is unknown. We describe a novel statistical method for using co-expression patterns in large tumor gene expression datasets to validate previously reported candidate cell type marker genes.

**Methods**

We used co-expression patterns in 9986 samples from The Cancer Genome Atlas (TCGA) to validate previously reported cell type marker genes. We compared immune cell scores derived from these genes to measurements from flow cytometry and immunohistochemistry. We characterized the reproducibility of our cell scores in replicate runs of RNA extracted from FFPE tumor tissue.

**Results**

We identified a list of 60 marker genes whose expression levels quantify 14 immune cell populations. Cell type scores calculated from these genes are concordant with flow cytometry and IHC readings, show high reproducibility in replicate RNA samples from FFPE tissue and reveal an intricate picture of the immune infiltrate in TCGA. Most genes previously reported to be enriched in a single cell type have co-expression patterns inconsistent with cell type specificity.

**Conclusions**

Due to their concise gene set, computational simplicity and utility in tumor samples, these cell type gene signatures may be useful in future discovery research and clinical trials to understand how tumors and therapeutic intervention shape the immune response.

**Fig. 37:**
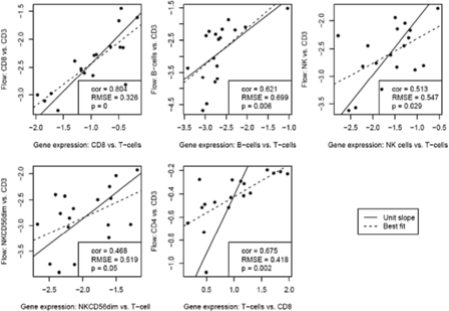
**(Abstract P66).** Comparison of gene expression and flow cytometry cell type measurements in PBMCs

**Fig. 38:**
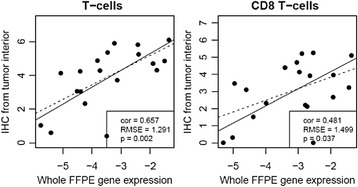
**(Abstract P66).** Comparison of gene expression and IHC cell type measurements in FFPE

**Fig. 39:**
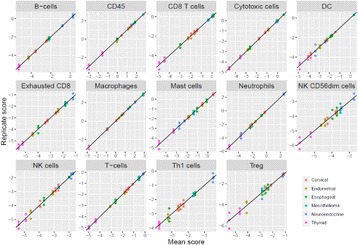
**(Abstract P66).** Reproducibility of gene expression measurements of immune cell types

### P67 Quantitative real-time PCR based diagnostic to assess NKT cell function in breast cancer patients

#### Roshanak Derakhshandeh, Tonya J Webb

##### University of Maryland, Baltimore, Baltimore, MD, USA

###### **Correspondence:** Roshanak Derakhshandeh (rderakhshandeh@som.umaryland.edu)

**Background**

Breast cancer is a leading cause of cancer-related death among women worldwide. Although, surgery, radiotherapy, and chemotherapy have improved the 5-year survival rate, new treatment methods are needed to combat this disease. To date, significant efforts have been invested into harnessing the therapeutic potential of the immune system for the treatment of cancer. However, tumor tolerance and immune suppression can severely limit its therapeutic efficacy. In fact, natural killer T (NKT) cells play an important role in cancer immune surveillance, but are reduced in cancer patients. In order to assess which patients will likely benefit from immune cell-based therapies, we have developed a quantitative method to rapidly assess the baseline function of NKT cells using stimulation with artificial antigen presenting cells followed by and quantitative real-time PCR (aAPC-qPCR).

**Methods**

In this study, we assessed NKT cell number and function in healthy donors and breast cancer patients by flow cytometry, ELISA, and qPCR. In addition, we assessed the percentage of tumor-infiltrating lymphocytes and PD-L1 expression within the tumor microenvironment by immunohistochemistry.

**Results**

Although % circulating NKT cell were significantly reduced in breast cancer patients (BCP), compared to healthy donors (HD), we detected NKT cell function in 82% HD (n=22) and 70% BCP (n=30). We compared high responders (high IFN-γ induction) to low responders and found that there was no significant difference in NKT cell number between these two groups. Following further characterization of these groups, it was found that low responders had a significant reduction in the induction of TNFα, LAG3, and LIGHT.

**Conclusions**

In summary, this data we have developed a novel diagnostic platform using aAPC-qPCR to determine NKT cell function in patients. This technology is important because NKT cell number did not correlate with function in our breast cancer patient cohort. Thus, our studies demonstrate that there is a critical need to assess baseline immune function prior to the initiation of immunotherapy. Future studies can focus identifying new breast cancer classifications according to immune gene expression patterns, and these tumor subtypes may provide a basis for new therapeutic opportunities.

**Acknowledgements**

Supported by NIH/NCI 1R21CA162273, R21CA184469, and R21CA199544 grants to Tonya Webb.

**References**

1. Siegel RL, Miller KD, Jemal A: **Cancer Statistics 2015**. *CA Cancer J Clin* 2015, **65(1)**: 5-29.

2. Hermans IF, *et al*: **NKT cells enhance CD4+ and CD8+ T cell responses to soluble antigen in vivo through direct interaction with dendritic cells**. *J Immunol* 2003, **171(10)**: 5140-5147.

**Fig. 40:**
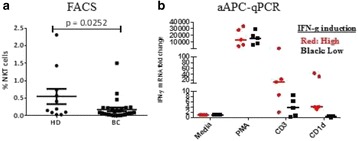
**(Abstract P67). a** Circulating % NKT cells are reduced in BC patients. Peripheral blood mononuclear cells (PBMC) were collected from healthy donors (HD) and breast cancer patients (BC), stained for NKT TCR and analyzed by flow cytometry. **b** NKT cell function correlates total T cell function in BC patients. PBMCs from BC patients were stimulated with CD1D-Ig aAPC loaded with a-GalCer to specifically activate NKT cells or anti-CD3/CD28 to activate T cells. Media serves as a negative control and PMA/ionomycin was the positive control. After 4 hours, RNA was extracted and qPCR was performed to assess IFN-g and 18S. Fold induction was calculated relative to the control

### P68 Arming of CD56^dim^ and CD56^bright^ NK cells in IL-15-infused cancer patients

#### Sigrid Dubois, Kevin Conlon, Bonita Bryant, Jennifer Hsu, Nancy Beltran, Jürgen Müller, Thomas Waldmann

##### Lymphoid Malignancies Branch, Center for Cancer Research, National Cancer Institute, Bethesda, MD, USA

###### **Correspondence:** Sigrid Dubois (duboiss@mail.nih.gov)

**Background**

Survival and expansion of NK cells depends on interleukin (IL)-15. It has recently been shown that IL-15 infusions caused NK cell expansions in cancer patients. The objectives of our study were to assess the effects of IL-15 on functions of CD56^dim^ and CD56^bright^ subpopulations of NK cells in IL-15-treated cancer patients.

**Methods**

We monitored numbers, phenotypic changes, cytokine production and lytic activities of CD56^dim^ and CD56^bright^ NK cell subpopulations in the blood of cancer patients that had received daily infusions of 2 μg/kg IL-15 for 10 days.

**Results**

Ten-day continuous infusion of IL-15 led to expansions of both CD56^dim^ and CD56^bright^ subpopulations of NK cells. Phenotypic analyses revealed that IL-15 infusions caused the expression of CD56^bright^–typical surface proteins on CD56^dim^ NK cells that included Trail, CD62L, NKG2D, CD94 and the IL-18 receptor, while CD56^bright^ NK cells remained essentially unchanged. CD56^bright^ NK cells retained their superior ability to produce IFNγ/TNFα in responses to IL-12/IL-18 when compared with CD56^dim^ NK cells, and IL-15 infusions increased the percentage of cytokine-producing CD56^bright^ NK cells. The cytotoxic capacity of CD56^dim^ NK cells remained superior to CD56^bright^ NK cells even after IL-15 infusions. However, cytotoxic competencies were increased for both subpopulations after IL-15 infusions that resulted in substantial lytic activities via natural cytotoxicity receptors, stress receptors, and antibody-dependent cytotoxicity even among CD56^bright^ NK cells.

**Conclusions**

These data show that IL-15 infusions increase the functional abilities of both types of NK cells in cancer patients.

### P69 Identification of a novel subset of tumor-resident human CD8+ T cells, marked by dual expression of CD103 and CD39

#### Rebekka Duhen^1^, Thomas Duhen^2^, Lucas Thompson^3^, Ryan Montler^2^, Andrew Weinberg^1^

##### ^1^Earle A. Chiles Research Institute, Providence Cancer Center, Portland, OR, USA; ^2^AgonOx, Inc., Portland, OR, USA; ^3^Juno Therapeutics, Portland, OR, USA

###### **Correspondence:** Rebekka Duhen (rebekka.duhen@providence.org)

**Background**

Homing and retention of T cells in tissues is mediated by the interaction of adhesion molecules with their respective ligands. Among those, the integrin CD103 interacts with its ligand E-cadherin and allows T cells to home to epithelial tissues. T cells expressing high levels of CD103 have recently been identified as tissue-resident memory (T_RM_) cells and play a crucial role in protecting epithelial tissues against viral infections. Previous reports have shown that CD103+ CD8+ T cells were present in some but not all human solid malignancies.

**Methods**

Cell sorting, gene expression analysis, and TCR sequencing.

**Results**

Here, while confirming these data, we identify a subset of CD103+ CD8+ T cells that co-express the ectonucleotidase CD39. This subset is enriched in primary tumors and metastatic lymph nodes but absent in the blood and normal lymph nodes of cancer patients. We compared several tumor histologies and found highest frequencies in head and neck squamous cell carcinomas (HNSCC), ovarian, lung and rectal cancers (ranging from 20-80% of tumor-infiltrating CD8+ T cells), whereas those cells were absent or low in primary colon cancer and colorectal liver metastasis. Gene expression analysis of CD103/39 double positive CD8+ T cells revealed a gene signature reminiscent of T_RM_ cells (CCR7^lo^, L-selectin^lo^, S1PR1^lo^ and CD69^hi^), and their activated phenotype (HLA-DR^hi^, Ki67^hi^, Granzyme B^hi^) implies strong tumor reactivity. Furthermore, TCR repertoire analysis shows high clonality and distinct CDR3 sequences in this subset compared to other CD8+ T cells present in the tumor. Based on this phenotype, gene signature, circulation pattern and clonality, we believe that CD103/39 double positive CD8+ T cells are being chronically stimulated within the tumor microenvironment, and may recognize neoantigens. In support of this finding, our *in vitro* data suggests that expression of CD39 is upregulated as a result of strong, sustained TCR stimulation in naïve CD8+ T cells. Finally, to better address the role of those cells *in vivo* we examined 9 different solid murine tumor models. Unexpectedly, CD103/39 double positive CD8+ T cells were only found in the transgenic MMTV-PyMT breast cancer model. Utilizing this model, we plan to study their differentiation and function *in vivo* and address their antigenic specificity and role in tumor development.

**Conclusions**

Taken together our findings suggest that targeting tumor-resident CD103/39 CD8+ T cells may be a promising approach to enhance immune-mediated tumor regression.

### P70 Characterization of tumor infiltrating T cell receptor (TCR) repertoire in non-muscle invasive bladder cancer (NMIBC) patients treated with Bacillus Calmette-Guérin (BCG)

#### Max Kates^1^, Brandon Early^2^, Erik Yusko^3^, Taylor H Schreiber^2^, Trinity J Bivalacqua^1^

##### ^1^Johns Hopkins University, Baltimore, MD, USA; ^2^Heat Biologics, Durham, NC, USA; ^3^Adaptive Biotechnologies, Seattle, WA, USA

###### **Correspondence:** Brandon Early (bearly@heatbio.com)

**Background**

Bladder cancer is the 5^th^ most common malignancy in the US, and the majority of bladder cancer is diagnosed while confined to the most superficial layer (non-muscle invasive bladder cancer, NMIBC) [1]. Treatment goals in this minimal residual disease setting are ideal for immunotherapy: reduce local disease recurrence following surgical resection and prevent progression to muscle invasive bladder cancer (MIBC). Bacillus Calmette-Guérin (BCG), an attenuated form of *Mycobacterium bovis*, is an intravesical immunotherapy which remains the mainstay of NMIBC treatment since 1976. Despite the tenure in bladder cancer treatment, full characterization of the immunologic mechanism of action of BCG is still lacking [2]. In a pilot study, we sought to investigate the diversity of T cells infiltrating bladder tumors and compare the changes in T cell diversity among patients who were responders and unresponsive to BCG.

**Methods**

Six patients were selected from the IRB-approved Johns Hopkins bladder cancer tumor repository. All patients had T1 disease without concurrent *carcinoma in situ* (CIS) and received standard of care BCG induction and maintenance. Three patients were classic “responders” to BCG, with disease-free intervals of 8, 28, and 34 months. Three patients were unresponsive to BCG: two patients progressed to MIBC and ultimately died of metastatic bladder cancer and one had recurrent disease requiring cystectomy. Pre-treatment and post-treatment tumor tissue samples were compiled; genomic DNA was isolated, amplified, and sequenced using Adaptive Biotechnologies’ ImmunoSeq assay.

**Results**

ImmunoSeq technology surveyed the T cells in all 12 samples. Median fraction of T cells in the samples pre-treatment was 0.16 (95% CI, -0.419 to 0.731) while post-treatment median was 0.36 (95% CI, 0.184 to 0.532), indicating a trend towards a higher T cell fraction after treatment. Clonality, an objective measure of T cell diversity, was low in most patients and unchanged post-treatment: some T cell clones in the tumor samples expanded or contracted, though most clones were stable post-treatment, and no clones expanded to be dominant. Clonality likewise did not appear to correlate with patient response to BCG.

**Conclusions**

Tumor infiltrating T cell kinetics do not appear to correlate with response to BCG in this pilot sample of patients. These data suggest that an alternative mechanism involving the innate immune cell population may be the primary driver of BCG response.

**References**

1. **SEER Cancer Statistics Factsheets: Bladder Cancer.** National Cancer Institute. http://seer.cancer.gov/statfacts/html/urinb.html. Accessed 25 Jul 2016.

2. Biot C, *et al*: **Preexisting BCG-specific T cells improve intravesical immunotherapy for bladder cancer.** *Sci Transl Med* 2012, **4**:137ra72.

### P71 An immune-related gene expression profile delineates features of the tumor microenvironment required for clinical response to PD-1 blockade

#### Mark Ayers^1^, Jared Lunceford^1^, Michael Nebozhyn^1^, Erin Murphy^1^, Andrey Loboda^1^, David R Kaufman^1^, Andrew Albright^1^, Jonathan Cheng^1^, S Peter Kang^1^, Veena Shankaran^2^, Sarina A Piha-Paul^3^, Jennifer Yearley^1^, Tanguy Seiwert^4^, Antoni Ribas^5^, Terrill K McClanahan^1^

##### ^1^Merck & Co., Inc., Kenilworth, NJ, USA; ^2^University of Washington, Seattle, Seattle, WA, USA; ^3^University of Texas MD Anderson Cancer Center, Houston, TX, USA; ^4^University of Chicago, Chicago, Chicago, IL, USA; ^5^University of California, Los Angeles, Los Angeles, CA, USA

###### **Correspondence:** Mark Ayers (mark.ayers@merck.com)

**Background**

PD-L1 expression, which predicts response to PD-1-directed therapies such as pembrolizumab in many cancer types, is often associated with an active T cell infiltrate in the tumor microenvironment, suggesting that the presence of activated intratumoral T cells may be a determinant of response to PD-1 checkpoint blockade. This study describes the stepwise derivation of an immune-related gene expression profile (GEP) that identifies key biological features of a T cell inflamed tumor microenvironment and predicts clinical response to pembrolizumab across a wide variety of solid tumors.

**Methods**

Associations between clinical response to pembrolizumab and gene expression signatures of IFN-γ signaling and activated T cell biology were evaluated using RNA isolated from formalin-fixed paraffin-embedded baseline samples of patients with multiple tumor histologies. Gene expression was analyzed on the NanoString nCounter system.

**Results**

Preliminary signatures, comprised of genes associated with IFN-γ and activated T cell biology, were initially evaluated in a discovery set of 19 patients with melanoma, and subsequently validated in an independent cohort of 62 melanoma patients [1, 2]. Refined versions of these signatures were independently tested and shown to predict objective response and progression free survival (PFS) in 40 patients with head and neck squamous cell carcinoma (HNSCC) and 33 patients with gastric cancer [3, 4]. Using data combined from 220 pembrolizumab-treated patients across 9 cancer types, a final 18-gene GEP was derived that included immune-related genes related to antigen presentation, chemokine expression, cytotoxic activity, and adaptive immune resistance. The predictive value of the GEP compared favorably with that of PD-L1 immunochemistry when evaluated in an additional independent cohort of PD-L1-unselected HNSCC patients (n=96).

**Conclusions**

The pan-tumor GEP described in this study, typified by indicators of a T cell inflamed microenvironment, captures hallmark characteristics of tumors that are responsive to anti-PD-1 therapy. Our data suggest that these immune-related components are generally necessary, but not always sufficient, for clinical response to pembrolizumab. The GEP represents a potential tumor type-agnostic determinant of response to PD-1 checkpoint blockade, and has undergone analytical validation as a potential diagnostic assay with a clinical utility profile that suggests good performance for maintaining high negative-predictive value and sensitivity [5].

**Acknowledgements**

Joanne Tomassini, Merck & Co., Inc., writing support.

**Trial Registration**

ClinicalTrials.gov identifier NCT01295827, NCT01848834, and NCT02054806.

**References**

1. Ribas A, *et al*: *J Clin Oncol* 2015, **33(suppl)**:Abstr 3001.

2. Shankaran V, *et al*: *J Clin Oncol* 2015, **33(suppl)**:Abstr 3026.

3. Seiwert T, *et al*: *J Clin Oncol* 2015, **33(suppl)**:Abstr 6017.

4. Doi T, *et al*: Presented at ASCO GI Cancers Symposium. 2016.

5. Wallden B, *et al*: *J Clin Oncol* 2016, **34(suppl)**:Abstr 3034.

### P72 Tumor mutational load and T cell inflamed microenvironment are independent determinants of response to pembrolizumab

#### Razvan Cristescu^1^, Robin Mogg^1^, Mark Ayers^1^, Andrew Albright^1^, Erin Murphy^1^, Jennifer Yearley^1^, Xinwei Sher^1^, Xiao Qiao Liu^2^, Michael Nebozhyn^1^, Jared Lunceford^1^, Andrew Joe^1^, Jonathan Cheng^1^, Elizabeth Plimack^3^, Patrick A Ott^4^, Terrill K McClanahan^1^, Andrey Loboda^1^, David R Kaufman^1^

##### ^1^Merck & Co., Inc., Kenilworth, NJ, USA; ^2^MSD China, Beijing, People’s Republic of China; ^3^Fox Chase Cancer Center, Philadelphia, PA, USA; ^4^Dana-Farber Cancer Institute, Boston, MA, USA

###### **Correspondence:** Mark Ayers (mark.ayers@merck.com)

**Background**

Non-synonymous, single-nucleotide variant mutational load (ML) is associated with response to cancer immunotherapies, including CTLA-4 and PD-1/L1 blockade, in some tumor types. A presumptive mechanism is increased tumor antigenicity through generation of neoepitopes not subject to central immune tolerance. A T cell inflamed microenvironment characterized by T cell infiltration, an IFNγ-centric gene-expression profile (GEP), and PD-L1/PD-L2 upregulation, likewise associates with response to PD-1/PD-L1-directed therapies. However, the interaction and independent predictive values of these two distinct aspects of tumor biology have not been well studied. This study evaluates the association between ML and outcome, as well as the independent predictive values of ML and GEP, in pembrolizumab-treated cancer patients across 21 indications.

**Methods**

Whole-exome sequencing and gene-expression profiling were performed on FFPE specimens from patients pooled from two multi-cohort, advanced solid tumor trials (KEYNOTE-028, n=80 and KEYNOTE-012, n=39). The previously defined GEP score was calculated as a weighted sum of normalized expression values for 18 genes. ML and neoantigen analytical approaches included public tools (GATK, MuTect, NetMHC). Statistical testing was pre-specified and adjusted for multiple-testing. Association of ML and GEP was further explored across cancer types using a proprietary database (Moffitt) of tumor gene expression (microarray RNA, n=16,000) and targeted exome (~1300 genes, n=2500) profiles, and TCGA datasets.

**Results**

ML and neoantigen load were both significantly associated with objective response (AUROC=0.76 and 0.78, nominal 1-sided p=0.0036 and 0.0083, respectively). Median numbers of mutations were 180 in responders and 61 in non-responders. The overall response rate (ORR) in all patients was 15 %. At a ML cutoff of 102 (associated with ROC Curve Youden Index), the ORR was 32.3%, with a prevalence of 31.0% and negative-predictive value of 92.8 %. GEP was also significantly associated with objective response (AUROC=0.76, nominal 1-sided p=0.0071). ML and GEP were modestly correlated (r=0.28), consistent with associations observed between ML and GEP in Moffitt (r=0.11) and TCGA databases (r=0.29). When jointly modeled, ML showed significant association with response (p=0.0078) after adjusting for GEP (also significant, p=0.0251).

**Conclusions**

These data validate the utility of ML and GEP across tumor types as independently predictive, complementary biomarkers of response to pembrolizumab. Moreover, these data suggest that tumor antigenicity and T cell infiltration have a non-linear, rather than tightly covariant, relationship. As measures of distinct, yet common features of a PD-1 checkpoint-blockade-responsive tumor, ML and GEP may have utility in characterizing responses to PD-1 blockade and potentially other cancer immunotherapies.

**Acknowledgements**

Joanne Tomassini, Merck & Co., Inc., writing support.

**Trial Registration**

ClinicalTrials.gov identifier NCT01848834 and NCT02054806.

### P73 Next generation techniques for biomarker development, validation and implementation reveal the importance of non-coding RNAs in predicting response to immunotherapy

#### Alex Forrest-Hay

##### Thermo Fisher Scientific, Newburyport, MA, USA

###### **Correspondence:** Alex Forrest-Hay (alex_forrest-hay@affymetrix.com)

**Background**

Cancer immunotherapies are changing treatment paradigms and expanding the therapeutic landscape for cancer patients. The current success of these therapies is well documented, however not all patients respond to immunotherapy and those that do can experience toxicities. There is a growing need to identify predictive and prognostic biomarkers to enhance understanding of the mechanisms underlying the interactions between the immune system and cancer. This presentation will discuss emerging biomarker techniques.

**Methods**

Several new technologies will be showcased in this presentation: (1) The ability to obtain deep transcriptomic data including both coding and non-coding transcripts from a single FFPE section - less than 1ng of RNA and from 50pg of RAN from exosomes; (2) RNA *in situ* methodologies to explore ncRNAs and secreted cytokines/chemokines and quantify their expression levels in the tumor micro-environment; and (3) immuno-assays for assessing soluble checkpoint markers from 25ul of serum/plasma.

**Results**

Data will be showcased that demonstrates the importance of exploring different isoforms of genes and non-coding RNA when searching for biomarkers. A new ncRNA discovered through single cell sequencing and microarray work is a surrogate marker for CD8+ T cell infiltration in a melanoma cohort and correlates with response to nivolumab in this initial small dataset (n=13). Additional data will be available by November. Finally, data will be presented that demonstrates that it is possible to detect "cleaved" or soluble PD-L2 upregulation in the serum of a glioblastoma patient who has undergone immunotherapeutic vaccine therapy via a novel multiplexed Luminex assay.

**Conclusions**

Biomarkers are critical as we move further into the age of combination immunotherapies. It is important to cast the net as broadly as possible and not restrict studies to small subsets of known genes as the likely next generation of clinically useful biomarkers will be non-coding elements or endogenous retroviruses. It is also critical to understand which spliced variant of any given transcript is differentially expressed to fully understand the mechanism that drives any given cancer or response to treatment. New technologies are now available to do this cost effectively from clinical samples such as fine needle aspirates, FFPE tissue sections and blood. This presentation demonstrates the importance of using the latest molecular tools to advance the field of immunotherapy to facilitate the next wave of therapeutic advances.

**References**

1. Das R, *et al*: **Combination Therapy with Anti–CTLA-4 and Anti–PD-1 Leads to Distinct Immunologic Changes In Vivo.** *J Immunol* 2015, **194**:950–959.

### P74 Rapid generation of new specificity MHC tetramers for the detection of antigen-specific T cells using a novel peptide exchange tetramer kit that allows for quantification of peptide exchange

#### Cheryl A. Guyre^1^, Kohei Narumiya^2^, Marc Delcommenne^2^

##### ^1^MBL International, Woburn, MA, USA; ^2^MBL International, Des Plaines, IL, USA

###### **Correspondence:** Cheryl A. Guyre (cheryl.guyre@mblintl.com)

**Background**

Major histocompatibility complex (MHC)-encoded glycoproteins bind peptide antigens through non-covalent interactions to generate complexes that are displayed on the surface of antigen-presenting cells for recognition by T cells. Peptide-binding site occupancy is necessary for stable assembly of newly synthesized MHC proteins and export from the endoplasmic reticulum. During this stage peptides produced in the cytosol compete for binding to MHC molecules, resulting in extensive peptide exchanges.

**Methods**

We have developed a kit based on the principle of peptide exchange to generate novel specificity MHC tetramer reagents in a four-hour assay. While alternate methodologies rely on UV cleavage of exiting peptide on monomeric MHC complexes and a subsequent lengthy tetramerization procedure, we have developed a fully formed fluorescently labeled MHC tetramer that requires only the addition of the peptide of interest and a proprietary peptide exchange factor to complete the reaction.

**Results**

A human HLA-A*02:01 tetramer made from monomer units folded with an irrelevant exchangeable peptide, along with peptide exchange factor, was used with exogenous peptides to generate new specificity tetramers. The efficiency of peptide exchange was quantified using a novel flow cytometry-based sandwich immunoassay using magnetic beads conjugated with an anti-HLA-A,B,C capture antibody and a FITC conjugated antibody reacting against the exiting peptide. Exogenous peptide exchange rates correlated with their theoretical binding affinity to HLA-A*02:01. Tetramers generated using peptides specific for CMV and Influenza demonstrated efficient exchange and detected similar percentages of antigen-specific CD8+ T cells as classically folded MHC tetramers in flow cytometry staining assays.

**Conclusions**

The application of this technology to screening and neoantigen cytotoxic T lymphocyte (CTL) epitope discovery will be discussed.

**References**

1. Reaper DR, Cresswell P: **Regulation of MHC class I assembly and peptide binding.** *Annu Rev Cell Dev Biol* 2008, **24**:343-368.

2. Mayerhofer PU, Tampé R: **Antigen translocation machineries in adaptive immunity and viral immune evasion.** *J Mol Biol* 2015, **427(5)**:1102-1118.

### P75 Selection of indications for JTX-2011 ICONIC clinical trial

#### Heather A Hirsch, Amit Deshpande, Jason Reeves, Jenny Shu, Tong Zi, Jennifer Michaelson, Debbie Law, Elizabeth Trehu, Sriram Sathyanaryanan

##### Jounce Therapeutics, Cambridge, MA, USA

###### **Correspondence:** Heather A Hirsch (hhirsch@jouncetx.com)

**Background**

ICOS (inducible co-stimulator of T cells) is a co-stimulatory molecule expressed primarily on T lymphocytes. Clinical data identified ICOS as a potentially key molecule in providing optimal anti-tumor benefit following anti-CTLA-4 therapy, with preclinical data confirming that engagement of the ICOS pathway plays a significant role in mediating anti-CTLA-4 driven anti-tumor responses. JTX-2011 is an ICOS agonist antibody that will be entering early phase clinical development for the treatment of advanced solid tumors in the ICONIC trial. JTX-2011 is designed to generate an anti-tumor immune response through stimulation of T effector cells and preferential reduction of intratumoral T regulatory (Treg) cells. Efficacy of an ICOS agonist in mouse syngeneic tumor models is greatest in tumors with the highest levels of intra-tumoral ICOS, suggesting a potential predictive biomarker approach for clinical development. In this study we report assessment of indications for enrollment in the clinic trial using *in silico* and IHC analyses across multiple tumor types.

**Methods**

Integrated analyses of RNA, DNA, and clinical data from the Cancer Genome Atlas (TCGA) were performed within multiple indications to understand the context in which ICOS is expressed. Using a proprietary ICOS IHC assay, ICOS expression analysis was performed on a subset of indications based on ranking from *in silico* analysis. ICOS expression was also compared to histology and molecularly defined indication subtypes as well as signatures of immune cell infiltrates to understand context of ICOS positivity.

**Results**

We analyzed ICOS mRNA expression in ~10,000 solid tumors samples across ~30 indications. These data were used to rank indications, and ICOS expression in key indications was orthogonally confirmed using IHC. Consistent with previous data showing ICOS protein expression on Treg cells, ICOS RNA expression significantly correlated with Treg marker expression across multiple indications in TCGA tumor samples. Based on frequency of high ICOS expression, we determined non-small cell lung cancer, head and neck squamous cell carcinoma, triple negative breast carcinoma, gastric adenocarcinoma, and melanoma to be potential indications for JTX-2011 therapy. We further compared ICOS expression to PD-L1 expression to understand if there is a distinct population within an indication that is not a candidate for PD-1/PD-L1 therapy but could benefit from JTX-2011 therapy. A subset of patients in multiple indications exhibit high ICOS levels but low PD-L1 expression.

**Conclusions**

In conclusion, these data support the prioritization of specific tumor types for treatment with JTX-2011 in the ICONIC trial.

### P76 A large-scale analysis of immune cell type proportions in diverse solid tumors

#### Brendan P Hodkinson, Natalie A. Hutnick, Michael E. Schaffer, Michael Gormley

##### Janssen R&D, Spring House, PA, USA

###### **Correspondence:** Brendan P Hodkinson (bhodkins@its.jnj.com)

**Background**

Anticancer immunotherapies eliminate cancer cells by targeting immune cells to promote immune activation or block immune suppression. Ipilumumab and nivolumab, targeting CTLA-4 and PD-1 respectively, have demonstrated dramatic responses in melanoma and lung cancer [1, 2]. However, poor response rates in other types of cancer underscore the need to better understand immunomodulatory mechanisms [3]. In this work, we extend previous methods for inferring immune cell type proportions from gene expression profiles [4], applying them to tumor and normal tissue samples from different tissue types to investigate interactions between tumors and the immune system.

**Methods**

Proportions of 22 immune cell types were inferred from breast, colorectal, lung, and prostate microarrays representing tumors and normal tissue using machine learning. Statistical hypothesis testing was used to investigate immune cell proportion differences (1) between tumor and normal tissues and (2) between tumor types. Prostate tumors were further investigated to determine whether particular cell types (1) were associated with androgen receptor (AR) signaling or (2) could serve as prognostic markers.

**Results**

All tumor types showed significantly higher proportions of M1 macrophages than normal tissues of the corresponding type, a characteristic of early-stage tumors and tumors of patients with better prognosis. Immunosuppressive regulatory T cells (Tregs), were detected more often at higher levels in tumors than normal tissue (significantly in breast and lung). Tumors of the prostate were most similar to normal tissue, possibly reflecting low immune infiltrate. Deeper research into prostate cancer showed (1) tumor AR activity to be positively correlated with plasma cells and negatively correlated with CD8+ T cells, Tregs, and monocytes and (2) indicated that more M1 macrophages (and lower M2/M1 ratios) were associated with better prognosis.

**Conclusions**

Our results indicate that immune cell infiltration differs greatly between (1) healthy and cancerous tissues and (2) different tumor types. With further development, the inference of immune cell type proportions from gene expression profiles may lead to improved prognosis, predictive biomarkers for immunotherapy to assist in patient stratification, and new immunoncology targets by indicating immunosuppressive mechanisms.

**References**

1. Hodi FS, *et al*: **Improved survival with Ipilimumab in patients with metastatic melanoma.** *N Engl J Med* 2010, **363**:711-723.

2. Risvi NA, *et al*: **Activity and safety of Nivolumab.** *Lancet Oncol* 2015, **16**:257-265.

3. Topalian SL, *et al*: **Safety, activity, and immune correlates of anti-PD-1.** *N Engl J Med* 2012, **366**:2443-2454.

4. Newman AM, *et al*: **Robust enumeration of cell subsets from tissue expression profiles.** *Nat Methods* 2015, **12**:453-457.

### P77 Increased antibody and T cell responses to neoepitope site peptides following combination immunotherapy with a complex cell-derived cancer vaccine

#### Tyler Hulett^1^, Shawn Jensen^1^, Carmen Ballesteros-Merino^2^, Christopher Dubay^1^, Michael Afentoulis^1^, Ashok Reddy^3^, Larry David^3^, Bernard Fox^1^

##### ^1^Providence Cancer Center, Portland, OR, USA; ^2^Earle A. Chiles Research Institute at Portland Providence Cancer Center, Portland, OR, USA; ^3^Oregon Health & Science University, Portland, OR, USA

###### **Correspondence:** Tyler Hulett (hulett@ohsu.edu)

**Background**

Here, we report novel correlations between the antibody and T cell responses that develop to tumor-specific molecular targets following complex cancer vaccination. Further investigation of these correlations might lead to new methods of monitoring T cell immune responses via their corresponding antibody responses.

**Methods**

This study involves a complex autophagosome-enriched vaccine (DRibbles) made from 4T1 murine mammary carcinoma cells combined with poly-IC adjuvant. Animals pre-treated with 4T1 DRibbles and poly-I:C benefit from a significant delay in 4T1 tumor growth upon tumor challenge (p < .001), and an increase in CD3+CD8+ tumor infiltrates by multi-spectral immunohistochemistry (p < .001).

**Results**

We designed an array of 150 paired mutant neoepitope and wild-type 4T1 variant site 15mer peptides; this array included all single-nucleotide polymorphism (SNP) variant sites which were an exact match between a published 4T1 sequence and variants identified from sequencing our own 4T1 cell bank. In pooled data from three independent biological experiments, we observed an increase in antibody responses to both the mutant and wild-type variant site peptides in combination vaccinated animals versus adjuvant alone (p=.0002). Surprisingly, we found that these antibody response increases strongly correlated with a higher maximum predicted MHCI binding score from NetMHCpan. To investigate T cell responses, we performed an *ex vivo* screen with a selection of predicted MHCI binding minimal peptides. IL2 expanded splenocytes from vaccinated animals were more likely than those from naïve animals to make an interferon gamma response to the SNP peptides whose parent proteins we had previously identified in the vaccine by mass spectrometry (p=.01). A similar trend was seen for mass spectrometry identified wild-type variant site peptides; these results are from a pool of three independent biological experiments.

**Conclusions**

This study identifies a previously unknown link between predicted MHCI binding affinity and the anti-tumor antibody response, a link which correlates with pooled T cell response data found in screening assays. We are confirming these data with larger scale T cell studies of screening assay hits *ex vivo*.

**Acknowledgements**

Chiles Foundation, Robert W. and Elise Franz, Lynn and Jack Loacker, Wes and Nancy Lematta, M.J. Murdock Charitable Trust, Harder Family, OCTRI-OSLER TL1, the Providence Medical Foundation, and the ARCS Foundation – Portland.

### P78 miRNA a real kid for early recognition for PANcreatic adenocarcinoma (MARKER PAN study)

#### Kumar Jayant, Swati Agrawal, Rajendra Agrawal

##### Sudha Hospital and Merdical Research Center, Kota, Rajasthan, India

###### **Correspondence:** Kumar Jayant (jayantsun@yahoo.co.in)

**Background**

Pancreatic ductal adenocarcinoma constitutes the majority of malignant pancreatic tumours. Pancreatic adenocarcinoma is one of the deadliest cancers for humankind to tackle. More so over, its lethality has increased in the presence of late presentation. The median survival in advanced stage is 5-6 months. The present state CA 19.9 is the most commonly used marker though its sensitivity is questionable regarding terms of any help in early diagnosis. One of the promising evolving entities is miRNA, which has recently come to light, as a possible biomarker and cellular target for pancreatic cancer. The main aim of this study is to create a potential pool of circulatory miRNA panel for diagnosis of pancreatic cancer.

**Methods**

We have evaluated the current literature for various reported miRNA of diagnostic value in the serum by using MeSH terms: pancreatic adenocarcinoma, serum miRNA, and urinary miRNA. Six studies related to miRNA were evaluated in detail and discussed here.

**Results**

Details analysis of various studies have outlined many promising miRNA as potential candidates for the biomarker pool. The most notable ones are miRNA 143, miRNA 223, miRNA 30e, miRNA 204, miRNA 486, miRNA 145, miRNA 150, miRNA 223, miRNA 200, miRNA 21, miRNA 155 are few notable ones. Details of all potential miRNA are outlined in the Table 6.

**Table 6:** **(Abstract P78).** Table showing pool of potential miRNA with diagnostic values

Study	Body Fluid Examined	Sample Size of pancreative cancer	miRNA	Remarks
Debernadi et al. 2016	Urine	59	miRNA 143, miRNA 223, miRNA 30e, 204	Sensitivity-83.3% Specificity-96.2%
Xu et al. 2016	Plasma	192	miRNA 486	AYC-0.861 Sensitivity-75% Specificity-87%
Schultz et al. 2014	Blood	409	miRNA 145, miRNA 150, miRNA 223, miRNA 636	AUC-0.86 Sensitivity-85% Specificity-64%
Liu et al. 2012	Serum	197	miR20a, miRNA 21, miRNA 24, miRNA 25, miRNA 99a, miRNA 99a, miRNA 185, miRNA 191	AUC-0.992 Sensitivity-89% Specificity-100%
Bauer et al. 2012	Blood	345	miRNA 320, miRNA 159, miRNA 225	AUC-0.973
Liu et al. 2012	Plasma	140	miRNA 16, miRNA 21, miRNA 155, miRNA 181a, miRNA 181b, miRNA 196a, miRNA 210, miRNA 199	AUC-95.6

**Conclusions**

miRNA will be a great potential tool to help in the disease diagnosis in the very early stage of a disease. Creating the database of these circulatory miRNAs will be of great potential in creating a panel that will aid in the development of a screening tool.

**References**

1. Debernardi S, *et al*: **Noninvasive urinary miRNA biomarkers for early detection of pancreatic adenocarcinoma**. *Am J Cancer Res* 2015, **5(11)**:3455–3466.

2. Xu J, *et al*: **Plasma miRNAs Effectively Distinguish Patients With Pancreatic Cancer From Controls**. *Ann Surg* 2015, **00(00)**:1.

3. Schultz N, *et al*. **MicroRNA biomarkers in whole blood for detection of pancreatic cancer**. *JAMA* 2014, **311(4)**:392–404.

4. Liu J, *et al*. **Combination of plasma microRNAs with serum CA19-9 for early detection of pancreatic cancer**. *Int J cancer* 2012, **131(3)**:683–691.

5. Bauer AS, *et al*: **Diagnosis of pancreatic ductal adenocarcinoma and chronic pancreatitis by measurement of microRNA abundance in blood and tissue**. *PLoS One* 2012, **7(4)**:e34151.6.

6. Liu R, *et al*. **Serum microRNA expression profile as a biomarker in the diagnosis and prognosis of pancreatic cancer**. *Clin Chem* 2012, **58(3)**:610–618.

### P79 Neutrophil lymphocyte ratio as a biomarker predictive of clinical outcome with nivolumab therapy in RCC

#### Ghayathri Jeyakumar^1^, Seongho Kim^1^, Heejin Kim^1^, Cynthia Silski^1^, Stacey Suisham^1^, Elisabeth Heath^1^, Ulka Vaishampayan^2^

##### ^1^Department of Oncology,^,^Barbara Ann Karmanos Cancer Institute, Wayne State University, Detroit, MI, USA; ^2^Karmanos Cancer Institute, Detroit, MI, USA

###### **Correspondence:** Ghayathri Jeyakumar (jeyakumg@karmanos.org)

**Background**

With numerous therapies available for treatment of RCC, there is a need to evaluate factors predictive of response to enable patient selection. We are reporting a preliminary analysis evaluating the neutrophil lymphocyte ratio (NLR) and response to prior vascular endothelial growth factor (VEGF) inhibitors as factors predictive of response and clinical outcome in RCC patients treated with nivolumab. We also evaluated factors such as race, smoking status and prognostic scoring by MSKCC (Memorial Sloan Kettering Cancer Center) and Heng criteria.

**Methods**

Regulatory approval was obtained. A retrospective chart review of RCC patients at Karmanos Cancer Institute treated with PD-1/PD-L1 inhibitors was conducted. Data was collected on demographics, smoking status, prognostic scoring (MSKCC and Heng), NLR pre and post 4 doses of nivolumab, response to prior therapies and correlated with clinical outcomes on immunotherapy therapies. Univariable and multivariable analyses were performed to evaluate any association with response rate (RR), progression free survival (PFS) and overall survival (OS).

**Results**

Twenty-six patients were evaluated; 25 received nivolumab and 1 received nivolumab and ipilumimab. The median age was 61 years (39-82). 7 patients (27%) were African American (AA) and 12 patients (46%) were smokers. Pretherapy NLR > 6 months and 2 prior therapies. On univariable Cox analysis, pretherapy NLR ≥ 4 was not a significant predictor of response, but approached borderline statistical significance in the prediction of shorter PFS and OS (PFS: p= 0.06, HR 2.532, OS: p=0.058, HR 4.926). Median OS was 2.79 months in the group with NLR ≥ 4 and 18.39 months in the group with NLR > 6 months had a negative impact on PFS and OS (PFS: p= 0.067, HR= 2.98; OS: p=0.028, HR=10.834). In univariable logistic analysis, AA patients had higher risk of non-response (OR 10.5, p=.018), and demonstrated shorter OS (HR15.81, p=0.01) with nivolumab therapy in RCC.

**Conclusions**

NLR is worthy of future investigation as a predictor of clinical outcomes with immune checkpoint inhibition therapy in RCC. Prior therapy that was ≥ 6 months had a negative effect on the PFS and OS with immunotherapy. Validation of this preliminary observation is required in a larger sample size.

### P80 CD47 is overexpressed on Merkel cell carcinoma and a target for SIRPαFc therapy

#### Natalie Vandeven^1^, Natasja Nielsen Viller^2^, Alison O'Connor^2^, Hui Chen^2^, Bolette Bossen^2^, Eric Sievers^2^, Robert Uger^2^, Paul Nghiem^1^, Lisa Johnson^2^

##### ^1^Fred Hutchinson Cancer Research Center, University of Washington, Seattle, WA, USA; ^2^Trillium Therapeutics, Mississauga, ON, Canada

###### **Correspondence:** Lisa Johnson (lisa@trilliumtherapeutics.com)

**Background**

Merkel cell carcinoma (MCC) is an aggressive, neuroendocrine skin cancer with currently no effective, durable treatments for advanced disease. CD47 delivers an anti-phagocytic (“do not eat”) signal by binding SIRPα on the surface of macrophages. While CD47 has recently emerged as a promising drug target in oncology, its role in MCC is unknown. In this study, we evaluated CD47 expression in MCC tumor samples in conjunction with markers of immune infiltrate, Merkel cell polyomaviral (MCPyV) status, and survival. In addition, we assessed the ability of TTI-621 (SIRPαFc), a CD47-blocking decoy receptor, to enhance macrophage-mediated phagocytosis of MCC tumor lines.

**Methods**

**Fig. 41:**
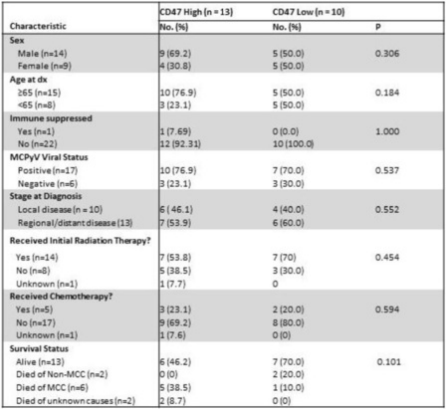
**(Abstract P80).** Patient Demographics

A formalin-fixed paraffin-embedded (FFPE) tissue microarray (TMA) from 23 MCC patients (Fig. 41) was simultaneously stained for DAPI, CD3, CK20, CD47, CD64, CD68, and CD163 and analyzed using multispectral imaging and inForm software (Perkin Elmer). An *in vitro* assay was used to determine the effect of TTI-621 on the phagocytosis of the MCC tumor cell line MCC26. Monocyte-derived macrophages were primed with IFN-γ and co-cultured with violet proliferation dye (VPD) labeled MCC26 cells +/- TTI-621 or control Fc for two hours. Phagocytosis was determined by flow cytometry as the percentage of VPD+CD14+ macrophages.

**Results**

Mean membranous CD47 levels on CK20+ tumor cells ranged from 0.55-17.63 (median 4.5) compared to 0.55-9.55 (median 3.33) on CD3+ cells. Using median CD47 expression on MCC cells, Kaplan-Meier analysis suggested that patients with lower CD47 expression have improved survival rates however, this did not reach statistical significance (p=0.121) in this small number of patients. There were few intratumoral T cells (146 ± 434.9 CD3+/mm^2^) compared to infiltrating macrophages (519±1781 CD68+/mm^2^). Spearman correlation analysis indicated a weak correlation between higher expression of tumor CD47 and decreased numbers of infiltrating T cells (r=-0.352, p=0.012). Additionally, MCPyV+ patients (17/23) had slightly higher mean expression levels of CD47 (6.08), and it was not significantly higher than the small number of MCPyV negative (6/23) patients represented on the TMA (mean = 4.17; p = 0.223).Phagocytosis of MCC26 was increased in the presence of TTI-621, in a dose-dependent manner.

**Conclusions**

CD47 is over-expressed in MCC, regardless of MCPyV status, suggesting that blockade of CD47 by TTI-621 may be particularly beneficial to patients with sparse T cell infiltrate. A phase I dose escalation clinical trial of TTI-621 in patients with solid tumors, including MCC, will initiate enrollment this year. This study will examine changes in the tumor microenvironment in response to TTI-621.

### P81 Withdrawn

### P82 Analytical validation of Ki67/CD8 duplex IHC assay using computational tissue analysis (cTA)

#### Staci J. Kearney, Joshua C. Black, Benjamin J. Landis, Sally Koegler, Brooke Hirsch, Roberto Gianani

##### ^1^Flagship Biosciences, Inc, Westminster, CO, USA

###### **Correspondence:** Staci J. Kearney (skearney@flagshipbio.com)

**Background**

Understanding the spatial relationships between immune cells and tumor cells in the context of cancer tissue is thought to be important for understanding and predicting therapeutic response. Investigation of single immune markers as predictors of clinical response can prove insufficient, necessitating several markers be evaluated for a single patient, which can be confounded by heterogeneity within the tissue block. Multiplex assays that measure two or more analytes in a single tissue section allow direct investigation of spatial relationships while using a fraction of the tissue required for multiple monoplex assays. Fluorescence immunohistochemistry (IHC) multiplex assays provide this advantage, but fluorescence high-complexity testing requires specialized instruments, training, and interpretation of results, potentially preventing their broad adoption by clinical diagnostic laboratories. Chromogenic assays, on the other hand, are standard methods that most of these laboratories have the equipment, expertise, and training to perform.

**Methods**

In this study, Flagship Biosciences analytically validated a chromogenic duplex IHC assay that quantifies Ki67 and CD8 in formalin-fixed, paraffin-embedded non–small cell lung cancer tissues. Similar to analytical validation studies for monoplex IHC assays, this study utilized an appropriate reference method and multiple days of staining. Distinct to this assay validation study, however, was the inclusion of an additional step in the reference standard comparison due to the potential for false-positive and false-negative results linked to the dual-staining methodology. Specifically, a reference method was used to qualify Ki67 and CD8 monoplex IHC assays that were then used as the reference standard to assess duplex assay performance. Five performance criteria were evaluated: reportable range, analytical sensitivity, analytical specificity, accuracy, and precision. These performance criteria were selected based on Clinical Laboratory Standards Institute guidelines.

**Results**

To ensure accurate and consistent measurement of chromogenic staining in the duplex setting, we quantified the percentage of cells positive for Ki67 nuclear staining and/or CD8 membrane staining, using our Computational Tissue Analysis (cTA^TM^) technology. Performance of the Ki67/CD8 chromogenic duplex IHC-cTA assay was considered acceptable for the performance criteria evaluated.

**Conclusions**

The multiplex setting requires additional assay performance assessments due to the complexity of the staining interpretation when multiple IHC stains are present on one tissue section. Flagship’s cTA technology allows for more consistent quantification of individual analytes on dual-stained tissue sections.

### P83 Combining the best of both worlds: immune profiling the tumor microenvironment with RNA and protein biomarkers by fluorescence multiplex RNA *in situ* hybridization and immunohistochemistry

#### Jeffrey Kim, Ming-Xiao He, Bingqing Zhang, Nan Su, Yuling Luo, Xiao-Jun Ma, Emily Park

##### Advanced Cell Diagnostics, Newark, CA, USA

###### **Correspondence:** Jeffrey Kim (jkim@acdbio.com)

**Background**

Recent triumphs in cancer immunotherapy have benefited many cancer patients across multiple malignancies, generating much interest from all sides. At the same time, there is an urgent need to develop predictive biomarkers to identify patients who are most likely to benefit from various immunotherapeutic strategies. While many biomarker analysis technologies are available, most do not provide spatial and cell type-specific information critical for assessing the specific immune cell types with lineage and functional information in the evolving microenvironment of each tumor . Furthermore, multiplexing capabilities are highly desirable in order to obtain comprehensive single cell-level co-expression information and to maximize the use of limited biopsied sample material.

**Methods**

In this study, we demonstrate the development of an improved fluorescence multiplex *in situ* hybridization (ISH) method to detect three RNA biomarkers simultaneously in FFPE and fresh frozen tissues on the Leica BOND RX automated slide staining system. The presented method detects RNA biomarkers in a highly specific and sensitive manner, overcoming the inherent challenge of auto-fluorescence in FFPE tissues. Individual RNA molecules are visualized as distinct bright dots using any fluorescence microscopy or multi-spectral fluorescence imaging system.

**Results**

We applied this technique to archived non-small cell lung cancer FFPE tissues to detect (1) the co-expression of various immune checkpoint markers (such as PD-1, LAG-3, TIM-3) in PD-L1 positive and negative tumor environments and (2) immune functional markers such as cytokines and chemokines in combination with cell lineage markers. We further demonstrate the flexibility of this technique to allow for detection of both RNA and protein biomarkers, where immune checkpoint and functional markers are detected by ISH and cell lineage markers (such as CD3, CD8) by IHC. The data on the co-expression and localization of multiple combinations of markers derived from serial sections of FFPE tissues provide comprehensive information regarding the immune network in each tumor microenvironment.

**Conclusions**

The newly developed multiplex fluorescence RNAscope assay and its combination with IHC presents a powerful tool to interrogate the various cell types and spatial heterogeneity within tumor tissues. Information revealed through simultaneous detection of multiple RNA and/or protein markers may provide new insights to maximize the benefits of current therapeutic approaches. This fully automated assay platform is well suited for developing and validating clinically relevant biomarkers in FFPE tissue.

### P84 Expression and prognostic significance of CD8/CD45RO tumor-infiltrating lymphocytes (TIL) and PD-L1 in cholangiocarcinoma

#### Dae Won Kim, Domenico Copploa, Nishi Kothari, Young doo Chang, Richard Kim

##### ^1^H. Lee Moffitt Cancer Center, Tampa, FL, USA

###### **Correspondence:** Dae Won Kim (daewon.kim@moffitt.org)

**Background**

Cholangiocarcinoma is a malignancy arising from the epithelial cells of the biliary tract with poor prognosis. TILs and PD-L1 have a prognostic impact in various solid tumors. We aimed to investigate TILs and PD-L1 expression and their clinical relevance in cholangiocarcinoma.

**Methods**

Formalin-fixed paraffin-embedded tumor samples from 41 patients with resected and histologically verified cholangiocarcinoma between 1990 and 2015 were identified and immunohistochemically (IHC) stained with anti-CD8, anti-CD45RO and the anti-PD-L1 mouse IgG1 (clone 5H1; Thompson) antibodies. Stained tumor samples were reviewed and enumerated by a GI pathologist. PD-L1 positivity was defined ≥5% of tumor cells with a minimum of 100 evaluable tumor cells. The association between expression of PD-L1, CD8 or CD45RO and survival was investigated using Kaplan-Meier survival and COX proportional hazard regression analyses.

**Results**

The median age of patients was 64 (41-85) with 53% male. 22%, 41% and 37% were stage I, II and III, respectively. 29 patients, 4 patients and 2 patients had R0 resection (microscopically margin-negative), R1 (microscopic residual tumor) and R2 (macroscopic residual tumor), respectively. 11 patients received post-surgical adjuvant treatment. CD8 was positive in 16 (39%), CD8CD45RO (memory CD8 cells) was positive in 5 (12%), and PD-L1 was positive in 19 (46%). With a median follow-up of 21.4 months, tumors with CD8+CD45RO+ TIL has better cancer specific survival (median: unreached vs 41.2mo, HR=0.27, 95% CI: 0.09-0.83, P=0.023) and overall survival (median: unreached vs 21.4mo, HR=0.34, 95%CI: 0.14-0.82, P=0.016) than CD45RO-. The expression of CD8 alone or PD-L1 in tumor was not associated with prognosis. Multivariate analysis showed that CD8+CD45RO+ TIL (HR=0.13, 95% CI: 0.02-0.99, P=0.049) as well as stage, adjuvant treatment and R0 resection were independent predictors of OS.

**Conclusions**

Presence of CD8 memory T cells (CD8+CD45RO+) in tumor microenvironment was associated with significant better clinical outcome, and the expression of PD-L1 on cholangiocarcinoma cells may suggest a potential therapeutic target of anti-PD-L1 antibody therapy in cholangiocarcinoma.

### P85 Immune cell based assay with suspension cells and wall-less DropArray plate

#### Namyong Kim, Melvin Lye, Ee Wan

##### Curiox Biosystems, San Carlos, CA, USA

###### **Correspondence:** Melvin Lye (melvin@curiox.com)

**Background**

The promising advances of immunotherapy in cancer patient suggest a need of parallel innovative solution to facilitate study and manipulation of immune cells. The design of cell based assays for studying patient T or B cells in conventional microtiter plate suffers from key limitations. The large microwell volume of classical microtiter plate is poorly suited to the limited amount of primary immune cells available and especially when a rare subpopulation study is needed. Furthermore, a cell based assay with multi-step staining procedures and immune cells often leads to significant cell loss and require a complex washing process optimization. Here, we present the use of DropArray wall-less microtiter plate for cell based assays with immune cells.

**Methods**

DropArray 384 well plates are designed with hydrophobic/hydrophilic patterning and hold an array of 2 μl drops in which cell based assays can be conducted conveniently and where cells and reagent consumption can be minimized by up to 90%. DropArray wall-less plates employ surface tension to retain suspension cells efficiently on the plate surface during a wash process with a convenient automatic washing station with no optimization required. DropArray plates are used in conjunction with common high content imaging platforms for analysis.

**Results**

DropArray plates display efficient retention of suspension cells such as PBMC, plasmacytoid dendritic cells, and B or T cells in a range of 70 to 90% after multiple wash steps. Among few cell based assays we highlight how the DropArray plate is used to visualize/quantify events of cytotoxic T cells mediating tumor cell killing and where bi-specific antibody efficacy is evaluated with high content imaging platforms in a real time assay.

**Conclusions**

DropArray wall-less plates constitute the next generation microtiter plate for running immune cell based assay with suspension cells.

### P86 Miniaturization of Luminex based multiplex cytokine assay with 96 and 384 DropArray plates

#### Namyong Kim, Melvin Lye, Ee Wan

##### Curiox Biosystems, San Carlos, CA, USA

###### **Correspondence:** Melvin Lye (melvin@curiox.com)

**Background**

Luminex® magnetic bead-based assays have contributed to the expansion of biomarker discovery by providing tools for simultaneous measurement of multiple analytes. However, high cost and sample volume requirements limit the use of this technology in drug discovery and research, especially when analyzing cerebrospinal fluid, tears and limited organic and body fluids. In this poster, we use Curiox’s DropArray technology to reduce Luminex reagents and sample use by 80% while generating similar or enhanced data as obtained by traditional Luminex® methods.

**Methods**

DropArray 96 (DA-96) plate is a wall-less plate that can accommodate 5 to 20 μl drops in each of its 96 circular imprints. DA-96 plate follows standard SLAS/ANSI format, compatible with standard microtiter instruments and follows the same Luminex workflow for magnetic bead-based assays. Each circular hydrophilic area of the DA-96 plate can hold 5μl of each Luminex reagent and samples thus effectively miniaturizing the Luminex assay by at least 80%. DA-96 plate is used sequentially on vortex/orbital shakers, washed in a fully automated station and used in Luminex instruments for data acquisition. We use Millliplex®, Bio-Plex® or Procartaplex™ kits coupled with DA-96 plates for cytokine analysis.

**Results**

Using Millliplex®, Procartaplex™ or Bio-Plex® based kits with DropArray 96 (DA-96) plate and washer, produce interpolated concentration, which correlates 99% with classic high multiplexing methods. Precision and accuracy analysis with DA-96 plates equals or excels conventional Luminex methods with intra assay CV% below 10% and recovery within acceptable range of 70-130%. Range of concentration detection conforms conventional methods with 3.5 to 4 logs range depending on analytes and produce similar sensitivities down to the pg/ml level. DA-96 plate offers excellent reproducibility well-to-well and plate to plate. 2μl and 5μl cytokine supernatant analysis from stimulated immune cells with Milliplex or Bio-Plex based kit DA-Bead method are presented as a functional example. Analysis of 150 serum samples with DA-96 plates indicate enhanced data detection of various analytes when compared to a conventional microtiter plate method.

**Conclusions**

The DA-96 plate and washer is an effective miniaturization platform for Luminex assays that provides similar or enhanced performance reducing costs and sample use by up to 80%. DA-96 plates maximise sample use by requiring only 1/5 of the sample volume. DA-96 plates workflow is similar to standard Luminex workflow. DA-96 plates can be used with Luminex xMAP based kit (Milliplex, Bio-plex, Procartaplex) and any Luminex based instrument.

### P87 Miniaturization of Singulex/Erenna based multiplex cytokine assay with wall-less DropArray 96 plates

#### Namyong Kim, Melvin Lye, Ee Wan

##### Curiox Biosystems, San Carlos, CA, USA

###### **Correspondence:** Melvin Lye (melvin@curiox.com)

**Background**

Recent expansion in biomarker discovery has been possible by the use of bead-based assays and single molecule detection capabilities such as the Singulex assay. Minimal volume requirement with the Singulex methodology requires 50-100μl per well and can be a major bottleneck with limited clinical samples. Furthermore, requirement of multiple plate transfers with the Singulex workflow present a significant delay and obstacle during the workflow when multiple plates are processed simultaneously. Here, we combine DropArray 96 plates (DA-96) and the Singulex assay to achieve similar performance with 80% less volume than the current method.

**Methods**

DA-96 plate is a wall-less plate with 96 circular hydrophilic imprints on a hydrophobic/plastic surface displayed in SLAS/ANSI format, compatible with standard microtiter instruments. Each circular hydrophilic area can hold conventional Singulex reagents such as magnetic beads/antibody/sample/standards up to a total of 20μl volume. Following a highly similar Singulex workflow and using conventional multichannel pipettes, DA-96 plates are used sequentially on vortex shakers and washed in a fully automated wash station. DA-96 to DA-96 plate bead transfer is performed conveniently with a Magnet Transfer Jig™ tool to bypass the need for multiple pipetting steps required in the conventional method. Finally, Beads on DA-96 plate are eluted out in a 384 well plate in a highly convenient manner which avoids centrifugation of beads and laborious plate transfer before acquisition into Singulex reader.

**Results**

Using Singulex Erenna based kits for cardiac Troponin-I, DA-96 plate miniaturized workflow displayed precision and accuracy analysis in line with conventional methods with intra assay CV% below 20% and recovery within acceptable range of 80-120%. Reliable sensitivity LLOQ reached 0.35pg/ml. DA-96 plate and washer offer excellent reproducibility well to well and plate to plate with 20μl of sample volume per well.

**Conclusions**

With similar performance as conventional plates, faster and streamlined workflow, DA-96 plate is the next generation plate for Singulex assay providing similar or improved sensitivity and a smaller sample volume requirement.

### P88 Phenotype, function and gene expression signatures of CD8+ T cells in patients with acute myeloid leukemia (AML)

#### Hanna A. Knaus^1^, Sofia Berglund^1^, Hubert Hackl^2^, Judith E Karp^3^, Ivana Gojo^1^, Leo Luznik^1^

##### ^1^Johns Hopkins University, Baltimore, MD, USA; ^2^Biocenter, Division of Bioinformatics Medical University of Innsbruck, Innsbruck, Tirol, Australia; ^3^Sidney Kimmel Comprehensive Cancer Center, Johns Hopkins University, Baltimore, MD, USA

###### **Correspondence:** Hanna A. Knaus (hknaus1@jhmi.edu)

**Background**

T cell dysfunction in AML remains poorly understood. Therefore, we aimed to genotypically, phenotypically and functionally characterize T cells of AML-patients before and after induction-chemotherapy.

**Methods**

To study transcriptional signatures, FACS-purified peripheral blood (PB) CD8^+^T cells from 6 patients [3 responders (R) and 3 non-responders (NR)] and 4 healthy controls (HCs) were analyzed by microarray. Significantly differentially expressed genes were selected based on >2FC between patient and HC, and p < 0.01. We phenotypically characterized PB T cells from 69 AML-patients before and after induction-chemotherapy, and from 55 HCs, by flow cytometry (FLC). To study AML-blast/T cell interactions, we FACS-purified and *in vitro* cultured T cells and primary AML-blasts from newly diagnosed patients for 3 days. T cells were cultured alone or in co-culture with blasts (1:10) and analyzed by FLC.

**Results**

The transcriptional profile of CD8^+^ T cells at AML diagnosis included significant upregulation of the immune inhibitory receptors genes 2B4, KLRG1, CD160 and TIGIT compared to HCs. In contrast, co-stimulatory receptor genes were downregulated, including CD40LG, CD28 and CD30LG. Ingenuity pathway analysis (IPA) revealed that the co-stimulatory CD28, ICOS and OX40 signaling pathways were downregulated. We performed confirmatory T cell phenotype characterization by FLC in a larger patient cohort (n=69). CD8^+^ T cells were phenotypically senescent (CD27^-^CD28^-^CD57^+^) and %T cells co-expressing 2-4 co-inhibitory receptors (2B4/KLRG1/CD160/CD57) was significantly higher in AML patients compared to HCs. Next, we compared R to NR after induction chemotherapy. R-patients upregulated immune-stimulatory receptor genes like ICOS, whereas NR-patients upregulated immune-inhibitory receptor TIM3; LST1 (inhibits lymphocyte proliferation); TWEAK-APRIL (T cell apoptosis); and CD39 (terminally exhausted T cells). In accordance with these findings, IPA showed enrichment of the co-stimulatory ICOS and OX40 signaling pathways in R-patients. In the confirmatory patient cohort, %senescent T cells and T cells co-expressing 2-4 co-inhibitory receptors was significantly decreased in R-patients (n=52), but unchanged in NR-patients (n=17) compared to pretreatment levels. The co-culture assay showed that the presence of AML blasts also significantly decreased the %primary AML T cells expressing co-stimulatory receptors 41BB, ICOS and OX40, while it increased the frequency of HC T cells expressing 2B4 and CD57.

**Conclusions**

Our study provides insight into AML-associated phenotypical and transcriptional changes in T cells. Our data suggest that that the AML-blasts influence the T cell phenotype and genotype. Response is associated with reversion to HC pattern, whereas NR patient remain in an exhausted/senescent state. Identification of their immune signature will hopefully help to rationally designing future clinical trials of immune-modulating strategies in AML.

### P89 Distinct transcriptional changes in non-small cell lung cancer patients associated with multi-antigenic RNActive® CV9201 immunotherapy

#### Henoch S. Hong^1^, Sven D. Koch^1^, Birgit Scheel^1^, Ulrike Gnad-Vogt^2^, Karl-Josef Kallen^1^, Volker Wiegand^2^, Linus Backert^3^, Oliver Kohlbacher^3^, Ingmar Hoerr^1^, Mariola Fotin-Mleczek^1^, James M. Billingsley^4^

##### ^1^CureVac AG, Tubingen, Baden-Wurttemberg, Germany; ^2^CureVac AG, Frankfurt am Main, Hessen, Germany; ^3^Eberhard-Karls-Universität Tübingen, WSI/ZBIT, Applied Bioinformatics Group, Tubingen, Baden-Wurttemberg, Germany; ^4^Division of Immunology, New England Primate Research Center, Harvard Medical School, Southborough, MA, USA

###### **Correspondence:** Sven D. Koch (sven.koch@curevac.com)

**Background**

RNActive® CV9201 is a novel mRNA-based therapeutic vaccine targeting five lung cancer-associated antigens. A phase I/IIa clinical trial was conducted in 46 patients with stage III or IV non- small cell lung cancer (NSCLC). We sought to comprehensively analyze changes in peripheral blood during the vaccination period to generate hypotheses facilitating the identification of potential biomarkers correlating with differential clinical outcomes post mRNA-based immunotherapy.

**Methods**

Whole-genome expression profiling was performed in peripheral blood mononuclear cells (PBMCs) derived from a subgroup of 22 stage IV NSCLC patients before and after initiation of treatment with CV9201. We used a modular approach to gene expression data analysis based blood transcriptional modules (BTMs), a previously described, sensitive tool for blood transcriptome data analysis. In addition, phenotypic T cell, B cell and NK cell analyses were performed by flow cytometry on the same PBMC samples.

**Results**

Patients segregated into two major clusters based on transcriptional changes post treatment with CV9201. The first group of patients was characterized by the upregulation of an expression signature associated with myeloid cells and inflammation, whereas the other group exhibited an expression signature associated with T and NK cells. Patients with an enrichment of T and NK cell modules exhibited significantly longer progression-free and overall survival compared to patients with an upregulation of myeloid cell and inflammatory modules. Notably, these gene expression signatures were mutually exclusive and inversely correlated. Furthermore, our findings correlated with phenotypic data derived by flow cytometry as well as the neutrophil-to-lymphocyte ratio suggesting changes in the cellular composition of peripheral blood leukocytes after mRNA immunotherapy.

**Conclusions**

We demonstrate non-overlapping, distinct transcriptional signatures associated with differential survival outcomes. These results warrant further validation in controlled, prospective clinical trials for the development of biomarker candidates for mRNA-based immunotherapy.

**Trial Registration**

ClinicalTrials.gov identifier NCT00923312.

### P90 Persistence and turnover of therapy-induced peripheral CD4+ T cell clones in patients with metastatic melanoma upon ipilimumab therapy

#### Yoshinobu Koguchi^1^, Valerie Conrad^1^, William Miller^1^, Iliana Gonzalez^1^, Tomasz Poplonski^1^, Tanisha Meeuwsen^1^, Ana Howells-Ferreira^1^, Rogan Rattray^1^, Mary Campbell^1^, Carlo Bifulco^2^, Christopher Dubay^3^, Keith Bahjat^1^, Brendan Curti^1^, Walter Urba^1^

##### ^1^Earle A. Chiles Research Institute, Providence Cancer Center, Portland, OR, USA; ^2^Robert W. Franz Cancer Research Center, Earle A. Chiles Research Institute, Providence Cancer Center, Portland, Oregon, USA, Portland, OR, USA; ^3^Providence Cancer Center, Portland, OR, USA

###### **Correspondence:** Yoshinobu Koguchi (yoshinobu.koguchi@providence.org)

**Background**

Ipilimumab is a human monoclonal antibody targeting CTLA-4, which is expressed in activated T cells and involved in negative regulation. 22% of patients with metastatic melanoma who received ipilimumab monotherapy are alive 3 years after treatment and the majority of these patients have had a durable response lasting ten years or more [1]. It is important to uncover underlying resistance mechanisms as most patients still fail to benefit from ipilimumab or other immunotherapies.

**Methods**

We assessed whether activation and/or proliferation of peripheral T cells correlated with clinical outcome of patients with metastatic melanoma treated with ipilimumab. PBMCs from patients at baseline and 12 weeks following initiation of treatment participating in a compassionate use trial of ipilimumab [2] were evaluated for ICOS+ or Ki-67+ frequencies on CD4+ and CD8+ T cells, as well as regulatory T cells, using flow cytometry. We conducted T cell receptor (TCR) CDR3 sequencing for sorted bulk CD4+ T cells from baseline, weeks 7 and 12 of ipilimumab treatment to understand their clonal behaviors in patients with metastatic melanoma upon treatment.

**Results**

Ipilimumab treatment significantly increased the frequency of ICOS+ T cells regardless of clinical outcome as previously reported by others [3]. Increased Ki-67+ CD4+ T cells upon ipilimumab treatment (>1.7 fold) was associated with prolonged overall survival. Our results suggest that ipilimumab’s anti-tumor effects may be mediated in part by proliferation of CD4+ T cells. However, some patients with melanoma progression showed increased Ki-67 expression in CD4+ T cells after ipilimumab, implying that CD4+ T cell proliferation alone is not a predictive biomarker. When we tracked destiny of newly emerging CD4+ T cell clones upon treatment (present at weeks 7 but not present at baseline), we found prominent turnover at weeks 12 in patients who progressed upon treatment. In contrast, a patient who showed complete response and later survived longer than 3 years showed persistence of new CD4+ T cell clones upon treatment.

**Conclusions**

Our results suggest that successful treatment with ipilimumab may be, in part, explained by appearance and persistence of potentially tumor-specific CD4+ T cell clones. We will be conducting TCR CDR3 sequencing for DNA extracted from tumor FFPE blocks to see whether clones of interests can be detected in tumor.

**Trial Registration**

ClinicalTrials.gov identifier NCT00495066.

**References**

1. Schadendorf D, *et al*: *J Clin Oncol* 2015, **33**:1889-1894.

2. Koguchi Y, *et al*: *Cancer Res.* 2015, **75**:5084-5092.

3. Ng Tang D, *et al*: *Cancer Immunol Res* 2013, **1**:229-234.

### P91 High frequencies of PD-1+CD8+ T cells are correlated with PD-L1+ circulating tumor cells (CTC) and have predictive/prognostic value in non-small cell lung cancer (NSCLC) patients

#### E-K Vetsika, G Kallergi, Despoina Aggouraki, Z Lyristi, P Katsarlinos, Filippos Koinis, V Georgoulias, Athanasios Kotsakis

##### Laboratory of Translational Oncology, University of Crete, School of Medicine, Heraklion, Greece

###### **Correspondence:** Athanasios Kotsakis (thankotsakis@hotmail.com)

**Background**

The use of antibodies against the negative immune checkpoints, PD-1 and PD-L1 proteins, is an effective therapy for NSCLC patients. In this study, we evaluated the levels of PD-1^+^ and PD-L1^+^ expressing- immune cells (ICs) and CTCs, and their association with the clinical outcome in advanced/metastatic NSCLC.

**Methods**

Peripheral blood was collected from 37 advanced/metastatic chemotherapy-naïve NSCLC patients before treatment. Flow cytometry was performed to enumerate the PD-1 and PD-L1 expressing-ICs with anti-tumor (CD4^+^ and CD8^+^ T cells, B cells, Dendritic cells) and suppressive functions [CD4^+^ and CD8^+^ Treg, Bregs, Myeloid-derived Suppressor cells (MDSC)]. Moreover, PD-1^+^ and PD-L1^+^ CTCs were enumerated by immunofluorescence. Correlation between the levels of PD-1^+^ and PD-L1^+^-expressing ICs and CTCs and their association with overall (OS) and progression-free survival (PFS) was evaluated; high expression of ICs was defined as the percentage of the cells above the mean value.

**Results**

The detection and quantification of all PD-1 and PD-L1-expressing ICs and CTCs, in NSCLC is presented. PD-1 and PD-L1 expression was detected on all tested effector cells and CTCs. In addition, PD-1 and PD-L1 expression was found on all immunosuppressive cells, except Bregs and monocytic-MDSCs. Importantly, the increased percentages of PD-1^+^CD8^+^ T cells were associated with worse response to treatment (p=0.032) and shorter PFS (p=0.023), indicating its clinical relevance. In contrast, the high expression of PD-1 on naive CD4^+^ Treg was correlated with longer PFS (p=0.017), implying that PD-L1/PD-1 axis could be a mechanism leading to Treg apoptosis and hence re-activation of immune system. In multivariate analysis, high percentage of PD-1^+^CD8^+^ T cells was an independent predictor for decreased PFS (HR: 4.1, p=0.0007), while the increased percentages of naive PD-1^+^CD4^+^ Treg cells were independently associated with increased PFS (HR: 3.8, p=0.018). Interestingly, high levels of PDL-1^+^ CTCs were correlated high levels of PD-1^+^CD8^+^T cells (p < 0.04), indicating a likely immune escape mechanism of CTCs. The levels of all other PD-1 and PD-L1 expressing cells were not associated with the clinical outcome.

**Conclusions**

For the first time the current study provides evidence for a possible interaction between the ICs and CTCs in NSCLC patients via the PD-1/PD-L1 axis. Moreover, these results indicate that PD-1 expression, on two distinct ICs with opposite functions, differentially impact NSCLC patient’s clinical outcome.

### P92 Objective and consistent scoring of a PD-L1 complementary diagnostic with Computational Tissue Analysis (cTA™)

#### Nathan T. Martin, Famke Aeffner, Staci J Kearney, Joshua C Black, Logan Cerkovnik, Luke Pratte, Rebecca Kim, Brooke Hirsch, Joseph Krueger, Roberto Gianani

##### Flagship Biosciences, Inc, Westminster, CO, USA

###### **Correspondence:** Nathan T. Martin (nmartin@flagshipbio.com)

**Background**

The PD-1/PD-L1 pathway mediates immunosuppression in the tumor microenvironment. Therapies targeting this pathway have been approved along with corresponding diagnostic assays for selected indications. These assays predict patient responses to therapy by measuring the percentage of tumor cells (Keytruda, Opdivo) or immune cells (durvalumab) that stain positive for PD-L1. The AACR-ASCO-FDA Blueprint Project seeks to facilitate harmonization of PD-L1 tests in order to enable their practical use, with the goal of building a method for objective, consistent, proper test interpretation. In response, we have developed Computational Tissue Analysis (cTA™) approaches that enable objective, consistent assessments of staining. In addition to evaluating staining concurrently in multiple cell types (eg, tumor epithelium, immune infiltrate) and ensuring reproducible scoring, these approaches may help clarify the differences between various PD-L1 assays. The present study evaluated Flagship’s cTA™ approaches to scoring samples stained with the Dako PD-L1 pharmDx (28-8) immunohistochemistry complementary diagnostic assay. The current manual scoring method considers any patient with at least 1% PD-L1–positive tumor cells a candidate for Opdivo treatment. However, a trend toward increased overall survival was observed in patient groups with greater PD-L1 positivity (5% and 10%), suggesting the importance of reliably distinguishing between positivity thresholds.

**Methods**

We developed a cTA™ approach to test the hypothesis that it could provide more consistent scoring around these low thresholds, which are challenging for pathologists. We stained 40 formalin-fixed, paraffin-embedded nonsquamous non–small cell lung cancer samples with the aforementioned Dako assay; a subset of these we stained on consecutive days to evaluate assay and scoring precision. The cTA strategy digitally identified tumor cells and quantified membrane staining intensity consistent with the manufacturer’s scoring guidelines. We also evaluated scoring of PD-L1 expression in immune infiltrate in the same analysis.

**Results**

The cTA™ membrane scoring approach for tumor cells met Flagship’s Clinical Laboratory Improvement Amendments (CLIA) validation criteria, with the manually scored assay as the reference standard. This approach was accurate and provided greater reproducibility of scoring over the dynamic range of PD-L1–positive cell frequencies.

**Conclusions**

This study demonstrated the utility of cTA™ approaches for consistently and objectively scoring PD-L1 expression in tumor cells within the CLIA environment. It also highlighted the challenge pathologists face in differentiating between 1%, 5%, and 10% positivity. cTA™ approaches could be used for consistent scoring of a single assay or to understand differences between assays in performance or relationship to clinical response.

### P93 Antigen recognition avidity dependent miR-155 upregulation in melanoma tumors correlates with increased CD8+ T cell infiltrates

#### Amaia Martínez-Usatorre^1^, Camilla Jandus^1^, Alena Donda^1^, Laura Carretero-Iglesia^2^, Daniel E. Speiser^1^, Dietmar Zehn^3^, Nathalie Rufer^4^, Pedro Romero^1^

##### ^1^Department of Fundamental Oncology, Ludwig Cancer Research Center, University of Lausanne, Epalinges, Vaud, Switzerland; ^2^Department of Fundamental Oncology, Centre Hospitalier Universitaire Vaudois (CHUV), Epalinges, Vaud, Switzerland; ^3^School of Life Sciences Weihenstephan, Technical University Munich, Freising, Bayern, Germany; ^4^Department of Oncology, Centre Hospitalier Universitaire Vaudois (CHUV), Epalinges, Vaud, Switzerland

###### **Correspondence:** Amaia Martínez-Usatorre (amaia.martinez@unil.ch)

**Background**

MicroRNAs (miRs) are noncoding small RNAs that regulate protein expression at the post-transcriptional level in all cells, including those forming the immune system. We previously showed that a single miR, miR-155, promotes effector CD8+ T cell responses in viral infection, vaccination and adoptive cell transfer for tumor therapy in mice [1]. However, little is known yet about miR-155 expression regulation in tumor infiltrating CD8+ T cells.

**Methods**

Our goal here was to study the dynamics of miR-155 expression, i) in tumor specific effector CD8+ T cells from mouse spleen and melanoma tumors expressing high or low affinity antigen ligands, and ii) in *ex vivo* sorted human CD8+ T cell subsets from melanoma patient's blood, non-tumor infiltrated lymph nodes (NLNs), tumor infiltrated lymph nodes (TILNs) and non-lymphoid tumor masses.

**Results**

We report that antigen recognition and T cell avidity are major determinants in the regulation of miR-155 expression in tumor antigen specific CD8+ T cells. Moreover tumor specific mouse and human effector memory (EM) CD8+ T cells from melanoma patients showed increased miR-155 expression levels in melanoma tumors and TILNs compared to T cells from tumor-free areas. In addition, high miR-155 expression levels correlated with increased tumor specific effector CD8+ T cell infiltrates and tumor control in mice. In agreement with these observations in mouse model systems, miR-155 expression levels in human EM CD8+ T cells positively correlated with their frequencies in TILNs and tumor masses in melanoma patients. In addition, high EM CD8+ T cell frequencies correlated with increased overall patient survival.

**Conclusions**

We propose miR-155 expression levels in CD8+ T cells from tumor infiltrated tissues as a surrogate marker for antigen specific CD8+ T cell responses *in situ* and a possible prognostic biomarker in melanoma.

**References**

1. Dudda JC, *et al*: **MicroRNA-155 is required for effector CD8+ T cell responses to virus infection and cancer.** *Immunity* 2013, **38(4)**:742-753.

### P94 Immune activation and prolonged benefit to avelumab (anti-PD-L1) therapy in a patient with metastatic EBV+ gastric cancer

#### Anshuman Panda^1^, Janice Mehnert^2^, Kim M Hirshfield^2^, Greg Riedlinger^2^, Sherri Damare^2^, Tracie Saunders^2^, Levi Sokol^3^, Mark Stein^2^, Elizabeth Poplin^2^, Lorna Rodriguez-Rodriguez^2^, Ann Silk^2^, Nancy Chan^2^, Melissa Frankel^2^, Michael Kane^2^, Jyoti Malhotra^2^, Joseph Aisner^2^, Howard L. Kaufman^2^, Siraj Ali^4^, Jeffrey Ross^4^, Eileen White^2^, Gyan Bhanot^2^, Shridar Ganesan^2^

##### ^1^Rutgers Cancer Institute of New Jersey, Rutgers University, Piscataway, NJ, USA; ^2^Rutgers Cancer Institute of New Jersey, New Brunswick, NJ, USA; ^3^University Radiology, New Brunswick, NJ, USA; ^4^Foundation Medicine, Inc., Cambridge, MA, USA

###### **Correspondence:** Janice Mehnert (mehnerja@cinj.rutgers.edu)

**Background**

The molecular basis for response to immune checkpoint therapy in different cancers is not well understood. In some cases response correlates with high tumor mutation burden, due to environmental exposure to carcinogens (UV exposure/tobacco), or intrinsic DNA repair defects (mismatch repair defects/*POLE* mutations) leading to generation of immunogenic neoantigens. However, some tumors with low mutation burdens are sensitive to immune checkpoint therapy, suggesting other mechanisms of immune activation.

**Methods**

Exceptional clinical benefit was observed in a patient with advanced gastric cancer treated with the PD-L1 inhibitor avelumab. Informed consent was obtained and she was enrolled to the Rutgers Cancer Institute of New Jersey genomic tumor-profiling protocol. Comprehensive genomic profiling was performed using the FoundationOne platform. In situ hybridization was performed to evaluate expression of Epstein Barr Virus (EBV)-encoded RNA. Data from The Cancer Genome Atlas (TCGA) dataset of gastric cancer was analyzed to review associations between EBV status, mutation burden, gene expression based immune signatures and histologic lymphocytic infiltration.

**Results**

This 57-year-old female presented with chemotherapy refractory metastatic gastric cancer causing complete esophageal obstruction, cervical and thoracic lymphadenopathy, and chronic anemia from tumor blood loss. Avelumab 10 mg/kg every 2 weeks was administered in a clinical trial. She experienced tumor reduction after 4 cycles. After 10 cycles, further tumor regression, improved anemia, and improved ability to ingest solid foods with > 15 lb weight gain was observed and continues at 20+ cycles. Genomic analysis of a pre-treatment tumor specimen did not show a high mutation burden or evidence of mismatch repair defects, but was strongly positive for EBV-encoded RNA. Approximately 9% of gastric cancers in the TCGA dataset have evidence of EBV RNA expression. These EBV+ cancers were micro-satellite stable with relatively low mutation burden, but high expression of immune checkpoint genes including PD-1 and PD-L1, and gene expression evidence of immune infiltration.

**Conclusions**

These data suggest that EBV+ gastric cancers are a subset of micro-satellite stable gastric cancers with low mutation burden that respond to immune checkpoint therapy. Tumor EBV expression may be a marker of sensitivity to immune checkpoint therapy that is independent of total mutation burden and microsatellite instability. A phase III trial of avelumab in patients with recurrent gastric cancer is ongoing.

**Acknowledgements**

We acknowledge our patient's participation.

**Trial Registration**

ClnicalTrials.gov identifier NCT01772004.

**Consent**

Written informed consent was obtained from the patient for publication of protocol data in this abstract and accompanying images. A copy of the consent is available for review.

### P95 Immune-enriched NSCLC biopsy tissue microarrays demonstrate that proliferating and checkpoint expressing TIL correlate with positive outcome

#### Anne Monette^1^, Derek Bergeron^1^, Amira Ben Amor^2^, Liliane Meunier^3^, Christine Caron^3^, Antigoni Morou^1^, Daniel Kaufmann^1^, Moishe Liberman^4^, Igor Jurisica^5^, Anne-Marie Mes-Masson^1^, Kamel Hamzaoui^2^, Rejean Lapointe^1^

##### ^1^Université de Montréal, Centre de recherche du CHUM (CRCHUM), Montreal, PQ, Canada; ^2^University of Tunis El Manar II, Tunis, Tunisia; ^3^Institut du Cancer de Montréal (ICM), Centre de recherche du CHUM (CRCHUM), Montreal, PQ, Canada; ^4^Centre Hospitalier de l'Université de Montréal (CHUM), Université de Montréal, Montreal, PQ, Canada; ^5^Ontario Cancer Institute, University of Toronto, Toronto, ON, Canada

###### **Correspondence:** Anne Monette (anne.monette@mail.mcgill.ca)

**Background**

Immune checkpoint (ICP) blockade therapies using anti-PD-1 have elicited positive responses in non-small cell lung cancer (NSCLC) patients, where non-responders are suspected to arise from the downstream expression of additional ICP on effector TIL. In this age of companion diagnostics, during the expanding of the list of predictive biomarkers like PD-L1, it will become invaluable to: 1) define which ICP impact prognosis; 2) determine whether ICP expression is co-dependent, ordered, and can be used to stratify patients; and 3) develop standardized methods for their use in scoring patients ahead of ICP blockade therapies.

**Methods**

An immune-cell-specific biopsy-based tissue microarray (TMA) from untreated NSCLC patients (n=81) was fabricated to profile TIL-ICP using multiplex immunofluorescence (MP-IF). ICP distributions and coexpression were correlated with clinical data, and results were compared to The Cancer Genome Atlas (TCGA) RNA-Seq lung adenocarcinoma (LUAD; n=520) and lung squamous cell carcinoma (LUSC; n=504) datasets. Using microfluidic qRT-PCR, coexpression of ICP with numerous effector TIL genes was assessed in CD8+ and CD4+ TIL isolated from freshly resected untreated tumors, normal tissues, and peripheral blood mononuclear cells (PBMC).

**Results**

TIL that are proliferating and expressing certain ICP positively correlate with the overall survival (OS) of NSCLC patients. From TMA staining data, ICP expression is increased (CTLA4) or decreased (PD-1, CD26, CD57, CD244) relative to patient mortality, and ICP coexpression (CD57-CD39, CD26-CD39) or TIL-ICP expression (CD3-CTLA4, CD3-TIM-3, increased; CD3-CD26, CD3-CD73, decreased) enhance statistical significance. Increased expression of ICP (TIM-3, CD26, LAG-3) or TIL-ICP (CD3-TIM-3, CD3-BTLA, CD3-LAG-3, CD3-CD26, CD3-CD39) correlates with improved OS. From both TMA results and TCGA datasets, ICP subset expression is observed to have heightened importance relative to OS at earlier time points, suggesting an ordered accruing of ICP by TIL. Hierarchical cluster analysis reveals ICP groupings (CD26, CD39, TIM-3; TIGIT, CTLA4, PDCD1; CD244, CD57) reflected by qRT-PCR results also revealing ICP distributions on CD8+ and CD4+ TIL and PBMC. TCGA datasets also provide evidence that overall ICP expression positively correlates with OS, and that advanced cancer stages have lowered ICP expression. Finally, ICP expression analysis defines an ordered build of ICP on NSCLC TIL.

**Conclusions**

This multi-cohort analysis performed using an array of techniques has resulted in the discovery of TIL ICP expression having the greatest benefit for different NSCLC subtypes. ICPs are coexpressed, and their early, stepwise acquisition may be an important determinant of OS. In this era of personalized medicine, use of ICP MP-IF panels may better stratify patients for ICP blockade therapies.

### P96 High sensitivity detection of low expressing interleukins and interferons for biomarker research analysis from FFPE samples using multiplexed NGS

#### Ann Mongan, Yuan-Chieh Ku, Warren Tom, Yongming Sun, Alex Pankov, Tim Looney, Janice Au-Young, Fiona Hyland

##### Thermo Fisher Scientific, South San Francisco, CA, USA

###### **Correspondence:** Ann Mongan (ann.mongan@thermofisher.com)

**Background**

There is growing evidence supporting the association of tumor infiltrating lymphocytes, inflammatory signaling molecules, and drug sensitivity to cancer checkpoint blockade therapy. At the same time, the exact markers that are predictive of response for each therapeutic agent are still the subject of active investigations. To address the need for better understanding of the effect of different T cell subsets, antigen presentation, and tumor killing, gene expression profiling presents an attractive means to simultaneously evaluate the tumor microenvironment and cancer cells. Furthermore, as most of the samples available for drug sensitivity research studies are derived from formalin-fixed paraffin-embedded (FFPE) slides with typically low RNA quality, target sequencing offers a cost-effective solution that provides significantly higher sensitivity and specificity over whole transcriptome sequencing or other gene expression profiling methods.

**Methods**

Here we report the results of a 395-gene expression panel profiling FFPE non-small cell lung cancer (NSCLC) and melanoma tumor research samples. The panel uses Ion AmpliSeq^TM^ technology to measure the expression of genes involved in T cell activation, markers of different leukocyte subsets, antigen presentation, and tumor characteristics (proliferation, adherence/migration, epithelial-to-mesenchymal transition, etc).

**Results**

Gene expression measured by the panel stratified NSCLC samples into groups that are consistent with histopathology classification and tumor infiltrating levels provided by pathologists. The panel offers reproducibility between replicates and high sensitivity of detection for low expressing genes such as IL-2, IL-10, IL-21, and IFNG, among others. We further demonstrated excellent concordance between fresh frozen and FFPE samples (correlation >0.95) as well as concordance with TaqMan^(R)^ qPCR. With a series of limiting dilution experiments between two cell lines, the assay showed broad dynamic range and linearity up to 200 fold. This Oncomine^TM^ Immune Response Research Assay* is accompanied by a Torrent Suite™ software package that provides run quality metrics, normalized gene expression, and hierarchical clustering among multiplexed samples.

**Conclusions**

In summary, the current gene panel offered an accurate and accessible tool for evaluating biomarkers that may be relevant to cancer immunotherapy.*For Research Use Only. Not for use in diagnostic procedures.

### P97 Validation of a custom RNA-Seq approach to cancer immune profiling

#### Jeff Conroy^1^, Carl Morrison^1^, Sean Glenn^2^, Blake Burgher^2^, He Ji^2^, Mark Gardner^2^, Ann Mongan^3^, Angela R Omilian^1^

##### ^1^Roswell Park Cancer Institute, Buffalo, NY, USA; ^2^OmniSeq, LLC, Buffalo, NY, USA; ^3^Thermo Fisher Scientific, South San Francisco, CA, USA

###### **Correspondence:** Jeff Conroy (jeff.conroy@roswellpark.edu)

**Background**

Immune checkpoint blockade with monoclonal antibodies directed at the inhibitory immune receptors has emerged as a successful treatment for cancer patients. Evaluation of tumor checkpoint blockade by IHC is subjective, not reproducible, and importantly not scalable. We have designed a custom 362 gene RNA-Seq immune profiling panel that uses a multi-analyte algorithmic analysis (MAAA) to evaluate checkpoint blockade, tumor infiltrating lymphocytes (TILs), and cytokine/chemokine interactions.

**Methods**

269 cancer samples of diverse histologies with both frozen and FFPE samples were evaluated by RNA-Seq with a custom 362-gene Ion AmpliSeq Immune Response Profiling Assay using the Ion Chef™ and Proton. RNA-Seq analysis was performed with the Torrent Suite™ followed by normalization. All samples were also included in a custom TMA and IHC was performed for 61 different markers for various immunotherapy targets. Custom TaqMan assays were also performed for each of the 61 different markers.

**Results**

Correlation of frozen and FFPE samples was directly related to level of expression at the gene level. High expression genes showed a better correlation than low expression genes. RNA-Seq and TaqMan results were highly correlated for all 61 genes evaluated. As compared to IHC the results for RNA-Seq were continuous rather than bimodal and allowed for a much more detailed analysis of the immune repertoire. Frequent difficult-to-interpret results for IHC for PD-L1 and other markers were easily interpreted by RNA-Seq, while subsets of TILs were identified allowing for a detailed analysis of this important parameter.

**Conclusions**

Immune profiling by RNA-Seq allows for the identification of samples with over-expression of multiple genes involved in checkpoint blockade, TILs, and cytokine/chemokine interactions. Subjectivity of interpretation of IHC as well as the lack of scalability will require a more high-throughput approach such as RNA-Seq. Our results show that such an approach is feasible with FFPE specimens and is more accurate and reproducible than IHC.

### P98 An algorithmic approach to cancer immune profiling

#### Jeff Conroy^1^, Wiam Bshara^1^, Omilian Angela^1^, Blake Burgher^2^, He Ji^2^, Sean Glenn^2^, Carl Morrison^1^, Ann Mongan^3^

##### ^1^Roswell Park Cancer Institute, Buffalo, NY, USA; ^2^OmniSeq, LLC, Buffalo, NY, USA; ^3^Thermo Fisher Scientific, South San Francisco, CA, USA

###### **Correspondence:** Carl Morrison (carl.morrison@roswellpark.org)

**Background**

Immune checkpoint blockade with monoclonal antibodies directed at the inhibitory immune receptors has emerged as a successful treatment for cancer patients. Evaluation of tumor checkpoint blockade by IHC is subjective, not reproducible, and importantly not scalable. We have designed a custom 362 gene RNA-Seq immune profiling panel that uses a multi-analyte algorithmic analysis (MAAA) to evaluate checkpoint blockade, tumor infiltrating lymphocytes, and cytokine/chemokine interactions. Additionally, we have previously profiled several hundred samples that are used for ranking of expression of each gene.

**Methods**

269 FFPE cancer samples of diverse histologies were evaluated by RNA-Seq with a custom 362-gene Ion AmpliSeq Immune Response Profiling Assay using the Ion Chef^TM^ and Proton. RNA-Seq analysis was performed with the Torrent Suite^TM^ followed by normalization. Results for each sample were then evaluated with a MAAA to provide a prediction score for 61 known immunotherapy drugs. Predictions were reported for a continuous scale of 1-100 and interpreted as low, neutral, or high. All samples were also included in a custom TMA and IHC was performed for known targets of various immunotherapies when available.

**Results**

At least one over expressed immunotherapeutic target was identified in more than 50% of the samples. By evaluating upstream and downstream mediators for each overexpressed target at least one high prediction score was identified in almost one-third of the samples. Not surprisingly the class of immune recognition, including checkpoint blockade, was the most frequently identified group of high prediction scores. High prediction scores were also identified for immune therapy drugs for the classes of immune inhibition (eg IDO1), tumor targeting mAbs (eg CD56), and microbe-associated (TLR7).

**Conclusions**

Profiling by RNA-Seq allows for the identification of samples with over expression of multiple genes involved in the tumor immune repertoire. By applying a MAAA to these results in the context of a known database of results we can provide a prediction score that guides selection of various immune therapies, including more than just checkpoint blockade. As immune therapy moves from treatment of last resort to first and second line treatment an accurate scalable interrogation of the total tumor immune microenvironment will be required.

### P99 PD-L1, PD-L2 and PD-1 expression in metastatic melanoma: correlation with tumor infiltrating immune cells and clinical outcome

#### Joseph M. Obeid^1^, Gulsun Erdag^2^, Mark E Smolkin^3^, Donna H Deacon^1^, James W Patterson^4^, Lieping Chen^5^, Timothy N Bullock^4^, Craig L Slingluff^6^

##### ^1^Department of Surgery, University of Virginia, Charlottesville, VA, USA; ^2^Department of Pathology, Johns Hopkins Medicine, Baltimore, MD, USA; ^3^Department of Public Health Sciences, University of Virginia, Charlottesville, VA, USA; ^4^Department of Pathology, University of Virginia, Charlottesville, VA, USA; ^5^Department of Immunobiology, Yale University, New Haven, CT, USA; ^6^University of Virginia, Charlottesville, VA, USA

###### **Correspondence:** Joseph M. Obeid (jmo3s@virginia.edu)

**Background**

The expression of PD-L1 in melanoma metastases limits immune control of tumor progression. Reliable biomarkers of patient survival, and response to treatment, are critical for patient care and selection. Yet the prognostic ability of PD-L1, or highly related PD-L2, remain controversial. We hypothesized that the expression of PD-L1 and PD-L2 by melanoma cells would correlate with both immune cell infiltration and patient survival, independent of checkpoint blockade therapy.

**Methods**

Tissue microarrays of metastatic melanoma samples from 147 patients were evaluated (median follow-up: 19 months). None had been treated with PD-1/PD-L1 blockade. Cancer vaccines had been administered to 49 of the patients (33%). Melanoma cells were assessed by immunohistochemistry for surface and cytoplasmic expression of PD-L1 (clone 5H1) and PD-L2 (R&D Systems, AF1224). Immune cells were enumerated with stains for PD-L1, PD-1, CD8, CD45, CD4, CD3, CD163, CD20, CD138, and FoxP3. Relationships between the proportions of PD-L1 and PD-L2 expressing tumor cells with immune cell counts were assessed (by Spearman correlation), and with immune cell distribution (Immunotype, by Wilcoxon rank sum tests). Associations with patient survival were evaluated using Kaplan-Meier curves and Log-rank tests. P values less than 0.05 were considered significant.

**Results**

Surface expression of either PD-L1 or PD-L2 by melanoma cells correlated significantly with increasing intratumoral densities of immune cells expressing each of the following: CD45, CD3, CD4, CD8, PD-1 and FoxP3 (p≤0.001). PD-L1 expression was more common in tumors of patients who had previously participated in melanoma vaccine trials (p = 0.034). Diffuse infiltration (Immunotype C), as opposed to infiltration limited to the perivascular spaces (Immunotype B), was associated with increased PD-L1 (p = 0.058) and PD-L2 (p=0.033) expression by melanoma cells. Expression of PD-L2 on ≥5% of tumor cells was associated with improved overall survival (p=0.043), and the simultaneous positive expression of both PD-1 ligands was even more strongly associated with improved survival (p=0.005, Figure).

**Conclusions**

Both PD-L1 and PD-L2 are markers of immune infiltration. PD-L2 alone, or in combination with PD-L1, is a marker for prognosis in metastatic melanoma patients. The prognostic associations of the combination of PD-L1 and PD-L2 support future studies of the predictive value of these ligands in the setting of combination checkpoint blockade therapy.

### P100 PD-L1 is expressed in inflamed non-small cell lung cancer (NSCLC) specimens and its expression predicts longer patient survival, despite co-expression of other checkpoint molecules

#### Joseph M. Obeid^1^, Gulsun Erdag^2^, Donna H Deacon^1^, Craig L Slingluff^3^, Timothy N Bullock^4^

##### ^1^Department of Surgery, University of Virginia, Charlottesville, VA, USA; ^2^Department of Pathology, Johns Hopkins Medicine, Baltimore, MD, USA; ^3^University of Virginia, Charlottesville, VA, USA; ^4^Department of Pathology, University of Virginia, Charlottesville, VA, USA

###### **Correspondence:** Joseph M. Obeid (jmo3s@virginia.edu)

**Background**

PD-L1 expression is induced by IFNγ, providing a feedback mechanism to control inflammation locally. There are conflicting data concerning its prognostic implication in patients with NSCLC. We hypothesized that PD-L1 is a positive prognostic indicator in NSCLC and that tumors expressing PD-L1 also express other immune checkpoint molecules and are highly infiltrated by T cells.

**Methods**

Tissue microarrays (TMA) were constructed using two 1 mm cores from formalin-fixed paraffin embedded surgical specimens of 151 NSCLC (90 adenocarcinomas (AdCA), 58 squamous cell, and 3 mixed histology). Patients were not treated with PD-1/PD-L1 antibodies, 93% had stage I/II disease (median follow-up: 27 months). TMAs were evaluated by immunohistochemistry for PD-L1 (clone 5H1, membranous or cytoplasmic staining), GAL9, CD155, and PD-1 on tumor cells and CD8, FoxP3, PD-1 and LAG3 on immune cells. PD-L1 expression was recorded as the percent of staining tumor cells. GAL9, CD155, and PD-1 were assessed by intensity of staining on tumor cells (scale 0-4). Stained immune cells were enumerated per core. Overall survival (OS) was assessed as a function of PD-L1 expression using Cox-proportional hazard. Correlations between PD-L1 expression and other markers were assessed using Pearson correlation coefficients. The Cancer Genome Atlas (TCGA) data were explored for validation of co-expression profiles using Fisher exact tests.

**Results**

Increased percentages of PD-L1^+^ tumor cells were associated with longer OS (univariate: p=0.03 and multivariate accounting for stage, age, histology, CD8 counts and tumor grade: p=0.04). PD-L1 expression also correlated with increasing CD8(p=0.002), FoxP3(0.013), LAG3(0.007) or PD-1(p=0.049) expressing lymphocytes and with the expression of PD-1, GAL9 and CD155 on tumor cells (p≤0.02). Tumors with high PD-L1 and CD8 expression (21%) also highly expressed inhibitory markers (FoxP3, LAG3, CD155 and GAL9, Figure). Similar significant correlations with PD-L1 mRNA expression were found in the TCGA cohort of 517 AdCA patients with PD-1 and CD8, and with checkpoint molecules LAG3 , TIM3, and PD-L2, as well as the TIM3 ligand Gal9 (Fig. 43).

**Fig. 42:**
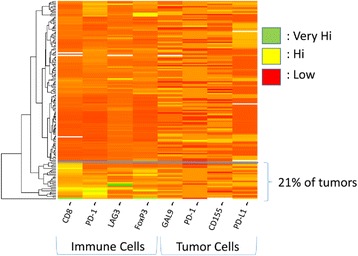
**(Abstract P100).** The expression of different immune markers. Heatmap depicts protein expression intensities as determined by IHC in the 151 NSCLC tumors

**Fig. 43:**
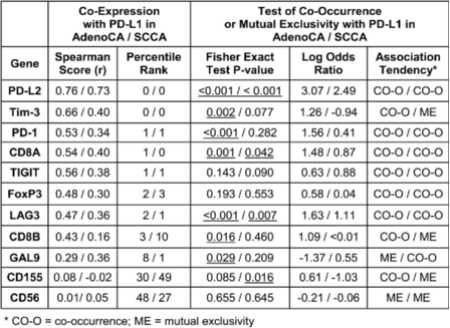
**(Abstract P100).** Genes expressed in association with PD-L1 in NSCLC. Gene expression analysis in the 517 adenocarcinoma and 501 squamous cell carcinoma specimens from the TCGA provisional database

**Conclusions**

Tumor cell expression of PD-L1 is a favorable prognostic indicator and correlates with CD8^+^ T cell infiltration and co-expression of other inhibitory checkpoint molecules and ligands. The simultaneous presence of additional tumor protective signals in these tumors may influence their response to PD-1/PD-L1 blockade. Patterns of co-expression of checkpoint molecules may guide selection of combination checkpoint blockade therapy.

**Acknowledgements**

Results presented are in part based upon data generated by the TCGA Research Network (http://cancergenome.nih.gov) (Cerami E, et al., PMID:22588877; Gao J, et al., PMID:23550210).

### P101 Enhanced vaccine-induced T cell responses observed with ipilimumab (anti-CTLA-4) treatment in a nonhuman primate pharmacodynamic model

#### John T. Loffredo, Raja Vuyyuru, Sophie Beyer, Vanessa M Spires, Maxine Fox, Jon M Ehrmann, Katrina A Taylor, Alan J Korman, Robert F Graziano

##### Bristol-Myers Squibb, Princeton, NJ, USA

###### **Correspondence:** John T. Loffredo (john.loffredo@bms.com)

**Background**

Currently, the preclinical assessment of immuno-oncology (I-O) agents relies heavily on murine tumor efficacy models, typically necessitating surrogate antibodies. Although nonhuman primate studies enable the use of human clinical candidate antibodies, such studies rely on limited pharmacodynamic (PD) markers. To more thoroughly evaluate the ability of I-O therapies to impact T cell immunity, we took advantage of the limited major histocompatibility complex (MHC) diversity in Mauritian cynomolgus macaques (MCMs) and utilized a highly immunogenic vaccine containing known MCM T cell epitopes. This design enabled longitudinal tracking of vaccine-elicited responses using antigen-specific T cell readouts. We then evaluated the effects of ipilimumab (anti-CTLA-4) in this nonhuman primate PD model.

**Methods**

Pre-selected MCMs with specific MHC class I alleles that restrict known CD8^+^ T cell epitopes were vaccinated intramuscularly with two non-replicating adenovirus serotype 5 (Ad5) viral constructs, one encoding simian immunodeficiency virus (SIV) Gag protein and the second encoding SIV Nef protein. Following vaccination, MCMs received intravenous administration of either saline as control or ipilimumab (anti-CTLA-4) at 10 mg/kg. Vaccine-induced SIV-specific T cell responses were longitudinally evaluated via flow cytometry, including the use of peptide loaded MHC class I tetramers and IFN-gamma ELISPOT assays.

**Results**

All vaccinated MCMs generated detectable levels of Gag- and Nef-specific T cell responses that typically peaked 2 to 3 weeks post-vaccination with a consistent immunodominance hierarchy. Addition of a single dose of ipilimumab resulted in robust augmentation of vaccine-induced CD8^+^ T cell responses both at the peak of the vaccine response and after the vaccine response waned (>6 weeks post-vaccination). Enhanced Ki-67 expression was also detected on bulk CD8^+^ and CD4^+^ T cells, signifying increased proliferative capacity with ipilimumab treatment.

**Conclusions**

Using this nonhuman primate PD model, we observed that the administration of ipilimumab enhanced the magnitude of vaccine-induced Ag-specific T cell responses. The ability to demonstrate such PD effects in monkeys allows for the testing of human clinical candidate antibodies in pharmacologically relevant models. This vaccine system can be used to provide early proof-of-concept data as well as aid in lead candidate selection by comparing antibodies targeting different epitopes or expressing different Fc domain formats (e.g., human IgG1 vs human IgG4). The described findings highlight the feasibility of this approach to investigate the impact of immunomodulatory antibodies/therapies on T cell immunity, particularly at an antigen-specific T cell level, and warrant the study of additional I-O agents.

### P102 Multispectral immunofluorescence as a novel and complementary method of characterizing tumor-infiltrating lymphocytes (TILs) in early stage breast cancer (ESBC)

#### David Page^1^, Katherine Sanchez^2^, Carmen Ballesteros-Merino^1^, Maritza Martel^2^, Carlo Bifulco^3^, Walter Urba^4^, Bernard Fox^3^

##### ^1^Earle A. Chiles Research Institute, Providence Portland Medical Center, Portland, OR, USA; ^2^Providence Portland Medical Center, Portland, OR, USA; ^3^Robert W. Franz Cancer Research Center, Earle A. Chiles Research Institute, Providence Cancer Center, Portland, Oregon, USA; ^4^Earle A. Chiles Research Institute, Providence Cancer Center, Portland, OR, USA

###### **Correspondence:** David Page (david.page2@providence.org)

**Background**

In ESBC, TILs by gold-standard H&E Salgado criteria are prognostic for survival. Salgado criteria employ a single-slide “average” pathologist estimation of stromal TILs and excludes analysis of immune cell “hotspots.” Alternatively in colorectal cancer, the immunoscore methodology is prognostic for survival but employs CD8+ assessment of immune cell “hotspots.” Recently, immune cell hotspots predicted response to anti-PD-L1 in breast cancer. Therefore, we wished to preliminarily evaluate both gold-standard and immune hotspot strategies for scoring TILs in ESBC, and to evaluate the utility of multispectral immunofluorescence (fluorescent mIHC), which allows for single-slide quantification of numerous immune cell subsets.

**Methods**

From 8 sequentially diagnosed ESBC specimens, FFPE-preserved diagnostic core biopsies and subsequent excision specimens were obtained via an IRB-approved biospecimen protocol. Adjacent slides were submitted for routine H&E and fluorescent mIHC. H&E TILs were scored by a trained breast pathologist using Salgado criteria. Fluorescent mIHC was conducted using the PerkinElmer Vectra platform using a validated antibody panel (CD3, CD8, FoxP3, CD163, PD-L1, cytokeratin, and DAPI) [1]. To recapitulate the Salgado “TIL average” scoring method using fluorescent mIHC, 9 randomly-selected tumor-bearing 20x fields were selected from a single slide, then stromal TIL scores were averaged across these fields. To recapitulate the immunoscore “immune hotspot” methodology, CD8+ high-density areas were visually identified at 4x magnification, then subsequently scored at 20x magnification.

**Results**

**Fig. 44:**
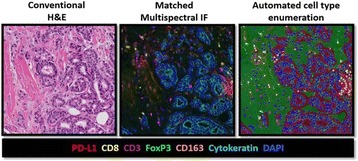
**(Abstract P102).** Representative H&E and IF Images

**Fig. 45:**
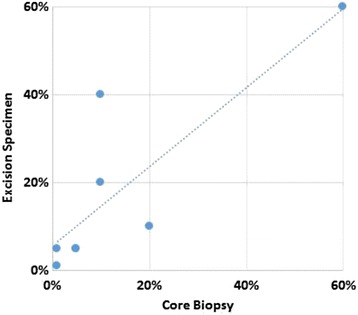
**(Abstract P102).** Correlation of H&E Salgado score, core biopsy versus excisional specimen

Representative images are shown in Fig. 44; all 16 specimens were high quality without significant crush artifact or nonspecific staining. Cytokeratin staining delineated stroma from tumor, allowing for automated quantification of both stromal and intratumoral TILs by fluorescent mIHC. H&E Salgado TIL scores ranged from 0-60%; core biopsy and excisional H&E TIL scores were highly correlated (r^2^=0.7, Fig. 45). By the “TIL average” fluorescent mIHC method, core biopsy and excisional biopsy immune cell counts were highly correlated (Table 7). By the “immune hotspot” fluorescent mIHC method, at least one hotspot was identified in each of the 16 specimens; however, core biopsy and excisional hotspot immune cell counts were only weakly correlated.

**Table 7:** **(Abstract P102).** Correlation of IF cell densities, core biopsy versus excisional specimen

Cell Type	“TIL average” method, r^2^	“Immune hotspot” method, r^2^
CD3+	0.67	0.04
CD8+	0.63	0.06
PD-L1+	.042	0.34
PD-L1+ CD3+	0.74	0.29
CD163+	0.61	0.43

**Conclusions**

In ESBC, fluorescent mIHC provides complementary data on TIL phenotype that may ultimately prove useful in personalizing immunotherapy. A “TIL average” strategy of assessing TILs by fluorescent mIHC may more stably characterize individual tumors compared to an “immune hotspot” approach; however, further evaluation is warranted.

**References**

1. Feng Z, Puri S, Moudgil T, *et al*: **Multispectral imaging of formalin-fixed tissue predicts ability to generate tumor-infiltrating lymphocytes from melanoma**. *J Immunother Cancer* 2015, **3**:47.

### P103 Changes in uveal melanoma immune infiltrate in response to checkpoint blockade

#### Sapna P. Patel, Mariana Petaccia De Macedo, Yong Qin, Alex Reuben, Christine Spencer, Michele Guindani, Roland Bassett, Jennifer Wargo

##### University of Texas MD Anderson Cancer Center, Houston, TX, USA

###### **Correspondence:** Sapna P. Patel (sppatel@mdanderson.org)

**Background**

Uveal melanomas are a subset of melanomas that contain few genomic mutations, thereby generating few neo-epitopes, and making them of low immunogenic potential. What is unknown is how systemic treatment for metastatic disease changes the immunogenicity of metastatic uveal melanomas. Previous work has demonstrated the immune infiltrate in cutaneous melanomas changes in response to treatment with checkpoint blockade. We undertook a pilot study to describe the changes in uveal melanoma immune infiltrate in response to treatment with systemic checkpoint blockade.

**Methods**

Twenty-two uveal melanoma patients who had received checkpoint blockade for metastatic disease, and who had archival pathologic tissue, were identified. Formalin-fixed paraffin-embedded tissue sections were obtained on available cases, and bleaching protocols were utilized to reduce heavy melanin pigmentation. Immunohistochemistry was performed using validated antibodies for CD3, CD8, CD68, FOXP3, PD-1, and PD-L1.

**Results**

Eleven uveal melanoma patients had at least one time point of tumor tissue collected and evaluated, most commonly before treatment initiation. Nine patients had at least two time points collected and evaluated, some while on treatment, others at progression of disease. In a representative case, a pre-treatment metastatic liver biopsy showed low level of CD8+ lymphocytes infiltrating the tumor. After 4 doses of ipilimumab, with best overall clinical response of disease progression, the patient had another liver biopsy. This tissue sample showed a notable increase in CD8+ lymphocyte infiltration via immunohistochemistry compared to the pre-treatment sample. This is consistent with similar data from cutaneous melanoma patients receiving checkpoint blockade, where CD8+ infiltrates at progression is lower than during treatment response, but higher than at baseline. Full details of all cases will be presented at the SITC Annual Meeting.

**Conclusions**

Immune infiltration does change in metastatic uveal melanoma in response to systemic checkpoint blockade. Some patients did experience an increase in infiltrating CD8+ T lymphocytes while on immunotherapy. The change in immune infiltrate did not, however, correlate with clinical response to treatment. Future analysis includes an evaluation for immune suppressive molecules, such as indoleamine 2,3-dioxygenase and transforming growth factor-beta, and evaluation of immune infiltration in response to combination checkpoint blockade as well as to targeted therapy.

**Acknowledgements**

The authors would like to acknowledge Zac Cooper, PhD, for his time at MD Anderson crafting the grant that funded this research.

### P104 Development of an automated brightfield duplex IHC for simultaneous detection of PD-L1 and CD8 on lung carcinoma and tonsil FFPE tissue sections

#### Adriana Racolta, Brian Kelly, Tobin Jones, Nathan Polaske, Noah Theiss, Mark Robida, Jeffrey Meridew, Iva Habensus, Liping Zhang, Lidija Pestic-Dragovich, Lei Tang

##### ^1^Ventana Medical Systems, Inc., Tucson, AZ, USA

###### **Correspondence:** Adriana Racolta (adriana.racolta@roche.com)

**Background**

Behavior of various tumor-infiltrating immune cells in the tumor environment correlates with patient responses to therapies. Particularly, the localization of CD8+ T cells within and around a tumor mass containing immune and tumor cells expressing the programmed death-ligand 1 (PD-L1) checkpoint marker is positively correlated with response to immunotherapy targeting PD-L1 blockade. Simultaneous detection of CD8+ T cells and PD-L1+ cells could become a valuable diagnostic tool. We developed a fully automated duplex immunohistochemistry (IHC) assay to detect these two biomarkers on a single formalin-fixed, paraffin-embedded (FFPE) tissue section using VENTANA BenchMark ULTRA stainers and brightfield microscopy.

**Methods**

The duplex IHC assay uses anti-PD-L1 (SP263) and anti-CD8 (SP239) rabbit monoclonal primary antibodies and goat anti-rabbit secondary antibodies conjugated to either horseradish peroxidase (HRP) or alkaline phosphatase (AP). The detection and amplification of signal is achieved upon activation of tyramide- or quinone methide-conjugated chromogens by the corresponding enzymes and covalent binding of chromogens. The detection of PD-L1, with a purple chromogen and CD8 with either yellow or cyan is performed sequentially and fully automated on the VENTANA BenchMark ULTRA stainers and includes a heat deactivation (HD) step between the two rounds of detection to prevent cross-reactivity of same species antibodies. The assay was developed and tested using normal human tonsil tissue, lung carcinomas, and multi tissue arrays. The staining was assessed by trained pathologists or measured using commercially available imaging software.

**Results**

**Fig. 46:**
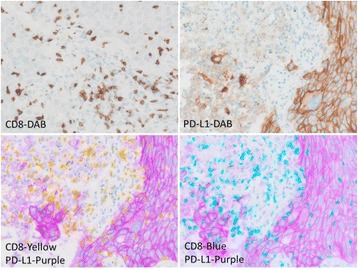
**(Abstract P104).** Comparative single and dual staining of PD-L1 and CD8 in lung tumor tissue

The PD-L1/CD8 duplex assay showed equivalent staining to the single PD-L1 or CD8 DAB staining (Fig. 46) on normal tonsil and lung carcinomas. The reproducibility test showed 100% agreement between three lots of reagents and 89% (blue detection) and 100% (yellow and purple detection) agreement between three testing laboratories. The staining precision is acceptable as the coefficient of variation of staining intensity was less than 2%. Effectiveness of heat deactivation was validated for the conditions of the duplex assay as we did not detect cross-reactivity or adverse effects on the epitopes and the chromogens.

**Conclusions**

We developed a fully automated and robust dual IHC staining assay for detection of PD-L1 and CD8 markers on the same slide. The chromogens used in this assay are compatible with regular counterstains and alcohol dehydration. The dual IHC assay could facilitate mining of spatial relationship between the two markers for evaluation of tumor microenvironment.

### P105 Application of a test developed for prediction of response to high dose interleukin-2 (HDIL-2) and the BDX008 test for prediction of outcomes following checkpoint inhibitors to cohorts of patients treated with HDIL-2 or nivolumab

#### Ryan J Sullivan^1^, Theodore Logan^2^, Nikhil Khushalani^3^, Kim Margolin^4^, Henry Koon^5^, Thomas Olencki^6^, Thomas Hutson^7^, Brendan Curti^8^, Joanna Roder^9^, Shauna Blackmon^10^, Heinrich Roder^9^, John Stewart^11^, Asim Amin^12^, Marc S Ernstoff^13^, Joseph I Clark^14^, Michael B Atkins^15^, Howard L Kaufman^16^, Jeffrey Sosman^17^, Jeffrey Weber^18^, David F McDermott^19^

##### ^1^Medical Oncology Department, Massachusetts General Hospital, Boston, MA, USA; ^2^Simon Cancer Center, Indiana University, Indianapolis, IN, USA; ^3^H. Lee Moffitt Cancer Center, Tampa, FL, USA; ^4^Department of Medical Oncology, City Of Hope, Duarte, CA, USA; ^5^Case Western Reserve University, Cleveland, OH, USA; ^6^The Ohio State University, Columbus, OH, USA; ^7^Texas Oncology-Baylor Charles A. Sammons Cancer Center, Dallas, TX, USA; ^8^Earle A. Chiles Research Institute, Providence Cancer Center, Portland, OR, USA; ^9^Biodesix, Inc., Boulder, CO, USA; ^10^Massachusetts General Hospital Cancer Center, Boston, MA, USA; ^11^Wake Forest Baptist Medical Center, Winston Salem, NC, USA; ^12^Levine Cancer Institute, Carolinas HealthCare System, Charlotte, NC, USA; ^13^Roswell Park Cancer Institute, Buffalo, NY, USA; ^14^Loyola University Medical Center, Maywood, IL, USA; ^15^Georgetown-Lombardi Comprehensive Cancer Center, Washington DC, DC, USA; ^16^Rutgers Cancer Institute of New Jersey, New Brunswick, NJ, USA; ^17^Robert Lurie Comprehensive Cancer Center of Northwestern University, Chicago, IL, USA; ^18^NYU Langone Medical Center, New York, NY, USA; ^19^Beth Israel Deaconess Medical Center, Boston, MA, USA

###### **Correspondence:** Joanna Roder (joanna.roder@biodesix.com)

**Background**

With multiple approved immunotherapies for metastatic melanoma (MM), tests that enable optimal treatment selection are needed. BDX008 is a serum protein-based test that identifies patients with better (BDX008+) or worse (BDX008-) survival (OS) on nivolumab [1]. Using similar methods and samples from the IL-2 Select trial, a test for prediction of progression-free survival (PFS) after HDIL-2 treatment has been developed that divides patients into two groups, A and B (better and worse PFS, respectively). Performance of these tests is compared in two cohorts of MM patients treated with either HDIL-2 or nivolumab.

**Methods**

**Fig. 47:**
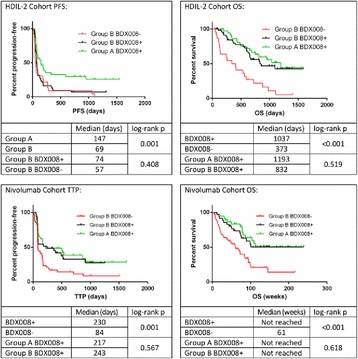
**(Abstract P105).**

MALDI mass spectra were generated for pre-treatment samples from 114 pts in the IL-2 Select trial and 119 patients from a trial of nivolumab with or without a peptide vaccine. (The IL-2 trial was used for development of the IL-2 test, the nivolumab/peptide trial for development of BDX008.) Both the IL-2 test and BDX008 were applied to all spectra generated from these trial samples. Outcomes included PFS (HDIL-2), time to progression (TTP; nivolumab/peptide), and OS (both), depending on the test and the trial agent (Fig. 47 and results).

**Results**

Of the 112 patients treated with HDIL-2 (2 failed BDX008 QC), 39 (35%) were classified as group A, and 89 (79%) as BDX008+. All group A samples were BDX008+. PFS was strongly correlated with IL-2 test classification: IL-2 test group B had inferior PFS compared with IL-2 test group A, but no difference whether BDX008+ or not. In contrast, OS was favorable with BDX008+ regardless of IL-2 test group. Hence, patients classified as IL-2 test group B and BDX008+ had a similar OS to group A patients despite an inferior PFS, possibly due to subsequent checkpoint inhibitor therapy or to pre-IL-2 factors with prognostic importance but not predictive for PFS after IL-2. In the nivolumab patients, 72 (61%) samples were BDX008+ and 37 (31%) fell into IL-2 test group A; all 37 were BDX008+. Both TTP and OS correlated closely with BDX008: BDX008+ had better outcomes than BDX008-; also, BDX008- outcomes were independent of IL-2 test status.

**Conclusions**

Patients classified into poor outcome categories by both BDX008 and the IL-2 test have poor outcomes on both immunotherapeutic regimens. For the remaining patients, the two tests perform differently, identifying partially overlapping groups of patients likely to have good outcomes with one or the other therapy.

**Trial Registration**

ClinicalTrials.gov identifier NCT01288963 and NCT01176461.

**References**

1. Weber J, *et al*: **Pre-treatment patient selection for nivolumab benefit based on serum mass spectra.** SITC2015.

### P106 A test identifying advanced melanoma patients with long survival outcomes on nivolumab shows potential for selection for benefit from combination checkpoint blockade

#### Jeffrey Weber^1^, Harriet Kluger^2^, Ruth Halaban^3^, Mario Snzol^2^, Heinrich Roder^4^, Joanna Roder^4^, Senait Asmellash^4^, Arni Steingrimsson^4^, Shauna Blackmon^5^, Ryan J Sullivan^6^

##### ^1^NYU Langone Medical Center, New York, NY, USA; ^2^Yale Medical Oncology, New Haven, CT, USA; ^3^Yale University School of Medicine, New Haven, CT, USA; ^4^Biodesix, Inc., Boulder, CO, USA; ^5^Massachusetts General Hospital Cancer Center, Boston, MA, USA; ^6^Medical Oncology Department, Massachusetts General Hospital, Boston, MA, USA

###### **Correspondence:** Joanna Roder (joanna.roder@biodesix.com)

**Background**

Multiple immunotherapeutic regimens for the treatment of metastatic melanoma (MM) are now approved and can provide long term benefit to a proportion of patients. Blood-based assays that can assist treatment selection are of significant clinical relevance. Using serum mass spectrometry, a test has been developed to predict good outcomes on anti-PD-1 therapy. We present results on the development and validation of this test.

**Methods**

MALDI mass spectra were generated for pretreatment serum samples from 119 patients treated with nivolumab at Moffitt Cancer Center. Using a classifier development platform optimized for creation of multivariate molecular diagnostic tests which can generalize well to independent datasets, a test was created to identify patients with particularly good outcomes. This test classifies samples into two groups: group 1 (very good outcome) and group 2 (inferior outcome). This test was validated in two independent cohorts of MM patients receiving anti-PD-1 agents: 30 patients from Yale University (YU) and 25 patients, most treated with pembrolizumab, from Massachusetts General Hospital (MGH). The test was also applied to pretreatment samples collected from 21 patients from YU receiving combination PD-1/CTLA-4 blockade. Difference in outcomes between patients classified as group 1 and group 2 within each cohort were assessed using log-rank p values and Cox proportional hazard ratios (HRs).

**Results**

Of the 119 patients used in test development, 34 (29%) were classified as group 1. Group 1 had better survival (OS) and time-to-progression (p=0.002 and p=0.014, respectively), with two-year survival of 67%. In the YU cohort receiving an anti-PD-1 agent, 13 (43%) classified as Group 1, which had better OS than Group 2 (p<0.001) and two-year survival above 80%. Eleven (44%) patients from the MGH cohort were classified as Group 1. There was a trend to improved OS for Group 1 compared to Group 2 (p=0.062, HR= 0.17). Within the cohort of patients receiving combination PD-1/CTLA-4 blockade, 13 (62%) classified as Group 1. Two-year survival was 83% in Group 1 and 63% in Group 2.

**Conclusions**

The test identifies a subgroup of patients with extremely good outcomes on anti-PD-1 therapy. High two-year survival in the Group 1 cohorts may indicate that the test has potential utility in identifying patients who derive significant benefit from anti-PD-1 monotherapy and might gain little benefit from the addition of an anti-CTLA-4 agent. As follow-up was only 2 years for OS, and the numbers are small, further validation is necessary.

**Trial Registration**

ClinicalTrials.gov identifier NCT01176461.

### P107 Reproducibility of automated and semi-automated seven-color immunofluorescence staining with tyramide signal amplification

#### Chichung Wang, Kristin Roman, Amanda Clement, Sean Downing, Clifford Hoyt

##### PerkinElmer, Hopkinton, MA, USA

###### **Correspondence:** Kristin Roman (kristin.roman@perkinelmer.com)

**Background**

Multiplexed immunohistochemistry has become increasingly important as cancer immunologists seek understanding of specific cell-to-cell interactions within tumor and its microenviroment. To support this need, we have developed staining approaches that leverage the properties of tyramide signal amplification (TSA) to increase signals above background and to provide photostable balanced signals. However, automation is needed before broad adoption occurs in research and eventually in clinical practice. We present two methods for automation, one fully automated on a Leica Bond Rx and the other semi-automated on a Biocare Intellipath. The fully automated approach uses sodium dodecyl sulfate (SDS) to denature endogenous myeloperoxidase (MPO) and eliminate cross reactivity among antibodies without affecting previously applied fluorescence labels. The semi-automated on the Intellipath automates all staining steps, except for antibody stripping which occurs off-line using microwave. In this presentation, we present detailed protocols and assess analytical performance.

**Methods**

Previously, we demonstrated reproducible immunofluorescence labeling with a manual protocol utilizing microwave exposure to remove antibodies between each marker (Methods 70 (2014) 46–58). In the fully automated approach using the Bond Rx, we replace the microwave step with a 7-minute exposure to 5% SDS at 50C. Tonsil and breast cancer sections were stained with a 6-plex, 7-color panel for PD-1, PD-L1, CD8, CD68, Foxp3, cytokeratin, plus DAPI counterstain. Serial sections were used to assess reproducibility, cross talk, interference, and signal-to-background ratio. Slides were scanned and analyzed using multispectral imaging (Vectra^TM^) to isolate fluorescence signals for accurate measurement.

**Results**

Balanced specific staining was achieved with both methods. Crosstalk was below the limit of detection. Signal-to-background was above 10:1 for each label, as measured by looking at signal strength on and off cells positive for the marker of interest. Coefficient of variation of measured signals was generally less than 20% for the semi-automated approach and less than 15% for the fully automated approach.

**Conclusions**

This is the first demonstration of reproducible and independent fully-automated TSA-based automated multiplexed immunofluorescence using SDS to perform antibody denaturation. It is also a demonstration of a semi-automated approach that performs antibody stripping off of the autostaining instrument and is compatible with a broad range of mid-range and conventional autostaining platforms.

**References**

1. Stack EC, Wang C, Roman KA, Hoyt CC: **Multiplexed immunohistochemistry, imaging, and quantitation: A review, with an assessment of Tyramide signal amplification, multispectral imaging and multiplex analysis.** *Methods* 2014 , **70**:46-58.

### P108 Spatial distribution of CD8+ T cells predicts response to ipilimumab in malignant melanoma

#### Nathalie Harder^1^, Guenter Schmidt^1^, Ralf Schoenmeyer^1^, Nicolas Brieu^1^, Mehmet Yigitsoy^1^, Gabriele Madonna^2^, Gerardo Botti^2^, Antonio Grimaldi^3^, Paolo A Ascierto^3^, Ralf Huss^1^

##### ^1^Definiens AG, Munich, Bayern, Germany; ^2^Istituto Nazionale dei Tumori di Napoli Fondazione “G. Pascale”, Naples, Campania, Italy; ^3^Istituto Nazionale Tumori Fondazione, Napoli, Italy

###### **Correspondence:** Guenter Schmidt (gschmidt@definiens.com)

**Background**

Although checkpoint blockade immunotherapies are successful in some cancer patients, the majority of patients still do not benefit from these therapies. The advances of digital pathology and the availability of quantitative whole slide image analysis allows for a systematic search for novel tissue-based biomarkers to predict therapy response and overall survival. Here, we present a Tissue Phenomics case study to discover a potential companion diagnostic test for ipilimumab (IPI) in malignant melanoma.

**Methods**

The patient cohort is a subset of the MISIPI study [1], comprising 30 melanoma patients. Consecutive sections from FFPE tissue were immunohistochemically stained with CD3, CD8, and FoxP3. The sections were digitized and automatically aligned using an advanced staining-independent image registration algorithm. Tumor regions were manually annotated by a pathologist, excluding artifacts and necrotic regions. A novel parameter-free cell segmentation approach was used to automatically detect and classify cells with respect to their protein expression profile [2] and to the spatial distance to the annotated tumor border. We computed for each of the CD3+, CD8+ and FoxP3+ cell populations the average cell density in the tumor in a border region within the tumor and in a border region just outside the tumor. By computing a potentially predictive score for each ratio of the measured cell densities, we identified the most promising patient stratification algorithm in terms of predictive values for therapy response and the Kaplan-Meier statistics p-value for overall survival.

**Results**

We identified a promising scoring algorithm which is based on the ratio of the CD8+ cell density at the inner tumor border to the CD8+ cell density in the tumor. It enables patient stratification into IPI responders and non-responders with high predictive values (positive predictive value 68%, negative predictive value 79%, prevalence 39%) and at the same time, excellent prediction of overall survival (p-value < 0.0015).

**Conclusions**

A novel companion diagnostic algorithm for ipilimumab in malignant melanoma was discovered by Tissue Phenomics. It provides high predictive power and enables considerably improved treatment decisions. However, the results are still preliminary and need to be further validated.

**References**

1. Bifulco C, Capone M, Feng Z, *et al*: **MISIPI study: Melanoma ImmunoScore evaluation in patients treated with ipilimumab**. *J Transl Med* 2014, **12(Suppl 1)**:P11.

2. Brieu A, Pauly O, Zimmermann J, Binnig G, Schmidt G: **Slide-specific models for segmentation of differently stained digital histopathology whole slide images**. *Proc SPIE* 2016, **9784**:978410.

### P109 Tumor-associated macrophages (TAMs) as a prognostic marker for prostate cancer progression

#### Maria Athelogou^1^, Harald Hessel^2^, Nathalie Harder^1^, Alexander Buchner^3^, Guenter Schmidt^1^, Christian Stief^3^, Ralf Huss^1^, Gerd Binnig^1^, Thomas Kirchner^2^

##### ^1^Definiens AG, Munich, Bayern, Germany; ^2^Institute of Pathology, Ludwig-Maximilians-University, Munich, Germany; ^3^Department of Urology, Ludwig-Maximilians-University, Munich, Klinikum Grosshadern, Germany

###### **Correspondence:** Guenter Schmidt (gschmidt@definiens.com)

**Background**

Tumor-associated macrophages (TAMs) and tumor infiltrating T-cells (TILs) have been associated with tumor progression in various tumor entities. In this study, we applied the Tissue Phenomics technology to discover new TAM-related prognostic factors to predict prostate cancer progression. This technology correlates clinical outcome data with image analysis results from (virtually) multiplexed tissue slides. Multiplexing enables the co-analysis of multiple immunohistochemically (IHC) stained tissue sections. Using this method, we investigated the prognostic relevance of M1/M2 TAMs (CD68/CD163) and TILs (CD3/CD8) within prostate cancer (PCa) resection specimens from low-risk and intermediate-risk patients with respect to Prostate Specific Antigen (PSA) recurrence after radical prostatectomy (RP).

**Methods**

Analysis and quantification were performed on three consecutive duplex stained FFPE tissue sections from 89 patients (on which 40 with PSA recurrence) with low- to intermediate-risk PCa after RP with a Gleason-Score ≤ 7a. M1- and M2-type macrophages were characterized by CD68 and CD163, T-cells by CD3 and CD8, tumor and non-tumor epithelial tissue regions by CK18 and p63. CK18 positive glands without any p63-stained basal cell nuclei are considered as tumor glands. The three duplex stained slides were analyzed and converted into one virtual tissue slide through image co-registration. TAMs and TILs were quantified separately in the distinct tumor regions and the tumor microenvironment. Both, the overall number of TILs and TAMs and the ratio of M1- and M2-positive macrophages were analyzed using the Tissue Phenomics technology. The correlation between various TIL and TAM cell densities within the different regions of interest (tumor and tumor microenvironment), the tumor grading and tumor stage, and clinical parameters (PSA recurrence, overall and disease-free survival) were performed.

**Results**

Prostate cancer patients without PSA recurrence show a significantly higher ratio of CD8 to CD163 positive cell densities in the tumor microenvironment. The PSA recurrence prediction accuracy is 76.8% with a Kaplan-Meier log-rank test p-value of 2.7e-5 for the disease free survival time.

**Conclusions**

These first results indicate a considerable prognostic potential of TAMs to predict PSA recurrence in PCa. The application shows that the Tissue Phenomics Technology enables the investigation and the evaluation of the prognostic relevance of certain immune cell populations. The analysis of the relation of TILs to TAMs is an excellent example for such kind of applications with a potentially high impact for prostate cancer patient treatment decisions.

### P110 Co-expression of PD-L1 and other targetable protein and genomic markers: opportunities for combination therapy

#### Shankar Sellappan^1^, Sheeno Thyparambil^1^, Sarit Schwartz^1^, Fabiola Cecchi^1^, Andrew Nguyen^2^, Charles Vaske^2^, Todd Hembrough^1^

##### ^1^NantOmics, Rockville, MD, USA; ^2^NantOmics, Culver City, CA, USA

###### **Correspondence:** Shankar Sellappan (shankar.sellappan@nantomics.com)

**Background**

Several immune checkpoint inhibitors have been approved to treat multiple solid tumor types. Immune responses could be modulated by targeted therapies as well as cytotoxic agents. The opportunity to combine immunotherapies with agents against targetable proteomic and genomic biomarkers may lead to improved outcomes and reduced toxicities; however, analysis of multiple biomarkers creates an increased demand for tissue. Using a multi-omic approach that incorporates mass spectrometry based proteomic analysis and NGS, we were able to objectively detect the expression of multiple therapeutically-relevant proteins and genomic alterations from a minimum two FFPE sections. Here, we report on patient samples that express the PD-L1 protein and either co-express therapeutically-relevant proteins (EGFR, HER2, MET, ROS1, TOPO1, etc.) or gene mutations (MET, KIT, BRAF, JAK3, etc.).

**Methods**

Formalin-fixed, paraffin-embedded (FFPE) sections of tumor tissue from patients with different cancer indications were obtained. For proteomics analysis, tumor area of 8 mm^2^ was marked by a board-certified pathologist. Following laser microdissection of the marked areas, tumor cell proteins were extracted using the Liquid Tissue® process and subjected to selected reaction monitoring mass spectrometry to quantify 27 targeted proteins in each patient sample. For genomics analysis, genomic content was isolated from the FFPE tumor tissue sections as well as from corresponding normal samples and subjected to NGS for whole genome sequencing (tumor and normal) and whole transcriptome sequencing (tumor only) analysis.

**Results**

Quantitative proteomic analyses of 1710 cancer patient samples across multiple indications revealed a wide range (144 – 1025 amol/mg) of PD-L1 protein expression. Several actionable protein targets were co-expressed with PD-L1, including targeted therapy markers (EGFR (80%), HER2 (29%), MET (56%), ROS1 (22%)) and chemotherapy markers (TOPO1 (100%), etc.). PD-L1 protein expressing tumors were also found to harbor therapeutically-relevant genomic mutations, including MET (N375S and T1010I), JAK3 (V722I), KIT (M541L), BRAF (V600E), CDKN2A (D84H), etc.

**Conclusions**

Tumor molecular profiling by both proteomic and genomic analysis revealed co-expression of targetable proteins (EGFR, HER2, MET, TOPO1, etc.) and genomic alterations (MET, KIT, BRAF, etc.) in patients that express the PD-L1 protein. From minimal amounts of tissue, we were able to assess multiple therapeutically-associated biomarkers using mass spectrometry based targeted quantitative proteomics and NGS based comprehensive genomic (DNA and RNA) analysis. Ongoing clinical trials involving immunotherapy and chemo/targeted therapies could benefit from molecularly stratifying patients by proteomic and genomic testing to increase efficacy and reduce toxicity.

### P111 Immunomonitoring of patients with colorectal cancer

#### Jan Spacek^1^, Michal Vocka^1^, Eva Zavadova^1^, Helena Skalova^1^, Pavel Dundr^1^, Lubos Petruzelka^1^, Nicole Francis^1^, Rau T Tilman^2^, Arndt Hartmann^3^, Irena Netikova^1^

##### ^1^General Hospital First Medical Faculty, Prague, Hlavni mesto Praha, Czech Republic; ^2^Institut für Pathologie, Universität Bern, Bern, Switzerland; ^3^Pathologie, Friedrich-Alexander-Universität Erlangen-Nürnberg, Erlangen, Germany

###### **Correspondence:** Jan Spacek (jan.spacek@vfn.cz)

**Background**

Colorectal cancer is the second leading cause of cancer death worldwide. This high mortality is because almost half of colorectal cancers are detected at an advanced stage of disease, and the currently used prognostic factors are not always accurate. Recently, the prognostic impact of the body’s immune response to cancer has been demonstrated. Immunoscore (*in situ* immune cell infiltrate in tumours) has been shown to be a very powerful prognositc indicator in patients with clinically localized colorectal cancer. These patients are usually treated with only surgical removal of the tumour; however, approximately 25% of these patients will have recurrence of their disease, indicating that occult metastases were already present at the time of curative surgery. No tumour-associated marker predicts the recurrence of this subgroup of patients that could benefit from adjuvant therapy. These days are investigated other parameters of the immune response – mostly cellular immunity and the production of immunosuppressive and neoangiogenic markers. VEGF is the factor responsible for neoangiogenesis and it is being considered as a possible prognostic marker of disease progression. Transforming growth factor-beta (TGF-beta) is also neoangiogenic and a highly immunosuppressive factor, as it suppresses the body’s natural immunity against tumours. TGF-beta is also being considered as another possible prognostic marker of disease progression. The purpose of this study was to monitor the immune response in patients with stage II colorectal cancer, with a focus on cellular as well as humoral immunity. TGF-beta and VEGF levels were followed.

**Methods**

22 patients with stage II colon cancer included in the research project received routine cancer teratment. Basic parameters – histological type and grade, proliferative markers – were established at baseline. Patients were evaluated by a clinical immunooncolgist to exclude any immune disorders of allergic or autoimmune origin. TGF-beta and VEGF were measured using ELISA, and anti-tumour cellular immunity (CD4, CD8, T-reg, B cells) were measured via flow cytometry.

**Results**

In patients with stage II colorectal cancer, predominantly a depression in cellular immunity was seen. Plasma levels of immunglobulins were also reduced, particularly the IgG4 subtype. Most patients showed some clinical symptoms of immunodeficiency, such as frequent respiratory tract infections and/or herpetic infections. TGF-beta and VEGF plasma levels were increased.

**Conclusions**

The correlation of these neoangiogenic and immunosuppressive factors, as well as the state of anticancer immunity, could help in the future as a prognostic marker and contribute to the selection of targeted immune therapy in patients with colorectal cancer.

### P112 Evaluation of immune cell infiltrates in non-small cell lung cancer as a potential comprehensive prognosticator

#### Carmen Ballesteros-Merino^1^, Julia Stump^2^, Amanda Tufman^2^, Frank Berger^3^, Michael Neuberger^4^, Rudolf Hatz^5^, Michael Lindner^6^, Rachel E Sanborn^7^, John Handy^7^, Bernard Fox^7^, Carlo Bifulco^7^, Rudolf M Huber^2^, Hauke Winter^4^, Simone Reu^8^

##### ^1^Earle A. Chiles Research Institute at Portland Providence Cancer Center, Portland, OR, USA; ^2^Ludwig Maximilian University of Munich and Thoracic Oncology Centre Munich, Germany; Comprehensive Pneumology Center (CPC) and Member of the German Center for Lung Research (DZL, CPC-M), Munich, Bayern, Germany; ^3^Department of Clinical Radiology, University of Munich, Munich, Bayern, Germany; ^4^Comprehensive Pneumology Center (CPC) and Member of the German Center for Lung Research (DZL, CPC-M); Department of General, Visceral, Transplantation, Vascular and Thoracic Surgery, Hospital of the Ludwig Maximilian University, Munich, Bayern, Germany; ^5^Comprehensive Pneumology Center (CPC) and Member of the German Center for Lung Research (DZL, CPC-M), Munich; Hospital of the Ludwig Maximilian University, Munich; Asklepios Clinic, Munich, Bayern, Germany; ^6^Comprehensive Pneumology Center (CPC) and Member of the German Center for Lung Research (DZL, CPC-M), Munich, Germany; Asklepios Clinic Munich-Gauting, Germany, Munich-Gauting, Bayern, Germany; ^7^Robert W. Franz Cancer Research Center, Earle A. Chiles Research Institute, Providence Cancer Center, Portland, Oregon, USA; ^8^Comprehensive Pneumology Center (CPC) and Member of the German Center for Lung Research (DZL, CPC-M); Institute of Pathology, University of Munich, Munich, Bayern, Germany

###### **Correspondence:** Julia Stump (julia.stump@med.uni-muenchen.de)

**Background**

Therapeutic strategies in non-small cell lung cancer (NSCLC) are based on the histopathological evaluation of the tumor tissue which, in conjunction with the TNM staging system, remains the gold standard for prognostic assessment. Nevertheless, histopathology and stage-specific clinical outcomes vary significantly, indicating that there is a need for additional prognosticators. A previous study reported that intense lymphocytic infiltrates were found in 6-11% of NSCLC and were associated with a significant increase in disease-free and overall survival [1]. The aim of our study was to use multispectral imaging and digital quantification to assess relationships between T cells and FoxP3+ or PD-L1+ cells in tissue microarrays (TMAs) cored from “hot spots” of dense infiltrates at the invasive margin and center of the NSCLC.

**Methods**

Tissue microarrays (TMA) were generated from formalin-fixed paraffin-embedded tissue of 98 curatively resected patients with stage IA-IIIA NSCLC. TMAs included 3 cores for tumor center and 3 cores for invasive margin selected from the areas with the most dense (hot spot) lymphocytic infiltrate. TMAs were immunolabeled with mIHC technique for the following antibodies: PD-L1, CD8, CD3, FoxP3, CD163, cytokeratin and DAPI and quantified with InForm software. Results were compared between groups of patients with squamous and non-squamous cell carcinoma and correlated with the course of the disease, overall/progression-free survival, metastases or relapse and clinicopathological parameters.

**Results**

In contrast to reports evaluating whole sections, analysis of “hot spots” in this cohort of patients failed to provide a prognostic biomarker for survival. Current studies are evaluating how an evaluation of the whole section correlates with results of this “hot-spot” analysis. Our preliminary data suggests that the “hot-spot” analysis of CD8, FoxP3 and PD-L1 does not allow us to identify outcome in NSCLC.

**Conclusions**

Multispectral assessment of CD8, FoxP3, and PD-L1 performed on “hot-spots” of stage I-III NSCLC did not provide a prognostic biomarker. Given other reports that immune infiltrates are associated with improved outcome, this suggests it may be important to evaluate a larger percentage of the tumor. These findings appear to contrast with the colon immunoscore project which identified a correlation with an enumeration of CD3+ and CD8+ T cell numbers in hot-spots and disease-free and overall survival.

**Acknowledgements**

C. Ballesteros-Merino and J. Stump contributed equally to this study. Supported by the Harder Family, Lynn and Jack Loacker, Robert W. Franz, Wes and Nancy Lematta, the Murdock Trust and Providence Medical Foundation.

**References**

1. Brambilla E, Le Teuff G, Marguet S, Lantuejoul S, Dunant A, Graziano S, *et al*: **Prognostic Effect of Tumor Lymphocytic Infiltration in Resectable Non-Small-Cell Lung Cancer.** *J Clin Oncol* 2016, **34**:1223-1230.

### P113 High NKG2A expression contributes to NK cell exhaustion and predicts a poor prognosis of patients with liver cancer

#### Cheng Sun, Weihua Xiao, Zhigang Tian

##### Institute of Immunology, The Key Laboratory of Innate Immunity and Chronic Disease (Chinese Academy of Medical Science), School of Life Sciences and Medical Center, University of Science & Technology of China, Hefei, Anhui, People’s Republic of China

###### **Correspondence:** Cheng Sun (charless@ustc.edu.cn)

**Background**

As the predominant lymphocyte subset in the liver, nature killer (NK) cells have been shown to be highly associated with the outcomes of patients with chronic hepatitis B virus infection (CHB) and hepatocellular carcinoma (HCC). Previously, we reported that NKG2A, a checkpoint candidate, mediates human and murine NK cell dysfunction in CHB. However, NK cell exhaustion and, particularly, the level of NKG2A expression within liver tumours have not been reported.

**Methods**

In this study, we analysed NKG2A expression and the related dysfunction of NK cells located in intra- or peritumour regions of liver tissue samples from 207 HCC patients, in addition to analysing disease outcomes.

**Results**

The expression of NKG2A in NK cells and the NKG2A ligand, HLA-E, in intratumour HCC tissues was observed to be increased. These NK cells, and particularly CD56^dim^ NK cells, with higher NKG2A expression showed features of functional exhaustion and were associated with a poor prognosis. The increase in NKG2A expression might be induced by IL-10, which was present at a high level in the plasma of HCC patients. Blocking IL-10 could specifically inhibit NKG2A expression in NK cells.

**Conclusions**

These findings indicate that NKG2A expression is influenced by factors from cancer nests and contributes to NK cell exhaustion, suggesting that NKG2A blockade has the potential to restore immunity against liver tumours by reversing NK cell exhaustion.

**Fig. 48:**
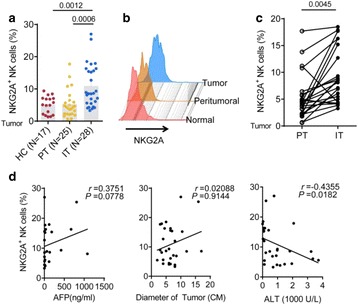
**(Abstract P113).** The frequency of intratumour NKG2A+ NK cells is increased in HCC. (A, B) The percentage of NKG2A-expressing NK cells in healthy livers (N=17) and IT (N=28) and PT (N=25) from HCC patients. The differences in the cumulative data were calculated using a two-tailed paired Student’s t test. (C) NKG2A expression in NK cells from the paired central tumour and PT from each HCC patient (N=23). (D) The correlation between NKG2A expression in hepatic NK cells from IT and the serum AFP and ALT levels and tumour diameters of HCC patients. Spearman’s correlation coefficients are shown.

**Fig. 49:**
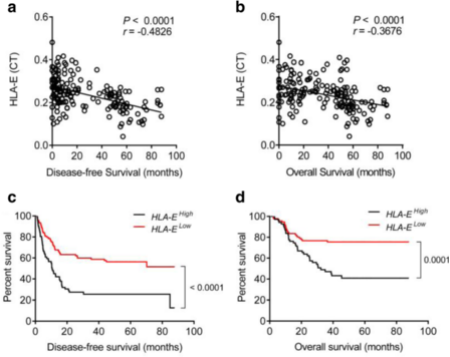
**(Abstract P113).** Shorter survival of patients with higher densities of HLA-E+ cells within tumours. (A, B) Correlations between DFS or OS and the density of HLA-E+ cells in the IT regions of HCC patients (N=177). Pearson’s correlation coefficients (R) and P values are shown. (C, D) Kaplan–Meier survival curves for the duration of DFS (C) and OS (D) in months, according to the HLA-E+ cells density in IT (low densities, Red line; high densities, Black line) (log-rank test)

### P114 Association of non-coding repeat RNA expression with immune infiltrates

#### Kshitij Arora^1^, Niyati Desai^1^, Anupriya Kulkarni^1^, Mihir Rajurkar^1^, Miguel Rivera^1^, Vikram Deshpande^2^, David Ting^1^

##### ^1^Massachusetts General Hospital Cancer Center, Harvard Medical School, Charlestown, MA, USA; ^2^Massachusetts General Hospital, Harvard Medical School, Boston, MA, USA

###### **Correspondence:** David Ting (dting1@mgh.harvard.edu)

**Background**

Colorectal cancer (CRC) is the third most common type of cancer in the United States. There have been exciting responses to immunotherapy in CRC, but the ability to predict which patients would benefit from these drugs remains elusive. Recent work has demonstrated the aberrant expression of non-coding repeat RNAs across cancers including colon cancer [1, 2]. These repeats appear to trigger elements of the innate immune response that may have implications for response to immunotherapy [1]. To determine if repeat RNAs are linked to the tumor immune microenvironment, we developed a novel combined repeat RNA *in situ* hybridization (RNA-ISH) with T cell immunohistochemistry (IHC) on fixed formalin paraffin embedded (FFPE) tissues. We assessed two different repeat types (HSATII and HERV-H) that have been well documented in human cancers and reported to have an association with innate immune responses [2]. To investigate correlation of host immune T cells with these repeats a pilot study of seventy-five patients of CRC was performed.

**Methods**

FFPE tissue sections of 75 patients were stained by RNA-ISH and IHC on the Leica Bond RX. HSATII and HERV-H repeat RNA-ISH and CD8 (cytotoxic) and FOXP3 (regulatory) T cell marker IHC was performed on each case. Repeat RNA scores were determined as HIGH or LOW based on relative cancer cell expression to normal adjacent tissues. T cell density was determined by counting the number of positive cells in a 400 x 200 μm area. T cell density differences were calculated by t-test.

**Results**

We found HSATII was HIGH in 47 (63%) and HERV-H was HIGH in 36 (48%) of CRC cases with a concordance between repeats of 51%. Analysis with T cell subsets revealed lower CD8+ T cells in HSATII HIGH tumors (306 vs 686 CD8+ T cells/mm^2^; p2; p=0.03).

**Conclusions**

Repeat RNAs are expressed across malignancies with varying levels that correlate with different immune infiltrates. Cancer expression of HSATII and HERV-H repeats offers a novel surrogate biomarker of cytotoxic and regulatory T cell tumor infiltration. The mechanistic effects of these repeats on immune cell function merits further investigation.

**Acknowledgements**

We thank Alex Forrest-Hay, Manoj Gandhi, Frank Witney, and Affymetrix, Inc. for sponsored research support.

**References**

1. Chiappinelli KB, Strissel PL, Desrichard A, Li H, Akman B, *et al*: **Inhibiting DNA Methylation Causes an Interferon Response in Cancer via dsRNA Including Endogenous Retroviruses.** *Cell* 2015, **162**:974-986.

2. Ting DT, Lipson D, Brannigan BW, Akhavanfard S, Coffman EJ, *et al*: **Aberrant overexpression of satellite repeats in pancreatic and other epithelial cancers.** *Science* 2011, **331**:593-596.

**Fig. 50:**
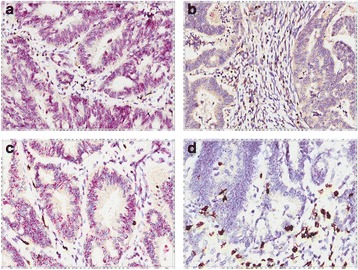
**(Abstract P114).** Colon adenocarcinoma FFPE tissue stained with dual color RNA-ISH (Red) for HERVH, HSATII and IHC (Brown) for FOXP3 & CD8. 400X magnification **a** High HervH and Low FOXP3 **b** Low HERVH and High FOXP3 **c** High HSATII and Low CD8 **d** Low HSATII and High CD8

### P115 Outcome disparities by sex in melanoma patients treated with anti-PD-1 therapy

#### Katy Tsai^1^, Adi Nosrati^1^, Simone Goldinger^2^, Omid Hamid^3^, Alain Algazi^1^, Paul Tumeh^4^, Jimmy Hwang^1^, Jacqueline Liu^1^, Lawrence Chen^1^, Reinhard Dummer^2^, Michael Rosenblum^1^, Adil Daud^1^

##### ^1^University of California, San Francisco, San Francisco, CA, USA; ^2^University Hospital of Zurich,, Zurich, Switzerland; ^3^The Angeles Clinic & Research Institute, Los Angeles, CA, USA; ^4^University of California, Los Angeles, Los Angeles, CA, USA

###### **Correspondence:** Katy Tsai (katy.tsai@ucsf.edu)

**Background**

PD-1 antibodies have shown significant clinical activity against advanced melanoma, but ORR with PD-1 monotherapy remains less than 50%. We previously reported and validated a clinical scoring model to predict response to anti-PD-1, and in our logistic regression analysis, female sex was found to be associated with lower response rate (OR 0.36, 95% CI 0.19-0.67). We report further on outcomes in immunotherapy-treated patients by sex and correlate this with results of T cell profiling of an exhausted, antigen-experienced phenotype (% PD-1^high^CTLA-4^high^ CD8 cells) from pre-treatment biopsy samples.

**Methods**

336 patients (118 women, 218 men) with advanced cutaneous melanoma were treated with pembrolizumab or nivolumab at 4 academic cancer centers between December 2011 and October 2013. Baseline demographics and clinical characteristics were collected from electronic health records. Tumor response was assessed using RECIST v1.1 criteria. Pre-treatment flow cytometry data from 122 patients (45 women, 77 men) treated with both PD-1 monotherapy and PD-1/CTLA-4 combination therapy were analyzed by sex for correlative data.

**Results**

**Fig. 51:**
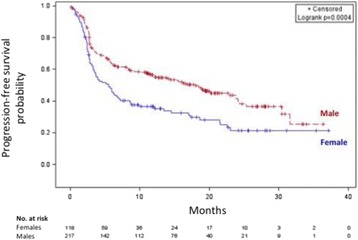
**(Abstract P115).** Progression-free survival estimates by sex in advanced melanoma patients receiving anti-PD-1 monotherapy

**Fig. 52:**
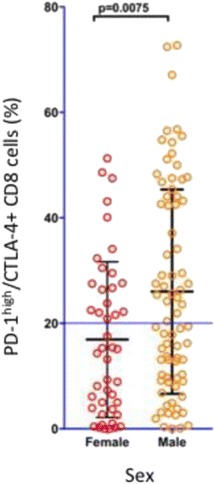
**(Abstract P115).** Pre-treatment of PD-1high/CTLA-4high CD8 T cells by sex

In the PD-1 monotherapy cohort, median ORR was 33.1% (95% CI 24.4-41.2) in females and 54.6% (95% CI 47.9-61.3) in males (p=0.0001). Median PFS was 5.5 months (95% CI 3.4-7.1) in females and 18 months (95% CI 11.3-23.8) in males (p=0.0004) (Fig. 51). Flow cytometry analysis of pre-treatment tumor biopsies revealed a statistically significant reduced proportion of exhausted PD-1^high^/CTLA-4^high^ CD8 T cells that persisted across age groups (Fig. 52).

**Conclusions**

There is a significant discrepancy between response to PD-1 monotherapy between men and women with advanced melanoma. The mechanisms of this may have immunologic basis given the difference in pre-treatment T cell profiles between men and women.

**Trial Registration**

ClinicalTrials.gov identifier NCT01295827, NCT01704287, NCT01721746, and NCT02156804.

### P116 A flexible and versatile toolbox for parallel multiplex immunohistochemical detection using recombinant epitope-tagged antibodies and monoclonal anti-tag antibodies

#### Tsu-Shuen Tsao^1^, Julia Ashworth-Sharpe^1^, Donald Johnson^1^, Srabani Bhaumik^1^, Christopher Bieniarz^1^, Joseph Couto^2^, Michael Farrell^1^, Mahsa Ghaffari^1^, Iva Habensus^1^, Antony Hubbard^1^, Tobin Jones^1^, Brian Kelly^1^, Jerome Kosmeder^1^, Cleo Lee^2^, Erin Marner^1^, Jeffrey Meridew^1^, Nathan Polaske^1^, Adriana Racolta^1^, Diana Uribe^1^, Hongjun Zhang^1^, Jian Zhang^1^, Wenjun Zhang^1^, Yifei Zhu^2^, Larry Morrison^1^, Lidija Pestic-Dragovich^1^, Lei Tang^1^

##### ^1^Ventana Medical Systems, Inc., Tucson, AZ, USA; ^2^Spring Bioscience, Pleasanton, CA, USA

###### **Correspondence:** Tsu-Shuen Tsao (tsu-shuen.tsao@roche.com)

**Background**

Contextual detection of multiple biomarkers on single formalin-fixed, paraffin-embedded (FFPE) slides for clinical applications remains an unmet need. Current multiplex immunohistochemistry (IHC) procedures entail successive rounds of antibody application and fluorophore attachment followed by antibody inactivation. We developed a parallel multiplex IHC approach using series of epitope-tagged antibodies and anti-epitope antibodies conjugated to fluorophores, haptens, or enzymes, and demonstrated feasibility by 5-plex fluorescent and duplex brightfield assays of markers relevant for immuno-oncology.

**Methods**

DNA sequences corresponding to peptide epitope tags were fused in-frame to rabbit monoclonal antibody cDNAs for expression in mammalian cells. Recombinant tagging bypasses the potential antibody inactivation associated with chemical-based tagging. Conjugation of fluorophores or haptens to antibodies was performed using NHS ester precursors. Horseradish peroxidase (HRP) and alkaline phosphatase (AP) were conjugated to reduced antibodies via NHS-maleimide linkers. Affinity of anti-epitope antibodies to peptides was assessed using biolayer interferometry. IHC of FFPE tissue sections was performed on VENTANA BenchMark ULTRA platform.

**Results**

Rabbit monoclonal antibodies against CD3, CD8, CD68, FoxP3, and PD-L1 were each engineered with a unique epitope tag. Epitope-tagged primary antibodies produced identical diaminobenzidine (DAB) staining intensity and pattern as untagged native antibodies. Multiple clones of recombinant rabbit monoclonal antibodies against each of the five epitope tags were conjugated and screened for retention of affinity, stability, and appropriate staining intensity and pattern in validated tissue microarrays. At least one clone of each anti-epitope antibody met the functional requirements and these were used to stain FFPE lung and tonsil tissue sections in conjunction with cocktailed epitope-tagged antibodies. Epitope-tagged antibodies were detected using one of three detection configurations in order of sensitivity: 1) fluor-conjugated anti-epitope antibodies, 2) hapten-conjugated anti-tag antibodies and fluor-conjugated anti-hapten antibodies, and 3) attachment of tyramide- or quinone methide-fluors to tissue specimens with HRP- or AP-conjugated anti-epitope antibodies. Titration of antibodies and assay optimization enabled pairings of particular biomarkers with detection configurations to generate specific fluorescence patterns and relative intensities similar to those produced by DAB stains using untagged antibodies and HRP-conjugated anti-species antibodies. Two-color brightfield stains were generated using enzyme-conjugated anti-tag antibodies and HRP- and AP-activated chromogens.

**Conclusions**

We have demonstrated feasibility of parallel multiplex IHC using a series of epitope-tagged antibodies and fluor-, hapten-, or enzyme-conjugated anti-epitope antibodies. Applying tagged antibodies and anti-tag antibody probes as cocktails streamlines multiplex detection and avoids the inactivation of antibodies that is necessary in multiplex approaches based on serially applied antibodies.

### P117 Multiplex immunohistochemistry and image cytometry analysis for phenotyping spatial immune characteristics

#### Takahiro Tsujikawa^1^, Rohan N Borkar^2^, Vahid Azimi^2^, Sushil Kumar^1^, Guillaume Thibault^1^, Motomi Mori^1^, Edward El Rassi^1^, Daniel R Clayburgh^1^, Molly F Kulesz-Martin^1^, Paul W Flint^1^, Lisa M Coussens^1^

##### ^1^Oregon Health & Science University, Portland, OR, USA; ^2^Intel Corporation, Hillsboro, OR, USA

###### **Correspondence:** Lisa M Coussens (coussenl@ohsu.edu)

**Background**

While immune therapies targeting select checkpoint molecules have revolutionized cancer medicine, the fact that many patients fail to respond underscores the need for improved patient stratification and application of multi-nodal biomarkers reflecting *in situ* status of the tumor immune microenvironment.

**Methods**

We developed a multiplexed immunohistochemical (IHC) imaging approach based on sequential IHC with iterative labeling, digital scanning and subsequent stripping of sections. To elucidate contextual information on leukocyte presence, complexity and functional phenotype *in situ*, we investigated these parameters in head and neck squamous cell carcinoma (HNSCC) utilizing multiplex IHC analyses in de-identified tissue microarray sections reflecting human papilloma virus (HPV)-positive and negative oropharyngeal HNSCC and normal pharynx. Quantitative multiparameter image cytometry was developed to enable quantification of cell density, composition and location of 16-immune cell lineages, along with monitoring of programed cell death-1 (PD-1) receptor and ligand (PD-L1) expression.

**Results**

Intratumoral immune cell densities were comprehensively evaluated by multiplex IHC/image cytometry via unsupervised clustering analysis and revealed presence of lymphoid, myeloid, and hypo-inflamed profiles correlating with HPV-status. Myeloid-inflamed profiles with low ratios of CD163^–^/CD163^+^ of CD68^+^ CSF1R^+^ macrophages and CD8^+^ T cell/CD68^+^ ratios was significantly associated with poor prognosis in both HPV-positive and negative HNSCC. Neoplastic cell nest vs. stroma region analyses revealed distinct distribution patterns for T_H_1 and T_H_2 lymphocytes, showing intratumoral T_H_1 orientation in HNSCC. Intratumoral CD66b^+^ granulocyte infiltration was associated with unfavorable prognosis. Further analysis for spatial characteristics of immune complexity revealed high T_H_1/T_H_2 ratios and CD8^+^ T cell infiltration within a distance of 20 μm to PD-L1^+^ cells, indicating an association between PD-L1 expression and regional characteristics of immune complexity.

**Conclusions**

These results demonstrate the capability of multiplex IHC-based image cytometry analysis toward improved understanding of heterogeneous tumor microenvironments.

**Acknowledgements**

This project was supported by Oregon Clinical and Translational Research Institute (OCTRI), grant number (UL1TR000128) from the National Center for Advancing Translational Sciences (NCATS) at the National Institutes of Health (NIH), and P30 CA069533-17 OHSU Knight Cancer Institute. LMC acknowledges support from the NIH/NCI, DOD BCRP Era of Hope Scholar Expansion Award, Susan G. Komen Foundation, Stand Up To Cancer – Lustgarten Foundation Pancreatic Cancer Convergence Dream Team Translational Research Grant, Breast Cancer Research Foundation, and the Brenden-Colson Center for Pancreatic Health.

### P118 Analysis of immunerelated prognostic markers in colon cancer patients randomized to surgery alone or surgery and adjuvant cytostatic treatment

#### Lisa Villabona, Giuseppe V Masucci

##### Karolinska Institutet, Stockholm, Stockholms Lan, Sweden

###### **Correspondence:** Lisa Villabona (lisa.villabona@ki.se)

**Background**

We have previously shown that human leukocyte antigen (HLA)-A*02, a common allele in the Scandinavian population, is a negative prognostic factor in epithelial ovarian cancer [1]. It is a strong predictor of patient outcome, only inferior to clinical staging. We have also shown that this prognostic trait in epithelial ovarian cancer is stronger by the presence of the gene compared with the expression of its protein, MHC class I [2]. Finally, we have shown that HLA-G expression in ovarian cancer is a negative prognostic marker [3]. Our aim was to analyse the prognostic markers HLA-A*02 genotype, MHC class I and HLA-G expression on tumour cells and the CD8+ lymphocyte infiltration in colon cancer.

**Methods**

Clinical information and primary tumours were collected from 520 colon cancer patients and followed for overall survival for 120 months. HLA-A*02 genotype was determined by conventional PCR. MHC class I expression and CD8+ lymphocyte infiltration were determined by immunohistochemistry.

**Results**

Patients with a stage III tumour and HLA-A*02 genotype had a better outcome if randomized to adjuvant chemotherapy versus surgery alone (P=0.03). There was an indication that patients with complete absence of MHC class I expression had a better overall survival compared to patients with a decreased or increased expression. Expression of HLA-G was a negative prognostic marker for the male patients (P=0.002). Also a high infiltration of CD8+ lymphocytes was important in the male patients, where a high frequency of infiltration correlated with a good prognosis (P=0.002). These factors were not, however, significant in the female population. The superior negative prognostic marker in the female patients was HLA-A*02 genotype.

**Conclusions**

HLA-A*02 positivity may be an important marker to determine which colon cancer patients should receive adjuvant chemotherapy. It also seems to determine the outcome of the female patients, which raises new questions as to why this gene, which most likely has the same function in both genders, can be devastating to one gender and not the other.

**References**

1. Gamzatova Z, *et al*: **Human leucocyte antigen (HLA) A2 as a negative clinical prognostic factor in patients with advanced ovarian cancer**. *Gynecol Oncol* 2006, **103(1)**:145-150.

2. Andersson E, *et al*: Correlation of HLA-A02* genotype and HLA class I antigen down-regulation with the prognosis of epithelial ovarian cancer. *Cancer Immunol Immunother* 2012, **61(8)**:1243-1253.

3. Andersson, *et al*: **Non-classical HLA-class I expression in serous ovarian carcinoma: correlation with the HLA-genotype, tumor infiltrating immune cells and prognosis**. *Oncoimmunology* 2015, **5(1)**:e1052213.

**Table 8:** **(Abstract P118).**

		N:o	%
Cohort		520	100
Genus	Women	249	47.8
	Men	271	52.2
Localisation	Colon dx	241	46.3
	Transversum	47	9
	Colon sin	41	8
	Sgmoideum	182	35
	Undertermined	9	1.7
Stage	Duke B	230	44.2
	Duke C	290	55.8
Treatment	Surgery	275	52.9
	Surgery + adj chemotherapy	245	47.1

Cohort descrition

**Fig. 53:**
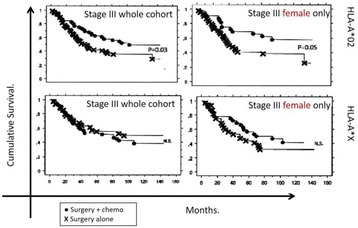
**(Abstract P118).** HLA-genotype and treatment

**Fig. 54:**
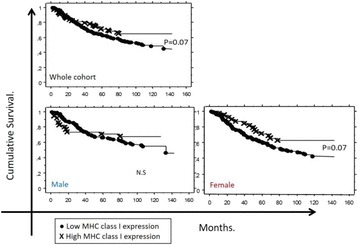
**(Abstract P118).** MHC class I expression

**Fig. 55:**
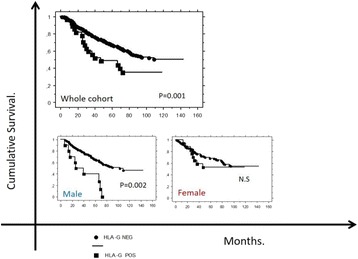
**(Abstract P118).** HLA G expression

**Fig. 56:**
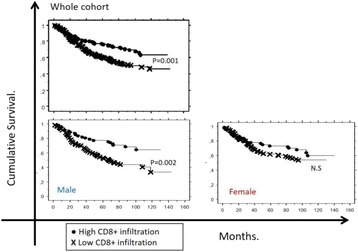
**(Abstract P118).** CD8+ infiltration

**Fig. 57:**
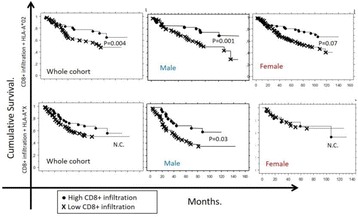
**(Abstract P118).** CD8+ infiltration and HLA-genotype

### P119 Profiling immune cell populations and functional state with simultaneous, multiplexed detection of RNA and protein on the nCounter® platform

#### Gary Geiss, Brian Birditt, Qian Mei, Alan Huang, Andrew M White, Maribeth A Eagan, Eduardo Ignacio, Nathan Elliott, Dwayne Dunaway, Lucas Dennis, Sarah Warren, Joseph Beechem

##### NanoString Technologies, Seattle, WA, USA

###### **Correspondence:** Sarah Warren (swarren@nanostring.com)

**Background**

One of the biggest challenges facing the field of immuno-oncology is development of a comprehensive understanding of how the immune system responds to a tumor. Multi-analyte profiling (DNA, RNA, and protein) from limited sample is crucial to furthering our understanding of tumor immunity. NanoString’s nCounter® technology has become an important platform for quantification of transcriptional responses by enabling direct digital quantification of up to 800 RNA targets from a single sample.

**Methods**

Now the nCounter technology can simultaneously quantify 770 RNA transcripts plus 30 proteins from as few as 20,000 cells (up to 50,000 primary cells such as PBMCs). NanoString has developed content and methods to allow digital quantification of both cell surface and intracellular protein targets that are essential to immuno-oncology research. These key targets include immune cell population markers, immune checkpoint proteins, transcription factors, chemokines, and cytokines. Protein detection is enabled via primary antibodies, which are covalently linked to single stranded DNA indexing oligos. Cells are stained with a multiplexed cocktail of labeled antibodies, and DNA oligos are subsequently released via cell lysis for hybridization to optical barcodes in the standard NanoString workflow. This technique enables quantitative, multiplexed protein detection over 5 logs of dynamic range.

**Results**

As proof of concept, RNA and protein were measured simultaneously from just 50,000 PBMCs treated with phorbol 12-myristate 13-acetate (PMA) and ionomycin, TNFα, IFNγ, or anti-CD3/CD28. NanoString’s multiplex RNA and protein detection provides a thorough evaluation of the immunological response in these experiments and demonstrates the value of multi-analyte profiling from the same sample by characterizing the breadth of the response (via 770 RNA measurements) and providing depth to the analysis (via tandem measurements of RNA and protein for key targets). As independent confirmation, the findings were validated with flow cytometry.

**Conclusions**

This advance in multi-analyte, multiplexed digital molecular profiling with low sample input will accelerate immuno-oncology research and may enable the discovery and development of novel immunotherapies and their associated companion diagnostics.

### P120 Spatially-resolved, multiplexed digital characterization of protein and mRNA abundance in FFPE tissue sections: application to immuno-oncology

#### Dwayne Dunaway, Jaemyeong Jung, Chris Merritt, Isaac Sprague, Philippa Webster, Yan Liang, Sarah Warren, Joseph Beechem

##### NanoString Technologies, Seattle, WA, USA

###### **Correspondence:** Sarah Warren (swarren@nanostring.com)

**Background**

Characterization of the abundance, distribution, and colocalization of key immunoregulatory proteins within the tumor microenvironment is necessary for a thorough understanding of tumor immune responses. Historically, immunohistochemistry and immunofluorescence have been used to assess spatial heterogeneity of proteins and nucleic acids in tissue slices, but these techniques are of limited utility due to the challenge of measuring multiple targets in parallel. We have developed a platform to enable spatially-resolved protein and RNA detection with the potential to simultaneously quantify up to 800 targets with greater than 5 logs of dynamic range from a single formalin-fixed paraffin-embedded (FFPE) slide.

**Methods**

The technology uses DNA oligo tags covalently linked to detection reagents (primary antibodies or mRNA-binding probes) via a UV photocleavable linker to identify targets in situ and enable quantitation via the standard nCounter® technology. A slide-mounted FFPE tissue section is incubated with a cocktail of oligo-labeled primary antibodies or mRNA binding probes, and a serial section is stained with low-plex visible/fluorescent probes (e.g., nuclear staining probes, or select antibody pairs such as anti-CD3 and anti-CD8) to generate an image of the FFPE tissue slice morphology. Regions of interest (ROI) in the tumor are then identified and sequentially illuminated with UV light to release the DNA-oligos. Following UV illumination, the photocleaved oligos are released into the aqueous layer above the tissue slice, collected via microcapillary aspiration, and stored in an individual well of a microtiter plate. Oligos are then hybridized to nCounter optical barcodes to permit *ex situ* digital counting of as many as 800 different analytes localized within a single ROI in the tumor, which can be referenced using image capture software.

**Results**

We demonstrate validation of this technology by characterization of a panel of immune proteins on FFPE tumor biopsies and tissue microarrays. We extend this technology to spatially-resolved multiplexed detection of RNA on a number of important immune targets (e.g., CD3, CD45). We further demonstrate that this approach enables protein detection at single cell resolution and enables simultaneous multiplexed detection of CD3, CD4, CD8, CD45, CD45R0, PD-1, PD-L1, Vista, TIM-3, B7-H3, Ki67 and additional key immuno-oncology-(IO) targets. Finally, we demonstrate detection of key IO RNA targets using direct hybridization of oligo-labeled probes.

**Conclusions**

The ability to measure DNA, RNA, and protein at up-to 800-plex from single slices of FFPE tissue may enable the discovery of key immune biomarkers in tumors and accelerate the development immunotherapy and their associated companion diagnostics.

### P121 Immunological biomarkers correlate to survival in CAR19-treated patients

#### Jessica Wenthe^1^, Gunilla Enblad^1^, Hannah Karlsson^1^, Magnus Essand^1^, Barbara Savoldo^2^, Gianpietro Dotti^2^, Martin Höglund^1^, Malcolm K Brenner^2^, Hans Hagberg^1^, Angelica Loskog^1^

##### ^1^Uppsala University, Uppsala, Uppsala Lan, Sweden; ^2^Baylor College of Medicine, Houston, TX, USA

###### **Correspondence:** Jessica Wenthe (jessica.wenthe@igp.uu.se)

**Background**

CD19-targeting chimeric antigen receptor (CAR) T cell therapy achieved remarkable results in patients with acute lymphoblastic leukemia. However, few studies have shown promising results in patients with other CD19^+^ B cell malignancies, including lymphomas. We have completed a trial treating 15 patients with various B cell malignancies with third generation CD19-targeting (CD28/4-1BB) CAR T cells with or without preconditioning using a moderate dose of cyclophosphamide (500mg/m^2^) and fludarabine (25mg/m^2^). Herein, we report the results of a biomarker screening pre and post CAR T cell treatment using the trial biobank.

**Methods**

A biobank consisting of plasma and peripheral blood mononuclear cells obtained from CAR T cell treated patients with relapsed or refractory CD19^+^ B cell malignancy was analyzed for immunological biomarkers. Samples were analyzed for immunosuppressive cells by flow cytometry and for systemic biomarkers using a 233-analyte proteomic analysis (ProSeek). Of the 15 treated patients, 11 patients had lymphoma and 6 had leukemia. During CAR T cell manufacture, lymphoma patients received standard treatment to control tumor burden. All patients were treated with one infusion of CAR T cells with dose ranges from 2x10^7^ to 2x10^8^ cells/m^2^. The two last patients received anti-PD-1 antibody therapy post CAR infusion. Correlation of biomarkers with survival was assessed by Spearman correlation.

**Results**

The patients were grouped into initial complete responders (6/15) and long-term survivors (4/15). In patient blood, CAR mRNA peaked at 1 week post infusion and could still be detected with varying levels over time up to 12 months post infusion. Baseline levels of monocytic myeloid-derived suppressor cells were negatively correlated with survival (P=0.0231) and M2-like macrophages tended to be increased at the time of relapse compared to the time of complete response (Mean %HLA-DR^+^CD80^-^CD163^+^ of CD11b^+^cells: 15.4% vs. 8.3%). The proteomic analysis is ongoing. So far, several chemokines (CXCL16, CCL16, CCL15) as well as Th2 response and macrophage-associated molecules (IL8, IL10, IL33R, GDF-15) are correlated negatively with survival, whereas IL12, 41BB, TRAIL and transferrin receptor show a positive correlation.

**Conclusions**

In summary, we analyzed immunological biomarkers in 15 patients treated with CAR T cells in Sweden. An increase of M2-like macrophages was seen prior to relapse and molecules connected with suppressive responses were correlated negatively to survival including angiogenic drivers such as IL8 and CCL16 warranting a trial combining CAR T cells with angiogenic inhibitors. Instead, Th1-related IL12 and 41BB correlated with a longer survival time.

**Trial Registration**

ClinicalTrials.gov identifier NCT02132624.

## Bispecific Antibodies

### P122 Multiple bispecific checkpoint combinations enhance T cell activity

#### Matthew J. Bernett, Gregory L. Moore, Michael Hedvat, Christine Bonzon, Seung Chu, Rumana Rashid, Kendra N Avery, Umesh Muchhal, John Desjarlais

##### Xencor, Inc., Monrovia, CA, USA

###### **Correspondence:** John Desjarlais (jrd@xencor.com)

**Background**

Combination checkpoint blockade has demonstrated increased clinical responses relative to single checkpoint blockade. However, the combination of nivolumab plus ipilimumab has also exhibited increased immune-related adverse events. We reasoned that dual checkpoint blockade could be accomplished using a bispecific antibody format, with the potential benefits of reduced cost and more selective targeting of tumor-reactive lymphocytes to improve safety. Numerous checkpoint receptors exist, including PD-1, CTLA-4, LAG-3, BTLA, and others, encouraging the exploration of multiple bispecific combinations.

**Methods**

Antibodies specific to PD-1, CTLA-4, LAG-3, and BTLA were assembled into a bispecific antibody format in select combinations and optimized for stability and affinity. *In vitro* T cell stimulation activity of these bispecific antibodies was measured in a SEB stimulation assay. IL-2 and IFNγ production was measured by ELISA. *In vivo* activity was evaluated by engrafting human PBMCs into NSG mice (huPBMC-NSG) and measuring the extent of T cell engraftment by flow cytometry after weekly treatment. An anti-tumor model was also developed with the huPBMC-NSG system, in which KG1a AML cells were first inoculated and allowed to establish tumors, followed by engraftment of huPBMC and treatment with antibody.

**Results**

We produced PD-1 x CTLA-4, PD-1 x LAG-3, CTLA-4 x LAG-3, and PD-1 x BTLA bispecific antibodies. All of the bispecifics enhanced IL-2 production in an *in vitro* SEB stimulation assay relative to control, indicating functional checkpoint blockade. In most cases, IL-2 production was similar or superior to that of anti-PD-1 alone. Treatment of huPBMC-NSG mice with checkpoint bispecifics promoted enhanced T cell engraftment relative to control. Engraftment levels promoted by bispecifics were generally superior to those found for anti-PD-1 treatment alone, and in some cases comparable to a combination of anti-PD-1 and anti-CTLA-4 antibodies. In one run of the model, while anti-PD-1 treatment alone promoted a 7-fold increase in human CD45+ cells, a PD-1 x CTLA-4 bispecific antibody induced a 22-fold increase, and a CTLA-4 x LAG-3 bispecific promoted a 12-fold increase. Combining CTLA-4 x LAG-3 with anti-PD-1 to achieve triple checkpoint blockade promoted a 67-fold increase in human CD45+ cells, leading to severe graft versus host disease. The bispecific antibodies also exhibited compelling anti-tumor activity in a mouse AML model.

**Conclusions**

Dual checkpoint blockade with bispecific antibodies is feasible and promotes strong T cell activation *in vitro* and *in vivo*. Multiple combinations display compelling activity, suggesting clinical development is warranted for the treatment of human malignancies.

### P123 Dual blockade of PD-1 and CTLA-4 with bispecific antibodies promotes human T cell activation and proliferation

#### Michael Hedvat, Matthew J Bernett, Gregory L Moore, Christine Bonzon, Rumana Rashid, Seung Chu, Kendra N Avery, Umesh Muchhal, John Desjarlais

##### Xencor Inc., Monrovia, CA, USA

###### **Correspondence:** Michael Hedvat (mhedvat@xencor.com)

**Background**

Treatment of melanoma patients with nivolumab plus ipilimumab increases progression-free-survival compared to each monotherapy. The increase in efficacy of the combination regimen is accompanied by an increase in adverse events. Since PD-1+CTLA-4+ tumor-infiltrating-lymphocytes are dysfunctional in the tumor microenvironment, we attempted to specifically target PD-1+CTLA-4+ double-positive T cells with a PD-1 x CTLA-4 bispecific antibody in an effort to recapitulate efficacy of the combination regimen while reducing toxicity.

**Methods**

Antibodies binding to PD-1 and CTLA-4 with favorable stability and functional properties were assembled in a bispecific antibody platform with substitutions in the Fc domain to suppress effector function. PD-1 x CTLA-4 bispecific antibodies were evaluated *in vitro* by measuring antibody binding and de-repression of super-antigen stimulated peripheral blood lymphocytes (PBMCs) and *in vivo* by monitoring the engraftment of human PBMCs in NSG mice (huPBMC-NSG) by flow cytometry. To evaluate anti-tumor efficacy we monitored the growth of established KG1a AML cancer cells in huPBMC-NSG following treatment.

**Results**

Optimized candidate single-chain Fvs were confirmed to bind PD-1 and functionally block PD-L1 and PD-L2 binding to PD-1. We also generated optimized anti-CTLA-4 Fabs. Anti-PD-1 and anti-CTLA-4 targeting components were assembled into a bispecific format and displayed favorable biophysical and manufacturing properties. PD-1 x CTLA-4 bispecific antibodies enhanced IL-2 secretion 5-fold relative to control (p<0.001, n=18 donors) in response to antigenic challenge of previously stimulated T cells, with 2-fold superior activity compared to anti-PD1 bivalent antibody (p<0.001, n=18 donors). PD-1 x CTLA-4 bispecific antibodies significantly enhanced T cell engraftment in huPBMC-NSG mice compared to vehicle controls (p<0.001, n=10/group). PD-1 x CTLA-4 bispecific antibodies promoted higher T cell engraftment than anti-PD-1 alone (p<0.01, n=10/group) and similar engraftment to a combination of anti-PD-1 and anti-CTLA-4 bivalent antibodies. PD-1 x CTLA-4 bispecific antibodies also exhibited compelling anti-tumor activity in a mouse AML model.

**Conclusions**

Dual blockade of PD-1 and CTLA-4 with a bispecific antibody results in T cell activation that is comparable to a combination of bivalent antibodies targeting PD-1 and CTLA-4. Specific targeting of human lymphocytes positive for both PD-1 and CTLA-4 with a bispecific antibody may promote similar efficacy compared to a combination of bivalent antibodies while reducing adverse events. These data suggest that clinical development of a PD-1 x CTLA-4 bispecific antibody is warranted for the treatment of human malignancies.

### P124 A LAG-3/PD-L1 bispecific antibody inhibits tumor growth in two syngeneic colon carcinoma models

#### Matthew Kraman, Katarzyna Kmiecik, Natalie Allen, Mustapha Faroudi, Carlo Zimarino, Mateusz Wydro, Jacqueline Doody

##### F-star Biotechnology Ltd., Cambridge, England, UK

###### **Correspondence:** Matthew Kraman (matthew.kraman@f-star.com)

**Background**

Lymphocyte activation gene-3 (LAG-3) is a member of the immunoglobulin superfamily expressed on activated T cells, NK cells, pDCs, B cells, and γδ T cells, and participates in immune suppression, particularly through persistent strong expression in a percentage of regulatory T cells (Tregs). Programmed cell death receptor (PD-1) binds to its ligand PD-L1, expressed not only on activated immune cells to inhibit cellular immune responses but also on tumor cells. Expression of LAG-3 and PD-1 leads to T cell exhaustion, allowing tumor escape from immune surveillance. Combining inhibitory antibodies to PD-1 and LAG-3 reactivates T cells leading to efficacy in murine models over single treatment [1] and prompted interest in combining these two in the development of a LAG-3 and PD-L1 bispecific antibody for inhibiting tumor growth. This format may additionally result in novel mechanisms of action that are impossible to attain with combinations.

**Methods**

A murine-specific anti-LAG-3 and PD-L1 bispecific antibody was engineered and analyzed for binding, blocking, and preventing LAG-3 and PD-L1-mediated T cell suppression *in vitro*. In addition, the LAG-3/PD-L1 bispecific antibody was tested in 2 syngeneic murine tumor models for its ability to suppress tumor growth.

**Results**

Not only is the bispecific anti-LAG-3/PD-L1 antibody (mAb^2^ ^TM^) able to bind both antigens simultaneously and with nanomolar affinities, but it also demonstrates functional activity against both targets *in vitro*. This potency translates into *in vivo* efficacy,where the anti-LAG-3/PD-L1 mAb^2^ decreased tumor burden in the MC38 colon carcinoma tumor model, both in early-established or well-established tumors. At the end of the study tumor-free animals were more numerous in the LAG-3/PD-L1 bispecific group than in the group given a combination of individual anti-LAG-3 and PD-L1 antibodies. The results were recapitulated in the CT26 murine colon cancer model, where the LAG-3/PD-L1 mAb^2^ showed an increase of anti-tumor activity as compared to the combination of anti-LAG-3 and anti-PD-L1 antibodies.

**Conclusions**

The promising preclinical results from this anti-mouse LAG-3/PD-L1 bispecific antibody supports continuing to progress with the development of an anti-human LAG-3/PD-L1 mAb^2^ for use in the treatment of cancer patients.

**Acknowledgements**

Alasdair Bell, Maude Berthelot, Katy Everett, Alexander Koers, Teresa Mallia, Emma McConnell, and Sarka Pechouckova.

**References**

1. Woo SR, *et al*: **Immune inhibitory molecules LAG-3 and PD-1 synergistically regulate T-cell function to promote tumoral immune escape.** *Cancer Res* 2012, **72(4)**:917-927.

### P125 A fusion monoclonal antibody (FmAb2) to target tumors and the immune system with a unique mechanism of action: preclinical proof-of-concept

#### Sreesha P. Srinivasa, Nagaraja Govindappa, Praveen Reddy, Aparajita Dubey, Sankar Periyasamy, Madhukara Adekandi, Chaitali Dey, Mary Joy

##### Biocon Research Ltd, Bangalore, India

###### **Correspondence:** Sreesha P. Srinivasa (sreeshap.srinivasa@biocon.com)

**Background**

We describe a novel therapeutic molecule that simultaneously inhibits two important targets which control pathways that are hallmarks of tumorigenesis. Epidermal growth factor receptor (EGFR) is important for the growth and survival of tumor cells, and transforming growth factor beta (TGFb) is a pleiotropic cytokine expressed in the tumor microenvironment that regulates important processes such as epithelial-to-mesenchymal cell transition (EMT), migration, invasion, and tumor-specific immunosuppression. To date, therapies have targeted these pathways individually; monoclonal antibodies (mAbs) and small molecule inhibitors of EGFR are in clinical use, and modulators of TGFb pathway are in late-stage clinical development.

**Methods**

FmAb2 is a recombinant fusion mAb consisting of the TGFb receptor II extracellular domain (T-ECD) fused to cetuximab, an anti-EGFR mAb. We hypothesized that using FmAb2 to block the EGFR and TGFb pathways simultaneously would have a synergistic effect on inhibition of tumor growth and EMT, and also induce a tumor-specific immune response. Furthermore, this agent would neutralize TGFb preferentially in the tumor microenvironment, thereby improving efficacy while reducing systemic toxicity.

**Results**

FmAb2 was expressed in Chinese hamster ovary (CHO) cells, purified using standard methodology and tested using *in vitro* and *in vivo* models. FmAb2 is secreted as an intact fusion protein and simultaneously binds both targets in *in vitro* binding assays. In surface plasmon resonance kinetic binding assays (Biacore), FmAb2 binds EGFR with a K_D_ of ~2.5 nM and TGFb1 with a K_D_ of ~60 nM. FmAb2 binds EGFR on the cell surface, neutralizes tumor cell-secreted TGFb1, inhibits proliferation of sensitive tumor cell lines and activates NK cell-mediated antibody-dependent cellular cytotoxicity (ADCC). Using fluorescently labeled FmAb2, we demonstrate that systemically administered FmAb2 in mice preferentially localizes to tumors and is 10-fold more effective in neutralizing tumor TGFb1 compared to T-ECD fused to IgG-Fc (T-ECD-Fc). Consistent with this, we also observe significantly better inhibition of tissue inhibitor of metalloproteinase-1 (TIMP-1) in FmAb2-treated tumors compared to T-ECD at equivalent doses. In a head-and-neck mouse xenograft model, FmAb2 administration is significantly superior to cetuximab or T-ECD-Fc alone at equivalent doses. Furthermore, FmAb2 treatment is more efficacious than the co-administration of cetuximab + T-ECD-Fc at equivalent doses, most likely due to better neutralization of tumor TGFb1.

**Conclusions**

FmAb2 is currently in preclinical development and is expected to enter clinical testing soon.

### P126 MCLA-117, a CLEC12AxCD3 bispecific IgG targeting a leukemic stem cell antigen, induces T cell mediated AML blast lysis

#### Pieter Fokko van Loo^1^, Henrike Veninga^1^, Setareh Shamsili^1^, Mark Throsby^1^, Harry Dolstra^2^, Lex Bakker^1^

##### ^1^Merus N.V., Utrecht, Netherlands; ^2^Department of Laboratory Medicine, Radboud University Medical Center, Nijmegen, Gelderland, Netherlands

###### **Correspondence:** Pieter Fokko van Loo (p.vanloo@merus.nl)

**Background**

Patients with acute myeloid leukemia (AML) have a dismal prognosis despite improvements in chemotherapy and supportive care. Novel, more effective therapies are needed for these patients. We report the characterization of MCLA-117, a novel T cell redirecting antibody for the treatment of AML that targets CLEC12A on leukemic cells and CD3 on T cells. CLEC12A is a myeloid differentiation antigen that is expressed on 90-95% of newly diagnosed and relapsed AML. Moreover, CLEC12A is selectively expressed on leukemic stem cells (LSCs) but not on normal early hematopoietic progenitors, including hematopoietic stem cells (HSCs).

**Methods**

MCLA-117 is a human common light chain CLEC12AxCD3 full length IgG1 bispecific antibody. Heterodimerization of the bispecific IgG is facilitated by amino acid residues introduced at the CH3 interface. MCLA-117 lacks Fc effector function following amino acid substitutions in the CH2 region.

**Results**

**Fig. 58:**
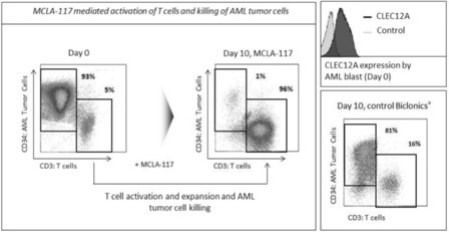
**(Abstract P126).** MCLA-117 efficiently induced T cell expansion and redirected lysis of AML blast in primary AML samples even at a low E:T ratio. Primary AML blast sample taken at diagnosis was incubated with MCLA-117 and a control Biclonics (binding CD3 and an irelevant antigen). CLEC12A expression, T cell proliferation and primary AML blast lysis was quantified using flow cytometry

MCLA-117 specifically binds to CLEC12A expressing myeloid cells and CD3 expressing T cells, as evaluated using healthy donor samples. In line with the CLEC12A expression MCLA-117 did not bind the HSCs, nor the common myeloid progenitor cells. The potency of MCLA-117 to induce T cell mediated lysis of CLEC12A^POS^ tumor cells was evaluated in cytotoxicity assays. In co-cultures with resting healthy donor T cells and AML tumor cells, MCLA-117 efficiently induced CLEC12A antigen dependent T cell activation (EC_50_ of 44 ng/mL), T cell proliferation and tumor target cell lysis (EC_50_ of 66±37 ng/mL). The efficacy of MCLA-117 to induce lysis of AML blasts by autologous T cells in primary AML samples with low T cell to AML blast ratios was examined in an *ex vivo* culture system. We demonstrated robust eradication of AML blasts by MCLA-117, even at low E:T ratios (Fig. 58). In 9/11 AML patient samples taken at diagnosis, MCLA-117 induced expansion of autologous T cells (7-226-fold) and killing (31-99%) of AML blasts at low E:T ratios (E:T 1:3–1:97).

**Conclusions**

MCLA-117 efficiently induced CLEC12A-mediated lysis of AML blasts by T cells present in AML samples, even at very low E:T ratios, and it provoked robust T cell proliferation. Based on its binding profile within the hematopoietic compartment, MCLA-117 is expected to specifically target myeloid blasts and LSCs while sparing the normal HSCs. A phase I clinical study (MCLA-117-CL01) is ongoing to evaluate the preliminary safety and efficacy of MCLA-117 in adult AML patients.

## Clinical Trials in Progress

### P127 KEYNOTE-361: randomized phase III study of pembrolizumab with or without chemotherapy versus chemotherapy alone in advanced urothelial carcinoma

#### Ajjai Alva^1^, Juergen Gschwendt^2^, Yohann Loriot^3^, Joaquim Bellmunt^4^, Dai Feng^5^, Christian Poehlein^5^, Thomas Powles^6^

##### ^1^University of Michigan, Ann Arbor, MI, USA; ^2^Technical University of Munich, Munchen, Germany; ^3^Gustave Roussy, Université Paris-Saclay, Villejuif, France; ^4^Dana-Farber Cancer Institute, Harvard Medical School, Boston, MA, USA; ^5^Merck & Co., Inc., Kenilworth, NJ, USA; ^6^Barts Cancer Institute, Queen Mary University of London, London, England, UK

###### **Correspondence:** Ajjai Alva (ajjai@med.umich.edu)

**Background**

Cisplatin-based combination chemotherapy is standard first-line treatment for patients with advanced bladder cancer. The median survival with these regimens is 13-15 months, and only 5-15% of patients attain long-term survival. Programmed death ligand 1 (PD-L1) is expressed in select urothelial cancer lesions. In the phase Ib KEYNOTE-012 study, monotherapy with the anti–programmed death 1 antibody pembrolizumab demonstrated antitumor activity (objective response rate, 28 %; 95 % CI, 13 %-47 %) and acceptable safety in patients with chemotherapy-refractory metastatic PD-L1–positive urothelial carcinoma, and higher tumor PD-L1 expression was associated with higher response rates. KEYNOTE-361 is a randomized, open-label, phase III study of pembrolizumab with or without chemotherapy versus chemotherapy alone in patients with advanced urothelial carcinoma.

**Methods**

Key eligibility criteria include age ≥18 years; histologically or cytologically confirmed diagnosis of advanced/unresectable or metastatic urothelial carcinoma of the bladder, renal pelvis, ureter, or urethra; measurable disease based on RECIST v1.1 as determined by the local site investigator/radiology assessment; no prior systemic chemotherapy for advanced/metastatic urothelial carcinoma (with the exception of neoadjuvant platinum-based chemotherapy with recurrence >12 months after completion of therapy; or adjuvant platinum-based chemotherapy following radical cystectomy with recurrence >12 months after completion of therapy); ECOG performance status 0-2; and provision of a fresh tissue sample for biomarker analysis. Approximately 990 patients will be randomized 1:1:1 to receive: pembrolizumab 200 mg on day 1 of each 3-week cycle (Q3W) alone or in combination with investigator’s choice of chemotherapy (gemcitabine [1000 mg/m^2^ on days 1 and 8 of each 3-week cycle] plus cisplatin [70 mg/m^2^ Q3W] or gemcitabine plus carboplatin [AUC 5 Q3W] for cisplatin-ineligible patients), or investigator’s choice of chemotherapy alone. Patients will be stratified based on chemotherapy regimen (cisplatin or carboplatin, chosen before randomization) and tumor PD-L1 expression (positive or negative). Treatments will continue until progressive disease, unacceptable adverse events (AEs), investigator decision, or 35 doses of pembrolizumab (pembrolizumab arms only). Response will be assessed per RECIST v1.1 by blinded independent central review (BICR) every 9 weeks until week 54, then every 12 weeks thereafter. AEs will be evaluated throughout treatment and for 30 days thereafter (90 days for serious AEs) and graded per National Cancer Institute Common Terminology Criteria for Adverse Events, version 4.0. The primary end points are PFS per RECIST v1.1 as assessed by BICR and OS; key secondary end points include objective response rate and safety and tolerability. Enrollment in KEYNOTE-361 is ongoing.

**Trial Registration**

ClinicalTrials.gov identifier NCT02853305.

### P128 Phase II study of pembrolizumab in patients with metastatic castration-resistant prostate cancer previously treated with targeted endocrine therapy and taxane chemotherapy: KEYNOTE-199

#### Emmanuel S Antonarakis^1^, Charles G Drake^2^, Haiyan Wu^3^, Christian Poehlein^3^, Johann De Bono^4^

##### ^1^Johns Hopkins University, Sidney Kimmel Cancer Center, Baltimore, MD, USA; ^2^Johns Hopkins University Cancer Center, Baltimore, MD, USA; ^3^Merck & Co., Inc., Kenilworth, NJ, USA; ^4^Royal Marsden Hospital, London, England, UK

###### **Correspondence:** Emmanuel S Antonarakis (eantona1@jhmi.edu)

**Background**

Treatment for patients with metastatic castration-resistant prostate cancer (mCRPC) has focused on suppression of testosterone and androgen receptor signaling, palliative radiation therapy, and chemotherapy. As expression of the programmed death-1 (PD-1) receptor and its ligand PD-L1 is present in mCRPC lesions, particularly after initial treatment with antiandrogen and/or chemotherapy, targeting this pathway may be an attractive treatment option. Pembrolizumab, an anti-PD-1 antibody that blocks interaction between PD-1 and its ligands, PD-L1 and PD-L2, produced durable responses in patients with heavily pretreated PD-L1-positive prostate cancer in the KEYNOTE-028 study. KEYNOTE-199 is a nonrandomized, multinational, open-label phase II study to evaluate pembrolizumab monotherapy in patients with mCRPC previously treated with chemotherapy.

**Methods**

Eligible patients must be ≥18 years old with histologically or cytologically confirmed adenocarcinoma of the prostate without small-cell histology, measurable disease per RECIST v1.1 or detectable bone metastases by whole-body bone scintigraphy and no RECIST v1.1 measurable tumors, supplied tumor sample for PD-L1 expression (new or archived), progression of disease within 6 months before screening, and ECOG performance status 0-2. Patients must have been treated with ≥1 targeted endocrine therapy (abiraterone or enzalutamide) and ≤2 chemotherapy regimens, 1 of which must have contained docetaxel. Patients also must have ongoing androgen deprivation with serum testosterone <50 ng/dL. Patients will be enrolled into 1 of 3 cohorts based on PD-L1 status and RECIST v1.1 measurability: patients with PD-L1-positive, RECIST v1.1 measurable disease (cohort 1, n=100), patients with PD-L1-negative, RECIST v1.1 measurable disease (cohort 2, n=100), and patients with bone metastases and RECIST v1.1 nonmeasurable disease (cohort 3, n=50). Patients will receive pembrolizumab 200 mg every 3 weeks until documented confirmed disease progression, unacceptable adverse events (AEs), or illness that prevents further treatment. Imaging response will be assessed every 9 weeks for approximately 1 year and every 12 weeks thereafter, per central imaging vendor review using RECIST v1.1 criteria and the Prostate Cancer Clinical Trials Working Group 3 guidelines. AEs will be monitored throughout the study and graded per Common Terminology Criteria for Adverse Events, version 4.0. Primary end points are the objective response rate and duration of response for cohorts 1 and 2 combined and by each cohort. Key secondary end points include safety and tolerability, disease control rate, radiographic progression-free survival, and overall survival for each cohort and all 3 combined. Exploratory translational analyses and expression of other immune checkpoints will also be evaluated.

**Trial Registration**

ClinicalTrials.gov identifier NCT02787005.

### P129 KEYNOTE-155: phase Ib study of pembrolizumab in combination with dinaciclib in patients with hematologic malignancies

#### Rajat Bannerji^1^, John Byrd^2^, Gareth Gregory^3^, Stephen Opat^4^, Jake Shortt^4^, Andrew J Yee^5^, Noopur Raje^5^, Seth Thompson^6^, Arun Balakumaran^6^, Shaji Kumar^7^

##### ^1^Rutgers Cancer Institute of New Jersey, New Brunswick, NJ, USA; ^2^The Ohio State University, Columbus, OH, USA; ^3^Monash Health and Peter MacCallum Cancer Centre, Clayton, Victoria, Australia; ^4^Monash Health, Clayton, Victoria, Australia; ^5^Massachusetts General Hospital, Boston, MA, USA; ^6^Merck & Co., Inc., Kenilworth, NJ, USA; ^7^Mayo Clinic, Rochester, MN, USA

###### **Correspondence:** Rajat Bannerji (bannerra@cinj.rutgers.edu)

**Background**

Pembrolizumab, a PD-1 immune checkpoint inhibitor, and dinaciclib, a cyclin-dependent kinase inhibitor, have demonstrated antitumor activity as monotherapies in various tumor types, including hematologic malignancies. In a preclinical study using a solid-tumor syngeneic model (MC38), enhanced antitumor activity was observed with the combination of pembrolizumab and dinaciclib, with no significant toxicities. KEYNOTE-155 is a phase Ib study designed to evaluate the safety and efficacy of the combination of pembrolizumab and dinaciclib in patients with relapsed or refractory chronic lymphocytic leukemia (CLL), multiple myeloma (MM), or diffuse large B cell lymphoma (DLBCL).

**Methods**

Key eligibility criteria include ≥18 years, ECOG performance status 0-1, and confirmed diagnosis of one of the following: CLL as defined by 2008 iwCLL criteria with ≥1 prior therapy; active MM with measurable disease and ≥2 prior therapies including an IMiD and proteasome inhibitor; or DLBCL with progression following ≥2 prior therapies including autologous stem cell transplantation (ASCT; or ineligible for ASCT). An initial cohort of 12 patients (≥3 with each disease type) will be enrolled in the dose-evaluation phase (two 21-day cycles) to determine dose-limiting toxicities (DLTs). In cycle 1, patients will receive pembrolizumab 200 mg on day 1, dinaciclib 7 mg/m^2^ on day 1, and dinaciclib 10 mg/m^2^ on day 8. In cycle 2 and beyond, patients will receive pembrolizumab 200 mg on day 1 and dinaciclib 14 mg/m^2^ on days 1 and 8. If ≤4 patients experience DLTs in the first 2 cycles, expansion cohorts (~30 patients each) will be opened for signal detection. If ≥5 DLTs occur in the first 2 cycles, a lower dose of dinaciclib will be studied in up to 24 patients. Treatment will continue until disease progression, unacceptable toxicity, or 35 cycles. Response will be assessed every 3 cycles for CLL and DLBCL, and at the start of each cycle for MM. Primary end points are safety and tolerability in both phases and objective response rate within each disease type per investigator assessment in the signal-detection phase. Secondary end points include duration of response, progression-free survival, and overall survival within each disease type in the signal-detection phase. Exploratory objectives include assessment of treatment effect on CD4, CD8, and quantitative immunoglobulins; the relationship between candidate biomarkers and antitumor activity; and the relationship between genomic variation and response to treatment. KEYNOTE-155 will enroll up to 138 patients from multiple sites.

**Trial Registration**

ClinicalTrials.gov identifier NCT02684617.

### P130 Avelumab in combination with axitinib versus sunitinib as first-line treatment for patients with advanced renal cell carcinoma: the phase III JAVELIN Renal 101 trial

#### Brian I. Rini^1^, Toni K Choueiri^2^, Mariangela Mariani^3^, Laurence Albiges^4^, John B Haanen^5^, Michael B Atkins^6^, James Larkin^7^, Manuela Schmidinger^8^, Domenico Magazzù^3^, Alessandra di Pietro^3^, Robert J Motzer^9^

##### ^1^Cleveland Clinic Taussig Cancer Institute, Cleveland, OH, USA; ^2^Dana-Farber Cancer Institute, Brigham and Women’s Hospital, Boston, MA, USA; ^3^Pfizer Inc., Milano, Lombardia, Italy; ^4^Gustave Roussy Cancer Campus, University of Paris Sud, Department of Cancer Medicine, Villejuif, Ile-de-France, France; ^5^Netherlands Cancer Institute, Amsterdam, Noord-Holland, Netherlands; ^6^Georgetown-Lombardi Comprehensive Cancer Center, Washington DC, USA; ^7^Royal Marsden Hospital, London, England, UK; ^8^Medical University of Vienna, Vienna, Wien, Austria; ^9^Memorial Sloan Kettering Cancer Center, New York, NY, USA

###### **Correspondence:** Brian I. Rini (rinib2@ccf.org)

**Background**

Checkpoint inhibitors and tyrosine kinase inhibitors may have complementary mechanisms of action in advanced renal cell carcinoma (aRCC), providing a rationale for investigating combination treatment. Inhibition of the programmed death-1 receptor ligand (PD-L1) pathway leads to reactivation of an effective antitumor immune response against multiple cancers, including RCC. Avelumab (MSB0010718C) is a fully human IgG1 anti-PD-L1 antibody that has shown clinical activity in patients with several tumor types. Axitinib is an anti-VEGF receptor tyrosine kinase inhibitor approved for treatment of aRCC after failure of 1 prior systemic therapy. In an ongoing phase Ib study, avelumab plus axitinib administered at standard monotherapy doses showed encouraging safety and antitumor activity in treatment-naïve patients with aRCC. JAVELIN Renal 101 is a global phase III trial comparing avelumab plus axitinib versus sunitinib as first-line treatment for patients with aRCC.

**Methods**

The primary objective of this randomized, multicenter trial is to demonstrate superiority of first-line avelumab plus axitinib versus sunitinib monotherapy in prolonging progression-free survival (PFS). Eligibility criteria include: aRCC with a clear cell component, availability of archival or fresh tumor biopsy, ECOG PS ≤1, no prior systemic therapy for advanced disease or prior immunotherapy, and measurable disease per RECIST v1.1. Approximately 583 patients will be randomized 1:1 to receive either: Arm A, avelumab intravenously over 1 hour every 2 weeks plus axitinib orally twice daily continuously (cycle length 6 weeks); or Arm B, sunitinib orally once daily for 4 weeks followed by 2 weeks off. Stratification factors are: ECOG PS (0 vs 1) and region (US vs Canada/Europe vs rest of world). Treatment is discontinued for unacceptable toxicity or if any criteria for withdrawal are met. Patients may continue treatment beyond progression if investigator-assessed clinical benefit is achieved and treatment is well tolerated. The primary endpoint is PFS per RECIST v1.1 by blinded independent central review. Secondary endpoints include overall survival, PFS by investigator assessment, objective response, duration of response, time to response, safety, and patient-reported outcomes. Pharmacokinetics, immunogenicity, and tumor tissue biomarkers will also be assessed. Enrollment in this pivotal trial began in March 2016.

**Trial Registration**

ClinicalTrials.gov identifier NCT02684006.

### P131 T cell therapy in combination with vemurafenib in BRAF mutated metastatic melanoma patients

#### Troels Holz Borch, Rikke Andersen, Per Kongsted, Magnus Pedersen, Morten Nielsen, Özcan Met, Marco Donia, Inge Marie Svane

##### Department of Oncology, Center for Cancer Immune Therapy, Herlev University Hospital, Herlev, Hovedstaden, Denmark

###### **Correspondence:** Troels Holz Borch (troelsholz@hotmail.com)

**Background**

Adoptive T cell therapy (ACT) with tumor infiltrating lymphocytes (TIL) has proven to be a powerful treatment option for patients with metastatic melanoma with response rates of approximately 50% and durable complete responses in about 15%. However, there is still a need for improving TIL efficacy and a promising strategy is combination with immunomodulating agents. One such agent is vemurafenib (vem), a selective BRAF inhibitor, which induces objective responses in about 50% of melanoma patients with tumors expressing BRAF^V600E/K^. In addition to the direct anti-cancer effect, vem has been shown to increase T cell infiltration into tumors, upregulate melanoma antigen expression and increase the frequency of TIL recognizing autologous melanoma cells. This trial was previously presented at the Society for Immunotherapy of Cancer annual meeting [1]. In this abstract an update on clinical responses is provided.

**Methods**

A total of 12 patients will be included in this open phase II non-randomized trial primarily to investigate safety when combining ACT and vem. Secondarily, clinical responses will be evaluated according to RECIST and extensive immune monitoring will be performed. Patients are treated with vem orally 960 mg BID one week prior to excision of tumor material for T cell generation and continue vem until hospital admission (4-7 weeks). During hospitalization patients will receive a preparative lymphodepleting regimen consisting of cyclophosphamide 60 mg/kg for 2 days and fludarabine 25 mg/m^2^ for 5 days. TIL infusion consists of 5-10 x 10^10^ T cells and patients are subsequently treated with continuous interleukin-2 infusion following the decrescendo regimen for 5 days. Patients are evaluated for up to 5 years or until progression.

**Results**

So far 5 patients have been treated. No unexpected toxicity has been observed. Three patients achieved confirmed ongoing partial response (9+ months, 5+, and 4+) and 2 patients had progressive disease at evaluation after TIL. Immune analyses are pending.

**Conclusions**

So far treatment combining vem and ACT using TILs has been safe with no unexpected toxicity and encouraging clinical benefit with durable responses has been observed.

**Trial Registration**

ClinicalTrials.gov identifier NCT02354690.

**References**

1. Borch TH, Andersen R, Kongsted P, *et al*: **T cell therapy in combination with Vemurafenib in BRAF mutated metastatic melanoma patients.** *J Immunother Cancer* 2014, **2(Suppl 3)**:67.

### P132 Neoadjuvant enoblituzumab (MGA271) in men with localized intermediate and high-risk prostate cancer–a pilot and biomarker study

#### Karim Boudadi^1^, Hao Wang^1^, James Vasselli^2^, Jan E Baughman^3^, Jon Wigginton^2^, Rehab Abdallah^1^, Ashley Ross^1^, Charles G Drake^4^, Emmanuel S Antonarakis^5^

##### ^1^Johns Hopkins Hospital, Baltimore, MD, USA; ^2^MacroGenics, Inc., Rockville, MD, USA; ^3^MacroGenics, Inc., South San Francisco, CA, USA; ^4^Johns Hopkins University Cancer Center, Baltimore, MD, USA; ^5^Johns Hopkins University, Sidney Kimmel Cancer Center, Baltimore, MD, USA

###### **Correspondence:** Karim Boudadi (kboudad1@jhmi.edu)

**Background**

B7-H3 is part of the B7 superfamily of immune checkpoint molecules. While its regulation and mechanism of action are not completely understood, B7-H3 expression correlates with adverse pathology and clinical outcomes in men with prostate cancer. B7-H3 expression appears to be associated with biochemical and clinical progression following treatment. The correlation between B7-H3 overexpression and poor prognosis suggests a role in tumor immune escape. In addition, B7-H3 levels remain high in the presence of androgen deprivation. These results suggest a potential role for B7-H3 in prostate cancer progression and support its use as a therapeutic target. Enoblituzumab is an anti-B7-H3 humanized monoclonal antibody with a proposed mechanism of action of antibody directed cellular cytotoxicity. We propose a neoadjuvant study to determine the anti-tumor, immunological and biological effects of enoblituzumab in men with localized prostate cancer. We hypothesize that administration of enoblituzumab will be safe and feasible in the neoadjuvant setting for men with localized intermediate and high-risk prostate cancer. We also hypothesize that enoblituzumab will produce measurable tumor cell death and antitumor immune responses in harvested prostate glands.

**Methods**

This is a single-center, single-arm, open-label pilot study of enoblituzumab 15mg/kg IV weekly for 6 doses beginning 50 days prior to radical prostatectomy. The target population (N=16) is men with localized, intermediate or high-risk prostate adenocarcinoma (Gleason sum ≥7), with adequate organ function, no adverse indications for standard radical prostatectomy, and no previous local treatment to the prostate, anti-androgen exposure, or immunotherapy. The primary endpoint will be frequency/severity of adverse events from time of first administration of enoblituzumab until the 90^th^ post-op day. Prostate glands will be harvested at time of radical prostatectomy and prostate tissue will be examined for the secondary endpoints and compared to pre-treatment biopsies. The secondary biological endpoint is tumor cell death, which will be quantified by caspase 3 staining and post-treatment apoptotic index. The trial will meet its biological endpoint if tumor cell apoptosis is two-fold higher after treatment, which we will be able to detect with an 80% power based on a one-sided t-test with a significance level of 0.05, with 16 subjects. Other secondary endpoints will include CD8+ T cell infiltration, CD8/Treg ratio, TCR repertoire, and B7-H3 expression, and will be exploratory in nature. If this study shows preliminary evidence of clinical activity and reasonable safety, our future goal will be to use enoblituzumab in the adjuvant and salvage settings.

### P133 Radiotherapy enhances natural killer cell homing and function in canine bone and soft tissue sarcoma

#### Robert J. Canter^1^, Jiwon Park^1^, Ziming Wang^1^, Steven Grossenbacher^1^, Jesus I Luna^1^, Sita Withers^2^, William Culp^2^, Mingyi Chen^1^, Arta Monjazeb^1^, Michael S Kent^2^, William J Murphy^1^

##### ^1^University of California, Davis, Sacramento, CA, USA; ^2^University of California, Davis, Davis, CA, USA

###### **Correspondence:** Robert J. Canter (rjcanter@ucdavis.edu)

**Background**

We have previously shown that radiotherapy (RT) increases natural killer (NK) cytotoxicity and homing in diverse pre-clinical models of human solid malignancies, including sarcomas. Since sarcomas commonly afflict dogs, we hypothesized that dog PBMC-derived NK cells would be effective in canine models of sarcoma, including adoptive transfer in a canine RT/NK clinical trial.

**Methods**

Canine NK cells were isolated from 15 mls whole blood using Ficoll separation and CD5 depletion. Isolated NK cells were expanded in co-culture with irradiated K562c9IL21 for 2-3 weeks. Using 6-month metastasis-free survival as the primary endpoint, a canine clinical trial is underway evaluating RT and adoptive intratumoral NK immunotherapy. For this trial, treatment consists of palliative RT weekly for one month followed by two intra-lesional injections of autologous canine NK cells. In correlative studies, including dog patient-derived xenografts (PDX), we assessed NK homing using eFluor 670 cell proliferation dye and NK function by expression of activation markers including IFNγ, granzyme B, and perforin.

**Results**

We have treated 7 of planned 14 dogs with osteosarcoma on protocol. Of 3 evaluable dogs who have reached the 6- month primary endpoint, we have observed 1 partial response and 2 are metastasis-free, including 1 dog with complete resolution of a suspicious 3 mm pulmonary nodule. In dog patients on trial, phenotyping of expanded NK cells from all patients showed > 90% granzyme B and IFNγ expression prior to adoptive transfer. Tagging experiments 1 week after intratumoral injection revealed that 11 – 60% of CD45+ cells are eFluor 670 positive, confirming persistence of injected NK cells post injection. Analysis of unactivated circulating PBMCs post-injection demonstrated a significant increase in granzyme B expression (2.25X ± 0.42, P < 0.01). Dog PDX studies demonstrate that focal RT increases NK homing to sarcomas on average 3.8X± 0.3 (P < 0.001) compared to unirradiated controls. Immunohistochemical analysis of irradiated dog sarcomas (historical controls) shows a significant increase in CD3+ tumor-infiltrating lymphocytes post RT (P < 0.04, see figure). Co-culture experiments of dog PDX sarcomas *ex vivo* with allogeneic NK cells showed RT-induced sensitization to NK killing at doses of 10 - 20 Gy (P < 0.01).

**Conclusions**

RT and NK immunotherapy appear synergistic in dog models of sarcoma, and preliminary results from a canine clinical trial of palliative RT and autologous NK transfer for osteosarcoma are promising, including possible abscopal effects. Further evaluation of this novel radio-immunotherapy approach is warranted.

**Acknowledgements**

The authors are grateful to Teri Guerrero and Heather Schrader for veterinary clinical trials support.

**Fig. 59:**
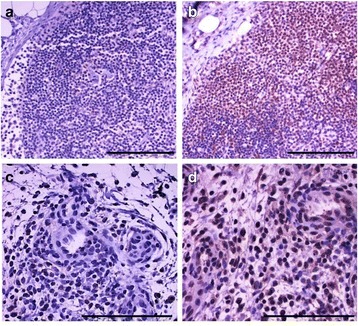
**(Abstract P133).** Canine Sarcomars Show Increased CD3+ Lymphocytes after Radiotherapy. **a** High power view of normal dog lymph node in the absence of mouse anti-dog CD3 antibody. **b** High power view of normal dog lymph node shows diffuse expression of CD3+ lymphocytes. **c** High power view shows absence of CD3+ lymphocytes following amputation of distal tibia dog osteosarcoma. **d** High power view positive increased CD3+ cells in dog osteosarcoma following palliative RT (scale bars 100 μm)

### P134 Interim results of a phase II trial of adoptive transfer of tumor infiltrating lymphocytes in patients with metastatic uveal melanoma

#### Smita Chandran^1^, Robert Somerville^1^, John Wunderlich^1^, David Danforth^1^, James Yang^1^, Richard Sherry^1^, Christopher Klebanoff^1^, Stephanie Goff^1^, Biman Paria^1^, Arvind Sabesan^1^, Abhishek Srivastava^1^, Steven A Rosenberg^2^, Udai Kammula^1^

##### ^1^National Cancer Institute, Bethesda, MD, USA; ^2^Surgery Branch, National Cancer Institute, National Institutes of Health, Bethesda, MD, USA

###### **Correspondence:** Smita Chandran (chandranss@mail.nih.gov)

**Background**

Uveal melanoma (UM) is a rare tumor variant with no established treatments once metastases develop. Although a variety of immune based therapies have demonstrated anti-tumor activity in metastatic cutaneous melanoma, their use in UM has been disappointing. Recently, adoptive T cell therapy has shown salvage responses in multiple refractory solid tumors. Based upon our identification from UM liver metastases of tumor infiltrating lymphocytes (TIL) with strong autologous reactivity, we sought to determine if clinical adoptive transfer of such TIL could mediate regression in patients with metastatic UM. Here we present an interim analysis of the ongoing clinical trial, NCT01814046.

**Methods**

In the main arm of this phase II study, patients with metastatic melanoma of ocular origin received nonmyeloablative lymphodepleting conditioning chemotherapy consisting of intravenous cyclophosphamide (60 mg/kg/daily) for 2 days and fludarabine (25 mg/m^2^/daily) for 5 days followed by an intravenous infusion of autologous TIL and high-dose interleukin-2 (720,000 IU/kg). Clinical responses were evaluated using Response to Evaluation Criteria in Solid Tumors (RECIST), version 1.0. The study was designed to initially enroll 19 patients and if 4 or more patients achieved an objective clinical response, accrual would expand in a second phase to a total of 33 patients.

**Results**

At the current time, 17 patients with metastatic UM have completed TIL treatment; 16 of these patients are evaluable for response assessment. Five of 16 (31%) UM patients demonstrated objective tumor regression. Among the responders, four patients demonstrated partial tumor regression lasting between 4 and 10 months. One patient achieved complete tumor regression, ongoing 15 months after TIL infusion. In the year prior to TIL therapy, this patient demonstrated marked progression of multiple liver metastases after anti-CTLA-4 and anti-PD-1 checkpoint therapy. However, after a single infusion of TIL, we observed rapid and complete tumor regression. Circulating serum tumor DNA specific for the patient’s *GNAQ* driver mutation demonstrated complete clearance by 10 days following cell therapy consistent with the patient’s clinical response. Overall in the trial, the frequency of tumor-reactive T cells within the administered infusion and the levels of IFN-γ released after autologous tumor stimulation were positively associated with clinical response (P=0.04, respectively).

**Conclusions**

To our knowledge, these clinical findings represent the first demonstration that autologous T cell transfer is capable of inducing complete and durable tumor regression of metastatic UM. Refinement of this cell therapy is aimed at identifying the antigenic targets associated with clinical response.

### P135 A phase Ib study of intratumoral CAVATAK (coxsackievirus A21) and ipilimumab in patients with advanced melanoma

#### Brendan Curti^1^, Jon Richards^2^, Mark Faries^3^, Robert H I Andtbacka^4^, Mark Grose^5^, Darren Shafren^6^

##### ^1^Earle A. Chiles Research Institute, Providence Cancer Center, Portland, OR, USA; ^2^Oncology Specialists, S.C., Niles, IL, USA; ^3^John Wayne Cancer Institute, Santa Monica, CA, USA; ^4^University of Utah, Huntsman Cancer Institute, Salt Lake City, UT, USA; ^5^Viralytics, Inc., Sydney, New South Wales, Australia; ^6^Viralytics Limited, Newcastle, New South Wales, Australia

###### **Correspondence:** Brendan Curti (brendan.curti@providence.org)

**Background**

CAVATAK^TM^ is a bio-selected oncolytic and immunotherapeutic strain of coxsackievirus A21 (CVA21). In a phase II study, intratumoral (i.t.) CVA21 injection of advanced melanoma lesions resulted in regression in 28.1% of patients including non-injected lesions, increases in tumor immune-cell infiltration, up-regulation of IFNγ response and immune-checkpoint genes, including CD122 which may be a marker for enhanced anti-tumor activity by anti-CTLA-4 blockade. Preclinical models using B16 ICAM confirmed enhanced antitumor effects when CVA21 was combined with anti-CTLA-4 or anti-PD-1. The preliminary data are presented from the open-label, phase Ib MITCI (Melanoma Intra-Tumoral Cavatak and Ipilimumab) in patients (pts) with advanced melanoma.

**Methods**

Pts received up to 3 x 10^8^ TCID_50_ CVA21 i.t. on study days 1, 3, 5, 8 and 22, and then q3w for up to 14 additional i.t. injections. Ipilimumab (3 mg/kg) was administered q3w for 4 doses starting at Day 22. The first response assessment (irRC) occurred on Day 106. The primary endpoint was to assess safety of CVA21 in combination with ipilimumab treatment (tx). Immune monitoring samples were obtained to analyze T cells with regulatory, memory, and effector phenotypes. Biopsies of injected lesions have been obtained in some patients.

**Results**

No DLT’s have been reported in the 18 patients enrolled. Combination tx has been well-tolerated with only one grade 3 or higher treatment-related AE being ipilimumab-related fatigue. Colitis or other immune related toxicities have been grade 1 or 2. The study met its primary statistical futility endpoint of achieving ≥ 4 confirmed objective responses (CR or PR) in the first 12 pts enrolled. Of the first 11 pts eligible for investigator response assessment, ORR for the ITT population is 57.1% (4/7), with the ORR for ipilimumab-naïve pts being 67% (4/6). The DCR (CR+PR+SD) on the ITT population is currently 86% (6/7). All responses were observed by 3.5 months with complete tumor responses being observed in individual injected and non-injected lesions. One three-fold increase in peripheral blood CD4+ and CD8+ T cells with central and effector memory phenotypes were observed comparing baseline to day 85 in most of the patients analyzed thus far.

**Conclusions**

CVA21 + ipilimumab tx of pts with advanced melanoma has been well tolerated. This combination immunotherapy induced anti-tumor activity in local, visceral and non-visceral lesions in a number of patients that have failed previous immunotherapies. Increases in T cell effector and memory subsets were also observed.

**Trial Registration**

ClinicalTrial.gov identifier NCT01636882.

### P136 KEYNOTE-177: randomized phase III study of pembrolizumab versus investigator-choice chemotherapy for mismatch repair-deficient or microsatellite instability-high metastatic colorectal carcinoma

#### Luis A. Diaz, Jr.^1^, Dung T Le^1^, Takayuki Yoshino^2^, Thierry André^3^, Johanna Bendell^4^, Minori Koshiji^5^, Yayan Zhang^5^, S Peter Kang^5^, Bao Lam^5^, Dirk Jäger^6^

##### ^1^Sidney Kimmel Comprehensive Cancer Center at Johns Hopkins University, Baltimore, MD, USA; ^2^National Cancer Center Hospital East, Kashiwa-shi, Chiba, Japan; ^3^Saint Antoine Hospital, Paris, Ile-de-France, France; ^4^Sarah Cannon Research Institute and Department of Medical Oncology, Tennessee Oncology, Nashville, TN, USA; ^5^Merck & Co., Inc., Kenilworth, NJ, USA; ^6^National Center for Tumor Diseases, Heidelberg, Baden-Wurttemberg, Germany

###### **Correspondence:** Luis A. Diaz, Jr. (ldiaz1@jhmi.edu)

**Background**

A subset of colorectal carcinomas (CRCs) have deficiencies in the mismatch repair (MMR) system, resulting in microsatellite instability (MSI). MSI-high tumors contain high levels of lymphocyte infiltrates and strong expression of the programmed death 1 (PD-1) immune checkpoint receptor and its ligand, PD-L1. Pembrolizumab is an anti–PD-1 monoclonal antibody that blocks the interaction between PD-1 and its ligands, thereby enabling an antitumor immune response. In the phase II KEYNOTE-016 study, pembrolizumab showed promising antitumor activity against MMR-deficient tumors in patients with treatment-refractory metastatic CRC, with an objective response rate of 47% (95% CI, 23%-72%). KEYNOTE-177 (ClinicalTrials.gov, NCT02563002) is an international, randomized, open-label phase III study designed to evaluate the efficacy and safety of pembrolizumab compared with standard-of-care chemotherapy as a first-line treatment for MMR-deficient or MSI-high metastatic CRC.

**Methods**

Key eligibility criteria include age ≥18 years, confirmed MSI-high or MMR-deficient stage IV CRC, measurable disease per RECIST v1.1 by local site assessment, Eastern Cooperative Oncology Group performance status 0-1, no active autoimmune disease or brain metastases, and no prior therapy for metastatic disease. Patients are to be randomized 1:1 to receive either pembrolizumab 200 mg every 3 weeks (Q3W) or investigator’s choice of standard-of-care chemotherapy. Chemotherapy must be chosen prior to randomization; options include mFOLFOX6 or FOLFIRI alone or in combination with bevacizumab or cetuximab. Treatment is to continue until progressive disease, unacceptable toxicity, patient/investigator decision, or completion of 35 cycles (pembrolizumab only). Response is to be evaluated by CT or MRI every 9 weeks per RECIST v1.1 by central imaging vendor review and per RECIST adapted for immunotherapy response patterns. Eligible patients may continue pembrolizumab beyond initial RECIST-defined progression. Patients in the standard-of-care arm who have progressive disease and meet crossover criteria may be eligible to receive pembrolizumab for up to 17 treatment cycles. Adverse events (AEs) are to be assessed throughout treatment and for 30 days thereafter (90 days for serious AEs) and graded per NCI CTCAE v4.0. Patients are to be followed for survival every 9 weeks. The primary end point is progression-free survival per RECIST v1.1; secondary end points are overall survival, objective response rate per RECIST v1.1, and safety and tolerability. Exploratory end points include duration of response and health-related quality of life. Enrollment in KEYNOTE-177 is ongoing, with 270 patients planned to be enrolled.

**Trial Registration**

ClinicalTrials.gov identifier NCT02563002.

### P137 Phase Ia/Ib trial investigating the CSF-1R inhibitor LY3022855 in combination with durvalumab (MEDI4736) or tremelimumab in patients with advanced solid malignancies

#### Todd M. Bauer^1^, Judy S Wang^2^, Jean K Lee^3^, Gulam A Manji^4^, Ragini Kudchadkar^5^, John S Kauh^6^, Shande Tang^6^, Naomi Laing^7^, Gerald Falchook^8^

##### ^1^Sarah Cannon Research Institute, Tennessee Oncology, PLLC, Nashville, TN, USA; ^2^Sarah Cannon Research Institute, Florida Cancer Specialists, Sarasota, FL, USA; ^3^Laura and Isaac Perlmutter Cancer Center at NYU Langone Medical Center, New York, NY, USA; ^4^Columbia University Medical Center, New York, NY, USA; ^5^Winship Cancer Institute at Emory University, Atlanta, GA, USA; ^6^Eli Lilly and Company, Bridgewater, NJ, USA; ^7^AstraZeneca Pharmaceuticals, Waltham, MA, USA; ^8^Sarah Cannon Research Institute at HealthONE, Denver, CO, USA

###### **Correspondence:** Todd M. Bauer (xxxx@aol.com)

**Background**

Checkpoint inhibitors of programmed cell death-1 protein (PD-1)/programmed cell death-ligand 1 (PD-L1) and cytotoxic T lymphocyte associated protein 4 (CTLA-4) pathways have demonstrated clinically meaningful improvement in survival for patients with various tumor types. Preclinical data demonstrate significant interplay between innate and adaptive immune systems. Targeting colony-stimulating factor 1 receptor (CSF-1R) may lead to disruption of the immunosuppressive effects of innate immune cells expressing CSF-1R. Treatment with an anti-CSF-1R monoclonal antibody (mAb) induces anti-tumor responses in multiple murine tumor models when combined with CTLA-4 blockade [1], suggesting that combining a checkpoint inhibitor with a CSF-1 pathway inhibitor may potentiate the anti-tumor response. Study I5F-MC-JSCC will evaluate the effects of CSF-1R inhibition using LY3022855 (anti-CSF-1R mAb) in combination with durvalumab (MEDI4736; anti-PD-L1 mAb) or tremelimumab (anti-CTLA-4 mAb) in participants with advanced solid malignancies.

**Methods**

JSCC is a phase Ia/Ib open-label, 3+3 dose-escalation (Part A), followed by dose-expansion (Part B) study of LY3022855 in combination with either durvalumab or tremelimumab. Eligible patients have confirmed solid malignancies (regardless of PD-L1 status) and ECOG PS 0-1. Patients must not have received small molecule therapy, chemotherapy, radiation therapy, monoclonal antibody treatment, or immunosuppressive medication within 14-28 days of start of study treatment, but prior immune checkpoint therapy is permitted. Pretreatment and on-treatment biopsies will be obtained (Part A all patients, Part B ovarian cohort). In the LY3022855+durvalumab regimen, LY3022855 (IV) will be administered at increasing dose levels as tolerated; durvalumab (IV) will be administered at a fixed dose. In the LY3022855+tremelimumab regimen, both LY3022855 and tremelimumab (IV) will be administered at increasing dose levels as tolerated. Once a maximum tolerated dose has been identified for each combination, enrollment to Part B (5 disease-specific expansion cohorts of 20 patients per cohort: LY3022855+durvalumab- non-small cell lung cancer, ovarian, melanoma; LY3022855+tremelimumab- mesothelioma, melanoma) will begin. The primary objective is to characterize the safety and tolerability of each combination in treatment of patients with advanced solid malignancies, as well as define a recommended phase II dose. Secondary objectives include assessment of antitumor activity of each combination, immunogenicity, and pharmacokinetics. Exploratory objectives are to assess immunomodulatory effects of the combinations. Approximately 178 patients are planned.

**Trial Registration**

ClinicalTrials.gov identifier NCT02718911.

**References**

1. Holmgaard RB, Brachfeld A, Gasmi B, *et al*: **Timing of CSF-1/CSF-1R signaling blockade is critical to improving responses to CTLA-4 based immunotherapy.** *OncoImmunology* 2016, **5**:e1151595.

### P138 Phase III study of carboplatin-paclitaxel/nab-paclitaxel chemotherapy with or without pembrolizumab for first-line metastatic, squamous non–small cell lung carcinoma: KEYNOTE-407

#### Edward B. Garon^1^, Balazs Halmos^2^, Hui Rina^3^, Natasha Leighl^4^, Sung Sook Lee^5^, William Walsh^6^, Konstanin Dragnev^7^, Bilal Piperdi^8^, Luis Paz-Ares Rodriguez^9^, Nabeegha Shinwari^8^, Ziewn Wei^8^

##### ^1^David Geffen School of Medicine at UCLA, Santa Monica, CA, USA; ^2^Montefiore Medical Center, White Plains, NY, USA; ^3^The Crown Princess Mary Cancer Centre Westmead, Westmead, New South Wales, Australia; ^4^Princess Margaret Cancer Centre, Toronto, ON, Canada; ^5^Inje University Haeundae Paik Hospital, Busan, Republic of Korea; ^6^University of Massachusetts Medical School, Worchester, MA, USA; ^7^Dartmouth Hitchcock Medical Center, Lebanon, NH, USA; ^8^Merck & Co., Inc., Kenilworth, NJ, USA; ^9^Gabinete Radiologico del Dr. Pita, NA, Madrid, Spain

###### **Correspondence:** Edward B. Garon (egaron@mednet.ucla.edu)

**Background**

Chemotherapy with a platinum doublet has traditionally been the standard of care for most patients with treatment-naive non–small cell lung carcinoma (NSCLC). Preliminary data from the phase I/II KEYNOTE-021 (ClinicalTrials.gov, NCT02039674) study suggest manageable toxicity and encouraging efficacy of carboplatin and paclitaxel chemotherapy plus the anti–PD-1 antibody pembrolizumab in treatment-naive NSCLC. KEYNOTE-407 (ClinicalTrials.gov, NCT02775435) is a randomized, double-blind, placebo-controlled phase III study to compare the efficacy and safety of paclitaxel or nab-paclitaxel plus carboplatin with or without pembrolizumab as first-line treatment in advanced squamous NSCLC.

**Methods**

Eligible patients are aged ≥18 years and have stage IV squamous NSCLC, an ECOG PS 0-1, and no prior systemic chemotherapy. Patients with mixed histology (e.g., adenosquamous) are allowed if there is a squamous component in the specimen; small cell histology is excluded. Approximately 560 patients will be randomly assigned (1:1) to pembrolizumab 200 mg every 3 weeks (Q3W) plus carboplatin area under the curve (AUC) 6 Q3W and paclitaxel 200 mg/m^2^ Q3W or nab-paclitaxel 100 mg/m^2^ day 1, day 8, and day 15 Q3W for 4 cycles followed by pembrolizumab 200 mg Q3W or to the same regimen in which pembrolizumab is replaced by a normal saline placebo. Patients are to be stratified by choice of taxane (paclitaxel vs nab-paclitaxel), PD-L1 status (tumor proportion score [TPS] ≥1% vs < 1%; and geographic region of the enrolling site (East Asia vs non–East Asia) before randomization. Pembrolizumab/placebo will continue for 35 cycles or until disease progression, intolerable toxicity, or investigator or patient decision to withdraw. Patients who received placebo may be able to cross over to pembrolizumab at the time of documented progression. Adverse events (AEs) will be monitored throughout the study and for 30 days (90 days for serious AEs) after treatment completion and graded per NCI CTCAE v4.0. Response will be assessed by RECIST v1.1 by independent central radiologic review at weeks 6, 12, and 18, then every 9 weeks until week 45, and every 12 weeks thereafter. The primary end points are progression-free survival (PFS) by independent radiologic review and overall survival; secondary end points are objective response rate, duration of response, and safety. Exploratory objectives include PFS by PD-L1 TPS status (≥1% vs < 1%) and choice of taxane (paclitaxel or nab-paclitaxel), patient-reported outcomes (EORTC QLQ-C30 and LC13 and EuroQoL-5D), pharmacokinetics, and biomarkers.

**Trial Registration**

ClinicalTrials.gov identifier NCT02775435.

### P139 Preliminary manufacturing, safety, and immune monitoring results of an allogeneic tumor lysate-pulsed dendritic cell vaccine for patients with newly diagnosed glioblastoma

#### Michael P. Gustafson, Mary L Maas, Michael Deeds, Adam Armstrong, Svetlana Bornschlegl, Tim Peterson, Sue Steinmetz, Dennis A Gastineau, Ian F Parney, Allan B Dietz

##### Mayo Clinic, Rochester, MN, USA

###### **Correspondence:** Michael P. Gustafson (gustafson.michael@mayo.edu)

**Background**

Clinical responses to dendritic cell (DC) vaccines for brain tumors have been demonstrably inconsistent. Several factors, including tumor and treatment related immunosuppression, poor DC maturation, and suboptimal antigen sources, likely contribute to this phenomenon. We report preliminary data from a clinical trial for patients with newly diagnosed glioblastoma (GBM) that combines current standard of care treatment with an autologous DC vaccine using mature DCs pulsed with allogeneic cultured GBM lysates.

**Methods**

Twenty adult patients with resected newly diagnosed GBM who had completed radiation/concurrent temozolomide were enrolled. DCs were manufactured *in vitro* from CD14+ monocytes purified from patient leukapheresis products and cultured to produce highly pure CD83+ mature DCs. The mature autologous DCs were pulsed with allogeneic tumor lysates with defined tumor-associated antigen expression. Patients received temozolomide plus intradermal injections of the vaccine (10-20 x10^6^ DCs) for up to 6 cycles followed by vaccine alone for up to 6 cycles. In some cases, patients received injections using a novel intradermal delivery device (3M human microstructured transdermal system).

**Results**

Allogeneic cultured GBM lysate pulsed DCs were manufactured successfully to produce 15 doses of at least 10x10^6^ DCs/injection in 100% (19/19) of patients to date. Vaccines were manufactured consistently as DCs collectively averaged 91.4% CD83+ and 98.1% CD80-positive for these maturation markers. Patients on trial have received a median of 8 injections and three patients have received all 15 doses. Of the toxicities potentially related to the vaccine, only grade 1 and 2 toxicities (fever, rash, fatigue) have been observed. Mean follow-up to date is 0.98 years (range 0.19 – 1.77 years); 15/20 patients are still alive. In the first 8 patients, median survival has not been reached with median follow-up 1.56 years. To assess immune responses to the vaccine, patients were monitored for over 120 immunophenotypes and circulating tumor antigen specific T cells by flow cytometry with up to 8 longitudinal samples for some patients.

**Conclusions**

The combination of adjuvant temozolomide with an autologous mature DC pulsed with allogeneic tumor lysate vaccine was shown to be safe, feasible, and able to produce tumor antigen-specific immune responses in newly diagnosed GBM patients. This successful strategy is well suited for continuing into later stage clinical trials.

**Acknowledgements**

This study is funded in part by the Ben and Catherine Ivy Foundation.

**Trial Registration**

ClinicalTrials.gov identifier NCT01957956.

### P140 AIM2CERV: a randomized phase III study of adjuvant AXAL immunotherapy following chemoradiation in patients who have high-risk locally advanced cervical cancer (HRLACC)

#### Thomas Herzog^1^, Floor J Backes^2^, Larry Copeland^3^, Maria Del Pilar Estevez Diz^4^, Thomas W Hare^5^, Warner Huh^6^, Byoung-Gie Kim^7^, Kathleen M Moore^8^, Ana Oaknin^9^, William Small^10^, Krishnansu S Tewari^11^, Bradley J Monk^12^

##### ^1^University of Cincinnati, Cincinnati, OH, USA; ^2^James Comprehensive Cancer Center, The Ohio State University, Columbus, OH, USA; ^3^James Cancer Hospital, The Ohio State University, Columbus, OH, USA; ^4^Instituto do Câncer do Estado de São Paulo - Faculdade de Medicina da Universidade de São Paulo, São Paulo, Brazil; ^5^Advaxis Immunotherapies, Princeton, NJ, USA; ^6^University of Alabama at Birmingham, Department of Obstetrics & Gynecology, Birmingham, AL, USA; ^7^Samsung Medical Center, Sungkyunkwan University School of Medicine, Seoul, Republic of Korea; ^8^Stephenson Oklahoma Cancer Center, Oklahoma City, OK, USA; ^9^Vall d´Hebron University Hospital, Vall d´Hebron Institute of Oncology (VHIO), Barcelona, Spain; ^10^Loyola University, Maywood, IL, USA; ^11^University of California, Irvine Medical Center, Orange, CA, USA; ^12^University of Arizona Cancer Center-Phoenix Creighton University School of Medicine at Dignity Health St. Joseph’s Hospital and Medical Center, Phoenix, AZ, USA

###### **Correspondence:** Thomas Herzog (thomas.herzog@uc.edu)

**Background**

Patients with HRLACC experience a 50% chance of disease recurrence/death following cisplatin-based chemoradiation (CCRT) plus brachytherapy, and represent a group with a significant unmet need for new treatments. Persistent infection with oncogenic strains of human papillomavirus (HPV) is the most common cause of CC, and provides rationale for therapeutic targeting of HPV. Axalimogene filolisbac (AXAL/ADXS11-001) is an irreversibly attenuated *Listeria monocytogenes*-listeriolysin O immunotherapy that secretes a HPV E7 fusion protein that induces HPV-specific cytotoxic T cell generation and reduces immune tolerance in the tumor microenvironment. Previous studies demonstrated AXAL was well tolerated and associated with objective tumor response and survival benefits in patients with recurrent/metastatic CC. AXAL has received FDA Fast Track Designation for the treatment of HRLACC.

**Methods**

This double-blind, placebo-controlled, multinational, multicenter randomized phase III trial is being conducted under a Special Protocol Assessment agreement with the FDA. The study will evaluate adjuvant AXAL in patients with HRLACC, defined as histologically confirmed squamous cell, adenocarcinoma, or adenosquamous carcinoma of the cervix and ≥1 of the following: 1) FIGO stage IB2, IIA2, IIB with biopsy-proven pelvic nodes, or ≥2 positive nodes by MRI/CT ≥1.5-cm diameter, or ≥2 positive pelvic nodes by PET; 2) all FIGO stage IIIA, IIIB, IVA; 3) any FIGO stage with para-aortic lymph node metastases criteria, defined by biopsy-proven para-aortic node(s), or ≥1 positive para-aortic node(s) by MRI/CT >1.5-cm shortest dimension, or ≥1 positive para-aortic node(s) by PET with SUV >2.5. Eligible patients must be disease free per RECIST 1.1 following completion of CCRT with curative intent and aged ≥18 with GOG performance status 0–1. Patients will be randomized 2:1 to AXAL (1×10^9^ colony-forming units) or placebo and receive a 60-minute infusion of treatment every 3 weeks for 3 doses (weeks 1, 4, and 7) for the first 3 months (Induction Phase). Thereafter, patients will receive treatment every 8 weeks for 5 doses or until disease recurrence (Maintenance Phase); patients will receive a 7-day course of oral antibiotics/placebo 72 hours after completion of each treatment in both phases. Primary objective is to compare disease-free survival (DFS) of AXAL with placebo; secondary objectives are safety and overall survival (OS). Exploratory objectives will determine if there is an association between HPV subtypes and DFS/OS, and patient-reported outcomes. The design provides 85% power for a sample size of 450 to demonstrate a reduction in the hazard of recurrence by 38%.

**Trial Registration**

ClinicalTrials.gov identifier NCT02853604.

### P141 KEYNOTE-057: phase II study of pembrolizumab in patients with Bacillus Calmette Guérin (BCG)–unresponsive, high-risk non–muscle-invasive bladder cancer (NMIBC)

#### Ashish M. Kamat^1^, Joaquim Bellmunt^2^, Toni K Choueiri^3^, Kijoeng Nam^4^, Maria De Santis^5^, Robert Dreicer^6^, Noah M Hahn^7^, Rodolfo Perini^4^, Arlene Siefker-Radtke^1^, Guru Sonpavde^8^, Ronald de Wit^9^, J. Alfred Witjes^10^, Stephen Keefe^4^, Dean Bajorin^11^

##### ^1^University of Texas MD Anderson Cancer Center, Houston, TX, USA; ^2^Dana-Farber Cancer Institute, Harvard Medical School, Boston, MA, USA; ^3^Dana-Farber Cancer Institute/Brigham and Women’s Hospital, Boston, MA, USA; ^4^Merck & Co., Inc., Kenilworth, NJ, USA; ^5^University of Warwick, Coventry, England, UK; ^6^University of Virginia School of Medicine, Charlottesville, VA, USA; ^7^Sidney Kimmel Comprehensive Cancer Center at Johns Hopkins University, Baltimore, MD, USA; ^8^University of Alabama at Birmingham Comprehensive Cancer Center, Birmingham, AL, USA; ^9^Erasmus MC Cancer Institute, Rotterdam, Netherlands; ^10^University Radboud, Nijmegen, Netherlands; ^11^Memorial Sloan Kettering Cancer Center, New York, NY, USA

###### **Correspondence:** Ashish M. Kamat (akamat@mdanderson.org)

**Background**

A large percentage of patients with NMIBC experience disease recurrence/progression after standard-of-care therapy with transurethral resection of bladder tumor (TURBT) and intravesical BCG instillation. The programmed death-1/programmed death ligand 1 (PD-1/PD-L1) pathway is frequently altered in cancer, leading to inhibition of active T cell-mediated immune surveillance of tumors. PD-L1 is widely expressed in urothelial tumors, providing a therapeutic rationale for targeting this pathway in NMIBC. KEYNOTE-057 is an open-label, phase II study designed to evaluate the efficacy and safety of the anti-PD-1 antibody pembrolizumab in patients with high-risk, BCG-unresponsive NMIBC.

**Methods**

Eligibility criteria include age ≥18 years, histologically confirmed diagnosis of high-risk, BCG-unresponsive NMIBC (high-grade Ta, T1, and/or carcinoma *in situ* [CIS] despite adequate BCG treatment), ineligible for or declined radical cystectomy, and ECOG performance status 0-2. Eligible patients must have undergone ≥2 cystoscopic procedures with the most recent ≤8 weeks before study start, including complete TURBT (tissue sample must be available). Patients will receive pembrolizumab 200 mg every 3 weeks for 24 months or until disease recurrence, progression, or unacceptable toxicity. Patients will be placed into cohorts based on presence (cohort A) or absence (cohort B) of CIS as determined by tissue pathology at screening. Response will be assessed using cystoscopy and urine cytology every 12 weeks for the first 2 years, every 24 weeks for the next 2 years, and every 52 weeks thereafter. CT imaging will be used to assess for metastatic or nodal disease. At 18 months, patients with no evidence of disease may discontinue treatment. Low-grade Ta recurrence will not be considered treatment failure; these patients may undergo repeat TURBT and remain on treatment. Adverse events (AEs) will be monitored throughout the study and for 30 days after end of treatment (90 days for serious AEs and events of clinical interest) and graded per Common Terminology Criteria for Adverse Events, version 4.0. Primary end points are complete response (cohort A) and disease-free survival (cohort B); secondary end points include progression-free survival, overall survival, duration of response, and the relationship between PD-L1 expression and response to treatment. Enrollment will continue until approximately 260 patients have enrolled.

**Trial Registration**

ClinicalTrials.gov identifier NCT02625961.

### P142 KEYNOTE-013: an open-label, multicohort phase Ib trial of pembrolizumab in patients with advanced hematologic malignancies

#### Justin Kline^1^, Philippe Armand^2^, John Kuruvilla^3^, Craig Moskowitz^4^, Mehdi Hamadani^5^, Vincent Ribrag^6^, Pier Luigi Zinzani^7^, Sabine Chlosta^8^, Seth Thompson^8^, Arun Balakumaran^8^, Nancy Bartlett^9^

##### ^1^Committee on Immunology and Department of Medicine, University of Chicago, Chicago, IL, USA; ^2^Dana-Farber Cancer Institute, Boston, MA, USA; ^3^Princess Margaret Cancer Centre and University of Toronto, Toronto, ON, Canada; ^4^Memorial Sloan Kettering Cancer Center, New York, NY, USA; ^5^Medical College of Wisconsin, Milwaukee, WI, USA; ^6^Institut Gustave Roussy, Villejuif, Ile-de-France, France; ^7^Institute of Hematology “L. e A. Seràgnoli,” Università di Bologna, Bologna, Emilia-Romagna, Italy; ^8^Merck & Co., Inc., Kenilworth, NJ, USA; ^9^Washington University School of Medicine in St. Louis, St Louis, MO, USA

###### **Correspondence:** Justin Kline (jkline@medicine.bsd.uchicago.edu)

**Background**

Pembrolizumab, a humanized monoclonal antibody that blocks interaction between PD-1 and its ligands, has demonstrated robust antitumor activity and a manageable toxicity profile in advanced solid tumors. KEYNOTE-013 is a multicenter, open-label, multicohort phase Ib trial designed to assess the safety and efficacy of single-agent pembrolizumab in patients with hematologic malignancies. The KEYNOTE-013 trial design has been updated to include a cohort evaluating combination therapy of pembrolizumab and lenalidomide in patients with relapsed/refractory (R/R) diffuse large B cell lymphoma (DLBCL) who have failed, are ineligible for, or refused stem cell transplantation (SCT). Combination therapy of pembrolizumab, lenalidomide, and dexamethasone in R/R multiple myeloma (MM) in KEYNOTE-023 has demonstrated synergistic efficacy and manageable toxicity [1].

**Methods**

Cohorts include patients with: (1) intermediate 1, intermediate 2, or high-risk myelodysplastic syndrome (MDS) that failed ≥4 cycles of prior treatment with a hypomethylating agent; (2) R/R MM that failed ≥2 lines of prior therapy, including a proteasome inhibitor and IMiD; (3) R/R nodular sclerosing or mixed cellularity Hodgkin lymphoma (HL) (cohort 3); (4) R/R non-Hodgkin lymphoma (NHL) who failed, were ineligible for, or refused SCT, including: (4a) R/R primary mediastinal large B cell lymphoma (PMBCL), (4b) any other R/R PD-L1–positive NHL, (4c) R/R follicular lymphoma (FL), (4d) R/R DLBCL; and (5) R/R DLBCL. Key eligibility criteria for all cohorts are age ≥18 years, ECOG performance status 0/1, measurable disease, and adequate hematologic, renal, and hepatic function. Patients in cohorts 1-4 enrolled under amendments 1-3 will be treated with pembrolizumab intravenously (IV) 10 mg/kg every 2 weeks; those enrolled under amendments 4-6 will be treated with pembrolizumab IV 200 mg every 3 weeks (Q3W) because of updated program-wide PK/PD information. Patients enrolled in cohort 5 will be treated with pembrolizumab IV 200 mg Q3W in combination with lenalidomide taken orally once daily for 21 days of each 28-day cycle. Treatment will continue until disease progression, intolerable toxicity, or up to 35 doses of pembrolizumab (~2 years). The primary end points are objective response rate and safety. Secondary objectives include duration of response, progression-free survival, overall survival, and association between PD-L1 expression and response. Enrollment is open for MM (cohort 2), PMBCL (cohort 4a), and FL (cohort 4c), evaluating single-agent pembrolizumab; and for DLBCL (cohort 5), evaluating pembrolizumab in combination with lenalidomide. Approximately 222 patients will be enrolled.

**Trial Registration**

ClinicalTrials.gov identifier NCT01953692.

**References**

1. Mateos MV, *et al*: *J Clin Oncol* 2016, **34[suppl]**:Abstr 8010.

### P143 Trials in progress: a phase II study of in situ therapeutic vaccination against refractory solid cancers with intratumoral poly-ICLC

#### Chrisann Kyi^1^, Rachel Sabado^1^, Yvonne Saenger^2^, Loging William^3^, Michael Joseph Donovan^3^, Erlinda Sacris^1^, John Mandeli^3^, Andres M. Salazar^4^, Philip Friedlander^1^, Nina Bhardwaj^1^

##### ^1^Tish Cancer Institute, Icahn School of Medicine at Mount Sinai, New York, NY, USA; ^2^Hematology and Oncology Division, Columbia University Medical Center, New York, NY, USA; ^3^Icahn School of Medicine at Mount Sinai, New York, NY, USA; ^4^Oncovir, Inc., Washington, DC, USA

###### **Correspondence:** Chrisann Kyi (chrisann.kyi@gmail.com)

**Background**

Poly-ICLC, a double-stranded RNA complex, can directly activate dendritic cells and trigger NK cells to kill tumor cells. It can be given intramuscularly (IM) to induce systemic inflammation and intratumorally (IT) to induce immune infiltration of tumors.

**Methods**

In this phase II study, eligible subjects are head and neck, skin (melanoma and non-melanoma), and sarcoma patients with recurrent or metastatic disease who have failed prior systemic therapy. In each treatment cycle, one accessible tumor site was targeted for IT injection of 1 mg of Poly-ICLC 3 times a week for 2 weeks followed by IM boosters biweekly for 6 weeks and then a 2 week rest period. This 10-week cycle was repeated in cycle 2, followed by a 6 week no-treatment period. Tumor biopsies were performed at baseline, week 3, and week 26. Pre- and post-vaccination tumors were evaluated by quantitative multiplex immunohistochemistry (IHC) and RNA sequencing. Blood samples were collected at baseline and prior to each cycle for immune response evaluations.

**Results**

A phase I pilot portion of this study showed 1 patient who achieved clinical benefit with stable disease (progression-free survival of 6 months). Poly-ICLC was well tolerated with principal side effects of fatigue and inflammation at injection site (< grade 2). One case of grade 3 tumor necrosis was observed. In the patient with clinical benefit, IHC analysis of tumor showed increased CD4(60x), CD8(10x), PD-1(20x) and PD-L1(3x) compared to patients with progressive disease whose tumor biopsies showed unchanged/decreased CD4, CD8, PD-1, and PD-L1 levels over treatment period. Furthermore, RNASeq analysis of the same patient’s tumor and peripheral blood mononuclear cells (PBMC) showed dramatic changes such as upregulation of interferon-stimulated genes, chemokines, and genes associated with T cell activation and antigen presentation indicative of local and systemic immune activation in response to Poly-ICLC treatment.

**Conclusions**

Preliminary findings show that Poly-ICLC is well tolerated in advanced solid cancer patients, and generates local immune response in tumor microenvironment and systemic immune response as evident in the patient achieving clinical benefit. These results warrant further investigation, and are currently being explored in this ongoing larger multicenter adaptive phase II clinical trial.

**Trial Registration**

ClinicalTrials.gov identifier NCT01984892.

**References**

1. Martins KA, Bavari S, *et al*: **Vaccine adjuvant uses of poly-IC and derivatives.** *Expert Rev Vaccines* 2015, **14**:447-459.

2. Salazar A, Erlich R, *et al*: **Therapeutic in situ autovaccination against solid cancers with intratumoral poly-iclc: case report, hypothesis, and clinical trial.** *Cancer Immunol Res* 2014, **2(8)**:720-724.

### P144 Phase I/Ib multicenter trial of CPI-444, an adenosine A2a receptor (A2aR) antagonist as a single agent and in combination with atezolizumab (atezo) in patients with advanced solid tumors

#### John Powderly^1^, Joshua Brody^2^, John Nemunaitis^3^, Leisha Emens^4^, Jason J Luke^5^, Amita Patnaik^6^, Ian McCaffery^7^, Richard Miller^7^, Ginna Laport^7^

##### ^1^Carolina BioOncology Institute, PLLC, Huntersville, NC, USA; ^2^Icahn School of Medicine at Mount Sinai, New York, NY, USA; ^3^Mary Crowley Cancer Research Centers, Texas Oncology, P.A., Gradalis, Inc., Medical City Dallas Hospital, Baylor University Medical Center, Dallas, TX, USA; ^4^Johns Hopkins University School of Medicine, Baltimore, MD, USA; ^5^University of Chicago School of Medicine, Chicago, IL, USA; ^6^South Texas Accelerated Research Therapeutics, LLC, San Antonio, TX, USA; ^7^Corvus Pharmaceuticals, Burlingame, CA, USA

###### **Correspondence:** Ginna Laport (glaport@corvuspharma.com)

**Background**

Adenosine is elevated within the tumor microenvironment and signaling through the A2aR is immunosuppressive. CPI-444 is an oral, selective A2aR antagonist that inhibits A2aR and demonstrates antitumor efficacy in mouse models alone and combined with PD-1/PD-L1 blockade. CPI-444 was well-tolerated in previous clinical trials in the non-oncology setting. This is the first report of adenosine blockade in cancer patients (pts).

**Methods**

This phase I/Ib, open label clinical trial uses a two-step adaptive design to study CPI-444 alone and combined with atezo in pts with selected advanced cancers. The objectives are to evaluate safety and efficacy and to optimize dose/schedule. Eligible pts have failed standard therapies with histologies: non-small cell lung (NSCLC), melanoma (MEL), triple-negative breast (TNBC), renal (RCC), prostate, head and neck (H&N), colorectal (CRC) or bladder cancers. In step 1 of the trial, pts are randomized to one of 4 cohorts (12 pts/cohort) including 3 single-agent cohorts or combined with atezo. Step 2 contains multiple disease-specific expansion cohorts to evaluate CPI-444 alone and combined with atezo.

**Results**

Step 1 has enrolled 19 pts to date, median age 67 years (range, 36-84); three pts each with NSCLC, TNBC, RCC and CRC, 2 pts each with MEL, prostate and bladder cancer and one pt with H&N. Median number of prior regimens was 3 (range, 1-5). Seven pts received prior anti PD-1/PD-L1 therapy; 4 pts were resistant and 3 were refractory. Twelve pts remain on study (range 1- 17 weeks) and 7 have discontinued due to disease progression. Of 8 pts evaluable for response at 2 months, 3 had stable disease (2 received CPI-444 alone); 11 pts have not reached the first response assessment timepoint. A prostate cancer pt in the combination cohort remains on treatment for >4 months with minimal regression of nodal disease and a transient decrease in serum PSA. A TNBC pt on CPI-444 alone showed 15% reduction in tumor burden. No grade 3 or 4 adverse events or DLTs related to CPI-444 alone or in combination with atezo were observed. Dose-dependent inhibition of A2aR signaling was demonstrated in peripheral blood mononuclear cells using a CREB phosphorylation assay. Evidence of immune activation was observed in some pts treated with CPI-444 alone as reflected by an increase in circulating activated PD-1+/CD8+ T cells.

**Conclusions**

Early data demonstrate that CPI-444 is well-tolerated alone and combined with atezo and demonstrates preliminary evidence of immune activation and clinical activity. Enrollment is ongoing.

**Trial Registration**

ClinicalTrials.gov identifier NCT02655822.

### P145 SEA-CD40 is a CD40 agonist with early evidence of pharmacodynamic and antitumor activity: preliminary results from a phase I study in advanced solid malignancies

#### Andrew L Coveler^1^, David C Smith^2^, Juneko E Grilley-Olson^3^, Thomas F Gajewski^4^, Sanjay Goel^5^, Shyra J Gardai^6^, Che-Leung Law^6^, Gary Means^6^, Thomas Manley^6^, Brendan Curti^7^

##### ^1^Seattle Cancer Care Alliance, University of Washington, Seattle, WA, USA; ^2^Univerisy of Michigan, Ann Arbor, MI, USA; ^3^University of North Carolina Lineberger Comprehensive Cancer Center, University of North Carolina Chapel Hill, Chapel Hill, NC, USA; ^4^University of Chicago Medical Center, Chicago, IL, USA; ^5^Montefiore Medical Center, Bronx, NY, USA; ^6^Seattle Genetics, Inc., Bothell, WA, USA; ^7^Earle A. Chiles Research Institute, Providence Cancer Center, Portland, OR, USA

###### **Correspondence:** Thomas Manley (tmanley@seagen.com)

**Background**

SEA-CD40 is a non-fucosylated, humanized IgG1 monoclonal antibody which binds CD40, an immune-activating TNF receptor. Binding to CD40 on antigen-presenting cells (APCs) and enhanced crosslinking via FcγRIIIa stimulates pro-inflammatory cytokine production and induction of immune co-stimulatory receptors, leading to T cell activation and antitumor activity. Further, when CD40 is expressed on malignant cells, SEA-CD40 induces antibody-dependent cellular cytotoxicity through enhanced NK cell binding.

**Methods**

This ongoing phase I dose-escalation study evaluates the safety, tolerability, pharmacodynamic biomarkers, and antitumor activity of SEA-CD40 in adult patients with advanced metastatic solid tumors (relapsed, refractory, or progressive disease after ≥1 prior systemic therapy). Antitumor activity is assessed after every 4 cycles of treatment by immune-related response criteria and RECIST v1.1.

**Results**

To date, 22 patients (median age 59 years; range, 28–76) with solid tumors have received a median of 2.5 cycles (range, 1–16) of SEA-CD40, 0.6–60 mcg/kg IV q3wk. Two dose-limiting toxicities occurred at 60 mcg/kg (1 G3 and 1 G4 infusion-related reaction [IRR]). AEs were primarily infusion-related toxicities. Treatment-emergent AEs in ≥25% of patients were: chills (77%), nausea (55%), fatigue (45%), vomiting (41%), dyspnea and headache (32% each), and flushing and lymphopenia (27% each). Changes in pharmacodynamic biomarkers consistent with CD40 and CD16 engagement were observed, including: dose-proportional increases in inflammatory cytokine levels; B cell depletion with partial recovery by next cycle; reduction in T regulatory cells; transient margination of monocytes and NK cells with recovery by Day 8; and upregulation of MHC class II on APCs. In 18 efficacy-evaluable patients, 4 had SD and 1 had PR by RECIST (28% disease control rate [DCR]). Two patients had durable SD (gastroesophageal junction tumor, 10 mcg/kg, PD after 12 cycles; mesothelioma, 10 mcg/kg, PD after 8 cycles). The patient with PR achieved PR after Cycle 4 (basal cell carcinoma, 60 mcg/kg, PD following Cycle 8). Four patients remain on treatment.

**Conclusions**

SEA-CD40 is a biologically and clinically potent molecule in heavily pre-treated patients with advanced solid tumors. Strategies to manage IRRs are being evaluated. Cytokine elevations correlate with preclinical models and the proposed mechanism of action through CD40 activation on APCs. Pharmacodynamic data and evidence of clinical activity (28% DCR), along with preclinical evidence for synergy when SEA-CD40 is combined with checkpoint inhibitors [1], presents a compelling opportunity to enhance immunotherapy for cancer.

**Trial Registration**

ClinicalTrials.gov identifier NCT02376699.

**References**

1. Gardai SJ, *et al*: **A sugar-engineered non-fucosylated anti-CD40 antibody, SEA-CD40, with enhanced immune-stimulatory activity alone and in combination with immune checkpoint inhibitors.** *J Clin Oncol* 2015, **33(15Suppl)**:3074.

### P146 A randomized phase II study of epigenetic therapy with azacitidine and entinostat with concurrent nivolumab versus nivolumab alone in recurrent metastatic non-small cell lung cancer

#### Kristen A. Marrone^1^, Gary Rosner^2^, Valsamo Anagnostou^1^, Joanne Riemer^1^, Jessica Wakefield^1^, Cynthia Zanhow^1^, Stephen Baylin^1^, Barbara Gitlitz^3^, Julie Brahmer^1^

##### ^1^Sidney Kimmel Comprehensive Cancer Center at Johns Hopkins University, Baltimore, MD, USA; ^2^School of Medicine Oncology Biostatistics, Baltimore, MD, USA; ^3^Keck School of Medicine of USC, Los Angeles, CA, USA

###### **Correspondence:** Kristen A. Marrone (kmarron1@jhmi.edu)

**Background**

While survival results with immune checkpoint blockade in the second-line treatment setting of non-small cell lung cancer (NSCLC) are impressive, many patients’ cancer progresses or becomes resistant to these therapies. Combinatorial approaches are being investigated to improve response and outcomes with these agents. Epigenetic therapy has been found to be effective component of cancer treatment. Hypermethylation of DNA promoter regions by DNA methyltransferases (DNMT) and histone deacetylation by histone deacetylases (HDAC) represent two of these critical epigenetic mechanisms of tumor-specific gene silencing. Interestingly, 5 of 5 patients treated with combination epigenetic therapy followed by PD-1/PD-L1 therapy achieved long-term clinical benefit at our institution while on clinical trial. Preclinical studies have also shown that combined HDAC/DNMT inhibition can induce susceptibility to immune checkpoint therapy and inhibit tumor growth [1].

**Methods**

**Fig. 60:**
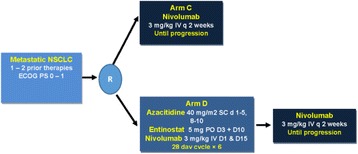
**(Abstract P146).** Study Schema

This is a multi-institution, open-label, randomized phase II study of azacitidine and entinostat with concurrent nivolumab compared to nivolumab alone in patients with recurrent, metastatic NSCLC. Patients with metastatic NSCLC who have received 1-2 prior therapies and are Eastern Cooperative Group (ECOG) performance status (PS) 0-1 are stratified by histology and randomized to receive azacitidine (40 mg/m2 subcutaneous (SQ) days 1-5, 8-10), entinostat (4 mg orally days 3 and 10) and nivolumab (3 mg/kg intravenous (IV) days 1 and 15) for 6 28-day cycles, followed by nivolumab (3 mg/kg IV days 1 and 15) alone [Arm D] or nivolumab (3 mg/kg IV days 1 and 15) on a 28-day cycle alone [Arm C] until disease progression (Fig. 60). Statistics: The primary endpoint of this trial is the percentage of patients progression-free at 24 weeks. Using planned study enrollment of 60 patients on each Arm (n=120) and using the exact Mantel-Haenszel test for analysis, this will provide 90% power to detect an odds ratio of 3 for combination therapy over single-agent nivolumab with respect to being progression-free at 24 weeks. Secondary endpoints include objective response rate, progression-free survival, time to progression, overall survival and safety and tolerability. Correlative Study: All patients are required to have pre- and post- treatment biopsies; Arm D patients will also have biopsies while on combination therapy. Plasma and peripheral blood mononuclear cells will also be drawn for analysis.

**Trial Registration**

ClinicalTrials.gov identifier NCT01928576.

**References**

1. Cameron EE, Bachman KE, Myohanen S: **Synergy of demethylation and histone deacetylase inhibition in the re-expression of genes silenced in cancer**. *Nat Genet* 1999, **21**:103-107.

### P147 KEYNOTE-427: phase II study of pembrolizumab in patients with locally advanced/metastatic renal cell carcinoma (mRCC)

#### David F. McDermott^1^, Sabina Signoretti^1^, Wenting Li^2^, Charles Schloss^2^

##### ^1^Beth Israel Deaconess Medical Center, Boston, MA, USA; ^2^Merck & Co., Inc., Kenilworth, NJ, USA

###### **Correspondence:** David F. McDermott (dmcdermo@bidmc.harvard.edu)

**Background**

Targeting the programmed death 1 (PD-1) pathway has been found to be effective in patients with previously treated, advanced or clear cell mRCC. Pembrolizumab is an anti–PD-1 antibody that blocks the interaction between PD-1 and its ligands, enabling an increased antitumor immune response. Most patients with mRCC progress within 1 year following the initiation of standard first-line treatment with antiangiogenic therapy, highlighting the need for therapies that provide more durable benefit in mRCC.

**Methods**

KEYNOTE-427 (ClinicalTrials.gov, NCT02853344) is a phase II, open-label study designed to evaluate the efficacy and safety of pembrolizumab monotherapy as a first-line treatment for advanced, recurrent or metastatic clear cell or non–clear cell mRCC. Target enrollment in KEYNOTE-427 is 105 patients in a clear cell cohort (cohort A) and 150 patients in a non–clear cell cohort (cohort B). Key eligibility criteria include locally advanced, metastatic or recurrent disease, measurable disease per RECIST v1.1 assessed by blinded independent central review (BICR), no prior systemic therapy for advanced RCC, provision of a tumor tissue sample for biomarker analysis, and Karnofsky performance status ≥70%. Patients are to receive pembrolizumab intravenously 200 mg once every 3 weeks until progressive disease, the occurrence of unacceptable adverse events (AEs), or for up to 35 doses in patients without progressive disease. Patients who stop pembrolizumab after 35 doses without progressive disease or who stop treatment after a complete response will be allowed treatment with an additional 17 doses of pembrolizumab upon progression. Response will be assessed per RECIST v1.1 by BICR using CT and/or MRI. Bone scans will be performed at baseline and throughout the study for patients with a positive bone scan at baseline. AEs will be graded per National Cancer Institute Common Terminology Criteria for Adverse Events, version 4.0. The primary objective is estimation of the objective response rate in each cohort per RECIST v1.1. An interim analysis for response rate will be performed for the non–clear cell RCC cohort when the 30th patient in this cohort has completed the third scan. Secondary objectives include estimation of duration of response, disease control rate, progression-free/overall survival, and safety and tolerability of pembrolizumab treatment by cohort.

**Trial Registration**

ClinicalTrials.gov identifier NCT02853344.

### P148 KEYNOTE-170: phase II study of pembrolizumab in patients with relapsed/refractory primary mediastinal large B cell lymphoma (rrPMBCL) or relapsed or refractory Richter syndrome (rrRS)

#### Jean-Marie Michot^1^, Philippe Armand^2^, Wei Ding^3^, Vincent Ribrag^1^, Beth Christian^4^, Arun Balakumaran^5^, Patricia Marinello^5^, Sabine Chlosta^5^, Yayan Zhang^5^, Margaret Shipp^2^, Pier Luigi Zinzani^6^

##### ^1^Institut Gustave Roussy, Villejuif, Ile-de-France, France; ^2^Dana-Farber Cancer Institute, Boston, MA, USA; ^3^Mayo Clinic, Rochester, MN, USA; ^4^The Ohio State University, Columbus, OH, USA; ^5^Merck & Co., Inc., Kenilworth, NJ, USA; ^6^Institute of Hematology “L. e A. Seràgnoli,” Università di Bologna, Bologna, Emilia-Romagna, Italy

###### **Correspondence:** Jean-Marie Michot (jean-marie.michot@gustaveroussy.fr)

**Background**

The 9p24.1 locus is frequently amplified in rrPMBCL, leading to overexpression of the PD-L1 and PD-L2 immune checkpoint ligands and providing a potential mechanism of immune evasion. Pembrolizumab is an anti–PD-1 monoclonal antibody that blocks the interaction between PD-1 and PD-L1 and PD-L2, thereby enabling an antitumor immune response. In the multicohort, phase Ib KEYNOTE-013 study, pembrolizumab was associated with a tolerable safety profile and promising antitumor activity (overall response rate [ORR] of 38% 6/16]) in patients with rrPMBCL. In the phase II MC1485 study, pembrolizumab demonstrated promising preliminary efficacy (ORR, 43% [3/7]) in patients with rrRS. The multicenter, phase II KEYNOTE-170 study was designed to further evaluate the safety and efficacy of pembrolizumab in patients with rrPMBCL or rrRS.

**Methods**

Eligible patients must be at least 18 years of age and fit into 1 or 2 profiles: (1) diagnosis of rrPMBCL according to World Health Organization 2008 criteria, failed to achieve a complete response (CR) or relapsed after autologous stem cell transplantation (auto-SCT), or are ineligible for auto-SCT and have failed to respond or relapsed after ≥2 lines of prior treatment; or (2) pathologic diagnosis per local institutional review of rrRS that transformed from underlying chronic lymphocytic leukemia (CLL), received at ≥1 prior therapy for rrRS, and have relapsed or refractory disease. Patients are to receive pembrolizumab 200 mg intravenously every 3 weeks for until disease progression, unacceptable toxicity, or 35 cycles of treatment. In patients with rrRS, additional standard therapies to treat the underlying CLL may be added according to the physician’s discretion. Eligible patients who attain a CR as determined by the independent central imaging vendor may stop trial treatment and may be eligible for retreatment upon disease progression. Response is to be assessed every 12 weeks by independent central imaging vendor review based on International Working Group (IWG) response criteria. Adverse events (AEs) are to be assessed throughout treatment and for 30 days thereafter (90 days for serious AEs) and graded per NCI CTCAE v4.0. Patients will be followed for survival every 12 weeks. The primary end point is ORR by independent central imaging vendor review according to IWG response criteria or IWG response criteria with special considerations for rrRS; secondary end points include progression-free survival, overall survival, and safety and tolerability. Enrollment is ongoing and will continue until approximately 106 patients are enrolled.

**Trial Registration**

ClinicalTrials.gov identifier NCT02576990.

### P149 Dose-seeking and efficacy study of anti-PD-1 therapy with pembrolizumab (P) combined with BRAF inhibitor (BRAFi) therapy with vemurafenib (V) for therapy of advanced melanoma

#### Yana G. Najjar^1^, Lin^1^, Lisa H. Butterfield^1^, Ahmad A. Tarhini^1^, Diwakar Davar^1^, Hassane Zarour^1^, Elizabeth Rush^1^, Cindy Sander^1^, John M Kirkwood^2^

##### ^1^University of Pittsburgh Cancer Institute, Pittsburgh, PA, USA; ^2^University of Pittsburgh Cancer Institute, UPMC Cancer Center, Pittsburgh, PA, USA

###### **Correspondence:** Yana G. Najjar (najjaryg@upmc.edu)

**Background**

BRAF inhibitors induce antitumor responses in >50% of patients (pts) with BRAF V600E/K mutant metastatic melanoma, but some patients do not respond and most responders have only partial responses, the median duration of which is 7-9 months. BRAFi markedly increase tumor infiltrating lymphocytes (TIL), although markers of T cell exhaustion and PD-L1 are increased. We hypothesize that BRAFi will drive TIL into the tumor parenchyma, and that by blocking PD-1/PD-L1 disinhibitory signals, pembrolizumab may improve the function and durability of TILs.

**Methods**

This study is an investigator-initiated phase I/II dose-seeking and efficacy trial of P and V for pts with unresectable/metastatic BRAF mutant melanoma. Primary objectives are to determine safety and maximum tolerated dose (MTD) of V combined with P in this population, and to assess ORR with the combination, in comparison to historical benchmarks. Secondary objectives are to evaluate PFS and OS. Exploratory objectives are to assess whether the combination favorably modulates the tumor microenvironment and decreases T cell exhaustion in sequential biopsies of tumor and blood samples. We aim to accrue up to 50 patients to this study. Using the modified toxicity probability interval (mTPI) will allow efficient identification of the MTD; we expect at least 30 patients to be enrolled at the recommended phase II dose. Pts will receive P 200 mg q3 wks, and V 480 mg, 720 mg, or 960 mg BID, per mTPI. Treatment with both V & P will start on day 1. The DLT monitoring period is 3 wks. Pts will have CT scans at baseline and wk 9, then q12 wks. For pts with biopsiable disease, biopsies at baseline and wk 3 are mandatory. Blood for correlative studies will be drawn at baseline, wk 3, wk 9 and progression. Pts will be treated until DLT or PD for up to 2 years.

**Results**

One patient has been started on therapy and is in wk 2 of the 3 wk DLT monitoring period, with no significant toxicities thus far.

**Conclusions**

We anticipate that treatment with pembrolizumab and vemurafenib will be safe and well tolerated. In addition to the primary efficacy endpoint of ORR and secondary endpoints of PFS and OS, this study includes extensive immune correlative analyses, including analysis of PD-1/PD-L1 and levels of Treg, MDSC, and inhibitory cytokines in the tumor parenchyma and peripheral blood. One patient on trial thus far is tolerating treatment well, with no significant toxicities to date.

**Trial Registration**

ClinicalTrials.gov identifier NCT02818023.

### P150 CX-1158-101: a first-in-human phase I study of a small molecule inhibitor of arginase (CB-1158) as monotherapy and in combination with an anti-PD-1 checkpoint inhibitor in patients with solid tumors

#### Siqing Fu^1^, Todd Bauer^2^, Chris Molineaux^3^, Mark K Bennett^3^, Keith W. Orford^3^, Kyriakos P Papadopoulos^4^

##### ^1^Department of Investigational Cancer Therapeutics, The University of Texas MD Anderson Cancer Center, Houston, TX, USA; ^2^Sarah Cannon Research Institute, Nashville, TN, USA; ^3^Calithera Biosciences, South San Francisco, CA, USA; ^4^South Texas Accelerated Research Therapeutics, San Antonio, TX, USA

###### **Correspondence:** Keith W. Orford (korford@calithera.com)

**Background**

T cells and natural killer (NK) cells require L-arginine for proliferation and activation. Arginine depletion by arginase in the tumor microenvironment induces immunosuppression and is associated with tumor immune evasion, advanced disease stage and poor outcomes. Arginase is secreted by granulocytic myeloid-derived suppressor cells (G-MDSCs) and its pharmacological inhibition is expected to restore arginine levels and relieve immunosuppression. CB-1158 is a potent, selective, and orally-bioavailable small molecule inhibitor of arginase (IC_50_=98 nM). CB-1158 reverses G-MDSC-mediated immunosuppression by blocking arginine depletion in an *ex vivo* human model. T cells activated in the presence of G-MDSC-conditioned media demonstrate reduced proliferation and suppressed production of Th1 cytokines; this effect is reversed by the addition of CB-1158. *In vivo*, twice daily dosing of CB-1158 causes dose-dependent increases in plasma and tumor arginine levels and is associated with single agent anti-tumor efficacy in multiple syngeneic models. CB-1158 also enhances the antitumor efficacy of checkpoint inhibitors.

**Methods**

CX-1158-101 is a phase I first-in-human study of CB-1158 in patients with solid tumors. The primary objective is to evaluate the safety and tolerability of CB-1158, as monotherapy and in combination with nivolumab. Secondary objectives include selection of the recommended phase II dose (RP2D), determination of CB-1158 pharmacokinetics and evaluation the anti-tumor effect, for monotherapy and nivolumab combination. Exploratory objectives include an evaluation of pharmacodynamic biomarkers, including plasma arginine, plasma arginase activity, and effects on immune function in the peripheral blood and tumor biopsies. In Part 1a, safety/tolerability of escalating doses of CB-1158 will be evaluated in patients with solid tumors of any type. In Part 2, three expansion cohorts will open at the monotherapy RP2D. These cohorts include non-small cell lung cancer (NSCLC, Cohort A), colorectal cancer (CRC, Cohort B), and Cohort C, which will include patients with squamous cell carcinoma of the head and neck (SCCHN), renal cell cancer (RCC), gastric or gastroesophageal junction (GEJ) cancer, urothelial cell cancer (UCC), and/or melanoma. Upon demonstration of sufficient PK and/or PD effect of CB-1158 in Part 1a, dose escalation of CB-1158 in combination with full dose nivolumab will open in Part 1b. The dose escalation will enroll patients with NSCLC, RCC, and melanoma. In Part 3, two expansion cohorts will enroll patients to the combination of CB-1158 and nivolumab at the RP2D. These cohorts will enroll NSCLC and melanoma patients that have received prior anti-PD-1/PD-L1 therapy and had progressive disease or prolonged (>24 weeks) stable disease.

### P151 A first-in-human, first-in-class phase I trial of the anti-CD47 antibody Hu5F9-G4 in patients with advanced cancers

#### Sukhmani K. Padda^1^, Sumit A Shah^1^, A Dimitrios Colevas^1^, Sujata Narayanan^1^, George A Fisher^1^, Dana Supan^1^, Heather A Wakelee^1^, Rhonda Aoki^1^, Mark D Pegram^1^, Victor M Villalobos^2^, Jie Liu^3^, Chris H Takimoto^4^, Mark Chao^4^, Jens-Peter Volkmer^5^, Ravindra Majeti^6^, Irving L Weissman^3^, Branimir I Sikic^1^

##### ^1^Stanford University School of Medicine, Stanford, CA, USA; ^2^University of Colorado Anschutz Medical Campus, Aurora, CO, USA; ^3^Institute for Stem Cell Biology and Regenerative Medicine and Forty Seven, Inc., Palo Alto, CA, USA; ^4^Institute for Stem Cell Biology and Regenerative Medicine, Forty Seven, Inc., Stanford University School of Medicine, Palo Alto, CA, USA; ^5^Forty Seven, Inc., Menlo Park, CA, USA; ^6^Institute for Stem Cell Biology and Regenerative Medicine; Stanford University School of Medicine, Stanford, CA, USA

###### **Correspondence:** Sukhmani K. Padda (padda@stanford.edu)

**Background**

Hu5F9-G4 is a humanized monoclonal antibody that targets CD47, blocking its anti-phagocytic “don’t eat me” signal through macrophage receptor SIRPα, leading to tumor phagocytosis. CD47 is overexpressed on human cancers and also on red blood cells (RBCs). In primate toxicology studies, Hu5F9-G4 caused a transient anemia that was improved with a single lower priming dose allowing higher maintenance doses.

**Methods**

Relapsed/refractory solid tumors and lymphomas were included. This dose escalation study included: Part A, to determine the priming dose and Part B, to determine the maintenance dose. The maximum tolerated dose (MTD) in part A was used for the single priming dose in part B (Hu5F9-G4 dosed weekly). The primary objective is to determine safety and secondary objectives are to determine PK and PD. Preliminary data reported from data cutoff of July 22, 2016.

**Results**

21 patients have enrolled. Part A included 0.1 (N=1), 0.3 (N=2), 1 (N=6), and 3 mg/kg (N=2). There were 2 dose-limiting toxicities (DLTs) in Part A at the 3 mg/kg dose: grade (G) 3 abdominal pain and G3 hemagglutination (H) (protocol-specific scale of 1+ H on peripheral blood smear (PBS) and G2 headache). 1 mg/kg was selected as the priming dose, with no >G2 anemia and almost 100% RBC receptor occupancy. Treatment-related adverse events (TRAE) in Part A included: anemia (3 G1, 3 G2), hyperbilirubinemia (3 G1, 2 G2; unconjugated), headache (6 G1, 1 G2), H on PBS (8 G1), and nausea (3 G1). Part B included 3 (N=4), 10 (N=3), and 20 mg/kg (N=3, ongoing). There have been no DLTs in 3 patients on 10 mg/kg (last fully evaluable cohort). Most toxicity was associated with the single priming dose and reversible. TRAE in Part B at 3 mg/kg included: anemia (2 G1, 2 G2), hyperbilirubinemia (1 G1, 1 G3; unconjugated), headache (3 G1), H on PBS (1 G1), retinal toxicity (G2 protocol-specific scale, asymptomatic). TRAE at 10 mg/kg included: anemia (3 G1), headache (2 G1), and nausea (1 G1). Target trough levels associated with preclinical activity are being achieved at the 10 mg/kg dose, and half-life increases with repeated dosing. Two patients with adenoid cystic carcinoma in Part A had stable disease for 16 and 8 months.

**Conclusions**

Hu5F9-G4 is well tolerated at 10 mg/kg weekly, with 1 mg/kg priming dose. Part B maintenance dose 20 mg/kg enrolling.

**Acknowledgements**

Stanford Clinical and Translational Research Unit; California Institute for Regenerative Medicine; Forty Seven, Inc.

**Trial Registration**

ClinicalTrials.gov identifier NCT02216409.

### P152 A pilot/phase II study evaluating pembrolizumab plus standard-of-care chemotherapy in metastatic triple-negative breast cancer, with companion comprehensive immunological monitoring

#### David Page^1^, Wendy Yu^2^, Alison Conlin^3^, Janet Ruzich^4^, Stacy Lewis^5^, Anupama Acheson^6^, Kathleen Kemmer^7^, Kelly Perlewitz^8^, Nicole M Moxon^3^, Staci Mellinger^3^, Carlo Bifulco^9^, Maritza Martel^3^, Yoshinobu Koguchi^10^, Bernard Fox^9^, Walter Urba^10^, Heather McArthur^11^

##### ^1^Earle A. Chiles Research Institute, Providence Portland Medical Center, Portland, OR, USA; ^2^Providence St. Vincent Medical Center, Portland, OR, USA; ^3^Providence Portland Medical Center, Portland, OR, USA; ^4^Providence Medical Group, Clackamas, OR, USA; ^5^Providence Medical Group, Portland, OR, USA; ^6^Providence Cancer Center, Portland, OR, USA; ^7^Oregon Health & Science University, Portland, OR, USA; ^8^Providence Medical Group, Newberg, OR, USA; ^9^Robert W. Franz Cancer Research Center, Earle A. Chiles Research Institute, Providence Cancer Center, Portland, Oregon, USA; ^10^Earle A. Chiles Research Institute, Providence Cancer Center, Portland, OR, USA; ^11^Memorial Sloan Kettering Cancer Center, New York, NY, USA

###### **Correspondence:** David Page (david.page2@providence.org)

**Background**

While sustained cytotoxic chemotherapy may be associated with lymphopenia and immunosuppression, chemotherapy may also facilitate antigen release/presentation, adaptive expression of PD-L1, and relative depletion of suppressive immune cell populations. Metastatic triple-negative breast cancer (MTNBC) is an aggressive and incurable disease associated with high mutational load and tumor-infiltrating lymphocytes, and is treated conventionally with sequential, sustained cytotoxic chemotherapy. In heavily pre-treated patients, anti-PD-1/L1 monotherapy yielded modest response rates of 9-19% [1]. However, we hypothesize that up-front treatment with 1^st^/2^nd^ line chemotherapy plus anti-PD-1/L1 might minimize immunosuppression (by treating patients before they are exposed to prolonged chemotherapy) and maximize immunostimulation (by treating less chemo-resistant disease and maximizing antigen release). In a preliminary cohort, anti-PD-L1 plus chemotherapy (nab-paclitaxel) was safe, with favorable response rates compared to historical controls [2].

**Methods**

In a pilot/phase II investigator-initiated trial, we will evaluate the safety and tolerability of anti-PD-1 (pembrolizumab 200mg IV every three weeks) plus investigator-selected 1^st^/2^nd^ line standard-of-care chemotherapy with either weekly paclitaxel (80mg/m^2^ IV) or oral capecitabine (2,000mg twice daily, weekly 1 on/1 off). Secondarily, we will evaluate efficacy of each combination employing a Simon 2-stage design, as measured by week 12 radiographic response. Because chemotherapy-associated immunosuppression could potentially influence immunotherapy efficacy, we will serially characterize general immune status and T cell activation using a validated real-time multi-parametric flow cytometry platform and peripheral blood T cell receptor sequencing (to measure clonal repertoire diversity). Via a companion biospecimen protocol, these data will be compared with data from MTBNC subjects receiving paclitaxel or capecitabine alone. Furthermore, baseline and post-treatment tumor-infiltrating lymphocytes will be characterized using multi-spectral immunofluorescence (using a validated panel including CD3, CD8, CD163, FOXP3, PD-L1, DAPI, and CK) and T cell receptor sequencing.

**Results**

As of 8/7/2016, five subjects have been registered for enrollment.

**Conclusions**

This investigator-initiated trial will provide important data, evaluating the efficacy of commonly used chemotherapies (paclitaxel or capecitabine) plus anti-PD-1, as well as evaluating the effect of these regimens on general immune status, peripheral T cell activation, and tumor infiltrating lymphocytes. A registrational MTNBC trial of nab-paclitaxel +/- anti-PD-L1 is ongoing.

**Trial Registration**

ClinicalTrials.gov identifier NCT02734290.

**References**

1. Emens LA, Kok M, Ojalvo LS: **Targeting the programmed cell death-1 pathway in breast and ovarian cancer**. *Curr Opin Obstet Gynecol* 2016, **28**:142-147.

2. Adams S, Diamond J, Hamilton E, *et al*: **Phase Ib trial of atezolizumab in combination with nab-paclitaxel in patients with metastatic triple-negative breast cancer (mTNBC)**. *ASCO Annual Meeting* 2016.

### P153 T cell therapy for patients with advanced ovarian cancer: a pilot study in progress

#### Magnus Pedersen^1^, Marie Christine Wulff Westergaard^1^, Troels Holz Borch^1^, Morten Nielsen^1^, Per Kongsted^1^, Trine Juhler-Nøttrup^2^, Marco Donia^1^, Inge Marie Svane^1^

##### ^1^Department of Oncology, Center for Cancer Immune Therapy, Herlev University Hospital, Herlev, Hovedstaden, Denmark; ^2^Department of Oncology, Herlev Hospital, Herlev, Hovedstaden, Denmark

###### **Correspondence:** Magnus Pedersen (magnus.pedersen@regionh.dk)

**Background**

Adoptive cell therapy (ACT) with tumor infiltrating lymphocytes (TILs) is based on infusion of activated and expanded cells isolated from autologous tumor tissue. The treatment has shown a clinical effect in approximately 50% of malignant melanoma patients with 20% obtaining a complete response [1]. Recent studies suggest that TIL based ACT can potentially be used with success in other cancers, including ovarian cancer (OC). OC is the 5^th^ leading cause of cancer death among women. If inoperable, the treatment is a combination of chemotherapy. Recurrent disease has a poor prognosis. The primary aim of this study is to assess feasibility and tolerability of T cell therapy for OC. Secondarily, to describe objective response using RECIST 1.1 and clarify if the treatment can induce measurable immune responses against tumor cells. This trial is presented at the European Society of Medical Oncology (ESMO) 2016 conference. In this abstract an update on clinical responses is provided.

**Methods**

Six patients will be included. Patients with progressive/recurrent OC and histologically verified serous adenocarcinoma are potential candidates. Surgical removal of tumor tissue for T cell expansion is performed. Stem cells are harvested for potential use if patients are having difficulties recovering from lymphodepleting chemotherapy. The treatment consists of lymphodepleting chemotherapy (60 mg/kg cyclophosphamide for 2 days, 25 mg/m^2^ fludarabine for 5 days) followed by T cell administration and high-dose decrescendo interleukin-2 for up to 5 days. Patients are evaluated for up to 5 years/until progression.

**Results**

Five patients have received treatment. The first had a partial metabolic response, stable disease (SD) with nearly 20% tumor regression and >50% reduction in CA-125 at 6 weeks, but progressive disease (PD) at 12 weeks. The second had SD at 6 weeks with a small decrease in CA-125, but PD at 12 weeks. The third had SD at 6 weeks with a 25% drop in CA-125, but PD at 12 weeks. The fourth had SD at 6 weeks with stable CA-125 and awaits 12 weeks evaluation. The fifth patient has not been evaluated yet. Only expected and manageable toxicities have been observed. All patients recovered without stem cell support. Immune analyses are pending.

**Conclusions**

So far, T cell therapy for patients with advanced OC seems to be manageable and tolerable.

**Trial Registration**

ClinicalTrials.gov identifier NCT02482090.

**References**

1. Rosenberg SA, Yang JC, Sherry RM, *et al*: **Durable complete responses in heavily pretreated patients with metastatic melanoma using T-cell transfer immunotherapy**. *Clin Cancer Res* 2011, **17(13)**:4550-4557.

### P154 Updated safety, efficacy, and pharmacokinetics (PK) results from the phase I study of BGB-A317, an anti- programmed death-1 (PD-1) mAb in patients with advanced solid tumors

#### Jayesh Desai^1^, Ben Markman^2^, Shahneen Sandhu^3^, Hui Gan^4^, Michael L Friedlander^5^, Ben Tran^6^, Tarek Meniawy^7^, Joanne Lundy^2^, Duncan Colyer^3^, Malaka Ameratunga^8^, Christie Norris^9^, Jason Yang^10^, Kang Li^10^, Lai Wang^10^, Lusong Luo^10^, Zhen Qin^10^, Song Mu^11^, Xuemei Tan^10^, James Song^11^, Michael Millward^7^

##### ^1^Royal Melbourne Hospital and Peter MacCallum Cancer Centre, Melbourne, Victoria, Australia; ^2^Monash Cancer Center, East Bentleigh, Victoria, Australia; ^3^Peter MacCallum Cancer Center, Melbourne, Victoria, Australia; ^4^Austin Hospital, Melbourne, Victoria, Australia; ^5^Prince of Wales Hospital, Sydney, New South Wales, Australia; ^6^Royal Melbourne Hospital, Melbourne, Victoria, Australia; ^7^Linear Clinical Research, Sir Charles Gairdner Hospital, Nedlands, Western Australia, Australia; ^8^Austin Health and Olivia Newton-John Cancer Research Institute, Melbourne, Victoria, Australia; ^9^The Prince of Wales Hospital, Randwick, New South Wales, Australia; ^10^BeiGene (Beijing) Co., Ltd, Beijing, People’s Republic of China; ^11^BeiGene (US) Co. Ltd., Fort Lee, NJ, USA

###### **Correspondence:** Zhen Qin (zhen.qin@beigene.com)

**Background**

BGB-A317 is a humanized IgG4 anti-PD-1 mAb with high specificity and affinity (K_D_=0.15 nM) for PD-1. It blocks PD-L1 and PD-L2 binding and inhibits PD-1-mediated negative signaling in T cell lines and tumor growth in a number of allogeneic xenograft models.

**Methods**

A phase I, multicenter study was conducted to evaluate the safety, tolerability, PK and antitumor activity of BGB-A317 in patients with advanced solid tumors. A 3+3 dose escalation design was undertaken. Patients received escalating doses of BGB-A317 intravenously at 0.5, 2, 5 and 10 mg/kg once every two weeks (Q2W). Additional patients were treated at 2 and 5 mg/kg once every three weeks (Q3W).

**Results**

As of 27 July 2016, 103 patients were treated across 4 dose-escalating cohorts of BGB-A317 Q2W (0.5 mg/kg, n=3; 2 mg/kg, n=6; 5 mg/kg, n=6 and 10 mg/kg, n=7) and 4 dose-expansion cohorts (2 mg/kg, Q2W, n=20; 2 mg/kg, Q3W, n=21; 5 mg/kg Q2W, n=20 and 5 mg/kg, Q3W, n=20). One DLT (1/6) of grade 3 colitis was observed in the 5 mg/kg Q2W dose-escalating cohort. Maximum tolerated dose was not reached. Recommended phase II dose is 5 mg/kg Q3W. The most common treatment-emergent adverse events (AEs) were grade (G) 1-2 fatigue (42%), nausea (30%), diarrhea (25%), abdominal pain (22%) and constipation (21%). Treatment–related G3 AEs included fatigue (n=2), hypotension (n=2), back pain (n=1), colitis (n=1), diabetes mellitus (n=1), diabetic ketoacidosis (n=1), dyspnea (n=1), elevated ALT (n=1), hyperglycaemia (n=1), hypoxia (n=1) and pneumonitis (n=1). Population PK analysis was conducted using 411 observed BGB-A317 serum concentrations from 31 patients who received doses of 0.5, 2, 5 and 10 mg/kg Q2W and 13 patients who received doses of 2 and 5 mg/kg Q3W. BGB-A317 PK is linear and the terminal elimination half-life is 16 days. Patients’ body weight is not a significant covariate on the clearance of A317. Among 99 evaluable patients, preliminary evidence of anti-tumor activities included 16 patients have partial responses (5 to be confirmed) and 20 patients exhibit stable disease. 13 responding patients remain on treatment, ranging from 18 to 38 weeks.

**Conclusions**

BGB-A317 demonstrates a favorable safety profile with AEs in keeping with the class effect. Early promising anti-tumor activity has been observed. BGB-A317 PK is linear and systemic clearance is not affected by body weight, which supports fixed dosing. The expanded phase IB study in selected cancer types is ongoing.

**Trial Registration**

ClinicalTrials.gov identifier NCT02407990.

**Fig. 61:**
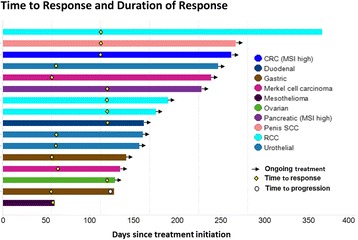
**(Abstract P154).** Sixteen patients demonstrated partial response (confirmed and unconfirmed), including 3 PRs in 5 urothelial carcinoma patients, 3 PRs in 9 RCC patients, 2 PRs in 4 gastric cancer patients, 2 PRs in 2 Merkel cell carcinoma patients, 1 PR in 1 colorectal cancer (CRC) patient with microsatellite instability high (MSI-h) status, among 12 CRC patients, 1 PR in 1 pancreatic cancer patient with MSI-h status, between 2 pancreatic cancer patients, 1 PR in 1 penis squamous cell carcinoma patient, 1 PR in 1 duodenal carcinoma patient, 1 PR in 22 ovarian cancer patients, and 1 PR in 7 mesothelioma patients

### P155 Preliminary safety data from a randomized multicenter phase Ib/II study of neoadjuvant chemoradiation therapy (CRT) alone or in combination with pembrolizumab in patients with resectable or borderline resectable pancreatic cancer

#### Matthew H G Katz^1^, Todd W Bauer^2^, Gauri R Varadhachary^3^, Nicolas Acquavella^4^, Nipun Merchant^4^, Gina Petroni^5^, Craig L Slingluff Jr.^6^, Osama E. Rahma^7^

##### ^1^Department of Surgical Oncology, Division of Surgery, The University of Texas MD Anderson Cancer Center, Houston, TX, USA; ^2^Department of Surgery, University of Virginia, Charlottesville, VA, USA; ^3^Department of Gastrointestinal (GI) Medical Oncology, Division of Cancer Medicine, The University of Texas MD Anderson Cancer Center, Houston, TX, USA; ^4^Sylvester Comprehensive Cancer Center, University of Miami, Miami, FL, USA; ^5^University of Virginia, Charlottesville, VA, USA; ^6^Division of Surgical Oncology, University of Virginia, Charlottesville, VA, USA; ^7^Dana-Farber Cancer Institute, Harvard University, Boston, MA, USA

###### **Correspondence:** Osama E. Rahma (osamae_rahma@dfci.harvard.edu)

**Background**

Tumor-infiltrating lymphocytes (TILs) do not reach the pancreatic cancer (PC) cells in significant numbers due to the presence of stroma and a suppressive microenvironment [1]. Neoadjuvant chemoradiation (CRT) can increase the presence of TILs in the PC microenvironment (PCME) [2]. We tested the combination of CRT and pembrolizumab for the first time in patients with pancreatic cancer in the neoadjuvant setting.

**Methods**

Patients with resectable or borderline resectable pancreatic cancer are randomized 2:1 to receive pembrolizumab intervenously every 3 weeks during concurrent CRT with capecitabine (825 mg/m2 orally twice daily, 5 days/week, on days of radiation only) and radiation (50.4 Gy in 28 fractions over 28 days) or Arm B to receive only concurrent CRT with capecitabine.

**Results**

**Fig. 62:**
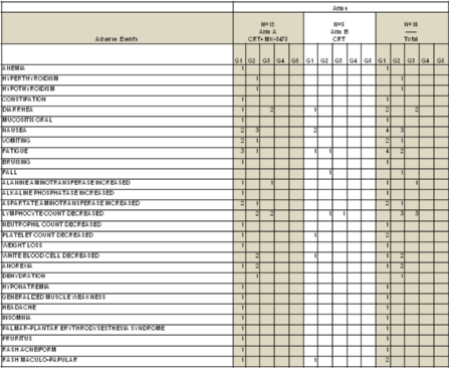
**(Abstract P155).** Maximum Grade Toxicities Related to Treatment

As of July 5, 2016 a total of 13 patients have been enrolled on the study (9 on Arm A and 4 on Arm B). Six patients had resectable disease (4 on arm A and 2 on arm B) while 7 patients had bordeline resectable disease (5 on Arm A and 2 on arm B). Post-neoadjuvant therapy, 2 patients were determined to have unresectable disease (both on arm B) and 4 patients underwent surgery (2 on each arm). Seven patients remain on neoadjuvant treatment. All grade toxicities related to treatment are summarized in Fig. 62. There were five grade 3 toxicities reported in Arm A: 2 patients had diarrhea attributed to CRT and resolved after holding the treatment; 2 patients had lymphopenia attributed to pembrolizumab or the combination; and one patient had elevated alkaline phosphatase related to the combination that met the definition of DLT and resolved after holding the treatment and receiving steroids. There was only one grade 3 toxicity reported on Arm B: lymphopenia attributed to CRT. There were no surgical complications reported within 30 days post-surgery in all patients who underwent surgery.

**Conclusions**

The study safety stopping boundaries have not been met and the study will continue enrolling patients.

**Trial Registration**

ClinicalTrials.gov identifier NCT02305186.

**References**

1. Kunk PR, Bauer TW, Slingluff CL, Rahma OE: **From bench to bedside a comprehensive review of pancreatic cancer immunotherapy.** *J Immunother Cancer* 2016, **15**:4-14.

2. Homma Y1, Taniguchi K, Murakami T, Nakagawa K, Nakazawa M, Matsuyama R, *et al*: **Immunological impact of neoadjuvant chemoradiotherapy in patients with borderline resectable pancreatic ductal adenocarcinoma.** *Ann Surg Oncol* 2014, **21(2)**:670-676.

### P156 KEYNOTE-426: randomized phase III study of pembrolizumab in combination with axitinib versus sunitinib monotherapy in treatment-naive advanced/metastatic renal cell carcinoma (mRCC)

#### Brian I. Rini^1^, Thomas Powles^2^, Mei Chen^3^, Yang Song^3^, Markus Puhlmann^3^, Michael B Atkins^4^

##### ^1^Cleveland Clinic Taussig Cancer Institute, Cleveland, OH, USA; ^2^Barts Cancer Institute, Queen Mary University of London, London, England, UK; ^3^Merck & Co., Inc., Kenilworth, NJ, USA; ^4^Georgetown-Lombardi Comprehensive Cancer Center, Washington DC, USA

###### **Correspondence:** Brian I. Rini (rinib2@ccf.org)

**Background**

Standard first-line treatments for mRCC include antiangiogenic agents sunitinib, pazopanib, and bevacizumab plus interferon, which yield a median progression-free survival (PFS) of approximately 10-11 months and median survival of 23-29 months. 5-year survival in mRCC remains low. Pembrolizumab, a programmed death 1 immune checkpoint inhibitor, has been tested in combination with axitinib, a vascular endothelial growth factor receptor inhibitor, in patients with treatment-naive mRCC. This combination has demonstrated manageable toxicity and promising clinical efficacy. The combination of pembrolizumab plus axitinib is being further tested for efficacy and safety in this open-label randomized phase III study, KEYNOTE-426 (ClinicalTrials.gov, NCT02853331), versus sunitinib monotherapy in patients with treatment-naive mRCC.

**Methods**

Key eligibility criteria include age ≥18 years, treatment-naive advanced/metastatic renal cell carcinoma with clear cell histology, measurable disease per RECIST v1.1, adequate organ function, and Karnofsky performance status ≥70%. Patients will be stratified per International Metastatic RCC Database Consortium risk category and geographic region, and then randomly assigned 1:1 to receive pembrolizumab 200 mg once every 3 weeks plus axitinib 5 mg twice daily or sunitinib 50 mg once daily for 4 weeks, then 2 weeks off. Treatment will continue until progressive disease, unacceptable adverse events, death, or withdrawal of consent. The primary end points are PFS per RECIST v1.1, as assessed by blinded independent central review, and overall survival. Secondary end points include objective response rate, duration of response, disease control rate, safety, and patient-reported outcomes. Approximately 840 patients will be enrolled globally.

**Trial Registration**

ClinicalTrials.gov identifier NCT02853331.

### P157 Translational studies identify ICOS agonist antibody JTX-2011 as a novel immunotherapy option for HNSCC patients

#### Sriram Sathyanaryanan^1^, Heather A Hirsch^1^, Jenny Shu^1^, Amit Deshpande^1^, Arun Khattri^2^, Jason Reeves^1^, Tong Zi^1^, Ryan Brisson^2^, Christopher Harvey^1^, Jennifer Michaelson^1^, Debbie Law^1^, Tanguy Seiwert^3^

##### ^1^Jounce Therapeutics, Cambridge, MA, USA; ^2^University of Chicago School of Medicine, Chicago, IL, USA; ^3^University of Chicago, Chicago, IL, USA

###### **Correspondence:** Sriram Sathyanaryanan (ssathy@jouncetx.com)

**Background**

ICOS (Inducible CO-Stimulator molecule) is a co-stimulatory molecule expressed primarily on T lymphocytes. Clinical data identified ICOS as a potentially key molecule in providing optimal anti-tumor benefit following anti-CTLA-4 therapy. JTX-2011 is an ICOS agonist antibody that will be entering early phase clinical development for the treatment of advanced solid tumors. JTX-2011 has a dual mechanism of action whereby it amplifies an immune response in T effector (Teff) cells while preferentially reducing the number of T regulatory cells (Treg).

**Methods**

An mRNA based gene-expression signature for ICOS expression and an IHC assay for ICOS and for T cell markers were developed. ICOS expression on intra-tumoral T cells and PD-L1 were analyzed in a cohort of 126 HNSCC patients collected at University of Chicago and in RNA sequencing data collected from The Cancer Genome Atlas (TCGA). ICOS expression was correlated with other immune signatures associated with response or resistance to anti-PD-1 therapy.

**Results**

Preclinical studies show that tumor models with the highest levels of intra-tumoral ICOS are most responsive to treatment with an ICOS agonist antibody. We hypothesize that tumors expressing high levels of ICOS would be expected to benefit from JTX-2011 treatment, we analyzed ICOS mRNA expression in ~10000 solid tumors across 30 indications. Highest levels of ICOS mRNA were observed in HPV+ and HPV- HNSCC. Flow cytometry showed highest expression of ICOS on intra-tumoral CD4+ Tregs, followed by CD4+ Teff and CD8+ T cells. We confirmed these results in a large set of clinical samples using a multiplex immunofluorescence (IF) assay. A wide range of ICOS cell density was observed in this dataset suggesting that identification of an ICOS “high” group may enrich for patients most likely to benefit from anti-ICOS targeted therapy. We further correlated ICOS expression to expression of PD-L1, a marker associated with response to anti-PD-1 therapy. Using IHC and gene-expression analyses, we identified a subset of patients that is less likely to respond to PD-1 therapy and more likely to respond to ICOS agonist therapy. In preclinical studies, treatment with anti-PD-1 therapy increased ICOS expression, suggesting that administration of JTX-2011 together with PD-1 blockade may provide an effective combination strategy.

**Conclusions**

These results support development of JTX-2011 in HNSCC patients expressing high levels of ICOS and suggest that combination of JTX-2011 with an anti-PD-1 agent may be an effective treatment paradigm.

### P158 KEYNOTE-183: randomized, open-label, phase III study of pembrolizumab in combination with pomalidomide and low-dose dexamethasone in patients with refractory or relapsed/refractory multiple myeloma

#### Jatin Shah^1^, Maria Victoria Mateos^2^, Morio Matsumoto^3^, Hilary Blacklock^4^, Albert Oriol Rocafiguera^5^, Hartmut Goldschmidt^6^, Shinsuke Iida^7^, Dina Ben Yehuda^8^, Enrique Ocio^2^, Paula Rodríguez-Otero^9^, Sundar Jagannath^10^, Sagar Lonial^11^, Uma Kher^12^, Patricia Marinello^12^, Jesus San-Miguel^9^

##### ^1^University of Texas MD Anderson Cancer Center, Houston, TX, USA; ^2^Complejo Asistencial Universitario de Salamanca/IBSAL, Salamanca, Castilla y Leon, Spain; ^3^National Hospital Organization, Shibukawa Medical Center, Gunma, Japan; ^4^Middlemore Hospital, Otahuhu, Auckland, New Zealand; ^5^Hospital Germans Triasi Pugoe, Barcelona, Spain; ^6^University Hospital Heidelberg, Heidelberg, Germany; ^7^Nagoya City University Graduate School of Medical Sciences, Nagoya, Japan; ^8^Hadassah Medical Center, Jerusalem, Italy; ^9^Clinica Universidad de Navarra, Pamplona, Navarra, Spain; ^10^The Mount Sinai Medical Hospital, New York, NY, USA; ^11^Winship Cancer Institute, Emory University, Atlanta, GA, USA; ^12^Merck & Co., Inc., Kenilworth, NJ, USA

###### **Correspondence:** Jatin Shah (jjshah@mdanderson.org)

**Background**

Response rates remain low for refractory or relapsed/refractory multiple myeloma (rrMM) treated with currently approved therapies, including proteasome inhibitors and immunomodulatory drugs (IMiDs). Lenalidomide reduces PD-L1 and PD-1 expression on MM cells and enhances checkpoint blockade–induced cytotoxicity; PD-1 blockade with pembrolizumab may thus act synergistically with IMiDs to enhance tumor suppression. In the phase I KEYNOTE-023 study, the combination of pembrolizumab + lenalidomide and low-dose dexamethasone was associated with an acceptable safety profile and a promising overall response rate (ORR) of 50% (20/40) in patients with rrMM, supporting further evaluation of pembrolizumab in combination with immunomodulatory agents. The randomized, open-label, multicenter, phase III KEYNOTE-183 study (ClinicalTrials.gov, NCT02576977) was designed to compare the efficacy and safety of pomalidomide and low-dose dexamethasone with or without pembrolizumab in patients with rrMM.

**Methods**

Key eligibility criteria include age ≥18 years, Eastern Cooperative Oncology Group performance status 0-1, confirmed diagnosis of rrMM with measurable disease, ≥2 lines of prior treatment, and documented progression on the last line of therapy. Prior therapies must have included an IMiD, such as lenalidomide or thalidomide, and a proteasome inhibitor, such as bortezomib, ixazomib, or carfilzomib. Patients are randomly assigned 1:1 to receive pomalidomide 4 mg daily on days 1-21 and low-dose dexamethasone 40 mg (20 mg for patients aged >75 years) daily on days 1, 8, 15, and 22 of repeated 28-day cycles, with or without pembrolizumab 200 mg every 3 weeks. Stratification is based on prior lines of treatment (2 vs ≥3) and disease status (refractory vs. sensitive to lenalidomide). Patients receiving pomalidomide must receive appropriate anticoagulation prophylaxis therapy. Treatment will continue until disease progression or unacceptable toxicity. Response will be assessed every 28 days by Clinical Adjudication Committee blinded central review and investigator review based on International Myeloma Working Group (IMWG) 2011 response criteria. Adverse events (AEs) will be assessed throughout treatment and for 30 days thereafter (90 days for serious AEs) and graded per NCI CTCAE v4.0. Patients will be followed for survival every 12 weeks. The primary endpoint is progression-free survival (PFS) based on IMWG criteria and overall survival; secondary endpoints are ORR, safety and tolerability, disease control rate, duration of response, and second PFS. Enrollment is ongoing and will continue until approximately 300 patients are enrolled.

**Trial Registration**

ClinicalTrials.gov identifier NCT02576977.

### P159 KEYNOTE-185: randomized, open-label, phase III study of pembrolizumab in combination with lenalidomide and low-dose dexamethasone in patients with newly diagnosed and treatment-naïve multiple myeloma (MM)

#### Jatin Shah^1^, Sagar Lonial^2^, Moacyr Ribeiro de Oliveira^3^, Habte Yimer^4^, Maria Victoria Mateos^5^, Robert Rifkin^6^, Fredrik Schjesvold^7^, Enrique Ocio^5^, Paula Rodríguez-Otero^8^, Jesus San-Miguel^8^, Razi Ghori^9^, Patricia Marinello^9^, Sundar Jagannath^10^

##### ^1^University of Texas MD Anderson Cancer Center, Houston, TX, USA; ^2^Winship Cancer Institute, Emory University, Atlanta, GA, USA; ^3^Jane Thompson Russell Cancer Care Center, Tacoma, WA, USA; ^4^Texas Oncology, Tyler, TX, USA; ^5^Complejo Asistencial Universitario de Salamanca/IBSAL, Salamanca, Castilla y Leon, Spain; ^6^Rocky Mountain Cancer Centers, Denver, CO, USA; ^7^Oslo University Hospital, Oslo, Norway; ^8^Clinica Universidad de Navarra, Pamplona, Navarra, Spain; ^9^Merck & Co., Inc., Kenilworth, NJ, USA; ^10^The Mount Sinai Medical Hospital, New York, NY, USA

###### **Correspondence:** Jatin Shah (jjshah@mdanderson.org)

**Background**

Lenalidomide in combination with low-dose dexamethasone is one of the standard-of-care treatments for MM. Preclinical and experimental data has shown that lenalidomide reduces PD-L1 and PD-1 expression on MM cells and enhances checkpoint blockade-induced cytotoxicity; thus, PD-1 blockade with pembrolizumab may act synergistically with lenalidomide to enhance tumor suppression. In the phase I KEYNOTE-023 study, the combination of pembrolizumab + lenalidomide and low-dose dexamethasone was associated with an acceptable safety profile and a promising overall response rate (ORR) of 50% (20/40) in patients with relapsed/refractory MM, supporting further evaluation of pembrolizumab in combination with lenalidomide. The randomized, open-label, phase III KEYNOTE-185 study was designed to compare the efficacy and safety of lenalidomide and low-dose dexamethasone with or without pembrolizumab in patients with newly diagnosed and treatment-naïve MM not eligible for transplantation.

**Methods**

Key eligibility criteria include age ≥18 years; newly diagnosed, treatment-naive, active MM with measurable disease; ineligibility for autologous stem cell transplantation; and Eastern Cooperative Oncology Group performance status 0-1. Patients are randomized 1:1 to receive lenalidomide 25 mg daily on days 1-21 and low-dose dexamethasone 40 mg (20 mg for patients aged >75 years) on days 1, 8, 15, and 22 of repeated 28-day cycles, with or without pembrolizumab 200 mg every 3 weeks. Stratification is based on age (<75 vs ≥75 years) and International Staging System (ISS) stage (ISS I or II vs ISS III). Treatment will continue until disease progression or unacceptable toxicity. Response will be assessed every 28 days by Clinical Adjudication Committee blinded central review and by investigator review based on International Myeloma Working Group (IMWG) 2011 response criteria. Adverse events will be assessed throughout treatment and for 30 days thereafter (90 days for events of clinical interest) and graded per NCI CTCAE v4.0. Patients will be followed for survival every 12 weeks. The primary end point is progression-free survival (PFS) as assessed by central review according to IMWG criteria; secondary end points are overall survival, overall response rate, duration of response, second PFS, and safety and tolerability. Enrollment is ongoing and will continue until approximately 640 patients are enrolled.

**Trial Registration**

ClinicalTrials.gov identifier NCT02579863.

### P160 KEYNOTE-122: phase II study of pembrolizumab versus standard-of-care chemotherapy in platinum-pretreated, recurrent or metastatic nasopharyngeal carcinoma

#### Anna Spreafico^1^, Victor Lee^2^, Roger K C Ngan^3^, Ka Fai To^4^, Myung Ju Ahn^5^, Quan Sing Ng^6^, Ruey-Long Hong^7^, Jin-Ching Lin^8^, Ramona F Swaby^9^, Christine Gause^9^, Sanatan Saraf^9^, Anthony T C Chan^4^

##### ^1^Princess Margaret Cancer Centre, Toronto, ON, Canada; ^2^Li Ka Shing Faculty of Medicine, The University of Hong Kong, Pok Fu Lam, Hong Kong, People’s Republic of China; ^3^Queen Elizabeth Hospital, Hong Kong, People’s Republic of China; ^4^The Chinese University of Hong Kong, Hong Kong, People’s Republic of China; ^5^Samsung Medical Center, Sungkyunkwan University, Seoul, Republic of Korea; ^6^National Cancer Centre Singapore, Singapore, Singapore; ^7^National Taiwan University Hospital, Taipei City, Taiwan, People’s Republic of China; ^8^Taichung Veterans General Hospital, Taichung City, Taiwan, People’s Republic of China; ^9^Merck & Co., Inc., Kenilworth, NJ, USA

###### **Correspondence:** Anna Spreafico (anna.spreafico@uhn.ca)

**Background**

Current treatment for recurrent/metastatic nasopharyngeal carcinoma (NPC) that progresses on a platinum-based regimen includes monotherapy with gemcitabine, capecitabine, or docetaxel. Although these treatments are used in clinical practice, they have not been formally approved for second-line NPC. Prolonged exposure to Epstein-Barr virus (EBV) in NPC leads to increased expression of programmed death-1 (PD-1), resulting in suppressed T cell immunity and tumor surveillance. Pembrolizumab is a monoclonal anti-PD-1 antibody designed to block the interaction between PD-1 and its ligands, PD-L1 and PD-L2. In the phase Ib KEYNOTE-028 study, pembrolizumab was associated with an overall response rate of 22% (6/27) in mostly heavily pretreated (≥3 lines) patients with NPC. KEYNOTE-122 is a multicenter, open-label, randomized phase II study designed to evaluate the efficacy and safety of pembrolizumab monotherapy versus chemotherapy in patients with platinum-pretreated, recurrent or metastatic NPC.

**Methods**

Key eligibility criteria include age ≥18 years, histologically confirmed nonkeratinizing differentiated NPC (WHO Class II) or undifferentiated NPC (WHO Class III), metastatic or recurrent disease, EBV positivity determined locally or centrally by EBV-encoded small RNA *in situ* hybridization, previous treatment with platinum-containing regimen, ECOG performance status 0-1, and measurable disease per RECIST v1.1. Patients will be randomly assigned 1:1 to receive pembrolizumab 200 mg every 3 weeks (Q3W) or investigator’s choice of chemotherapy (capecitabine 1000 mg/m^2^ twice daily on days 1-14 of each 3-week cycle, gemcitabine 1250 mg/m^2^ once per week on days 1 and 8 of each 3-week cycle, or docetaxel 75 mg/m^2^ Q3W). Treatment will continue until disease progression, unacceptable toxicity, investigator decision, or 35 cycles of pembrolizumab. Patients who complete 35 pembrolizumab cycles or discontinue pembrolizumab following a complete response may receive an additional 17 pembrolizumab cycles upon disease progression. Response will be evaluated every 6 weeks for the first year of treatment and every 9 weeks thereafter per RECIST v1.1 by central imaging assessment. Adverse events (AEs) will be assessed throughout treatment and for 30 days thereafter (90 days for serious AEs) and graded per National Cancer Institute Common Terminology Criteria for Adverse Events, version 4.0. Upon disease progression, patients will be followed for survival every 12 weeks. Primary end points are overall survival and progression-free survival per RECIST v1.1 by central imaging assessment; secondary end points include objective response rate and duration of response per RECIST v1.1 by central imaging assessment. Enrollment is ongoing and will continue until approximately 160 patients have enrolled.

**Trial Registration**

ClinicalTrials.gov identifier NCT02611960.

### P161 CX-839-004: a phase I/II study of the safety, pharmacokinetics, and pharmacodynamics of the glutaminase inhibitor CB-839 combined with nivolumab in patients with renal cell carcinoma, melanoma, and non-small cell lung cancer

#### Elaine Lam^1^, Nizar M Tannir^2^, Funda Meric-Bernstam^2^, Ulka Vaishampayan^3^, Keith W Orford^4^, Chris Molineaux^4^, Matt Gross^4^, Andy MacKinnon^4^, Sam Whiting^4^, Martin Voss^5^

##### ^1^University of Colorado Denver, Aurora, CO, USA; ^2^University of Texas MD Anderson Cancer Center, Houston, TX, USA; ^3^Karmanos Cancer Institute, Detroit, MI, USA; ^4^Calithera Biosciences, South San Francisco, CA, USA; ^5^Memorial Sloan Kettering Cancer Center, New York, NY, USA

###### **Correspondence:** Sam Whiting (swhiting@calithera.com)

**Background**

T cells require glucose and glutamine for activation and proliferation. Tumor consumption of nutrients within the tumor microenvironment may contribute to localized immune suppression, termed a “metabolic checkpoint,” and selective inhibition of tumor metabolism may reverse this immunosuppression. CB-839 is a potent, first-in-class, oral inhibitor of glutaminase 1 that inhibits the tumor TCA cycle by blocking glutaminase 1-mediated conversion of glutamine to glutamate. *In vitro* studies of CB-839 demonstrated that blocking glutaminolysis in tumor cells increased extracellular glutamine and stimulated proliferation of T cells. In the CT26 syngeneic colon carcinoma model, combining CB-839 with a PD-1/PD-L1 inhibitor doubled the rate of complete regressions over the checkpoint inhibitor alone. Safety and preliminary evidence of activity of CB-839 as monotherapy and in combination with everolimus and paclitaxel were reported previously.

**Methods**

CX-839-004 is a phase I/II trial evaluating CB-839 + nivolumab in patients with clear cell RCC (ccRCC), melanoma (mel), and NSCLC. The primary objectives are to evaluate (i) safety and tolerability and (ii) anti-tumor effect of the combination. Secondary objectives include determining the MTD/RP2D of CB-839 in combination with standard dose nivolumab. Exploratory objectives include characterization of the pharmacodynamics of the combination and evaluation of biomarkers that may predict anti-tumor effect. Eligibility criteria include incurable metastatic or locally advanced ccRCC, mel or NSCLC previously treated with standard of care therapy, ECOG 0-1, and measurable disease by RECIST 1.1. In the phase I portion, a minimum of 6-9 patients will receive escalating doses of CB-839 orally BID and nivolumab 3 mg/kg IV on Days 1 and 15 every 28 days. Subsequent to determining the MTD or RP2D, patients will be enrolled into phase II cohorts as follows: Cohort 1 ccRCC checkpoint inhibitor naïve; Cohort 2 ccRCC with documented progressive disease (PD) or stable disease (SD) > 6 mo on nivolumab in most recent line of therapy; Cohort 3 ccRCC with no better than SD before documented PD on any checkpoint inhibitor in any prior line of therapy; Cohort 4 melanoma with documented PD on a PD-1/PD-L1 inhibitor in most recent line of therapy; Cohort 5 NSCLC with documented PD or SD > 6 mo on PD-1/PD-L1 inhibitor in most recent line of therapy. Tumor burden will be assessed approximately every 8 weeks by RECIST 1.1 and irRECIST. Adverse events will be graded per NCI CTCAE. Samples obtained for pharmacodynamic and biomarker analyses will include pre-treatment and on-treatment tumor biopsies, blood and plasma.

**Trial Registration**

ClinicalTrials.gov identifier NCT02771626.

### P162 KEYNOTE-365: multicohort, phase Ib/II study of pembrolizumab combination therapy in patients with metastatic castration-resistant prostate cancer (mCRPC)

#### Evan Y. Yu^1^, Haiyan Wu^2^, Charles Schloss^2^

##### ^1^Seattle Cancer Care Alliance, Seattle, WA, USA; ^2^Merck & Co., Inc., Kenilworth, NJ, USA

###### **Correspondence:** Evan Y. Yu (evanyu@uw.edu)

**Background**

Approved treatments for mCRPC include second-generation endocrine therapies (abiraterone and enzalutamide) and taxanes (docetaxel and cabazitaxel). These agents may increase programmed death ligand 1 (PD-L1) expression and/or facilitate release of neoantigens. Additionally, the PARP inhibitor, olaparib, has activity in patients with mCRPC who have DNA-repair defects. Pembrolizumab is an anti–programmed death 1 (PD-1) antibody that blocks the interaction between the immune checkpoint receptor PD-1 and its ligands. KEYNOTE-365 (ClinicalTrials.gov, NCT02861573) is an international, nonrandomized, multicohort, open-label phase Ib/II study evaluating the safety and response rate of pembrolizumab in combination with olaparib (cohort A), docetaxel + prednisone (cohort B), or enzalutamide (cohort C) in patients with mCRPC.

**Methods**

Key eligibility criteria include: histologically or cytologically confirmed adenocarcinoma of the prostate without small-cell histology, documented prostate cancer progression within 6 months preceding screening, and ongoing androgen deprivation with serum testosterone < 50 ng/dL. Cohort A will include patients previously treated with docetaxel (prior treatment with 1 other chemotherapy is allowed, as well as ≤2 second-generation hormonal manipulations); cohort B will include patients previously treated with either abiraterone acetate or enzalutamide (but not both) before chemotherapy; and cohort C will include patients previously treated with abiraterone acetate before chemotherapy. Patients will receive pembrolizumab 200 mg once every 3 weeks (Q3W) (all cohorts) and either olaparib 400 mg twice daily (cohort A), docetaxel 75 mg/m^2^ Q3W + prednisone 5 mg twice daily (cohort B), or enzalutamide 160 mg every day (cohort C). Treatments will continue until disease progression or unacceptable adverse events (AEs). There will be a maximum of 35 cycles of pembrolizumab (all cohorts) and 10 cycles of docetaxel + prednisone (cohort B). Patients who must discontinue a combination component may continue with the other drug until progression/unacceptable AEs. Response will be assessed by evaluating prostate-specific antigen (PSA) levels Q3W, and with radiologic imaging every 9 weeks for the first year and every 12 weeks thereafter. Primary endpoints are safety/tolerability and PSA response rate (decline of ≥50% from baseline measured twice at least 3 weeks apart). Secondary end points include time to PSA progression, radiographic progression-free survival based on investigator-assessed RECIST v1.1 per the Prostate Cancer Working Group 3 guidelines, and overall survival. Approximately 70 patients will be enrolled in each cohort.

**Trial Registration**

ClinicalTrials.gov identifier NCT02861573.

## Clinical Trials: Cutting-Edge (Completed Trials)

### P163 Final statistical analysis of a pilot trial of hu14.18-IL2 in patients with completely resectable recurrent stage III or stage IV melanoma

#### Mark R. Albertini^1^, Erik A Ranheim^2^, Jacquelyn A Hank^2^, Cindy Zuleger^1^, Thomas McFarland^1^, Jennifer Collins^3^, Erin Clements^4^, Sharon Weber^1^, Tracey Weigel^4^, Heather Neuman^1^, Greg Hartig^1^, David Mahvi^5^, MaryBeth Henry^1^, Jacek Gan^1^, Richard Yang^1^, Lakeesha Carmichael^1^, KyungMann Kim^1^, Stephen D Gillies^6^, Paul M Sondel^2^

##### ^1^University of Wisconsin, Madison, WI, USA; ^2^University of Wisconsin School of Medicine and Public Health, Madison, WI, USA; ^3^University of Wisconsin Carbone Cancer Center, Madison, WI, USA; ^4^Westchester Medical Center, New York Medical College, Valhalla, NY, USA; ^5^Department of Surgery, Medical University of South Carolina, Charleston, SC, USA; ^6^Provenance Biopharmaceuticals Corp., Carlisle, MA, USA

###### **Correspondence:** Mark R. Albertini (mralbert@wisc.edu)

**Background**

Phase I testing of the hu14.18-IL2 immunocytokine (IC), a mAb reactive with GD2 disialoganglioside, linked to IL2, in patients (pts) with melanoma showed immune activation, reversible toxicities, and a maximal tolerated dose of 7.5 mg/m^2^/day. Preclinical data in IC-treated tumor bearing mice with low tumor burden documented striking antitumor effects. The main goal of this study was to obtain pilot data with hu14.18-IL2 in subjects with advanced melanoma who achieved a complete response (CR) through surgery.

**Methods**

Pts with completely resectable recurrent stage III or stage IV melanoma were scheduled to receive 3 cycles of IC at 6 mg/m^2^/d IV over 4 hours on days 1, 2 and 3 of each 28-day cycle. Pts were randomized to surgical resection of all sites of disease either following (Group A) or prior to (Group B) IC cycle 1. Primary objectives were 1) to evaluate histological evidence of anti-tumor activity in terms of apoptosis/necrosis of hu14.18-IL2 and 2) to evaluate recurrence-free survival (RFS) and overall survival (OS). The secondary objectives were 1) to evaluate adverse events associated with treatment and 2) to evaluate biological endpoints.

**Results**

Twenty melanoma pts (11 recurrent stage III, 8 stage IV, one unknown primary) were randomized to Group A (11 pts) or B (9 pts). Two Group B pts did not receive IC due to persistent disease following surgery. Six of 18 IC-treated patients remained free of recurrence, with a median recurrence-free survival of 5.7 months (95% CI: 1.8-not reached). The 24-month RFS rate was 38.9% (95% CI: 17.5-60.0%). The median follow-up of surviving patients was 50.0 months (range: 31.8-70.4). The 24-month OS rate was 65.0% (95% CI: 40.3-81.5%). Twelve pts had evaluable tumor samples for GD2 analysis and 6/12 showed expression of GD2 on melanoma cells. There was no significant difference in RFS (p=0.791) or OS (p=0.567) based on GD2 expression. There was a significant difference in the change in C-reactive protein values from baseline to cycle 1, day 3 (p < 0.02). There were significant differences in the change in lymphocyte counts from baseline to post-baseline time points. Toxicities were similar to those previously reported for IC. Immunohistologic evaluations of resected tumors showed variable inflammation and tumor necrosis between pts and no clear differences between Groups A and B.

**Conclusions**

Prolonged tumor-free survival was seen in some melanoma pts at high risk for recurrence who were treated with IC.

**Trial Registration**

ClinicalTrials.gov identifier NCT00590824.

### P164 Cytokine production by intratumorally administered activated dendritic cells correlates with survival in a phase I clinical trial

#### Vivek Subbiah^1^, Ravi Murthy^1^, Lori Noffsinger^2^, Kyle Hendricks^2^, Marnix Bosch^3^

##### ^1^University of Texas MD Anderson Cancer Center, Houston, TX, USA; ^2^Cognate Bioservices, Inc, Hanover, MD, USA; ^3^Northwest Biotherapeutics, Bellevue, WA, USA

###### **Correspondence:** Marnix Bosch (marnix@nwbio.com)

**Background**

Activated, autologous dendritic cells (aaDC) can be used to induce anti-tumor immune responses. A unique method of applying aaDC is through intratumoral injection, where the tumor cells serve as the source of antigen required for an adaptive anti-tumor response. A local effect may also occur as a result of cytokine production by the injected DC which makes the tumor more susceptible to a pre-existing or an induced immune attack.

**Methods**

Forty patients with locally advanced or metastatic solid tissue cancers were treated in a dose escalation trial in which aaDC were injected percutaneously under image guidance into a single tumor. Subjects had a median of 3 tumors (range 1 – 5) and had received an average of 3.1 prior treatments. To generate the aaDC, autologous monocytes were converted *ex vivo* into DC which were then activated. All batches of DC were released based on pre-specified criteria which included immunophenotyping and a T cell-stimulation assay, as well as sterility and endotoxin levels. Cytokine levels produced by the activated DC during manufacturing were measured and patient outcomes were correlated to these expression levels.

**Results**

All three doses levels were well tolerated. The main adverse events related to treatment were grade 1 and 2 fevers. Twenty-one patients achieved stable disease (SD) 8 weeks after initiating treatment, and this was found to correlate with survival (p=0.01). Levels of certain cytokines, such as such IL-8 and IL-12 p40, and TNFα were substantially elevated *in vitro* and IL-8 and IL-12 p40 production were predictive of survival (p=0.001 and p=0.008, respectively). TNFα levels also correlated with SD at week 8 (p=0.01). More than 70% of patients tested were found to have significant T cell responses, and/or *de novo* or significantly enhanced PD-L1 expression in the tumor post treatment, with a trend towards improved survival (p=0.1).

**Conclusions**

Study outcomes such as stabilization of disease and survival correlated with high DC cytokine levels, in the absence of meaningful toxicity. The DCVax treatment may be mediated through direct cytotoxic effects, as well as modulation of the tumor microenvironment to increase tumor infiltration by T cells, and attraction of inflammatory cells to the tumor. The development of PD-L1 expression likely reflects an induced immune response.

### P165 Augmentation of tumor infiltrating CD8+ T cells and specific response to autologous tumor antigens in a phase I trial of *in situ* vaccination with CCL21 gene-modified dendritic cells

#### Jay M Lee^1^, Mi-Heon Lee^1^, Edward B Garon^1^, Jonathan W Goldman^1^, Felicita E Baratelli^1^, Dorthe Schaue^1^, Gerald Wang^1^, Frances Rosen^1^, Jane Yanagawa^1^, Tonya C Walser^1^, Ying Q Lin^1^, Sharon Adams^2^, Franco M Marincola^3^, Paul C Tumeh^1^, Fereidoun Abtin^1^, Robert Suh^1^, Karen Reckamp^4^, William D Wallace^1^, Gang Zeng^1^, David A Elashoff^1^, Sherven Sharma^5^, Steven M. Dubinett^1^

##### ^1^David Geffen School of Medicine at UCLA, Los Angeles, CA, USA; ^2^National Institutes of Health Clinical Center, Bethesda, MD, USA; ^3^Sidra Medical and Research Center, Doha, Qatar; ^4^City of Hope and Beckman Research Institute, Duarte, CA, USA; ^5^VA Greater Los Angeles Healthcare System, Los Angeles, CA, USA

###### **Correspondence:** Steven M. Dubinett (sdubinett@mednet.ucla.edu)

**Background**

Intratumoral (IT) infiltration by activated immune effector cells is associated with a significantly better prognosis, however, tumor-associated immune suppression commonly occurs in non-small cell lung cancer (NSCLC). CD8^+^ T cell or dendritic cell (DC) infiltration is an independent favorable prognostic indicator. CCL21 is a lymphoid chemokine that chemoattracts both lymphocytes and DC. Our preclinical studies revealed potent systemic antitumor immune responses following IT *in situ* vaccination with DC overexpressing CCL21. In clinical evaluation of this strategy, we investigated anti-tumor specific systemic immune responses and tumor-infiltrating CD8^+^ T cells (CD8^+^ TIL) in NSCLC patients in response to *in situ* vaccination via IT administration of autologous DC transduced with a replication-deficient adenoviral (Ad) vector expressing the secondary lymphoid chemokine (SLC/CCL21) gene.

**Methods**

In a phase I trial, sixteen stage IIIB/IV NSCLC subjects received two vaccinations (1 x 10^6^, 5 x 10^6^, 1 x 10^7^, or 3 x 10^7^ dendritic cells/injection) by CT- or bronchoscopic-guided IT injection (days 0 and 7). Immune responses were assessed by tumor antigen-specific peripheral blood lymphocyte induction of IFN-γ in ELISPOT assays. Tumor biopsies were evaluated for CD8^+^ T cells and tumor PD-L1 by immunohistochemistry.

**Results**

Twenty-five percent (4/16) of patients had stable disease at day 56 follow-up by RECIST criteria. Four possible vaccine-related grade 1 adverse events (AE) occurred in 3 patients with no clear association to dose or schedule; the AE included flu-like symptoms, blood-tinged sputum after each injection, nausea, and fatigue. ELISPOT assays revealed 38% (6/16) of patients had systemic responses against autologous tumor associated antigens (TAA). Tumor CD8^+^ T cell infiltration was induced in 54% of subjects (7/13; 3.4 fold average increase in the number of CD8^+^ T cells per mm^2^). Patients with increased intratumoral CD8^+^ T cells following vaccination showed significantly increased PD-L1 mRNA expression (p=0.02).

**Conclusions**

Intratumoral vaccination with Ad-CCL21-DC was well-tolerated and resulted in 1) induction of systemic tumor antigen-specific immune responses and 2) enhanced tumor CD8^+^ T cell infiltration accompanied by increased PD-L1 expression. DC-CCL21 *in situ* vaccination may be an effective approach to induce tumor CD8^+^ T cell infiltration in combination with checkpoint inhibitor therapy. This will be assessed in combination with PD-1/PD-L1 checkpoint inhibition.

**Acknowledgements**

Supported by NCI R21CA105705, VA 2I01BX000359-05, NIH/NCATS UL1TR000124, NIH/NCI K23CA131577, NIH NCI L30CA142223, NIH NCI 5K12CA076905, the Thoracic Surgery Foundation and the NCI Experimental Therapeutics (NExT) Program.

**Trial Registration**

ClinicalTrials.gov identifier NCT00601094.

### P166 Increased immune responses in melanoma patients pre-treated with CDX-301, a recombinant human Flt3 ligand, prior to vaccination with CDX-1401, a dendritic cell targeting NY-ESO-1 vaccine, in a phase II study

#### Nina Bhardwaj^1^, Philip Friedlander^2^, Anna C Pavlick^3^, Marc S Ernstoff^4^, Brian Gastman^5^, Brent Hanks^6^, Mark R Albertini^7^, Jason J Luke^8^, Tibor Keler^9^, Tom Davis^9^, Laura A Vitale^9^, Elad Sharon^10^, Patrick Danaher^11^, Chihiro Morishima^12^, Martin Cheever^13^, Steven Fling^13^

##### ^1^Tish Cancer Institute, Icahn School of Medicine at Mount Sinai, New York, NY, USA; ^2^Icahn School of Medicine at Mt Sinai, New York, NY, USA; ^3^NYU Perlmutter Cancer Center, New York, NY, USA; ^4^Roswell Park Cancer Institute, Buffalo, NY, USA; ^5^Cleveland Clinic, Cleveland, OH, USA; ^6^Duke Cancer Institute, Durham, NC, USA; ^7^University of Wisconsin, Madison, WI, USA; ^8^University of Chicago School of Medicine, Chicago, IL, USA; ^9^Celldex Therapeutics, Hampton, NJ, USA; ^10^Cancer Therapy Evaluation Program, National Cancer Institute, National Institutes of Health, Rockville, MD, USA; ^11^NanoString Technologies, Seattle, WA, USA; ^12^University of Washington, Seattle, WA, USA; ^13^Cancer Immunotherapy Trials Network, Fred Hutchinson Cancer Research Center, Seattle, WA, USA

###### **Correspondence:** Steven Fling (sfling@fhcrc.org)

**Background**

Patients with high-risk melanoma have a 20-60% recurrence rate with 5-year OS between 45% and 70%. We evaluated vaccination with CDX-1401, a fusion protein consisting of human monoclonal IgG1 antibody targeting the dendritic cell (DC) receptor DEC-205 linked to the full-length NY-ESO-1 tumor antigen, with or without pretreatment with CDX-301, a recombinant human Flt3 ligand (Flt3L), in a phase II, randomized study of subjects with resected Stage IIb-IV melanoma. The primary objective was to determine whether the immune response to NY-ESO-1 elicited by vaccination with CDX-1401 and Hiltonol® (Poly-ICLC, from Oncovir) is substantially increased by prior expansion of circulating dendritic cells (DC) with CDX-301 therapy. Prevention of disease recurrence was not a clinical endpoint for this study.

**Methods**

60 pts with resected melanoma were randomized to two cohorts. Cohort 1 (CH1) received CDX-301 pretreatment (25 mcg/kg SQ daily x 10 days) in the first 2 of 4 monthly cycles of vaccination with CDX-1401 (1 mg IC day 1) + poly-ICLC (2 mg SQ, days 1 and 2). Cohort 2 (CH2) received 4 monthly cycles of vaccine with CDX-1401 and poly-ICLC w/o prior CDX-301. Antigen-specific immune responses were measured by Elispot and ELISA; flow cytometry and T cell assays were performed to evaluate the effects on immune cell subsets.

**Results**

The combination regimens of Flt3L, DC targeted NY-ES0-1 and poly-ICLC were well tolerated. Substantial increases in innate immune cells (DCs, NK cells and monocytes) were elicited in Flt3L treated (CH1) patients and w Flt3L treatment was associated with significant increases in activated T cells. T cell responses were elicited in both cohorts but were elicited earlier in CH1 and the number of individual responders to NY-ES0-1 in CH1 was significantly greater than in CH2. In addition, anti-NY-ES0-1-specific T cell responses in CH1 were significantly more robust. Significant increases in antibody titer were noted in both cohorts, but the average peak titer was significantly higher in CH1 vs CH2. Additional flow analyses, gene expression profiling and functional phenotyping of antigen-specific T cells are in progress.

**Conclusions**

In melanoma patients, DC mobilization with Flt3L is safe and leads to substantially enhanced B and T cell responses to DC targeted vaccines.

**Trial Registration**

ClinicalTrials.gov Identifier NCT02129075.

**Fig. 63:**
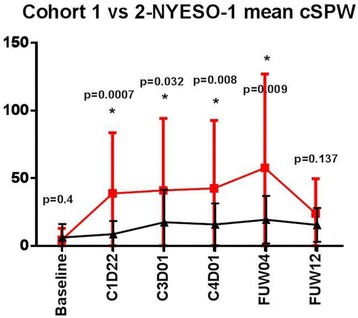
**(Abstract P166).** Anti-NY-ES0-1 specific responses in Cohort 1 are more robust and detected earlier vs Cohort 2. Differences between Cohort 1 and Cohort 2 at each time point are shown for Mean (of cohort) corrected SPW. Asterisks (*) indicate highly significant differences

### P167 A first-in-human phase I dose-escalation study of NHS-IL12 in solid tumors

#### Christopher R. Heery^1^, Joseph W Kim^2^, Elizabeth Lamping^3^, Jennifer Marte^3^, Sheri McMahon^3^, Lisa Cordes^3^, Farhad Fakhrejahani^3^, Ravi Madan^3^, Kwong Tsang^4^, Caroline Jochems^1^, Rachel Salazar^5^, Maggie Zhang^5^, Christoph Helwig^6^, Jeffrey Schlom^7^, James L Gulley^8^

##### ^1^Laboratory of Tumor Immunology and Biology, National Cancer Institute, Bethesda, MD, USA; ^2^Yale Cancer Center, New Haven, CT, USA; ^3^National Cancer Institute, Bethesda, MD, USA; ^4^Laboratory of Tumor Immunology and Biology, National Cancer Institute, NIH, Bethesda, MD, USA; ^5^EMD Serono, Billerica, MA, USA; ^6^Merck KGaA, Darmstadt, Hessen, Germany; ^7^Laboratory of Tumor Immunology and Biology, Center for Cancer Research, National Cancer Institute, Bethesda, MD, USA; ^8^Genitourinary Malignancies Branch, Center for Cancer Research, National Cancer Institute, National Institutes of Health, Bethesda, MD, USA

###### **Correspondence:** Christopher R. Heery (heerycr@mail.nih.gov)

**Background**

NHS-IL12 (MSB0010360N; M9241), a tumor-targeting immunocytokine comprising 2 heterodimers of IL-12 fused to the H-chain of the NHS76 antibody, has affinity for single- and double-stranded DNA and is designed to target necrotic tissue and deliver IL-12 to tumor. In previous trials with human recombinant IL-12, clinical activity was limited by toxicity. The goal is to reduce toxicity associated with systemic IL-12 through highly specific delivery to the tumor.

**Methods**

A 3+3 dose-escalation study in patients with advanced solid tumors was designed to demonstrate the safety of NHS-IL12 while evaluating pharmacokinetic (PK) and pharmacodynamic (PD) effects. Dosing started at 0.1 mcg/kg, with a planned maximum DL9 (21.8 mcg/kg). Patients were admitted for PK/PD draws and 48-hour observation after the first 2 doses. Restaging evaluations were performed every 8 weeks using standard RECIST 1.1 and immune-related response criteria. An expansion cohort (n=10) was enrolled at the maximum-tolerated dose (MTD) for further PK/PD analysis.

**Results**

From 12/2011–05/2016, 58 patients enrolled in DLs 1–9. 22 patients enrolled in single-dose cohorts (SDC); 36 patients in multiple-dose cohorts (MDC; q4wk). In SDC, all patients completed treatment; there was 1 death unrelated to study treatment. In MDC, 32 patients discontinued due to: progression (20), adverse events (AEs, 4), alternative treatment (3), treatment-schedule conflict or withdrawal (4), and 1 death unrelated to study treatment. Median administrations in the MDC was 2.5 doses (range 1–22). Treatment-related AEs occurred in 47/58 patients (81%). Dose-limiting toxicities were observed in 1/6 patients on DL8 (16.8 mcg/kg, grade 3 alanine aminotransferase (ALT) elevation) and 2/6 patients in DL9 (21.8 mcg/kg, grade 3 aspartate aminotransferase (AST) and ALT elevation and lipase elevation), making the MTD 16.8 mcg/kg. 10 patients were enrolled in expanded DL8 at 16.8 mcg/kg. Most common AEs in DL8 (n=16) were decreased lymphocyte count (81.3%), increased AST (81.3%), decreased white blood cell count (75%), increased ALT (75%), and fever (62.5%). Most AEs were transient and resolved within 10 days. Most symptoms were controlled with NSAIDs or acetaminophen. Serum cytokines showed time- and dose-dependent changes for IFN-gamma, IL-10, IL-12P70, and TNF-a. Best overall response was stable disease in 15/30 evaluable subjects. Five patients stayed on study treatment ≥182 days.

**Conclusions**

NHS-IL12 administered s.c. q4wk was safe and well-tolerated with predictable adverse events. Analysis of paired tumor biopsies to determine intratumoral effects are ongoing. Updated data will be presented at the conference.

**Trial Registration**

ClinicalTrials.gov identifier NCT01417546.

### P168 Salvage intravesical Mycobaterium phlei cell wall-nucleic acid complex (MCNA) for BCG-unresponsive patients

#### Roger Li^1^, John Amrhein^2^, Zvi Cohen^3^, Monique Champagne^3^, Ashish Kamat^1^

##### ^1^University of Texas MD Anderson Cancer Center, Houston, TX, USA; ^2^McDougall Scientific Ltd., Toronto, ON, Canada; ^3^Telesta Therapeutics Inc., Saint-Laurent, PQ, Canada

###### **Correspondence:** Roger Li (rli4@mdanderson.org)

**Background**

A standardized definition of BCG-unresponsive disease has been recently agreed upon to assess the efficacy of salvage treatments for patients with bladder cancer recurrence despite intravesical BCG therapy. Previously, we have reported the results from a single-arm trial of intravesical MCNA treatment in patients who have previously failed BCG [1]. We now report the results of a retrospective analysis, looking at efficacy with attention paid to the specific category of BCG-unresponsive patients.

**Methods**

High-risk NMIBC patients with either papillary and/or carcinoma *in situ* (CIS) having failed intravesical BCG treatment were enrolled. BCG treatment failure was defined as cancer recurrence after at least 2 courses of BCG (at least 5 of 6 induction instillations and at least 2 of 3 maintenance instillations, or 2 induction courses). Patients received induction treatment with 6 weekly doses of 8 mg MCNA intravesically, followed up by maintenance treatment of 3 weekly instillations at months 3, 6, 12, 18 and 24. Treatment efficacy was evaluated by cystoscopy, urine cytology and biopsy, with disease-free status achieved when central review of biopsy reveals absent high grade disease.

**Results**

Of the 129 patients originally enrolled, 94 fit the newly defined BCG unresponsive criteria. The cohort consisted of 68 (72.3%) with CIS, with/without papillary tumor, and 26 (27.7%) with exclusively papillary tumor upon recurrence after BCG. In the CIS-containing group, the complete response rate measured at months 6, 12, and 24 were 39.7% (28-52.3%), 23.5% (14.1-35.4%), and 13.2% (6.2-23.6%), respectively. In the group with only papillary tumors, the corresponding disease-free survival rates were 61.2% (38.2-7.8%), 61.2 (38.2-77.8%), and 50.1% (27.5-69%).

**Conclusions**

Intravesical MCNA therapy is an alternative therapy to radical cystectomy in the patients with recurring disease after intravesical BCG treatment. It has the potential to offer 24% of patients with CIS and 60% of patients with papillary tumors a chance to safely preserve their bladder.

**References**

1. Morales A, Herr H, Steinberg G, Given R, Cohen Z, Amrhein J, Kamat AM: **Efficacy and Safety of MCNA in Patients with Nonmuscle Invasive Bladder Cancer at High Risk for REcurrence and Progression after Failed Treatment with bacillus Calmette-Guerin.** *J Urol* 2015, **193**:1135-1143.

**Fig. 64:**
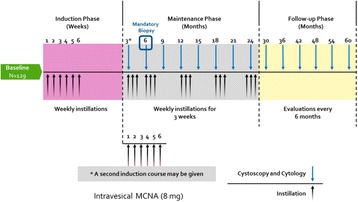
**(Abstract P168).** Study Design

**Fig. 65:**
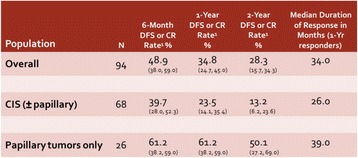
**(Abstract P168).** Disease Free Survival

## Coinhibition and Costimulation

### P169 DNA methylation changes in CD8+ T cells following 4-1BB costimulation

#### M. Angela Aznar^1^, Sara Labiano^1^, Angel Diaz-Lagares^2^, Manel Esteller^3^, Juan Sandoval^4^, Ignacio Melero^1^

##### ^1^Center for Applied Medical Research (CIMA), University of Navarra, Pamplona, Navarra, Spain; ^2^Bellvitge Biomedical Research Institute (IDIBELL), Barcelona, Catalonia, Spain; ^3^Bellvitge Biomedical Research Institute (IDIBELL), Barcelona, Navarra, Spain; ^4^Medical Research Institute La Fe, Valencia, Comunidad Valenciana, Spain

###### **Correspondence:** M. Angela Aznar (maznargo@alumni.unav.es)

**Background**

4-1BB costimulation imprints long term changes in the behavior of costimulated T cells [1]. There is not a satisfactory mechanistic explanation yet.

**Methods**

To determine the specific DNA methylation changes occurring upon 4-1BB costimulation, purified human CD8+ T cells from three healthy donors were activated *in vitro* for 5 days with anti-CD3 monoclonal antibody (OKT3) and either with anti-4-1BB monoclonal antibody urelumab or its corresponding isotype (huIgG4). Activated lymphocytes were left 5 days in culture with huIL-7 without further stimulation. Such back-to-resting CD8+ lymphocytes were reestimulated with OKT3 for 12, 24 and 36h to validate the expression of the genes differentially methylated upon primary stimulation at mRNA and protein levels. DNA methylation profiles of both activated and resting cell subsets were characterized with Infinium 450K DNA methylation array (Illumina). To further confirm our observations, identical experimental procedure was performed with anti-4-1BB 6B4 agonist antibody in a CD8+ T cell donor. Differentially methylated genes between OKT3+anti-4-1BB versus their corresponding control counterparts were validated by pyrosequencing on activated and resting CD8+ from independent group of healthy donors (n=8 for Urelumab and n= 11 for 6B4). Expression changes were confirmed by qRT-PCR and flow cytometry in activated, rested and reestimulated CD8+.

**Results**

853 genes were differentially methylated in urelumab-treated CD8+ T cells compared with their controls, 52 of which were shared with 6B4-costimulated CD8+ T lymphocytes. A number of differentially methylated genes are involved in i) T cell migration, ii) T cell activation, survival and homeostasis and iii) regulation of gene expression including key T cell transcription factors.

**Conclusions**

4-1BB costimulation induces CD8+ T lymphocytes that are poised to respond more effectively to a second antigen exposure. These acquired functions are imprinted in the genomic DNA of the CD8+ T cells by DNA methylation during 4-1BB co-stimulation, and involve key genes for CD8+ T cells.

**References**

1. Hendriks J, Xiao Y, Rossen JW, van der Sluijs KF, Sugamura K, Ishii N, Borst J: **During viral infection of the respiratory tract, CD27, 4-1BB, and OX40 collectively determine formation of CD8+ memory T cells and their capacity for secondary expansion**. *J Immunol* 2005, **175**:1665-1676.

### P170 Novel tetravalent anti-GITR antibody is a potent anti-tumor agent in vivo

#### Susannah D. Barbee^1^, David I Bellovin^1^, John C Timmer^2^, Nebiyu Wondyfraw^1^, Susan Johnson^1^, Johanna Park^1^, Amanda Chen^1^, Mikayel Mkrtichyan^1^, Amir S Razai^2^, Kyle S Jones^2^, Chelsie Y Hata^2^, Denise Gonzalez^1^, Quinn Deveraux^2^, Brendan P Eckelman^2^, Luis Borges^1^

##### ^1^Five Prime Therapeutics, South San Francisco, CA, USA; ^2^Inhibrx, La Jolla, CA, USA

###### **Correspondence:** Susannah D. Barbee (susannah.barbee@fiveprime.com)

**Background**

Glucocorticoid-induced TNFR-related (GITR, TNFRSF18) is a member of the TNFR superfamily with pleiotropic T cell modulatory activity. In the mouse, GITR is expressed at high levels on regulatory T cells (T_reg_) and has been reported to antagonize their suppressive capacity, whereas it is expressed at moderate levels on conventional effector T cells (both CD4^+^ and CD8^+^) for which where it exerts stimulatory activity. Studies have indicated that GITR-targeting agents mediate anti-tumor control via two mechanisms: depletion and possibly suppression of T_reg_ and/or direct agonism of effector T cells.

**Methods**

We have developed a novel anti-GITR antibody with enhanced agonist activity using single-domain antibodies (sdAbs) in a multivalent format. A tetravalent anti-GITR agonist antibody induces NF-kB activation and T cell stimulation *in vitro* that is superior to a conventional bivalent antibody; multivalency confers agonist activity in the absence of Fc-mediated crosslinking. T_reg_-depleting activity is obtained with an Fc effector-competent format.

**Results**

The tetravalent antibody potently controls tumor growth *in vivo* following a single dose with activity as low as 0.08 mg/kg. Treatment reduces T_reg_ frequency, thereby altering the ratio to effector T cells within the tumor to create a favorable environment for an effective anti-tumor immune response. CD8^+^ T cell activation and proliferation is observed *in vivo*, and treatment confers long-term immunity to tumor re-challenge.

**Conclusions**

In summary, multivalent GITR agonist antibodies are a promising modality for the treatment of cancer and we are exploring candidates for clinical development.

### P171 Evaluation of AP-1 signaling by interleukin-13 in human glioblastoma cells

#### Rukmini Bhardwaj, Raj K Puri, Akiko Suzuki, Pamela Leland, Bharat H Joshi

##### Centre for Biologics Evaluation and Research (CBER), U.S Food and Drug Administration, Silver Spring, MD, USA

###### **Correspondence:** Rukmini Bhardwaj (rukmini.bhardwaj@fda.hhs.gov)

**Background**

Glioblastoma (GBM) is one of the deadliest and most aggressive forms of brain cancer with a median survival of ≤ two years. Previously, our lab has demonstrated that Interleukin-13 receptor (IL-13R) alpha2 (α2) is a novel tumor antigen and is overexpressed in ~78% of the GBM tumors. IL-13Rα2 can be targeted by cancer vaccines, chimeric antigen receptor modified T cells (CAR-T) and a chimeric fusion immunotoxin consisting of IL-13 and truncated *Pseudomonas* exotoxin (IL-13-PE). However, the significance and regulation of the IL-13/IL-13R axis is not completely understood in the context of cancer immunology and in particular IL-13 signaling through the IL-13Rα2 chain.

**Methods**

We characterized and confirmed IL-13Rα2 expression in GBM tumor cell lines and its biological function by performing RT-qPCR and testing their sensitivity to the cytotoxic effect of IL-13-PE. We examined the activation of AP-1 family of transcription factors after treating GBM cell lines; U251, A172, U87MG (IL-13R+) and T98G (IL-13R-) with IL-13. Five members of the AP-1 family (c-Jun, Jun-D, Jun-B, c-Fos and Fra-1) were studied by immunocytochemistry (ICC) after treating 40,000 glioma tumor cells with IL-13 (20ng/ml) for 30 minutes in a glass chamber slide. We used IL-2 treatment as a negative control, since these cell lines did not express IL-2 receptors.

**Results**

We observed an overexpression of IL-13Rα2 mRNA in three of four GBM cell lines (U251, A172 and U87MG), which also showed high sensitivity to IL-13-PE immunotoxin. By ICC analysis of tumor cells, we found that three members of AP-1 family (c-Jun, Jun-D and Fra-1) were activated only in IL-13Rα2 positive glioma cells when treated with IL-13. In contrast, IL-13Rα2 negative T98G cells did not show activation of any of the AP-1 members. Two other members of the AP-1 family, Jun-B and c-Fos, were not activated after treatment with IL-13. We also observed that the extent of immunostaining and percent positive cells for c-Jun and Fra-1 in IL-13Rα2 positive glioma cells were significantly higher than IL-13R negative cells (P< 0.001).

**Conclusions**

Our results show that IL-13 mediates signaling in IL-13Rα2 positive GBM cell lines through the AP-1 pathway by activating c-Jun, Jun-D and Fra-1. IL-13 did not activate any of the AP-1 family members in a receptor negative GBM cell line. These results indicate that IL-13Rα2 is a key player in the IL-13/IL-13Rα2 axis for initiating signal transduction through the AP-1 pathway in GBM tumor cells and may be a good target for immunotherapy.

### P172 Activation of liver-resident myeloid cells to produce IL-27 initiates 4-1BB hepatotoxicity

#### Todd Bartkowiak^1^, Ashvin Jaiswal^1^, Casey Ager^1^, Midan Ai^2^, Pratha Budhani^2^, Renee Chin^2^, David Hong^2^, Michael Curran^1^

##### ^1^Department of Immunology, University of Texas MD Anderson Cancer Center, Houston, TX, USA;^2^University of Texas MD Anderson Cancer Center, Houston, TX, USA

###### **Correspondence:** Michael Curran (mcurran@mdanderson.org)

**Background**

4-1BB agonist antibodies were the second T cell co-stimulatory agents to enter the clinic after αCTLA-4. Despite impressive efficacy against both hematologic and solid tumors and an ability to suppress adverse events associated with checkpoint blockade, their development has been stymied by poor understanding of their underlying mechanism and the resulting inability to separate off-target liver toxicity from on-target anti-tumor immunity. We sought to uncover the mechanisms by which 4-1BB agonist antibodies trigger hepatotoxicity in hopes of discovering approaches by which the anti-tumor and hepatotoxic effects could be separated.

**Methods**

C57BL6/J mice (Taconic) were treated every 3 days for 3 doses with the 4-1BB antibody 3H3 (250ug), αPD-1 (RMP1-14, 250ug), αCTLA-4 (9D9 or 9H10 100ug), or αCD40 (FGK4.5 100ug). For 4-1BB knockout reconstitution experiments, mice were sublethally irradiated and then transferred with 2x10^6^ splenocytes as on d-1. CCR5, CXCR3, CCR2, Ebi3, IL27Ra, MHCII, and B2m knockout mice and FoxP3-DTR and OT-1 transgenics were purchased from Jackson. Serum liver enzymes were read on day 16 by the MDACC mouse pathology core.

**Results**

We find that α4-1BB mediated liver damage initiates through stimulation of myeloid cells, followed by subsequent recruitment and activation of CD8 and CD4 T cells in the liver. Moreover, we show that the inflammatory cytokine IL-27 is essential in myeloid conversion of T cells into mediators of liver damage. Conversely, FoxP3+ regulatory T cells (Treg) act to suppress 4-1BB agonist induced liver inflammation, and liver pathology worsens significantly when they are ablated. Further, we show that concomitant CTLA-4 blockade ameliorates 4-1BB hepatotoxicity by expanding peripheral Tregs, but that this effect is lost with addition of αPD-1. Additional differences exist between the tumor and liver microenvironments, which may be exploited to selectively promote on target activation of 4-1BB by agonist antibodies in the future.

**Conclusions**

4-1BB activation of liver-resident myeloid cells promotes the subsequent recruitment and activation of T cells in an IL-27 dependent manner. These T cells mediate the liver pathology associated with 4-1BB antibodies unless their activity is suppressed by Tregs. Our results support the use of 4-1BB agonists in rational combinations, in particular with CTLA-4 blockade, which may expand Tregs in the liver to ameliorate α4-1BB mediated toxicities.

**Acknowledgements**

We thank the MD Anderson IRG program and DOD PRCRP grant CA140792 for funding. Dr. Robert Mittler kindly provided 4-1BB knockout mice and Drs. Aymen Al-Shamkhani, Martin Glennie, and Stephen Beers kindly provided LOB12 antibody.

### P173 Restoration of tumor-infiltrating lymphocyte function by panobinostat and tumor eradication with the combination of panobinostat and PD-1 blockade

#### William D Hastings^1^, Maria Pinzon-Ortiz^1^, Masato Murakami^2^, Jason R. Dobson^1^, David Quinn^1^, Joel P Wagner^1^, Xianhui Rong^1^, Pamela Shaw^3^, Ernesta Dammassa^2^, Wei Guan^1^, Glenn Dranoff^1^, Alexander Cao^4^

##### ^1^Novartis Institutes for BioMedical Research, Inc., Cambridge, MA, USA; ^2^Novartis Institutes for BioMedical Research, Inc., Werk Klybeck, Basel-Landschaft, Switzerland; ^3^Cellaria Biosciences, LLC, Cambridge, MA, USA; ^4^Bristol-Myers Squibb, Princeton, NJ, USA

###### **Correspondence:** Jason R. Dobson (jason.dobson@novartis.com)

**Background**

Tumor immunotherapy is a unique therapeutic modality in our fight against human cancers. The recent success of immune checkpoint therapies highlights the value and potential of this approach. Epigenetic regulation of tumor immunology is becoming a key area of investigation. Experimental data have linked HDAC-inhibition to the enhancement of tumor antigen expression and presentation, the activation of effector T and NK cells, and the suppression of T regulatory cells. These observations are confounded by the potential immune-inhibitory effects by HDAC-inhibitors on DC and T cell activation. Here, we have examined the immune modulatory effects of pan-HDAC-inhibitor, panobinostat (LBH589), and its interaction with a PD-1 antibody, in preclinical settings.

**Methods**

Panobinostat was tested *in vitro* in human peripheral blood mononuclear cell (hPBMC) cultures at concentrations from 5 nM to 500 nM. Next, panobinostat was examined *in vivo* as single agent and in combination with the PD-1 antibody, 1D2, in MC38, a murine syngeneic tumor model.

**Results**

Panobinostat restored IL-2, IFNg, and TNFa expression that was inhibited during T cell exhaustion. Elevation of tumor-infiltrating lymphocytes (TIL) was observed by both flow cytometry and immunohistochemistry. Of note, the proportion of CD8+ effector-memory cells was increased by panobinostat. Proteomic analysis of the treated MC38 tumors revealed increases in IFNg levels under panobinostat treatment. Molecular profiling of tumor samples by NanoString indicated that panobinostat treatment led to increases of MHC class I, II and invariant chain expression. This is coupled with inductions of chemokine and cytokine expression and increases in effector T cell functions as measured by Granzyme A and B expression. Finally, while panobinostat and PD-1 antibody each achieved some level of anti-tumor efficacy, the combination of panobinostat and PD-1 antibody achieved complete responses in 10 out of 10 mice tested. The tumor regression was durable as none of the treated mice had any recurrence of tumors more than 60 days after the cessation of treatment.

**Conclusions**

Our preclinical data point to a dichotomy of immune modulation by panobinostat. While it may be immune-suppressive during priming, panobinostat is immune-stimulatory under antigen-experienced, immune-exhaustive environment. With the latter more reflective of the tumor microenvironment, we saw evidence of panobinostat being immune-stimulatory on TIL in a preclinical setting, with induction of both TIL percentages as well as activity. The positive effects on TIL and the promising combination efficacy with PD-1 antibody *in vivo* support further testing of panobinostat as a possible immuno-oncology agent both in preclinical and clinical settings.

### P174 Imprime PGG, a soluble yeast β-glucan, is a systemically administered PAMP that activates DCs and supports T cell priming, showing synergy with cancer immunotherapies

#### Ross B. Fulton, Steven Leonardo, Kathryn Fraser, Takashi O Kangas, Nadine Ottoson, Nandita Bose, Richard D Huhn, Jeremy Graff, Jamie Lowe, Keith Gorden, Mark Uhlik

##### Biothera Pharmaceuticals Inc., Eagan, MN, USA

###### **Correspondence:** Ross B. Fulton (rfulton@biothera.com)

**Background**

Pathogen-associated molecular patterns (PAMPs) provide crucial activating signals to the immune system. Importantly, due to their potent ability to induce pro-inflammatory cytokines that can cause systemic toxicity, most PAMPs are limited to local administration such as subcutaneous or intratumoral injection. Imprime PGG (Imprime) is a soluble yeast β-1,3/1,6 glucan that has been administered intravenously (i.v) to >400 healthy volunteers and cancer patients with minimal adverse effects. Imprime has shown promising efficacy when combined with other therapeutic antibodies in multiple clinical trials. Imprime has been previously shown to have promising efficacy in combination with immune checkpoint inhibitors in pre-clinical mouse tumor models and is currently being explored clinically in combination with anti-PD-1 therapy. In data presented here, we sought to further understand how Imprime functions to link the innate and adaptive immune systems via dendritic cells (DCs) to induce T cell priming providing benefit to checkpoint inhibitor therapy.

**Methods**

To examine *in vivo* effects of Imprime, C57BL/6 mice were injected i.v. with 1.2 mg of Imprime. 16hr post treatment, lymph nodes (LNs) were harvested and mRNA levels were determined using the QuantiGene Plex platform (Affymetrix). To study Imprime’s effect on CD8 T cell priming, 1x10^5^ OT-I CD8 T cells were transferred into congenic hosts and immunized with H-2K^b^/OVA_257-264_ peptide +/- Imprime. Separately, healthy human volunteers were infused with 4mg/kg Imprime and serum cytokines/chemokines were examined using the Luminex platform.

**Results**

Following i.v. administration of Imprime in non-tumor bearing mice, Imprime rapidly bound resident and migratory DC subsets, caused DC maturation, and increased DC recruitment into LNs. Transcriptional profiling in LNs showed increased mRNA levels of chemokines important in immune cell trafficking, pro-inflammatory cytokines, and a strong type I interferon signature. Many of these chemokines were also increased in the blood of healthy volunteers, as was detection of Imprime binding to DCs. In congenic mouse recipients that were immunized with peptide +/- Imprime after transfer of OVA-specific OT-I CD8 T cells, those primed in the presence of Imprime demonstrated greater overall expansion and acquisition of effector functions than peptide alone. Imprime’s transcriptional profile and ability to enhance T cell priming was dependent on the C-type lectin receptor Dectin-1.

**Conclusions**

Together, these data demonstrate that Imprime acts as a unique i.v.-administered PAMP that primes the immune system and inspires a coordinated adaptive immune response. These qualities make Imprime an attractive candidate to synergize with cancer immunotherapies.

### P175 Functional characterization of CDX-1140, a novel CD40 antibody agonist for cancer immunotherapy

#### Laura A Vitale^1^, Thomas O'Neill^1^, Jenifer Widger^1^, Andrea Crocker^1^, Li-Zhen He^1^, Jeffrey Weidlick^1^, Karuna Sundarapandiyan^1^, Venky Ramakrishna^1^, James Storey^2^, Lawrence J Thomas^2^, Joel Goldstein^1^, Henry C Marsh^2^, Tibor Keler^1^

##### ^1^Celldex Therapeutics, Hampton, NJ, USA; ^2^Celldex Therapeutics, Needham, MA, USA

###### **Correspondence:** Joel Goldstein (jgoldstein@celldex.com)

**Background**

For the development of agonist antibodies that stimulate immune responses, a balance is required between sufficiently strong immune stimulation and the untoward effects of systemic immune activation. This is particularly true for CD40, a molecule expressed by antigen presenting cells, which is critical in the regulation of immune responses. Agonist CD40 antibodies are highly effective in preclinical tumor models either through direct interaction with CD40-expressing lymphomas, or indirectly through the activation of adaptive anti-tumor immunity. There are several agonist CD40 antibodies in clinical development, but limited data are available with regard to their clinical activity and safety profile. Data reported for the agonist anti-CD40 mAb CP-870,893 (Roche/Pfizer) [1] demonstrated clinical activity in cancer patients, but its low maximum tolerated dose (0.2 mg/kg) may impede the full potential of activating this pathway. We set out to develop a CD40 agonist antibody with strong immune stimulating properties and a safety profile that allows for systemic dosing.

**Methods**

Anti-CD40 monoclonal antibodies (mAbs) were generated by immunization of human Ig transgenic mice with recombinant and cell surface expressed human CD40. We selected hybridomas that produced human antibodies with an assay using a reporter cell line engineered to express CD40 and NFκB-responsive luciferase. The variable regions of lead antibodies that displayed differential activity were cloned in human IgG1 or IgG2 constant domains and expressed in CHO cells.

**Results**

From this panel, CDX-1140, a human IgG2 antibody, was selected for further development. CDX-1140 is a potent inducer of human B cell proliferation and activation, and similarly directly stimulates dendritic cells to upregulate costimulatory molecules and secrete cytokines. Importantly, CD40 stimulation with CDX-1140 is independent of the Fc domain of the antibody. *In vivo*, CDX-1140 has potent anti-lymphoma activity in xenograft models, and a pilot study in non-human primates has shown good pharmacodynamic and safety profiles. A summary of these functional activities will be presented.

**Conclusions**

These data support CDX-1140 as a novel CD40 agonist with potential for immunotherapy of cancer patients.

**References**

1. Vonderheide RH, *et al*: **Clinical activity and immune modulation in cancer patients treated with CP-870,893, a novel CD40 agonist monoclonal antibody.** *J Clin Oncol* 2007, **25**:876–883.

### P176 Releasing the breaks: quantitative cell-based bioassays to advance individual or combination immune checkpoint immunotherapy

#### Jamison Grailer, Julia Gilden, Pete Stecha, Denise Garvin, Jim Hartnett, Frank Fan, Mei Cong, Zhi-jie Jey Cheng

##### Promega Corporation, Madison, WI, USA

###### **Correspondence:** Jamison Grailer (jamison.grailer@promega.com)

**Background**

Blockade of immune checkpoint receptors (e.g., PD-1, CTLA-4) has emerged as a promising new approach to enhance anti-tumor responses. While immunotherapies directed against PD-1 and CTLA-4 are showing unprecedented efficacy in the treatment of cancer, some patients and tumor types remain refractory to these therapies. This has resulted in a broadening of immunotherapy research and development to include additional inhibitory receptors (e.g., LAG-3, TIGIT, Tim-3) targeted individually or in combination with other immune checkpoint receptors. A major challenge in the development of biologics is access to quantitative and reproducible functional bioassays. Existing methods rely on primary cells and measurement of complex functional endpoints. These assays are cumbersome, highly variable, and fail to yield the data quality required for drug development. To address this need, we have developed a suite of immune cell line-based bioluminescent reporter bioassays for individual and combination immune checkpoint immunotherapy targets including PD-1 (PD-L1 or PD-L2), CTLA-4, LAG-3, TIGIT, PD-1+TIGIT and more.

**Methods**

These reporter-based bioassays were rationally designed to reflect the mechanism of action (MOA) of drug candidates targeting individual and combination immune checkpoint receptors. Each assay consists of an effector cell and an artificial antigen presenting cell (aAPC). Effector cell lines were engineered on a T cell background to express inhibitory receptor(s) and a luciferase reporter driven by specific response elements under the precise control of intracellular signals mediated by the T cell receptor (TCR) and inhibitory receptor target(s). The aAPCs were specially engineered to be able to activate the TCR in an antigen-independent manner and also stably express the ligand(s) of checkpoint receptor(s). These cell lines were developed in Thaw-and-Use format and can be used in assays without cell culture.

**Results**

Upon co-culture of effector cells with aAPCs, TCR activation in effector cells is suppressed by signaling from immune checkpoint receptor(s), which can then be specifically reversed by blocking antibodies targeting the inhibitory receptor(s) and/or their ligand(s). The bioassays were demonstrated to be specific to research-grade antibodies with known blocking activity as well as FDA-approved drugs (e.g., nivolumab, ipilimumab). In addition, the bioassays are pre-qualified according to ICH guidelines and demonstrate the performance required for use in antibody screening, potency testing and stability studies.

**Conclusions**

We have developed a suite of MOA-based bioassays for immune checkpoint receptors. These assays are easy to use and demonstrate high assay specificity, sensitivity and reproducibility. They are suitable for drug development in a quality-controlled environment.

### P177 Costimulatory T cell engagement by PRS-343, a CD137 (4-1BB)/HER2 bispecific, leads to tumor growth inhibition and tumor-localized CD8+ T cell expansion in a humanized mouse model

#### Marlon J. Hinner^1^, Rachida-Siham Bel Aiba^1^, Corinna Schlosser^1^, Thomas Jaquin^1^, Andrea Allersdorfer^1^, Sven Berger^1^, Alexander Wiedenmann^1^, Gabriele Matschiner^1^, Julia Schüler^2^, Ulrich Moebius^1^, Christine Rothe^1^, Olwill A Shane^1^

##### ^1^Pieris Pharmaceuticals, Inc., Freising, Bayern, Germany; ^2^Oncotest GmbH, Freiburg, Baden-Wurttemberg, Germany

###### **Correspondence:** Marlon J. Hinner (hinner@pieris.com)

**Background**

CD137 (4-1BB) is a key costimulatory immunoreceptor and a highly promising therapeutic target in cancer. To overcome limitations of current mAb-based approaches which monospecifically target CD137, we generated PRS-343, a CD137/HER2 bispecific designed to promote CD137 clustering by bridging CD137-positive T cells with HER2-positive tumor cells, thereby providing a potent costimulatory signal to tumor antigen-specific T cells.

**Methods**

We have previously described the generation of PRS-343 as a fusion of a CD137-specific Anticalin® protein to a variant of the HER2-targeting monoclonal antibody trastuzumab with an engineered IgG4 backbone. PRS-343 was found to efficiently activate T cells *ex vivo* in the presence of HER2-positive cells. Here, *in vivo* proof of concept data is presented utilizing a humanized mouse model in immunocompromised mice and the SK-OV-3 cell line as a HER2-positive xenograft. Tumor-bearing mice received human PBMC and weekly injections of PRS-343 for three weeks. An IgG4 isotype antibody served as the negative control, while a CD137-targeting benchmark antibody and trastuzumab with an engineered IgG4 backbone (“tras-IgG4”) served as controls for monospecific targeting of CD137 and HER2, respectively.

**Results**

PRS-343 activity was investigated at four different weekly doses, ranging from 4μg to 200μg. We found that PRS-343 dose-dependently led to strong tumor growth inhibition compared to treatment with the isotype control. Tumor response was accompanied by a significantly higher frequency of hCD45(+) tumor infiltrating lymphocytes (TIL) as determined by immunohistochemistry (IHC). Single IHC stainings against the T cell markers hCD3, hCD4 and hCD8 indicated that the rise in TIL frequency was due to an expansion of CD3+CD8+ T cells, while CD4+ lymphocytes remained at a low frequency both in the treatment and control groups. Interestingly, we observed neither tumor growth inhibition nor an increase in human TIL frequency with the anti-CD137 benchmark. The tras-IgG4 control was also devoid of lymphocyte infiltration into the tumor, but displayed a tumor growth inhibition comparable to PRS-343.

**Conclusions**

We report potent costimulatory T cell engagement of the immunoreceptor CD137 in a HER2-dependent manner, utilizing the CD137/HER2 bispecific PRS-343. PRS-343 displays dual activity *in vivo* based on monospecific HER2-targeting and bispecific, tumor-localized costimulation of CD137. Compared to known CD137-targeting antibodies in clinical development, PRS-343 has the potential to provide a more localized activation of the immune system with higher efficacy and reduced peripheral toxicity. The positive functional data of PRS-343 support investigation of its anti-cancer activity in clinical trials.

### P178 IL-2 signaling on tumor-infiltrating CD8+ T cells is not required for successful 4-1BB combination immunotherapy

#### Brendan Horton^1^, Stefani Spranger^2^, Thomas F Gajewski^3^

##### ^1^University of Chicago, Chicago, IL, USA; ^2^The Department of Pathology, The University of Chicago, Chicago, IL, USA; ^3^University of Chicago Medical Center, Chicago, IL, USA

###### **Correspondence:** Brendan Horton (blhorton@uchicago.edu)

**Background**

Antibodies against the PD-1/PD-L1 pathway have yielded impressive clinical results; however, not all patients benefit from these treatments, and many experience only partial responses. There is therefore a continued interest in developing new strategies to further boost anti-tumor immune responses and maximize therapeutic efficacy. CD8^+^ Tumor infiltrating lymphocytes (TIL) in both human and mouse tumors have been shown to express the co-stimulatory molecule 4-1BB. In addition to PD-1, 4-1BB agonists can induce tumor regression in pre-clinical models that is further boosted by anti-PD-L1 mAbs, but the detailed mechanism remains unclear.

**Methods**

We utilized the murine melanoma cell line B16, expressing the model antigen SIY, implanted subcutaneously into syngeneic C57BL/6 mice for these experiments.

**Results**

To determine if TIL already present in the tumor were sufficient for tumor regression, we utilized the S1P inhibitor FTY720. Therapeutic efficacy was preserved when anti-41BB combination immunotherapy was administered with FTY720, arguing it works directly on T cells in the tumor microenvironment. A markedly augmented number of antigen-specific CD8^+^ TIL occurred after combination immunotherapy even with FTY720 administration, arguing that antigen-specific TIL were expanded directly in the tumor microenvironment. To assess the mechanism of TIL accumulation, we performed BrdU labeling to measure proliferation, and detection of active caspase-3 to measure apoptosis. Unexpectedly, we found that proliferation of CD8^+^ TIL was not affected by combination immunotherapy. Instead, a significant decrease of active caspase-3 levels occurred in CD8^+^ TIL after immunotherapy, arguing that the accumulation of antigen specific TIL was due to decreased apoptosis. To further study the molecular mechanism of intratumoral TIL accumulation, we performed a gene expression profiling on untreated and immunotherapy-treated CD8^+^ TIL. Pathway analysis revealed that IL-2 and NF-kB were major hubs of differentially regulated genes. Consistently, we found increased IL-2 production in CD8^+^ TIL after immunotherapy. Therefore, we tested if T cell-intrinsic IL-2 signaling within the tumor site was required for successful immunotherapy. Intratumoral antibody-mediated blockade of IL-2 did not decrease the efficacy of combination immunotherapy. Mixed bone marrow chimeras of wild type (WT) and CD25^-/-^ bone marrow confirmed that there was no defect in CD25^-/-^ CD8^+^ TIL accumulation after immunotherapy.

**Conclusions**

These results suggest that restored IL-2 production by TIL is a marker of successful immunotherapy but is not required for therapeutic efficacy. Current studies are focusing on the role of T cell-intrinsic NF-kB signaling in successful combination immunotherapies that utilize agonist 4-1BB antibodies.

### P179 Dual function STAT3 inhibitor (CpG-STAT3ASO) generates systemic antitumor immune responses resulting in eradication of bone-localized prostate tumors in mice

#### Dayson Moreira, Tomasz Adamus, Xingli Zhao, Piotr Swiderski, Sumanta Pal, Marcin Kortylewski

##### City of Hope, Duarte, CA, USA

###### **Correspondence:** Marcin Kortylewski (mkortylewski@coh.org)

**Background**

STAT3 transcription factor promotes prostate cancer progression and sustains potently immunosuppressive tumor microenvironment. STAT3 activity in both cancer cells and in tumor-associated immune cells is likely responsible for resistance of metastatic prostate cancers to current treatments, including immunotherapy.

**Methods**

We previously demonstrated that ligands for endosomal Toll-like Receptor 9 (TLR9), CpG oligonucleotides, allow for cell-selective delivery of therapeutics to TLR9^+^ myeloid immune cells and tumor-propagating cells in prostate cancers. Here, we describe new CpG-conjugated STAT3 antisense oligonucleotides (ASO), chemically modified for improved nuclease resistance (T_1/2_ = 106 h in human serum).

**Results**

CpG-STAT3ASOs are quickly and efficiently internalized by human (DU145, LN-TLR9) and mouse (Myc-CaP, Ras/Myc-driven RM9) prostate cancer cells as well as by TLR9^+^ immune cells, including polymorphonuclear myeloid-derived suppressor cells (PMN-MDCSs) derived from blood of prostate cancer patients. In contrast to STAT3ASO alone, the naked CpG-STAT3ASO was internalized by rapid scavenger receptor-mediated mechanism even at low concentrations. Correspondingly, CpG-STAT3ASOs showed improved potency of *STAT3* knockdown at mRNA and protein levels in target cells. As assessed by biodistribution studies in mice, the intravenous (IV) injections of fluorescently-labeled CpG-STAT3ASO^Cy3^ effectively targeted TLR9^+^ myeloid cells and cancer cells in organs, such as spleen and bone marrow. For efficacy studies, we used syngeneic (RM9) and xenotransplanted (LN-TLR9, DU145), castration-resistant prostate tumors implanted intratibially in mice. Repeated IV injections of 5 mg/kg CpG-STAT3ASO resulted in regression of bone-localized RM9 tumors while treatments using STAT3ASO alone or CpG-scrambled ODN failed to control tumor progression. Antitumor effects of CpG-STAT3ASO depended on combination of direct and immune mediated cancer cell killing as suggested by reduced antitumor efficacy in xenotransplanted tumor models in immunodeficient mice. Both STAT3ASO and CpG-STAT3ASO reduced STAT3 activity in tumor and tumor-associated immune cells, but only the latter resulted in tumor infiltration by neutrophils and T cells. These effects were associated with reduced PD-L1 expression on MDSCs and the reduced percentage of regulatory T cells. Our preliminary results using blood samples from prostate cancer patients’ suggest that CpG-STAT3ASO effectively alleviates tolerogenic effects of human PMN-MDSCs on T cell activity.

**Conclusions**

Overall, our strategy allows for two-pronged targeting of metastatic, castration-resistant prostate cancers using safer and more efficient reagents based on TLR9-targeted oligonucleotide delivery.

### P180 A nanoparticle based approach for optimizing T cell activation, signaling, and proliferation for adoptive immunotherapy

#### Alyssa Kosmides^1^, Kevin Necochea^2^, Jonathan Schneck^3^

##### ^1^Johns Hopkins School of Medicine, Baltimore, MD, USA; ^2^Johns Hopkins, Irvine, CA, USA; ^3^Johns Hopkins Medical Institute, Baltimore, MD, USA

###### **Correspondence:** Alyssa Kosmides (akosmides@jhmi.edu)

**Background**

Artificial antigen presenting cells (aAPCs) are platforms that present the two necessary signals for T cell activation. Traditionally, aAPCs have signaling molecules randomly dispersed on their surface, although data has shown the importance of nanoscale signal organization and co-stimulatory molecule choice on stimulation [1]. Traditional aAPCs are thus inefficient for studying the dynamics of T cell activation. Here, we have developed a novel platform where signal 1 and 2 molecules are on distinct nanoparticles that are co-clustered on the T cell surface within a magnetic field. By manipulating nanoparticle properties and the types of co-stimulatory molecules, we show the benefit of this platform as a tool to study T cell stimulation.

**Methods**

Iron-dextran or polystyrene particles where conjugated with signal 1 peptide-MHC and signal 2 anti-CD28 mAb either on the same or separate 30-4500nm particles. Murine CD8+ cells were stimulated with nanoparticles and a 0.2T magnetic field. Activation was measured by cell proliferation, cytokine secretion, and cytotoxicity.

**Results**

**Fig. 66:**
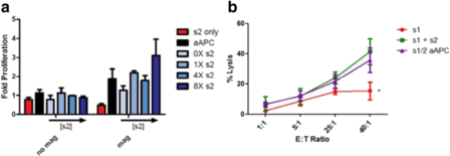
**(Abstract P180). a** TCR transgenic PMEL CD8+ T cells were stimulated for 7 days with Db-gp100 signal 1 only particles with increasing molar doses of anti-CD28 signal 2 (s2) only particles (i.e. 2X = 2:1 molar excess signal 2). aAPC refers to traditional signal 1 and 2 aAPCs. Cells were incubated in the presence or absence of a magnetic field for the first three days, and cell counts were taken on day 7. **b** PMEL CD8+ T cells were stimulated for 7 days with signal 1 (s1) only particles, signal 1 and 2 on separate particles (s1 + s2), or signal 1 and 2 on the same particle (s1/2 aAPC). After 7 days, T cell mediated killing of target B16-F10 cells was measured (*p<0.05 by two-way ANOVA)

**Fig. 67:**
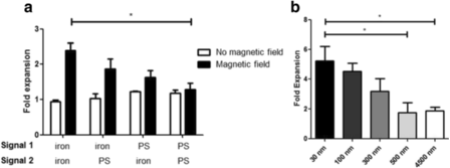
**(Abstract P180). a** Db-gp100 signal 1 and anti-CD28 signal 2 mAbs were conjugated to the surface of 100 nm iron dextran or polystyrene nanoparticles. PMEL CD8+ T cells were stimulated with different combinations of these particles in the presence or absence of a magnetic field, and cell counts were taken after 7 days (*p<0.05 by two-way ANOVA). **b** PMEL CD8+ T cells were stimulated with signal 1 and 2 on separate iron-dextran particles of increasing size within a magnetic field. Cell counts were taken after 7 days (*p<0.05 by one-way ANOVA)

We first showed that signal 1 and 2 can be separated onto distinct particles when these particles are clustered within a magnetic field (Fig. 66a). The resultant CD8+ T cells have equivalent cytotoxicity as compared to cells stimulated with traditional aAPCs (Fig. 66b). T cell activation is dependent on the co-clustering of both signaling molecules – when either is presented on a non-paramagnetic polystyrene particle, proliferation is decreased (Fig. 67a). The two signaling molecules also must be clustered sufficiently close, at the scale of tens of nanometers, as there was an inverse correlation between particle size and T cell proliferation (Fig. 67b). Finally, signal separation allows for greater enrichment of the clonal T cell subsets using signal 1 only particles, and thus greater activation over the same time period after the addition of signal 2 particles and a magnetic field. Signal separation also allows for easy optimization of signal 2 type and ratio (preliminary data not shown).

**Conclusions**

Here, we have demonstrated a novel T cell activation platform that involves simpler aAPC synthesis without sacrificing activation potential. This platform enables the study of the clustering of signaling molecules as well as easy manipulation of the types and ratios of different co-stimulatory signals.

**Acknowledgements**

AKK thanks the NSF (DGE-1232825), JHU INBT (2T32CA153952-06), and NCI (F31CA206344) for fellowship support.

**References**

1. Perica K, *et al*: **Magnetic Field-Induced T Cell Receptor Clustering by Nanoparticles Enhances T Cell Activation and Stimulates Antitumor Activity**. *ACS Nano* 2014, **8(3)**:2252-2260.

### P181 A secreted PD-L1 splice variant expressed across tumor types inhibits lymphocyte function

#### Kathleen M. Mahoney^1^, Sachet A Shukla^2^, Nikolaos Patsoukis^3^, Apoorvi Chaudhri^2^, Hung Pham^2^, Ping Hua^2^, Xia Bu^2^, Baogong Zhu^2^, Nir Hacohen^4^, Catherine J Wu^2^, Edward Fritsch^5^, Vassiliki A Boussiotis^3^, Gordon J Freeman^2^

##### ^1^Beth Israel Deaconess Medical Center, Dana-Farber Cancer Institute, Boston, MA, USA; ^2^Dana-Farber Cancer Institute, Boston, MA, USA; ^3^Beth Israel Deaconess Medical Center, Boston, MA, USA; ^4^Massachusestts General Hospital, Boston, MA, USA; ^5^Neon Therapeutics, Inc., Cambridge, MA, USA

###### **Correspondence:** Kathleen M. Mahoney (kmmah5@bidmc.harvard.edu)

**Background**

Targeting immune checkpoint pathways, such as programmed death ligand- 1 (PD-L1, also known as CD274 or B7-H1) or its receptor programmed cell death-1 (PD-1) have shown clinical benefit in patients with many different types of tumors. While PD-L1 expression is not a reliable biomarker for predicting response to therapy, the expression of the coinhibitory molecule PD-L1 on the surface of tumor cells is associated with worse prognosis in many tumors. This negative effect of PD-L1 expression is seen even when only a small fraction of the tumor expressed PD-L1 on the tumor cell surface. Here we describe a splice variant (secPD-L1) that does not splice into the transmembrane domain, producing a secreted form of PD-L1.

**Methods**

Lymphoma, kidney and breast cancer cell lines were analyzed for full length and secPD-L1 mRNA expression by qRT-PCR. RNASeq analysis in the Cancer Cell Line Encyclopedia confirmed expression of secPD-L1 in human tumor cell lines. RNASeq analysis of full-length PD-L1 and secPD-L1 was performed on The Cancer Genome Atlas, the GTEX database of non-malignant human tissue and sorted immune cells from healthy donors. Monocyte-derived dendritic cells were generated from healthy donors, stimulated with TNFa/PGE, polyIC, or LPS and assayed for expression of full-length PD-L1 and secPD-L1. Recombinant His-tagged secPD-L1 was produced to test whether it functioned as an inhibitor of proliferation and IFNg production in coactivation assays with T lymphocytes isolated from healthy donors.

**Results**

The secPD-L1 variant is expressed by cancer cell lines that also express the full-length PD-L1, as well as non-malignant immune cells particularly activated monocyte-derived dendritic cells. In The Cancer Genome Atlas, expression of secPD-L1 is found in primary tumors with higher expression of full-length PD-L1. Furthermore using recombinant secPD-L1 we found that secPD-L1 contains a unique 18 amino acid tail containing a cysteine and can dimerize, and inhibit T cell proliferation and IFN-gamma production *in vitro*.

**Conclusions**

This is the first report that a secreted splice variant of PD-L1 is expressed in a subset of many types of tumors, homodimerizes and is functionally active. Since secPD-L1 does not require cell-to-cell interaction to mediate its inhibitory effect, it may function as a paracrine negative immune regulator within the tumor microenvironment.

**Acknowledgements**

2014 AACR Basic Cancer Research Fellowship, Grant Number 14-40-01-MAHO, the 2014 ASCO Young Investigator Award supported by Kidney Cancer Association, and Claudia Adams Barr Program for Innovative Cancer Research (KMM); DF/HCC Kidney Cancer SPORE P50CA101942.

### P182 Combined targeting of OX40 and PD-L1 metabolically reprograms T cells promote regression of large established tumors

#### Amy E. Moran^1^, Fanny Polesso^1^, Lisa Lukaesko^2^, Andrew Weinberg^1^

##### ^1^Earle A. Chiles Research Institute, Providence Cancer Center, Portland, OR, USA; ^2^Oregon Health & Science University, Portland, OR, USA

###### **Correspondence:** Amy E. Moran (amy.moran@providence.org)

**Background**

The protective capability of tumor antigen specific T cells is regulated by both co-stimulatory and inhibitory signals in the tumor microenvironment. Current approaches in cancer immunotherapy seek to restore the function of unresponsive T cells by blocking inhibitory pathways. However, providing exogenous co-stimulatory signals are showing clinical promise and provide novel approaches for combination therapy.

**Methods**

Multiple solid tumor mouse models in multiple different mouse strains were used (MCA205/B6, CT26/Balb/C, d42m1T3/129) to determine the synergy of aOX40 (clone OX86) with aPD-L1 (clone 10F.9G2) in large, established tumors. Tumors were allowed to grow until they were ~50-70mm^2^ before treatment began. Animals were treated either alone or concurrently with 250μg aOX40 or 200ug aPD-L1 for a total of 500μg aOX40 and 600μg aPD-L1. Animals were euthanized and T cells isolated from secondary lymphoid organs and tumors. T cells were sorted for TCRβ chain sequencing based on Nur77GFP expression and or Foxp3RFP expression. TCRβ chain sequencing was performed by Adaptive Biotechnologies. T cell metabolic function was assessed using 2NBDG and TMRE as well as a Seahorse Analyzer. Phenotyping and functional assays were performed using traditional methods of flow cytometry.

**Results**

We demonstrate for the first time that agonist OX40 monoclonal antibodies metabolically reprogram CD4 Th1 skewed and CD8 effector T cells. In addition, using tetramers to track tumor-antigen specific CD8 T cells, we observe a significant expansion of effector memory CD8 T cells with enhanced cytotoxic function. Moreover, despite increased regulatory T cell (Treg) proliferation in the periphery, the CD8/Treg and CD4/Treg ratio is significantly increased in the blood and tumor of combination therapy treated animals. Furthermore, using a TCR signal strength reporter system, we observed the clonal expansion of a number of tumor infiltrating CD8 T cells that were actively receiving strong TCR signals *in situ*. Unlike aOX40 or aPD-L1 alone, combination immunotherapy focused the CD8 T cell repertoire response such that the 10 most frequent clones within the tumor made up almost 40% of the total CD8 T cell response.

**Conclusions**

Our findings suggest that concurrently targeting both positive (OX40) and negative (the PD-1/PD-L1 axis) T cell pathways can restore the function of failed tumor antigen specific T cells and promote tumor regression of well established tumors.

### P183 Characterization of infiltrating lymphocytes in benign, malignant, and healthy prostate tissue

#### Emelie Rådestad^1^, Lars Egevad^1^, Jonas Mattsson^1^, Berit Sundberg^1^, Lars Henningsohn^1^, Victor Levitsky^2^, Michael Uhlin^1^

##### ^1^Karolinska Institutet, Stockholm, Stockholms Lan, Sweden; ^2^Molecular Partners, Schlieren-Zurich, Zurich, Switzerland

###### **Correspondence:** Emelie Rådestad (emelie.radestad@ki.se)

**Background**

Prostate cancer (PC) is the second most common cancer type among men worldwide. Other non-malignant conditions of the prostate, such as benign prostatic hyperplasia (BPH), affect a majority of the older male population. Furthermore, chronic prostatic inflammation is common and is discussed to be a potential driver of benign and malignant prostate conditions. We aimed to compare the phenotype of lymphocytes present in prostates without pathologic changes with those infiltrating PC or BPH lesions, focusing on characterization of co-inhibitory receptor expression.

**Methods**

Lymphocytes were isolated from prostates of patients with PC (n=5), BPH (n=31), and deceased transplantation donors (n=5). Two tissue samples per PC patient were processed, one primary malignant and one adjacent non-malignant. Prostate-infiltrating lymphocytes were isolated using mechanical dissociation followed by density gradient centrifugation and flow cytometric analysis.

**Results**

Analysis showed that the majority of prostate-infiltrating T cells in all prostate conditions were CD8^+^ and had a CD45RO^+^CCR7^-^ effector memory phenotype. The ratio of CD4^+^/CD8^+^ T cells was comparable in all conditions, except BPH, where it was slightly higher than in healthy prostates. T cells expressing CD25 were more abundant in prostate tissue of PC patients (median 13.7% at malignant site, 15.1% at non-malignant site) and BPH (13.6%) compared to healthy controls (3.3%). Analysis of co-inhibitory receptor expression revealed that PD-1 was expressed by a larger proportion of T cells in PC specimens (71.0%, 60.0%) than in BPH (34.6%) and control prostates (47.2%). The opposite was found for LAG-3, which was expressed by a larger proportion of T cells in BPH (18.1%) than in PC (8.6%, 2.6%). Median frequency in control prostates was 13.1%. There were no differences between the sample types regarding frequencies of T cells expressing TIM-3 or CTLA-4, nor in quantity of Tregs or ϒδ T cells. Compared to peripheral blood, the frequency of Tregs was significantly higher in all prostate tissue types; 17.0%, 16.6%, 16.5% and 20.5% in control, BPH, malignant and non-malignant site compared to 7.2% and 5.4% in blood of BPH and PC patients.

**Conclusions**

Many prostate-infiltrating T cells seem to express co-inhibitory receptors LAG-3 and PD-1, regardless of prostate condition. Furthermore, presence of Tregs does not seem to be unique to the PC environment. However, we did find an increased frequency of T cells expressing PD-1 in PC lesions. It is of great importance to elucidate expression of co-inhibitory receptors for different solid cancers to narrow down patient groups that might benefit from different immunotherapies.

### P184 Understanding the therapeutic effectiveness of alloreactive immune responses in both murine and human acute myeloid leukemia during nonegraftment cellular therapy

#### William Rafelson^1^, John L Reagan^2^, Loren Fast^2^

##### ^1^Rhode Island Hospital, Warren Alpert School of Medicine, Brown University, Providence, RI, USA; ^2^Warren Alpert School of Medicine, Brown University, Providence, RI, USA

###### **Correspondence:** William Rafelson (william_rafelson@brown.edu)

**Background**

In stem cell transplant, few have studied the recipient lymphocytes’ recognition of mismatched donor antigens and MHC molecules, and how these primed host T cells may then recognize and kill leukemic cells. Previously, our group infused haploidentical, G-CSF mobilized, donor T cells into patients with refractory hematological malignancies preconditioned with 100cGy total body irradiation. Strong recipient immune responses, with some durable remissions, were seen in hematologic malignancies. In a second trial without G-CSF mobilization or recipient radiation, patients exhibited weaker inflammatory and anti-leukemic responses [1]. We therefore decided to characterize the alloreactive antileukemic response in a murine preclinical model with the AML leukemic cell line, C1498.

**Methods**

B6D2F1 mice were injected with C1498 leukemic cells and subsequently on day 7 were injected with haploidentical spleen cells from C57BL/6 mice. They were then monitored for signs of tumor progression and euthanized when they became moribund. T lymphocytes obtained from these euthanized mice were also tested for their ability to generate anti-C1498 responses when stimulated with haploidentical stimulator cells. We performed vitro experiments whereby healthy B6D2F1 splenocytes were stimulated with mitomycin-treated haploidentical spleen cells from a C57BL/6 mouse, and grown in mixed lymphocyte culture and tested on day 5 for their ability to lyse Cr51-labeled syngeneic blasts and C1498 leukemic target cells.

**Results**

Results of the murine model demonstrated the ability of alloreactive cytolytic T lymphocytes to lyse C1498 leukemic cells but not syngeneic blast cells. In addition, isolated CD3+ cells obtained from C1498 bearing mice were able to generate anti-C1498 lytic activity when stimulated with haploidentical cells *in vitro*. These responses are being explored further *in vivo*.

**Conclusions**

In previous trials, increased PD-1 expression in recipients following initial markers of T cell activation suggest that T cell exhaustion mediates leukemic cell survival. Increased PD-L1 expression on leukemic cells provides one explanation for their eluding the host inflammatory response. Total body irradiation may be disrupting the equilibrium of host-tumor tolerance. G-CSF mobilization of donor lymphocytes increases donor antigens for host recognition and subsequent alloreactivity. The murine model shows that alloreactive T cells can generate lysis specific to tumor but not self antigens. This model of alloreactive tumor response can be explored as a adjuvant therapy to other therapeutic approaches, including checkpoint inhibition.

**Acknowledgements**

NIH/NRSA funding is acknowledged in this study.

**References**

1. Reagan JL, Fast LD, Nevola M, *et al*: **Nonengraftment donor lymphocyte infusions for refractory acute myeloid leukemia**. *Blood Cancer J* 2015, **5**:e371.

### P185 First-in-class orally bioavailable checkpoint inhibitors targeting single and multiple immune inhibitory pathways

#### Pottayil Sasikumar, Naremaddepalli Sudarshan, Raghuveer Ramachandra, Nagesh Gowda, Dodheri Samiulla, Talapaneni Chandrasekhar, Sreenivas Adurthi, Jiju Mani, Rashmi Nair, Amit Dhudashia, Nagaraj Gowda, Murali Ramachandra

##### Aurigene Discovery Technologies Limited, Bangalore, Karnataka, India

###### **Correspondence:** Murali Ramachandra (murali_r@aurigene.com)

**Background**

Immune checkpoint inhibitors have changed the landscape of cancer therapy with the general acceptance that they will be the mainstay of future therapies. This is evident from a large number of ongoing clinical trials evaluating checkpoint inhibitors as a single agent or in combination with other therapeutic modalities. While these antibody-based therapies show impressive clinical activity, they suffer from the shortcomings including the failure to show responses in a majority of patients, immune-related adverse events (irAEs) due to the breaking of immune self-tolerance and need to administer by intravenous injection. The recent reports on severe demyelinating polyradiculoneuropathy leading to death observed in two patients with the anti-PD-1 immunotherapy also point to the need for short-acting agents for the better management of irAEs. Towards addressing these shortcomings, we are developing small molecule agents targeting one or more immune checkpoint pathways to increase the response rate and dosing by oral route with relatively shorter pharmacokinetic exposure.

**Methods**

We at Aurigene have devised a strategy to identify agents targeting single or multiple immune checkpoint proteins by taking advantage of the sequence/structural similarities among immune checkpoint ligands and receptors. In this strategy, high affinity shortest pharmacophore derived from the extracellular domain of checkpoint proteins are first identified and transformed into therapeutic agents with optimized drug-like properties. Our strategy has resulted in the identification of agents targeting PD-L1 alone, VISTA alone, PD-L1 and VISTA, and PD-L1 and TIM-3. The first compound from this approach AUPM-170/CA-170, a first-in-class dual antagonist targeting PD-L1 and VISTA, is undergoing clinical trials.

**Results**

Herein we report the pharmacological evaluation of another novel small molecule antagonist dually antagonizing PD-L1 and TIM-3 pathways. Potent functional activity comparable to that obtained with an anti-PD-1 or anti-TIM-3 antibody in rescuing T cell proliferation and effector functions was observed with the lead compound, AUPM-327. AUPM-327 showed selectivity against other immune checkpoint proteins as well as in a broad panel of receptors and enzymes. In preclinical models of melanoma, breast and colon cancers, AUPM-327 showed significant efficacy in inhibition of tumor growth upon oral dosing with excellent tolerability.

**Conclusions**

These findings support further development of AUPM-327 in the clinic.

### P186 A new target for immune checkpoint inhibition in urothelial carcinoma

#### Alexander Sankin, Benjamin Gartrell, Kerwin Cumberbatch, Hongying Huang, Joshua Stern, Mark Schoenberg, Xingxing Zang

##### Montefiore Medical Center, Bronx, NY, USA

###### **Correspondence:** Alexander Sankin (asankin@montefiore.org)

**Background**

Tumor cells evade immune surveillance by expressing cell surface ligands such as PD-L1 that are known to disrupt normal T cell function. Inhibition of these immunologic checkpoints has led to durable clinical remissions in metastatic melanoma, lung cancer, and bladder cancer [1-3]. We have recently described a new checkpoint family member HHLA2 that plays a significant role in inhibiting T cell activity [4]. We sought to characterize HHLA2 expression patterns in human urothelial carcinoma.

**Methods**

After obtaining IRB approval, we identified urothelial tumor cases from our institutional database and obtained paraffin-embedded tissue specimens originating from either transurethral resections (TUR) or radical cystectomies. A validated immunohistochemistry (IHC) protocol for HHLA2 staining has been previously described by the authors [4]. Tumor slides were examined by a single pathologist with no prior knowledge of patient clinical characteristics. Tumors were considered positive for HHLA2 expression (HHLA2+) if the number of positively stained tumor cells exceeded 1/100.

**Results**

**Fig. 68:**
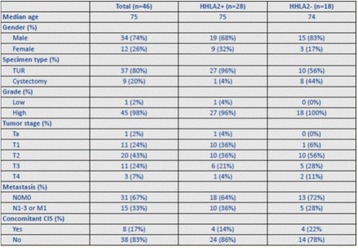
**(Abstract P186).** Clinicopathologic characteristics of patients by HHLA2 staining status

**Fig. 69:**
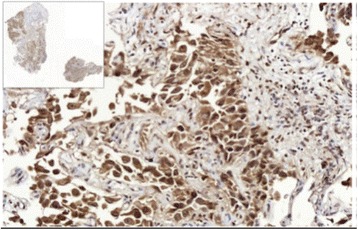
**(Abstract P186).** Example of a human urothelial tumor that expresses HHLA2

We identified 46 urothelial tumor specimens with adequate tissue for staining (Fig. 68). 37/46 (80%) specimens were obtained from TUR and 9/46 (20%) were obtained from radical cystectomy. Our cohort was enriched with aggressive tumor characteristics, specifically 45/46 (98%) were high grade, 34/46 (76%) were stage pT2 or higher, and 15/46 (33%) had metastases to lymph nodes and/or distant organs. HHLA2 was expressed in 28/46 (61%) tumors (Fig. 69).

**Conclusions**

This is the first study to demonstrate a robust expression pattern of the newly discovered immune checkpoint ligand HHLA2 in human urothelial tumors. Future studies will need to be conducted to further characterize the functional role of HHLA2 in bladder cancer immune evasion pathways and to develop therapeutic strategies to enhance immune mediated tumor destruction.

**References**

1. Brahmer JR, Tykodi SS, Chow LQ, Hwu WJ, Topalian SL, Hwu P, *et al*: **Safety and activity of anti-PD-L1 antibody in patients with advanced cancer**. *N Engl J Med* 2012, **366(26)**:2455-2465.

2. Powles T, Eder JP, Fine GD, Braiteh FS, Loriot Y, Cruz C, *et al*: **MPDL3280A (anti-PD-L1) treatment leads to clinical activity in metastatic bladder cancer**. *Nature* 2014, **515(7528)**:558-562.

3. Topalian SL, Hodi FS, Brahmer JR, Gettinger SN, Smith DC, McDermott DF, *et al*: **Safety, activity, and immune correlates of anti-PD-1 antibody in cancer**. *N Engl J Med* 2012, **366(26)**:2443-2454.

4. Zhao R, Chinai JM, Buhl S, Scandiuzzi L, Ray A, Jeon H, *et al*: **HHLA2 is a member of the B7 family and inhibits human CD4 and CD8 T-cell function**. *Proc Natl Acad Sci* 2013, **110(24)**:9879-9884.

### P187 Therapeutic T cell activation using engineered variant IgSF domains

#### Ryan Swanson, Michael Kornacker, Lawrence Evans, Erika Rickel, Martin Wolfson

##### Alpine Immune Sciences, Seattle, WA, USA

###### **Correspondence:** Ryan Swanson (ryan.swanson@alpineimmunesciences.com)

**Background**

The large number of immunoglobulin super family (IgSF) receptors on immune cells and tumors are attractive targets for the development of cancer immunotherapies. While nearly all commercial strategies targeting this family are focused on antibody-based biologics, we have developed a novel affinity ligand platform based upon yeast affinity maturation of human IgSF family extracellular domains (ECDs), which we term variant Ig Domains (vIgDs). vIgDs unique biochemical properties, including small size, monomeric/single domain structure, and capacity to interact with multiple counterstructures, combined with the near universal expression of IgSF family members and their counterstructures on immune cells and tumors, position the vIgD platform as an exciting new option for development of immuno-oncology biologics with first-in-class mechanisms of action.

**Methods**

IgSF domains of interest were mutagenized and cloned as yeast display libraries. Soluble counterstructure ligands were used to stain the display libraries, and yeast clones exhibiting the highest binding were isolated by flow cytometry sorting. vIgDs from the sorting outputs were subcloned into a mammalian Fc fusion expression vector, and individual vIgD-Fc fusions were recombinantly expressed in HEK293 cells. Purified preparations of vIgD-Fc fusions were used to stain eukaryotic cells transfected to surface express the target counterstructure. vIgD-Fc fusion proteins possessing desirable binding properties were tested for functional activity using *in vitro* assays with human primary T cells.

**Results**

Random and rationally designed mutant vIgD libraries were stained with counterstructure ligands and successfully sorted to isolate vIgDs with increased binding to the target counterstructures as demonstrated by elevated binding curves of successive selection outputs. vIgDs were expressed as recombinant Fc fusion protein constructs and demonstrated superior binding of transfectants expressing counterstructures compared to their parental wild-type IgSF domain Fc fusion protein. The resulting variants also demonstrated superior costimulatory activity when co-immobilized with anti-CD3 in human primary T cells.

**Conclusions**

Receptors built from IgSF domains are critical orchestrators of cellular communication in the immune system. However, IgSF receptors typically exhibit relatively low affinities for their target counterstructures, prohibiting their use as oncology therapeutics. Engineering of IgSF domains using yeast display affinity maturation allows the generation of variant proteins possessing superior binding to single or multiple native counterstructures. These binding improvements translate into beneficial alterations of functional activity, including improved costimulatory activity. The vIgD therapeutic platform is a new option for both development of soluble recombinant therapeutics or improvement of engineered cellular therapies.

### P188 Impact of BTK/ITK inhibition with ibrutinib on neuroblastoma and osteosarcoma syngeneic solid tumor models

#### Sandrine Valsesia-Wittmann^1^, Tala Shekarian^1^, François Simard^2^, Rodrigo Nailo^2^, Aurélie Dutour^2^, Anne-Catherine Jallas^1^, Christophe Caux^3^, Aurélien Marabelle^4^

##### ^1^Centre Leon Berard, Lyon, Rhone-Alpes, France; ^2^Centre Leon Berard CRCL UMR Inserm 1052/CNRS 5286, Lyon, Rhone-Alpes, France; ^3^UCBL1,Centre Leon Berard UMR Inserm 1052/CNRS 5286, Lyon, Rhone-Alpes, France; ^4^CRCL, UMR Inserm 1052/CNRS 5286, IHOP, Centre Léon Bérard, Gustave Roussy, Université Paris-Saclay, Lyon, Rhone-Alpes, France

###### **Correspondence:** Sandrine Valsesia-Wittmann (sandrine.wittmann@lyon.unicancer.fr)

**Background**

Ibrutinib, a covalent inhibitor of BTK, is currently revolutionizing B cell lymphoma treatment. Most solid tumors do not express BTK. However, BTK has been identified as a critical pathway in synthetic lethality assays in MYC dependent tumor cell lines. Interestingly, BTK can also be expressed in myeloid cells downstream to Fcg receptors. Also, ibrutinib inhibits interleukin-2-inducible T cell kinase (ITK), with potential ability to shift the balance from Th2 to Th1 T cells. Therefore, ibrutinib might have immunomodulatory effects in addition to its direct effects on cancer cells. Interestingly, aggressive MYCN amplified neuroblastoma (NB) are also highly infiltrated by myeloid cells. In osteosarcoma (OS), osteoclasts are derived from myeloid lineage. Thus, BTK blockade could be of double interest in NB and OS through both tumor- and immune-targeted effects.

**Methods**

We analyzed BTK expression by quantitative RT-QPCR and FACS analysis in different NB or OS cell lines and tumors from patients. We assessed the impact of ibrutinib on macrophage differentiation assays. *In vivo* anti-tumoral ibrutinib efficacy was evaluated in a mouse transplantable NB and rat orthotopic OS syngeneic models.

**Results**

High levels of BTK expression in some neuroblastoma tumor samples and variable levels of expression of BTK in patient tumor cell lines were observed. Interestingly, ibrutinib therapy had a positive impact on Neuro2A syngeneic NB models. No synergic effect of ibrutinib and anti-PD-L1 therapy could be obtained. In the rat OS model, ibrutinib had also therapeutic activity with a positive impact on growth of orthotopic bone tumors. However, we observed a negative impact of ibrutinib therapy on the number of spontaneous OS lung metastasis. Ibrutinib had no impact on the viability and differentiation of M1, M2 or Mo-DCs. However, it impaired human M1 macrophages differentiation towards a more immunosuppressive M2 phenotype with lower CD86 and CD163 expression, but no variation of HLA-DR and PD-L1. This negative effect was reversed by a TLR4 agonist.

**Conclusions**

Ibrutinib therapy has shown a positive therapeutic efficacy on the growth of primary tumors of a mouse NB and rat OS syngeneic models. However, in the OS model, ibrutinib therapy resulted in a higher number of spontaneous lung metastasis. This effect could be due to a negative impact of ibrutinib on anti-tumoral M1 macrophage differentiation. However this effect can be blocked by TLR4 stimulation suggesting that combinations of ibrutinib with TLR agonists might be of interested in solid tumors.

**Acknowledgements**

Pharmacyclics for providing ibrutinib.

